# Regio- and Stereoselective Halogenation by an Iron(II)- and 2‑Oxoglutarate-Dependent Halogenase in the Biosynthesis of Halogenated Nucleosides

**DOI:** 10.1021/jacs.5c16374

**Published:** 2025-12-16

**Authors:** Philip M. Palacios, Xiaoyun Li, Simahudeen Bathir Jaber Sathik Rifayee, Haoyu Tang, Tatyana Karabencheva-Christova, Christo Christov, Wei-chen Chang, Yisong Guo

**Affiliations:** † Department of Chemistry, 6612 Carnegie Mellon University, Pittsburgh, Pennsylvania 15213, United States; ‡ Department of Chemistry, 6798 North Carolina State University, Raleigh, North Carolina 27695, United States; § Department of Chemistry, 3968 Michigan Technological University, Houghton, Michigan 49931, United States

## Abstract

Iron­(II)- and 2-oxoglutarate-dependent (Fe/2OG) enzymes have garnered strong research interest in past decades due to their ability to catalyze regio- and stereoselective C–H functionalization via a single reactive intermediate, the oxyferryl species. In addition to the hydroxylation reaction that is commonly observed, other reaction outcomes have also been discovered in Fe/2OG enzymes. Among them, halogenation has attracted much research effort with the goal of revealing the molecular determinants to favor halogenation over hydroxylation; however, a full mechanistic picture is still missing. In this study, by investigating a recently identified Fe/2OG halogenase, AdeV, from the biosynthetic pathway of Adechlorin, we show, via biochemical, kinetics, and spectroscopic characterizations, that two oxyferryl intermediates are formed during the AdeV reaction in a sequential manner, which interconvert but only one shows kinetic competency to enable C–H activation and leads to the conversion of 2′-deoxyadenosine monophosphate (2′-dAMP) and 2′,3′-dideoxyadenosine monophosphate (ddAMP) to 2′-Cl-dAMP and 2′-Cl-ddAMP, respectively. By applying chemical synthesis and product characterization by detailed NMR analysis, the stereochemical assignment of the AdeV-catalyzed reaction is resolved, whereof the C–H bond cleavage and the C–Cl bond formation occur in a suprafacial manner. Using the experimental observations as a guide, the computational studies reveal that the kinetically competent oxyferryl intermediate structurally exhibits an offline configuration. However, this offline oxyferryl intermediate requires a structural conversion to a metastable inline configuration to perform a regio- and stereospecific C–H activation via a σ reaction channel. The subsequent conversion back to the offline configuration in the hydroxy-ferric state facilitates the final C–Cl bond formation.

## Graphical abstract

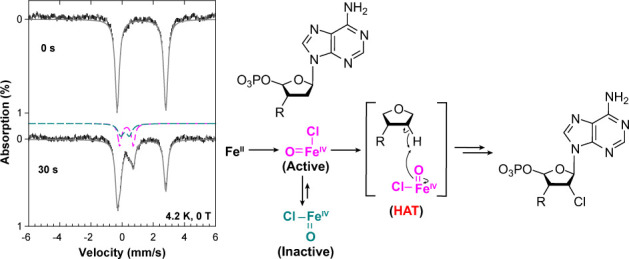

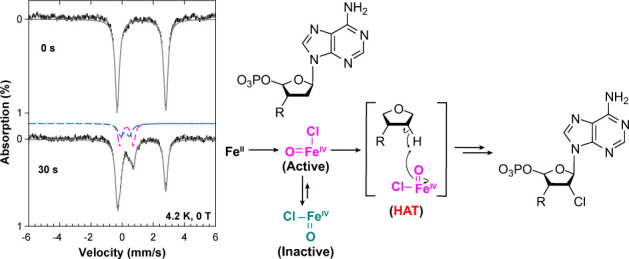

## Introduction

Metalloenzyme-catalyzed transformations are distinguished by their capacity to mediate regio- and stereoselective C–H bond functionalization, thereby enabling access to a repertoire of synthetically challenging reactions, including ring closure and expansion,^1–5^ cyclopropanation,^6–8^ aziridination,^9,10^ and halogenation,^11–17^ thus complementing synthetic methodologies for organic transformations. These biocatalytic reactions expand the accessible chemical space and have yielded diverse small molecules, many of which display significant pharmacological potential.^18^ However, limited mechanistic understanding often restricts the broad development of metalloenzymes as reliable tools for complex synthetic transformations. A notable example is the introduction of carbon–halide (i.e., Cl^–^ and Br^–^) bonds by iron­(II)- and 2-oxoglutarate-dependent (Fe/2OG) enzymes.^11–17^ Although these enzymes have demonstrated utility in the synthesis of both natural products and new-to-nature compounds,^19^ the lack of fundamental insight into the structure–reactivity relationship of the oxyferryl (ferryl, Fe­(IV)-oxo, or Fe^IV^=O) intermediate, the common reactive intermediate of these enzymes, remains a key barrier to advancing environmentally benign catalytic strategies for synthetic applications.

Several Fe/2OG halogenases have been discovered to catalyze chlorination and, in some examples, non-native anion transfer reactions.^11–17,19^ Among them, SyrB2, CytC3, and WelO5 identified in the biosynthesis of syringomycin E, γ,γ-dichloroaminobutyrate, and welwitindolinone have been subjected to detailed mechanistic studies,^20–27^ wherein the observation of C–H bond activation by a chloro-ferryl (Cl–Fe^IV^=O) species with a subsequent radical rebound from a chloro-ferric hydroxyl (Cl–Fe^III^–OH) intermediate has been recognized as a mechanistic paradigm and design principle for the development of radical transfer reactions to forge C–X (X = Cl^–^, Br^–^, N_3_^–^, or NO_2_^–^) bonds (Scheme 1). In addition, these halogenases also produce hydroxylated compounds as side products. Based on experimental observations and computational studies, various mechanistic models have been proposed to address the preference of halogenation over hydroxylation, including substrate-Fe-cofactor disposition (relative substrate positioning toward the Cl–Fe­(IV)=O or the Cl–Fe^III^–OH moieties),^23,28–31^ the C–H activation reaction pathways (σ-pathway vs π-pathway),^24–26^ the coordination flexibility of the Cl–Fe^IV^=O and/or the Cl–Fe^III^–OH intermediate (e.g., the inline vs the offline configurations, Scheme 1),^31–36^ the different intrinsic rebound reactivity of the Fe­(III)–OH bond and the Fe­(III)–Cl bond,^26,37^ and the secondary coordination sphere hydrogen bond interactions toward the hydroxyl ligand of the Cl–Fe^III^–OH intermediate.^17,27,38,39^ In addition, one of the computational studies has also proposed that CO_2_ formed during O_2_ activation may react with the hydroxyl group of the Cl–Fe^III^–OH intermediate to form carbonate, thereby preventing rebound hydroxylation.^40^ Despite the substantial studies on the reaction mechanism of Fe/2OG-dependent halogenation, several fundamental mechanistic questions remain. For example, in SyrB2 and CytC3, the existence of two chloro-ferryl species have been revealed by Mössbauer spectroscopy, which exhibit rapid equilibrium kinetics.^21,22^ So far, it is unclear whether both chloro-ferryl species are capable of C–H activation and whether these two species represent two structural isomers used to fine-tune the reaction outcomes (e.g., halogenation vs hydroxylation). However, a recent study on BesD, another halogenase that catalyzes 4-chloro-lysine formation,^17^ showed that it elicits only a single chloro-ferryl intermediate to enable both chlorination and hydroxylation.^41^ In addition, it is not known what the stereochemistry of the installed C–Cl bond is in the halogenation reaction and how it relates to the stereochemistry of the activated C–H bond. Addressing these questions is expected to provide critical yet still elusive mechanistic insights into the structure–function relationship of the chloro-ferryl intermediate during C–H activation and into the factors governing the chemical selectivity of the radical rebound step in halogenases.

**Scheme 1:**
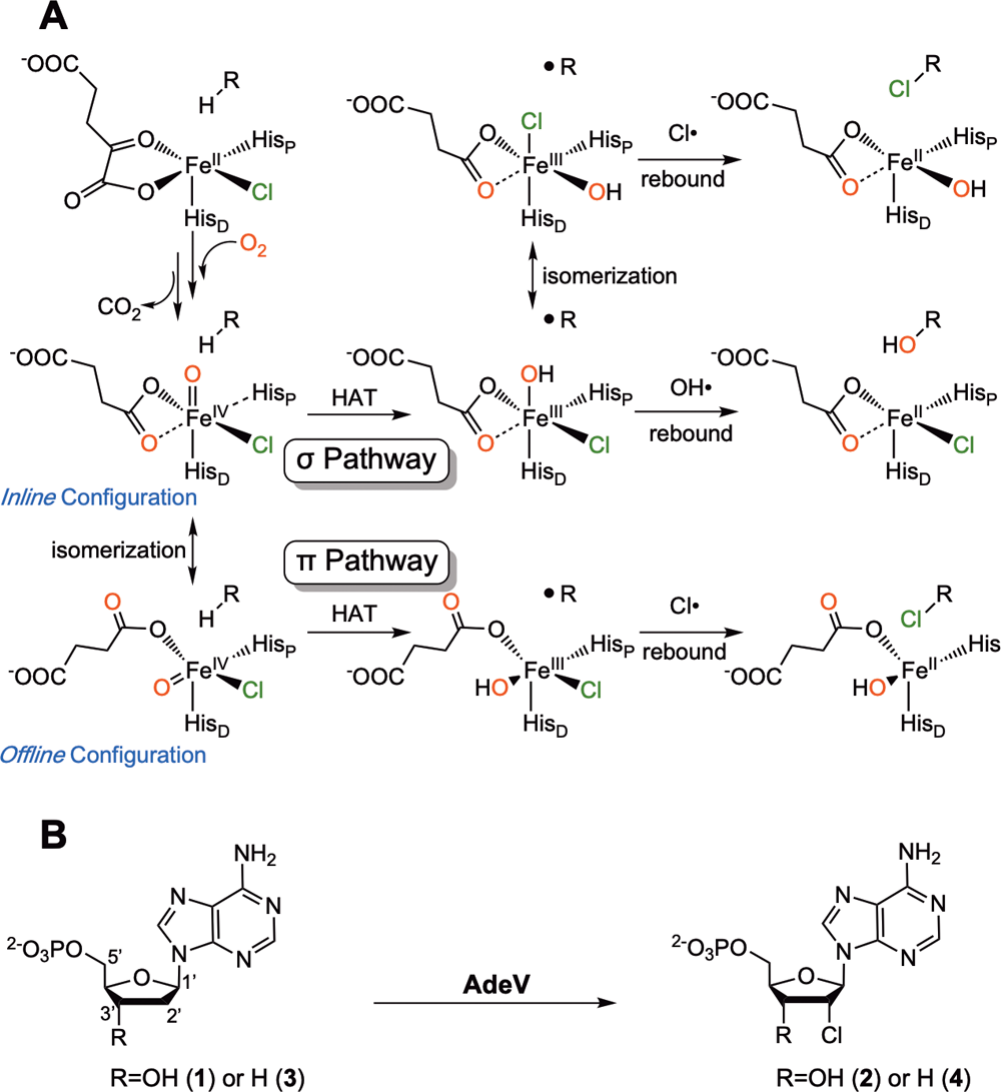
Current Understanding of the Reaction Mechanism of Fe/2OG Halogenase and the Overall Reaction Catalyzed by AdeV^a^

a (A) Current understanding of the reaction mechanism of Fe/2OG halogenase. The inline configuration of the chloro-ferryl intermediate features an Fe^IV^=O moiety *trans* to the histidine residue close to the C-terminus of the protein (His_D_), and the offline configuration features an Fe^IV^=O moiety *trans* to the histidine residue close to the N-terminus (His_P_). The hydrogen atom transfer (HAT) step could proceed via either a σ-pathway with a close-to-linear Fe^IV^=O···H angle or a π-pathway with an obtuse Fe^IV^=O···H angle. A structural isomerization at the chloro-ferric-hydroxo intermediate is also proposed. (B) Overall reaction catalyzed by AdeV.

In the current study, we use AdeV (or AdaV), a recently discovered Fe/2OG halogenase, to examine the aforementioned mechanistic conundrums. AdeV from *Actinomadura* sp. ATCC 39365 catalyzes the chlorination reaction on nucleoside substrates (Scheme 1).^42,43^ It converts 2′-deoxyadenosine monophosphate (2′-dAMP, **1**) to 2′-Cl-dAMP (**2**) and is involved in the biosynthesis of adechlorin, a rare halogenated nucleoside natural product. Herein, by product analysis via LC–MS and NMR, pre-steady-state enzyme kinetics, and Mössbauer spectroscopy, we show that two Cl–Fe^IV^=O intermediates form in a sequential fashion in which the early intermediate, termed Fe^IV^=O^first^, is exclusively used for C–H bond activation and is thus responsible for both chlorination as the major product and hydroxylation as the minor product. Fe^IV^=O^first^ converts reversibly to the other Cl–Fe­(IV)=O species (Fe^IV^=O^second^), which is long-lived and not involved in C–H bond cleavage. Importantly, C–H bond cleavage and C–Cl bond formation (both are at the C2′ position of the ribose ring) occurs in a suprafacial manner (retention of the stereochemistry). Note that this is the first time that the stereochemistry of the halogenation reaction by Fe/2OG enzymes is determined. By using these experimental observations as a guide, computational studies using MD and QM/MM reveal that the relative positioning between the Fe^IV^=O moiety of Fe^IV^=O^first^ and the substrate C–H bond is most likely in an offline fashion where the Fe^IV^=O bond is located perpendicular to the C–H bond. Indeed, only the Fe^IV^=O^first^ is capable of facile C–H activation and Fe­(III)–Cl rebound as well as Fe­(III)–OH rebound, but the Cl rebound outcompetes the OH rebound to direct the reaction flux to halogenation. This study provides key insights into the understanding of the chlorination mechanism of AdeV and setup foundation to elucidate governing factors that lead to nonhydroxylation outcomes in Fe/2OG enzymes.

## Results

### Observation of Two Chloro-Ferryl Species Appearing in a Sequential Fashion

AdeV was overexpressed in *E. coli*. The purification via metal affinity chromatography led to high-purity AdeV (Figure S1). The subsequent metal chelation, concentration, and gas exchange treatments rendered anaerobic apo-AdeV in high concentration (>2 mM in protein stock solution). The addition of an excess amount of 2OG (3.2 mM), Cl^–^ in the form of NaCl (63 mM), and substrate 1 (6.3 mM) to an anaerobic AdeV solution (0.35 mM) preincubated with ferrous (Fe^2+^) ion (0.32 mM) generated a pink species, which originated from a broad optical absorption feature centered at ∼510 nm with an additional absorption shoulder at ∼585 nm (Figure S2). This optical absorption feature has previously been assigned to the metal-to-ligand charge transfer (MLCT) between 2OG and Fe^44^ and is ubiquitously observed in Fe/2OG enzymes including Fe/2OG-dependent halogenases.

The stopped-flow absorption spectroscopic (SF-Abs) studies of the AdeV reaction were then performed. Rapidly mixing anaerobic AdeV•Fe^2+^•2OG•Cl^–^•**1** (0.25 mM AdeV, 0.23 mM Fe^2+^, 2.3 mM 2OG, 23 mM NaCl, and 12 mM **1**, all concentrations reported are values after mixing) with O_2_ saturated buffer (∼0.9 mM after mixing) leads to two major time-dependent optical absorbance changes (Figure 1 and S3): (1) the decay and the slow reformation of the Fe­(II)-2OG MLCT band with the representative wavelengths at 510 and 585 nm; (2) a gradual absorbance increase below 400 nm and centered at 310 nm that reaches a maximum after 10 s and shows only a slow decay after 500 s. The time-dependent absorbance change of this broad 310 nm feature (a formation followed by a decay) correlates with the time-dependent absorbance change of the Fe­(II)-2OG MLCT band (an initial decay followed by a reformation), especially of the 585 nm feature, suggesting that the 310 nm feature forms at the expense of the AdeV ferrous reactant complex upon reacting with O_2_. Thus, the 310 nm feature may be associated with the chloro-ferryl species that has also been observed in other halogenases (i.e., SyrB2,^22^ CytC3,^20,21^ and BesD^41^). However, its formation and decay kinetics are much slower in AdeV (detailed kinetics simulations have been carried out in conjunction with the Mössbauer data, see below) than those of other halogenases. In SyrB2 and CytC3, this feature completely decayed after 100 s, and in BesD, this feature completely decayed after 10 s.^21,22,41^ In addition, the time-dependent absorbance change at 510 nm showed an initial increase and was maximized ∼8 s before its eventual decay. The 510 nm feature mainly represents the Fe­(II)-2OG MLCT band of the AdeV ferrous reactant complex, but previous reports have also shown that the ferryl intermediates absorb in this wavelength region.^25,45,46^ Therefore, the initial absorbance increase upon O_2_ addition at 510 nm observed here is most likely due to the initial accumulation of the chloro-ferryl intermediate, which apparently exhibits a higher extinction coefficient than that of the Fe­(II)-2OG MLCT band at this wavelength.

**Figure 1:**
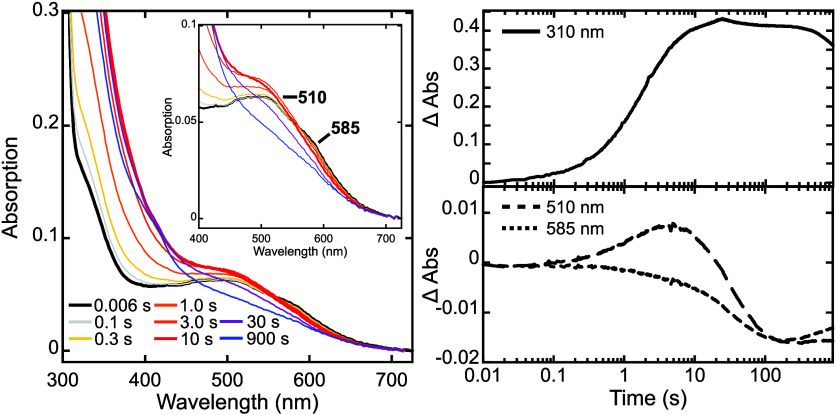
AdeV reaction with O_2_ was monitored by stopped-flow optical absorption spectroscopy. Left: time-dependent optical absorption spectra of the AdeV•Fe­(II)•2OG•Cl^–^•**1** complex reacting with O_2_ at selected reaction time points as shown. The inset shows the changes in the Fe­(II)-2OG MLCT band region. Right: time-dependent absorbance changes of the optical feature centered at 310 nm (top) along with the changes in the optical features at 510 and 585 nm (bottom).

To reveal the nature of the observed 310 nm optical feature and validate the SF-Abs results, we carried out freeze-quench (FQ) Mössbauer experiments to provide direct evidence of the chloro-ferryl species. Rapid mixing of the AdeV (1.1 mM AdeV•Fe­(II) complex) in the presence of 2OG (9.6 mM), Cl^–^ (25 mM), and **1** (6.7 mM) with O_2_-saturated buffer (∼0.9 mM) initiated the reaction. Subsequently, the reaction was quenched in liquid ethane cooled to 90 K at selected reaction times established by SF-Abs. A Mössbauer quadrupole doublet with Mössbauer parameters typical of high-spin ferrous species (δ = 1.24 mm/s, |Δ*E*_Q_| = 3.12 mm/s) was observed in the anaerobic AdeV reaction complex (Figure 2, left panel). At 1.0 s, a new quadrupole doublet was developed, which represents ∼20% of the total absorption of the spectrum (Figure S4). Concomitantly, ∼20% of the initial ferrous quadrupole doublet disappeared. This new doublet has parameters that are consistent with a high-spin (*S* = 2) Fe­(IV)-oxo species (δ = 0.23 mm/s, |Δ*E*_Q_| = 0.94 mm/s) reported in other Fe/2OG enzymes and closely resemble the parameters from one of the two chloro-ferryl intermediates observed in SyrB2 and CytC3 as well as the single chloro-ferryl species observed in BesD (Table S1).^21,22,41^ The observed Fe­(IV)-oxo (“Fe­(IV)=O^first^”) species reached a maximum accumulation of 45% at 10 s and decayed to 26, 13, 5, and <2% at 30, 60, 100, and 500 s, respectively (Figure 2, left panel, and Figure S4). Interestingly, at 30 s, a third quadrupole doublet that accounts for ∼12% of the total absorption was clearly observed. It slightly increased to 15% after 60 s and maintained a similar concentration level up to 500 s. This new doublet (“Fe­(IV)=O^second^”) has Mössbauer parameters (δ = 0.18 mm/s, |Δ*E*_Q_| = 0.55 mm/s) that fall within the range of an *S* = 2 Fe­(IV)-oxo species and are distinctly different from those of the first observed chloro-ferryl species (Table S1). Finally, the slow decay of the Fe­(IV)-oxo species led to the regeneration of resting Fe­(II) (δ = 1.24 mm/s, |ΔE_Q_| = 3.12 mm/s). Furthermore, we performed variable-field Mössbauer measurements. The spectra recorded under 7 T applied fields (parallel to γ radiation) clearly identified that both of the chloro-ferryl species exhibit an *S* = 2 spin state with the values of the principal components of ^57^Fe hyperfine coupling tensor (A tensor) falling in the general range of the previously reported *S* = 2 enzymatic ferryl intermediates (Figure 2 right panel and Tables S1 and S2). Specifically, Fe­(IV)=O^first^ exhibits *A*_*x*_ and *A*_*y*_ values that are slightly larger than those of Fe­(IV)=O^second^ (−18 vs −17 T, assuming the same value for the zero-field splitting parameter, *D* = 10 cm^–1^) (Table S1). The variable-field Mössbauer analysis also identified a mononuclear ferric species, which develops to a maximum of ∼10% after 10 s and does not exhibit further changes afterward. This ferric species is likely generated via an unproductive pathway where O_2_ addition to the ferrous center results only in the oxidation of the iron center from Fe­(II) to Fe­(III). (All of the time-dependent iron speciation obtained by Mössbauer studies are listed in Table S2.) Overall, two chloro-ferryl species are observed in the AdeV reaction with **1**, which appeared in a sequential manner.

**Figure 2:**
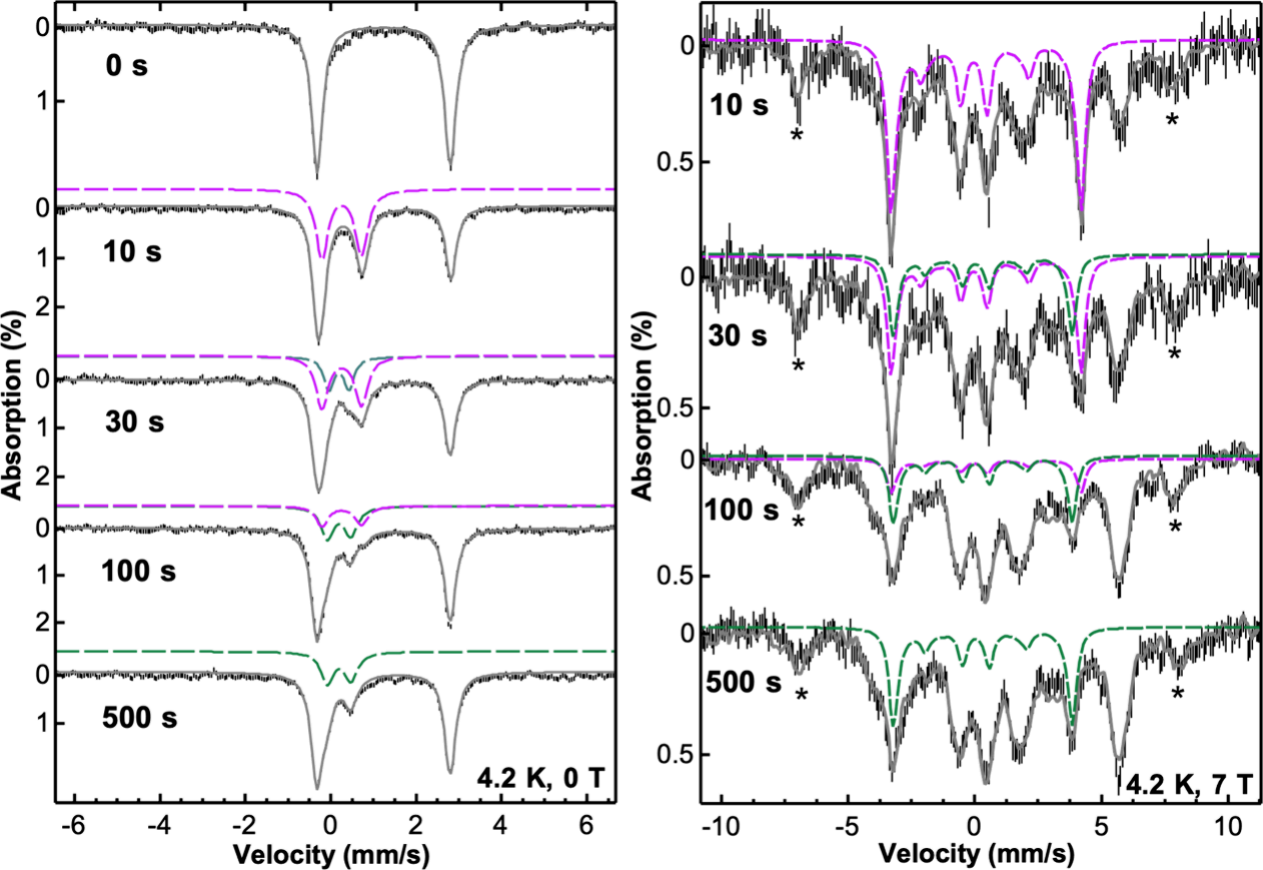
Mössbauer spectra reveal two chloro-ferryl intermediates in the AdeV reaction. Left: zero-field Mössbauer spectra recorded on the samples generated by freeze quenching the reaction between the AdeV•Fe­(II)•2OG•Cl^–^•**1** complex and O_2_ at selected time points as indicated in the figure. Right: corresponding high-field (7 T) Mössbauer spectra. The black vertical bars are the experimental data. The solid gray lines are the total spectral simulations. The purple and green lines represent the spectral simulations of the Fe­(IV)=O^first^ and Fe­(IV)=O^second^ intermediates, respectively. The asterisks indicate the spectral features of mononuclear high-spin ferric species clearly observed in the high-field spectra.

### Only the First Chloro-Ferryl Species Is Capable of C–H Activation

While the appearance of two chloro-ferryl species has been observed in both SyrB2 and CytC3,^21,22^ in these enzymes, both ferryl intermediates formed and decayed simultaneously as opposed to AdeV where two species appear in a sequential manner. Therefore, AdeV provides a unique opportunity to elucidate their roles in the reaction. To provide insights, we prepared chemical quench samples from the Mössbauer samples by quenching them into an acetonitrile and acetic acid mixture. LC–MS analysis on these samples confirmed that the formation of the chlorinated product (**2**) is consistent with the decay of Fe^IV^=O^first^. At 1 s, where minimal Fe^IV^=O^first^ is accumulated, minimal production of **2** is detected. At 10 s, ∼250 μM **2** is produced (corresponding to ∼25% of the total iron concentration), and it continues to accumulate for up to 100 s when Fe^IV^=O^first^ mostly decays (Figure 3). Therefore, the formation of **2** is in accordance with Fe­(IV)-oxo decay kinetics, specifically the decay kinetics of Fe^IV^=O^first^. Additionally, we also detected the minor hydroxylated product, and its formation occurred simultaneously with **2**. From 10 to 100 s, ∼4-fold of this product is detected. The ratio between these two products was maintained at ∼20:1 with **2** as the dominant product. CQ–MS results do not support the hypothesis that chlorination and hydroxylation result from different chloro-ferryl species. Instead, these results are aligned with the reaction pathway where the two products are both generated directly from Fe­(IV)=O^first^. Therefore, Fe­(IV)=O^first^ is the only kinetically competent intermediate for triggering C–H bond cleavage, further leading to chlorination and hydroxylation.

**Figure 3:**
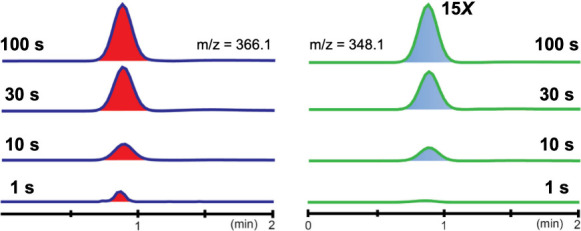
Liquid chromatography mass spectrometry (LC–MS) analysis of the AdeV-catalyzed reaction. The chromatograms of product **2** (*m*/*z* = 366.1, left) and the hydroxylated product (*m*/*z* = 348.1, right) quenched at different reaction times (1, 10, 30, and 100 s) show that both products are produced in a time-dependent manner and follow a similar kinetic trend.

### Kinetics Analysis of AdeV-Catalyzed Chlorination

The FQ-Mössbauer studies and the CQ–MS analysis described above reveal interesting reaction kinetics for AdeV where two chloro-ferryl intermediates are observed, but only the early intermediate seems to be capable of performing chemistry. To further derive a kinetic model, we performed additional SF-Abs experiments by varying the substrate concentrations in the AdeV reaction. The substrate concentration ([substrate])-dependent measurements revealed that the observed formation rate (*k*_obs_^form^(310 nm)) of the 310 nm optical feature, representing the chloro-ferryl intermediate, showed saturation kinetics at high substrate concentration, which is best illustrated by a [substrate]-vs-*k*_obs_^form^(310 nm) plot (Figures 4 and S5). This kinetic behavior indicates that a rapid equilibrium step takes place prior to the formation of the 310 nm feature. This rapid equilibrium step is most likely the substrate binding and dissociation step at the ferrous AdeV complex (AdeV•Fe^2+^•2OG•Cl^–^+**1** ↔ AdeV•Fe^2+^•2OG•Cl^–^•**1**) based on the iron species observed in the FQ-Mössbauer analysis (only the ferrous species was observed prior to the accumulation of the chloro-ferryl species). The observation that *k*_obs_^form^(310 nm) does not fully saturate even in the presence of the 60 mM substrate (0.23 mM ferrous AdeV complex used in the experiment) indicates that AdeV forms only a weak reactant complex with its primary substrate (*K*_d_ is large; see below). Considering the kinetic data from the SF-Abs experiments, the spectroscopic characterizations of the chloro-ferryl species, and product analysis on the reaction mixture quenched at different times, the following kinetic model could be derived to describe the involvement of the two chloro-ferryl intermediates (Fe^IV^=O^first^ and Fe^IV^=O^second^) and the overall AdeV catalysis, which features a reversible interconversion between Fe^IV^=O^first^ and Fe^IV^=O^second^

1 $$E+S\underset{K_{d}}{↔}ES+O_{2}\overset{k_{2}}{\to }{Fe\left(IV\right)}^{1st}\overset{k_{3}}{\to }EP\overset{k_{4}}{\to }E+P$$

2 $${Fe\left(IV\right)}^{1st}\overset{k_{5}}{\underset{k_{-5}}{↔}}{Fe\left(IV\right)}^{2nd}$$

3 $$ES+O_{2}\overset{k_{x}}{\to }X$$

where E represents the AdeV•Fe^2+^•2OG•Cl^–^ complex, S represents primary substrate **1**, ES represents the AdeV•Fe^2+^•2OG•Cl^–^•**1** complex, EP represents the enzyme product complex, and P represents product **2** (here the formation of the hydroxylated product was not included in the kinetic model due to its very low concentration, ≤5%, relative to **2**). The observed saturation kinetics of the formation rate (*k*_obs_^form^) of Fe^IV^=O^first^ (Figure 4, left) suggests that the first step in eq 1, the substrate binding and dissociation, is in fast equilibrium with *k*_1_ (the association rate constant) and *k*_–1_ (the dissociate rate constant), much faster than the O_2_ addition rate constant (*k*_2_), in particular *k*_–1_ ≫ *k*_2_. Under this fast equilibrium, *k*_obs_^form^ can be expressed as $k_{obs}^{form}=\frac{K_{1}k_{2}\left[sub\right]\left[O_{2}\right]}{K_{1}\left[sub\right]+1}+k^{\prime }$ , where *K*_1_ is the substrate association constant (*K*_1_ = 1/*K*_d_), *k*_2_ is the O_2_ addition rate constant, and *k*′ is the sum of the rate constants of the subsequent kinetically resolved steps (Figure 4, left). By using this expression, the substrate dissociation constant is extracted to be *K*_d_ = 28 mM, *k*_2_ is ∼1.80 mM^–1^ cm^–1^ (assuming 0.9 mM O_2_ upon mixing of the O_2_ saturated buffer with the AdeV ferrous reactant complex), and *k*′ is ∼0.06 s^–1^. Using this kinetic information and the kinetic model shown in eqs 1–3, kinetics simulations on the time-dependent iron speciation derived from the FQ-Mössbauer data and the product formation derived from the CQ–MS data are further carried out (Figure 4, right). The simulation results indicate that Fe^IV^=O^first^ slowly interconverts to Fe^IV^=O^second^ with a forward rate constant (*k*_5_) of ∼0.015 s^–1^ and a backward rate constant (*k*_–5_) of ∼0.001 s^–1^. The large difference in *k*_5_ and *k*_–5_ leads to a favorable accumulation of Fe^IV^=O^second^ at a longer reaction time (>100 s) as illustrated by the FQ-Mössbauer data. However, only Fe^IV^=O^first^ enables the C–H activation and the chloride rebound step (with *k*_3_ ≈ 0.05 s^–1^) to form the product. Here we assume that the product release is very fast (*k*_4_ is large) so that no EP complex is accumulated. This is at least consistent with the Mössbauer results, where only a single ferrous species (most likely a mixture of E and ES given the large *K*_d_ on substrate binding) is observed throughout the reaction. The observation of mononuclear ferric species in the Mössbauer analysis suggests that a small portion of the enzyme–substrate complex reacts with O_2_ and undergoes a nonproductive pathway with *k*_*x*_ ≈ 0.25 mM^–1^ s^–1^. (All of the equilibrium constant and rate constants used for the kinetics simulations are listed in Table S3.)

**Figure 4:**
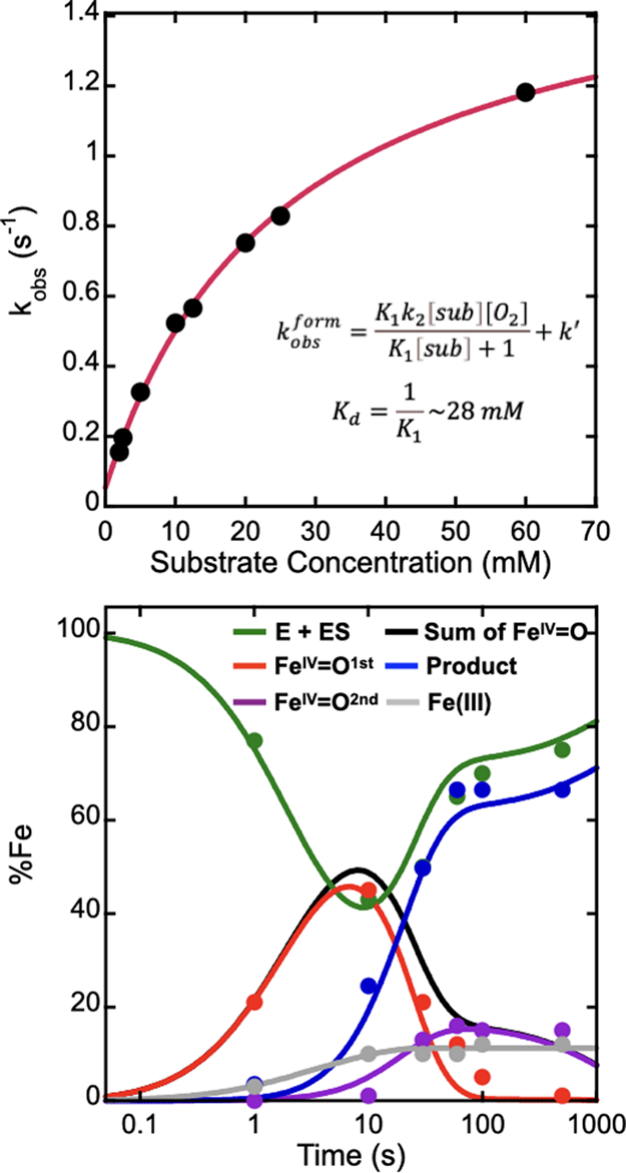
Kinetics simulations of the AdeV reaction. Top: substrate-dependent formation rates (dots) of the chloro-ferryl intermediate observed by monitoring the 310 nm absorbance change in the SF-Abs experiment and the corresponding fitting (red solid line) using the expression shown in the figure. Bottom: time-dependent iron speciation and product formation determined by FQ-Mössbauer analysis and CQ–MS analysis (dots) and the kinetics simulations (solid lines) using the kinetic model shown in eqs 1–3.

To provide further support for the chloro-ferryl interconversion, we measured a Mössbauer sample that was generated by incubating the anaerobic enzyme–substrate complex (1 mM) with a limiting amount of O_2_ (∼0.9 mM) for 1800 s. As predicted by the above kinetic model, at a longer time (i.e., >1000 s), both chloro-ferryl species should be completely decayed and the iron should return to Fe­(II). Indeed, this 1800s-O_2_-incubated sample exhibits an ∼90% ferrous signal identical to that of the ES complex and an ∼10% mononuclear ferric species signal, devoid of any chloro-ferryl signal (Figure S4). The ferric species is most likely generated by the reaction uncoupling, which was already present at the earlier reaction times (Figure 2). Together, the current observations support the interconversion of two chloro-ferryl species. In addition, this is the first experimental confirmation that the chloride and oxygen rebound pathways are initiated through the same chloro-ferryl species (i.e., Fe^IV^=O^first^), which thus suggests that the two pathways (e.g., chlorination and hydroxylation) branch at the same Fe^III^–OH-carbon-radical complex and the chloride rebound pathway has a lower reaction barrier than that of the oxygen rebound pathway. It is also intriguing to reveal that the long-lived Fe^IV^=O^second^ neither participates in C–H activation nor leads to self-decay. Instead, it converts back to Fe^IV^=O^first^ (more discussion is included in the computational section).

### AdeV-Catalyzed Chlorination Retains the Stereochemistry

Quite a few Fe/2OG enzyme-mediated chlorination reactions including SyrB2, CytC3, WelO5, and BesD have been reported.^11–17^ However, the stereochemistry of chlorination remains unknown. We reasoned that this information is not only important to fully characterize the outcome of the AdeV-catalyzed chlorination but also intimately connected to the relative spatial orientation between the Cl–Fe^IV^=O moiety and the target C–H bond and between the Cl–Fe–^III^–OH moiety and the carbon-centered radical, thus revealing the key information on the C–H bond cleavage and the chloride rebound steps.

Herein, using a substrate isotopologue and the characterization of the AdeV reaction products, the stereochemistry of AdeV-catalyzed chlorination is determined. To verify that AdeV produces only **2** but not the epimer (epi-**2**), we chemically synthesized **2** and epi-**2** (Figure S24, see Supporting Information for the synthetic details and the product characterizations for all the compounds used in this study, Figures S6–S34). As shown in Figure 5a, epi-**2** and **2** have different retention times on LC (7.7 and 7.9 min for **2** and epi-**2**, respectively). A side-by-side comparison of the AdeV reaction with the standards reveals that the AdeV reaction product has retention time matches with that of **2** but not epi-**2**. This observation firmly establishes that AdeV catalyzes stereospecific chlorination.

**Figure 5:**
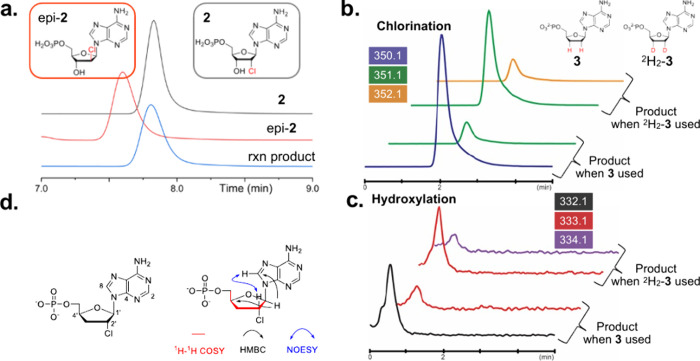
(a–c) LC–MS and (d) NMR analysis establish the stereochemical outcome of the AdeV-catalyzed halogenation. (a) LC–MS analysis shows that the enzymatic product has the same retention time as the synthetic **2** but is distinct from epi-**2**. (b, c) LC–MS analysis of the AdeV reactions with **3** or ^2^H_2_-**3**. Compared to the reaction using **3**, both chlorination and hydroxylation show an M + 1 shift in the presence of ^2^H_2_-**3**, suggesting that one deuterium is retained in both chlorination (b) and hydroxylation (c) products. (d) Two-dimensional NMR analysis used to determine the chemical structure of the chlorination product (**4**).

To establish the stereochemistry of chlorination, a correlation between C–H cleavage and C–Cl formation needs to be established. Because AdeV also catalyzes the chlorination of 2′,3′-dideoxyadenosine monophosphate (dAMP, **3**), we use it to elucidate the stereochemical relationship of C–H cleavage and C–Cl formation. First, we synthesized both **3** and [2′,3′-*R*,*S*-^2^H_2_]-**3**. In our synthesis, using the steric hindrance at substituents C1′ and C5′, stereospecific hydrogenation is achieved. When deuteron gas is applied, [2′,3′-*R*,*S*-^2^H_2_]-**3** can be synthesized. The stereochemistry of deuteron is confirmed using detailed NMR analysis (Figures S31 and S34). To test whether pro-*R* or pro-*S* H cleavage takes place, **3** and [2′,3′-*R*,*S*-^2^H_2_]-**3** are reacted with AdeV. As shown in Figure 5b,c, when **3** is used, a chlorination product with *m*/*z* = 350.1 is observed. Additionally, a peak with an *m*/*z* value that matches hydroxylation (*m*/*z* = 332.1) is also detected. In the presence of [2′,3′-*R*,*S*-^2^H_2_]-**3**, *m*/*z* shifts of +1 in chlorination and hydroxylation are observed (*m*/*z* 350.1, 351.1 and 332.1, 333.1, respectively), confirming that both chlorination and hydroxylation products have only one deuterium retention. Next, we carried out a large-scale enzymatic reaction and isolated the chlorinated product (**4**). Based on the two-dimensional NMR spectra including ^1^H–^1^H COSY, ^1^H–^1^H NOESY, and ^1^H–^13^C HMBC, the structure of **4** is determined (Figure 5d). Specifically, the observation of ^1^H–^1^H NOESY correlation of C2′–H and the C8–H of adenine reveals that the stereochemistry at C2′ is at the *R* configuration (Figure 5d). Taken together, the LC–MS result and the enzymatic product structure determination by NMR establish the stereochemical correlation of C–H bond cleavage and C–Cl formation. Following the C–H cleavage that results in substrate radical formation, the chloride rebound retains the stereochemistry. While we cannot perform the full NMR characterization of the hydroxylation product due to the inadequate quantity formed during enzymatic reaction, it is most likely that hydroxylation also occurs at the C2′ carbon. Therefore, both C–Cl and C–OH formation pathways proceed through C2′-pro*R* H cleavage; however, the chloride rebound outcompetes the oxygen rebound to enable chlorination as the major product.

### Computational Investigation of AdeV-Catalyzed Chlorination

To derive further insights into the reaction mechanism of chlorination by AdeV, molecular dynamics (MD) simulations and quantum mechanics/molecular mechanics (QM/MM) calculations were carried out to reveal the protein dynamics in the Fe­(III)-superoxo and the chloro-ferryl state and the reaction profiles of the O_2_ activation, C–H activation, and Cl• and OH• rebound steps. The computational results were further correlated with the experimental observations to elucidate the detailed mechanism of AdeV catalysis. (See the SI for the description of calculation details. Additional calculation results are shown in Figures S35–S54 and in Tables S6–S23.) The reaction paths described in the following sections are calculated at zero-point-corrected energies (QM­(B3)/MM level as described in the SI), in line with the established practice in enzyme catalysis studies.^47–49^

#### Conformational Dynamics of the Fe^III^-Superoxo Complex and Reaction Mechanism of O_2_ Activation in AdeV

Several computational studies have reported the O_2_ activation mechanism of nonheme Fe­(II)/2OG-dependent hydroxylases and halogenases.^35,36,50–59^ In this study, to explore the mechanism of the formation of the two chloro-ferryl intermediates observed experimentally, we also examined the chloro-Fe^III^-superoxo species and the associated O_2_ activation mechanism. We structurally modeled this species in two forms, the offline and inline forms, as proposed in several computational studies. The two chloro-Fe^III^-superoxo species are two conformational isomers, with one having the superoxo ligand *trans* to H250 (inline) and the other with the superoxo ligand trans to H192 (offline). Initially, we performed MD simulations of both offline and inline superoxo systems to obtain well-equilibrated trajectories of both systems (Figures S36 and S37). The atomistic analysis revealed stable hydrogen bonds formed by R175 with the C1 carboxylate of 2OG in the offline system (Figure S38), while Q201 forms a hydrogen bond with the C1 carboxylate of 2OG in the inline system (Figure S39). Furthermore, in both the inline and offline systems, the C5 carboxylate of 2OG was stabilized by hydrogen bonds with R263 and S265 (Figures S38 and S39). Further, we implemented dynamic cross-correlation analysis (DCCA) to understand long-range-correlated motions in both the offline and inline chloro-Fe^III^-superoxo complexes. In both complexes, the Fe center and the first coordination sphere ligands show highly correlated motions with residues 186–211, which contain the iron-binding H192 and the loops connecting the β-sheets of the DSBH fold. In the inline system, there was a strong anticorrelation motion between the Fe center and residues 256–264 (Figure S40), containing residues stabilizing the C5 carboxylate of 2OG (Figure S40). However, the magnitude of correlation/anticorrelation was higher in the case of the inline system than the offline system. Additionally, residues 256–264 showed anticorrelated motion with residues 91–105 in both systems, which surround the substrate, 2′-dAMP (**1**). Principal component analysis (PCA) (Figure S40) also revealed that these three regions were more flexible in the inline system than in the offline system, in agreement with the stronger correlated/anticorrelated motions observed for the inline system in the DCCA analysis. Understanding the dynamic features of these two Fe^III^-superoxo complexes provides us insight into the interplay among the active site, the second coordination sphere, and long-range residues of AdeV in the O_2_ activation reaction and the subsequent chloro-ferryl formation.

To explore the O_2_ activation reaction, we obtained a snapshot from both offline and inline chloro-Fe^III^-superoxo systems and performed QM/MM optimization of the geometries to obtain the offline superoxo reactant complex (Off-SO-RC) and the inline superoxo reactant complex (In-SO-RC). The QM region definitions used for these calculations are described in the QM/MM calculations section in the SI. From both Off-SO-RC and In-SO-RC, the initial O_2_ activation reaction involves Od-C2 bond formation between O_2_ and 2OG and C1–C2 bond breakage within 2OG, resulting in decarboxylation to form the chloro-Fe^II^-peroxo-succinate complexes (Off-SO-IM1 and In-SO-IM1). The initial decarboxylation reaction required reaction barriers of 10.6 and 7.5 kcal/mol in Off-SO-RC and In-SO-RC, respectively (Figures S41 and S42). In the case of In-SO-RC, along the reaction path, the angle formed by the nitrogen (N) of H250 with Fe-Op (∠N–Fe–Op) decreased from 162.7° in the reaction complex to 129.8° in In-SO-IM1 (Figure S41). However, in Off-SO-RC, the same angle increased from 87.0 to 113.9° in Off-SO-IM1 (Figure S42). The subsequent Op–Od bond cleavage required a small barrier of 2.2 kcal/mol in the offline system and became barrierless in the inline system at the QM­(B3)/MM level, which led to the formation of an Fe^III^–O^–^ intermediate at In-SO-IM2 and Off-SO-IM2 intermediate states, respectively, along with the succinate. The Fe–Op and Op–Od distances were 1.75 and 2.11 Å in these two states, indicating a partial bond between Op and Od. In addition, at In-SO-IM2, ∠N–Fe–Op decreased to 125.1°, reaching the offline orientation with Op almost trans to H192 (Figure S41). However, in Off-SO-IM2, ∠N–Fe–Op reduced to 95.9°, retaining the offline orientation (Figure S42). Further complete breakage of the Op-Od bond and rearrangements of ∠N–Fe–Op led to offline chloro-ferryl species with the oxo group trans to His192 with Fe–Op distances of 1.62 and 1.61 Å in both In-SO-PD and Off-SO-PD, respectively. Hence, the calculations show that irrespective of the initial configuration of the chloro-Fe^III^-superoxo complex (In-SO-RC and Off-SO-RC), the O_2_ activation reaction leads to offline chloro-ferryl formation. These computational results are consistent with the experimental observation, which showed that O_2_ activation in AdeV initially led to only the formation of a single chloro-ferryl intermediate (Fe^IV^=O^first^). Fe^IV^=O^first^ then reversibly converted to the second chloro-ferryl intermediate (Fe^IV^=O^second^).

#### Conformational Dynamics of the Two Chloro-Ferryl Intermediates in AdeV

Based on the above-described computational results and the experimental results, we hypothesized that the two chloro-ferryl species are possibly two interconverting structural isomers, with one having the oxo ligand facing the substrate (inline: *trans* to H250) and the other having the oxo ligand facing away from the substrate (offline: *trans* to H192). Based on these structural models, we performed MD simulations. We obtained well-equilibrated trajectories for both systems (Figures S43 and S44). In the inline complex, the simulations predicted that the nonbonded oxygen of the C1 carboxylate of succinate forms a hydrogen bond with the guanidino group of R180, while the C4 carboxylate of succinate forms a hydrogen bond with R263. Conversely, in offline dynamics, the C1 carboxylate forms a hydrogen bond with S265 while the C4 carboxylate maintains the hydrogen bond with R263. In both systems, iron-coordinated histidines (H250 and H192) form hydrogen bonds with each other. The phosphate group of substrate 2′-dAMP (**1**) forms hydrogen bonds with R175 and R235 in both systems. Interestingly, in the offline system, the adenine group of 2′-dAMP forms hydrogen bonds with D103 and I195 while the ribose hydroxy group forms hydrogen bonds with Q196. However, such interactions are not observed in the inline system. Due to this difference in the hydrogen bonding interactions, the substrate binding is more stable in the offline system than in the inline system (Figure S45).

To understand the correlated motions present in both offline and inline systems, we implemented DCCA on 1 μs MD simulations. Based on DCCA, the Cl–Fe^IV^=O complex and the coordinated groups in the inline system depict highly correlated motions with residues 186–211, which form the DSBH fold, and anticorrelated motions with residues 241–256, which contain iron-binding residues (Figure 6). However, in the offline system, the anticorrelated motions presented between these regions and residues 91–105 in the inline system are lost, while the correlated motions between the Fe center and residues 186–211 are present (Figure 6). A similar trend is reflected in the PCA analysis, which depicts the regions containing residues 91–105, residues 186–211, and residues 241–256, showing more flexibility in the inline system than in the offline system, similar to the dynamics of the Fe^III^-superoxo systems (Figures 6 and S40). The variations in the correlated motions and flexibilities of these residues between the inline and offline systems might be instrumental for the stereospecific access to the pro-R hydrogen of the substrate in the offline system. Based on the conformational analysis, our results suggest considerable differences in the overall flexibility and the correlated motions presented in the inline and offline chloro-ferryl systems, and overall, the offline system exhibits less dynamics and flexibility than the inline system. This is also true for the binding dynamics of the substrate, where in the offline system the substrate binds more tightly with less mobility in the active site than in the case of the inline system (Figure S45).

**Figure 6:**
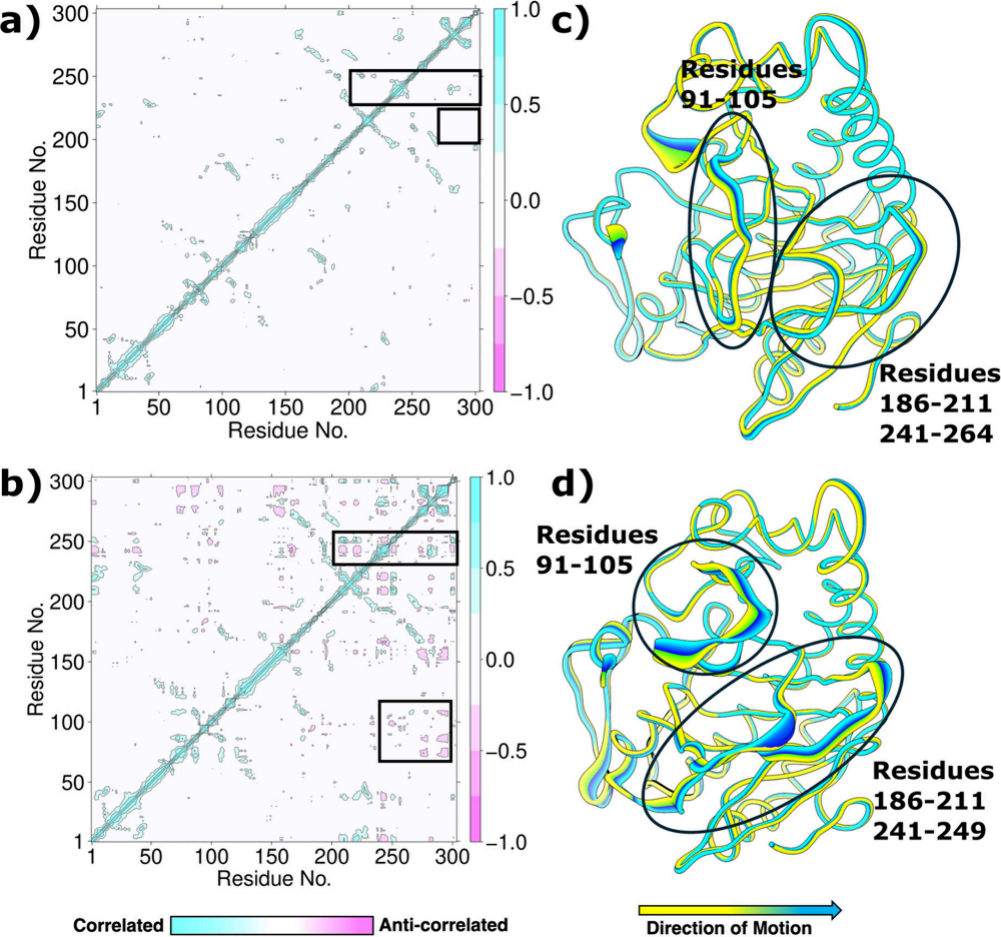
Overall protein dynamics of the offline and inline ferryl systems. The dynamic cross-correlation matrix shows the regions of correlated and anticorrelated motions in (a) offline and (b) inline ferryl systems, and principal component analysis shows the flexible regions of the (c) offline and (d) inline ferryl systems. The boxed regions show the correlated/anticorrelated motion of residues 91–105, 186–211, and 241–264 with the active site residues (a and b), and circled regions show the flexibility of the corresponding residues (c and d).

#### Mechanism of Chlorination in AdeV

We next performed QM/MM reaction path calculations on the chloro-ferryl complexes obtained from the inline and offline systems to explore the reaction mechanism of chlorination. We chose snapshots from the equilibrated portion of the 1 μs MD trajectories of offline and inline ferryl and optimized to obtain the offline ferryl-substrate complex (Off1-RC) and the inline ferryl-substrate complex (In1-RC), respectively. (The QM region definition is described in the QM/MM Calculations section in the SI.) The Fe=O distances were 1.62 and 1.61 Å in Off1-RC and In1-RC, respectively. In the Off1-RC state, the distance between the ferryl oxygen and the pro-*R* hydrogen of C2′ (O–H2′′) is 4.70 Å, whereas in the In1-RC state, the pro-*S* hydrogen of C2′ (O–H2′) is closer to ferryl oxygen with 3.31 Å. Thus, in Off1-RC, sterically, pro-*R* hydrogen is favorable for HAT, which is consistent with the experimental results, and in In1-RC, pro-*S* hydrogen may be favorable for HAT, which is not observed experimentally. The altered access to the hydrogens of the C2′ of the substrate in the offline and inline configurations can result from the difference in conformational flexibility of the substrate in both systems (Figure S45). The reaction initiates from Off1-RC and In1-RC through a hydrogen atom transfer (HAT) from the C2′ carbon to the ferryl oxygen. Although the distance between the oxo ligand and the targeted H from C2′ is longer in Off1-RC (O–H2′′, pro-*R* H, 4.70 Å) than in In1-RC (O–H2′, pro-*S* H, 3.31 Å), the HAT reaction from Off1-RC gives a lower barrier of 23.1 kcal/mol via transition state Off1-TS1_pro‑R_ than that from In1-RC, which gives a higher barrier of 32.1 kcal/mol via transition state In1-TS1_pro‑S_ (Figures 7 and S46).

**Figure 7:**
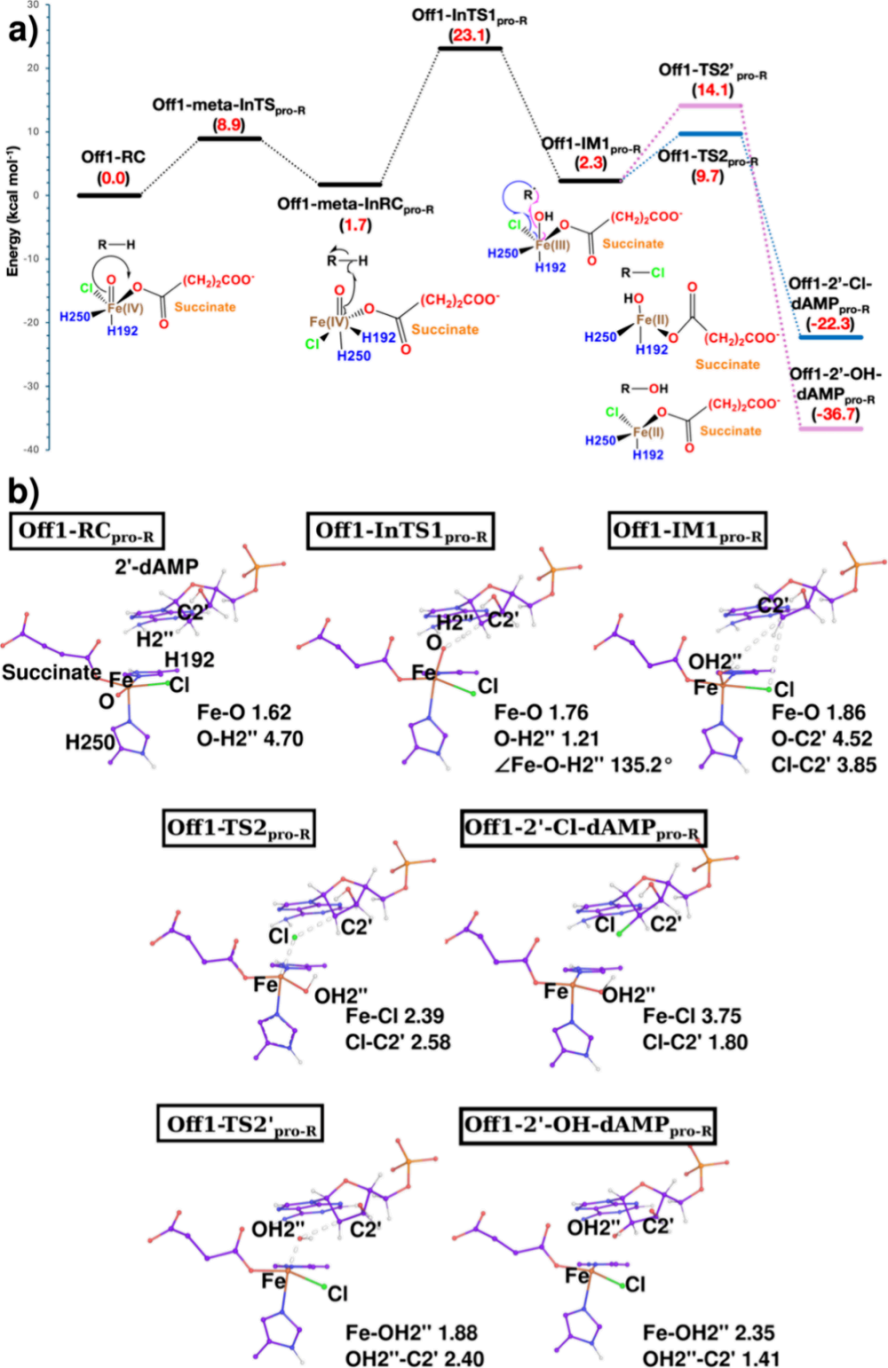
Reaction profile and molecular structures of the iron center derived from QM/MM calculations. (a) Reaction profile of halogenation and hydroxylation mechanism in the offline ferryl system. Relative energies are given in kcal/mol, calculated at the zero-point corrected energies (QM­(B3)/MM level). (b) Representations of QM/MM optimized structures obtained during the halogenation and hydroxylation reaction in offline ferryl. Nonpolar hydrogens are hidden except for the substrate for clarity. Distances are given in angstroms. Structures of Off1-meta-InTS and Off1-meta-InRC are given in the SI (Figure S39).

A closer examination suggested that for the offline system, the HAT is actually initiated by a transformation of Off1-RC from offline to a metastable inline configuration (Off1-meta-InRC_pro‑R_) through a transition state (Off1-meta-InTS) with a barrier of 8.1 kcal/mol (Figures 7 and S47). Such an Fe^IV^=O isomerization from the offline to the inline configuration during HAT was also reported in computational studies on halogenase SyrB2 ^33^ and hydroxylases such as AsqJ,^60^ PHF8,^54^ and AlkB.^55^ In hydroxylases, isomerization occurs from the offline to the inline state, potentially through an Fe-bound water molecule in the ferryl state; additionally, the system remains in the inline state after HAT. In the current study, from Off1-meta-InRC_pro‑R_, the true HAT begins, proceeding through Off1-InTS1_pro‑R_ with a barrier of 21.4 kcal/mol relative to Off1-meta-InRC_pro‑R_, which compares reasonably well with the experimental rate constant of 0.05 s^–1^ (19.4 kcal/mol). At Off1-InTS1_pro‑R_, the O–H2′′ distance is 1.21 Å and ∠Fe–O–H2′′ is 135.2°, while at In1-TS1_pro‑S_ (the HAT transition state for the inline chloro-ferryl system), the O–H2′ distance is 1.26 Å and ∠Fe–O–H2′ is 160.5° (Figures 7 and S46). The distances obtained in the RCs and TSs are consistent with previously reported computational studies on nonheme Fe­(II)/2OG halogenases.^26,33,38,61^ Importantly, the intermediate Cl–Fe^III^–OH complex (Off1-IM1), formed after the completion of the HAT in the reaction path of the offline system, recovers the original offline orientation with a *trans* configuration to H192 (Off1-IM1, Figure 7). The above-described offline-to-inline isomerization of the Fe^IV^=O moiety for HAT is unique only to the offline system and was recently reported computationally for an ethylene-forming enzyme.^62^ In contrast, for the inline system, the inline configuration is maintained throughout the HAT step from In1-RC to the product Cl–Fe^III^–OH complex (In1-IM1) without an Fe^IV^=O isomerization step (Figure S46). Regarding the HAT reaction channel (σ-pathway vs π-pathway), consistent with ∠Fe–O–H calculated at the TS states, the spin density analysis shows that the HAT follows the σ pathway in both the inline and offline systems, which shows the presence of a β electron at the C2′ carbon of the 2′-dAMP substrates at Off1-IM1 and In1-IM1 (Figure S48 and Tables S14–S17). Multiple reports on halogenases demonstrated that the HAT could proceed from both the offline and the inline ferryl configurations and follow either the σ or the π pathway. In particular, the studies on SyrB2 halogenases demonstrated that the offline configuration with either the σ or π pathway^24–26,32^ and the inline configuration via the σ pathway can be operative.^32,33^ A study of HctB halogenase also suggested an inline/σ pathway for C–H activation,^63^ while the studies on BesD and WelO5 indicated an offline/π mechanism.^30^ Our computational results on the HAT step are thus generally consistent with previously reported computational studies on Fe/2OG halogenases and further feature an offline-to-inline isomerization step en route to the HAT transition state for the offline ferryl species. Furthermore, we also explored HAT from the pro-*S* hydrogen of C2′ in Off1-RC and pro-*R* HAT from In1-RC. The pro-*S* HAT from Off1-RC again led to Off1-meta-InRC_pro‑S_ with an offline-to-inline Fe^IV^=O isomerization, which was exothermic by −8.3 kcal/mol. From Off1-meta-InRC_pro‑S_, the actual HAT required a higher barrier of 31.9 kcal/mol through Off1-TS1_pro‑S_ (Figure S49), reiterating that pro-*R* HAT is preferred over pro-*S* HAT in the case of Off1-RC, consistent with the experimental observation. The pro-*R* HAT from In1-RC required a conformational change of the substrate where the phosphate group undergoes rearrangement, requiring a barrier of 28.8 kcal/mol to form an exothermic intermediate (In1-RC_pro‑R_) with a −14.8 kcal/mol energy. From In1-RC_pro‑R_, the HAT reaction required an unfeasible activation barrier of 41.8 kcal/mol (Figure S50). Hence, the calculations suggest that the inline chloro-ferryl system (In1-RC) favors the pro-*S* HAT with a barrier of 32.1 kcal/mol over the pro-*R* HAT with a barrier of 41.8 kcal/mol. However, both reaction barriers are much higher than the pro-R HAT calculated for the offline chloro-ferryl system (23.1 kcal/mol), thus suggesting that the pro-*R* HAT by the offline chloro-ferryl system is the most favorable HAT pathway for AdeV, which would outcompete all other HAT pathways examined here.

We also calculated the kinetic isotope effect (KIE) for pro-*R* and pro-*S* HAT for the Off1-RC and In1-RC complexes by replacing the hydrogen with the heavier isotope deuterium, as tunneling is sensitive to the mass of the tunneling particle. At 303 K, in Off1-RC, the KIE value for pro-*R* HAT was calculated to be 31.9, and the same for pro-*S* HAT was 44.9. Similarly, in In1-RC, the KIE values for pro-*R* and pro-*S* HAT were calculated to be 29.3 and 33.6, respectively. Although the calculated KIEs indicate considerable tunneling contributions in both Off1-RC and In1-RC, with a larger value for the pro-*S* HAT (especially for the one starting from Off1-RC), the observed pro-*R* stereoselectivity indicates the conformational positioning of the substrate revealed by MD simulations and the QM/MM calculations appear to be the predominant factor for the stereoselectivity, which might outweigh the tunneling contribution during HAT.

Next, we examined the rebound step. At Off1-IM1, the Cl^–^ ion is closer to the C2′ radical with a 3.85 Å distance compared to the OH group, which is 4.52 Å away from the C2′ carbon radical (Figure 7). Due to the closer access of the Cl^–^ ion to the C2′ carbon radical in Off1-IM1, chlorination is preferred with a barrier of 7.4 kcal/mol compared to the 11.7 kcal/mol barrier required for hydroxylation in the offline system, and the formed C–Cl bond in the product state exhibits an *R* configuration, consistent with the experimental observations. Conversely, at In1-IM1, the Cl^–^ ion (6.92 Å) is much further away from the C2′ radical than the OH group (3.95 Å) (Figure S46). Thus, chlorination is not preferred in the inline system, with the 57.3 kcal/mol barrier required for chlorination and hydroxylation requiring only 13.2 kcal/mol (Figure S46). But this hydroxylation barrier by In1-IM1 is still higher than both the chlorination barrier (7.4 kcal/mol) and the hydroxylation barrier (11.7 kcal/mol) by Off1-IM1. Hence, based on the overall QM/MM reaction path calculations, we propose that the offline chloro-ferryl system favors the chlorination of 2′-dAMP with an *R* configuration.

To further explore the HAT step, we chose additional snapshots of the inline and offline systems and optimized them to obtain In2-RC and Off2-RC (Figures S51 and S52). In the Off2-RC snapshot, a hydrogen bond exists between the ferryl oxygen and the Q201 residue, contrary to that of the Off1-RC (Figure S51). In the In2-RC snapshot, the 2′-dAMP has a phosphate group that is differently oriented with a hydrogen bond to R175, which is not present in the In1-RC snapshot (Figure S52). Similar to Off1-RC, in Off2-RC, ferryl oxygen is sterically favorable for HAT for pro-*R* hydrogen with an O–H2′′ distance of 5.5 Å. However, in In2-RC, the ferryl oxygen sterically also favors the access of pro-*R* hydrogen with an O–H2′′ distance of 3.54 Å, contrary to In1-RC, which favors pro-*S* hydrogen. Based on the stereochemistry of the product obtained during the experiments mentioned above, the pro-*R* hydrogen should be the site for HAT, which is observed in both offline RCs. During HAT from the Off2-RC, the reaction proceeds with a 27.6 kcal/mol barrier comparable to the barrier in the Off1-RC snapshot (Figure S53). However, HAT from In2-RC requires a much higher barrier of 32.2 kcal/mol (Figure S53). In1-RC has a hydrogen bonding network between the ferryl oxygen and R175 through water molecules (Figure S52). However, such an interaction is absent in In2-RC, as the phosphate group of the substrate forms a hydrogen bond with R175 (Figure S52). During HAT in Off2-RC, the system transitions to an inline orientation with a barrier of 13.4 kcal/mol (Figure S53), similar to that of Off1-RC, despite the presence of a hydrogen bond between the ferryl oxygen and the side chain of Q201 before hydrogen abstraction (Figure S51). The hydrogen is abstracted following this transition, and the chloro-Fe^III^–OH intermediate typically reverts to the offline orientation (Off2-IM1) similar to the Off1-IM1.

Based on our MD and QM/MM studies, the conformational landscape of the offline chloro-ferryl system is predicted to strongly favor both HAT at the pro-*R* C2′–H2′′ site and the subsequent chlorination or hydroxylation with the retention of the stereochemistry, but Cl• rebound is energetically more favorable than OH• rebound based on the reaction barrier (7.4 vs 11.7 kcal/mol). All of these calculation results are fully consistent with experimental observations. However, if the offline chloro-ferryl system transitions into a fully stable inline complex, then the protein relaxes into a less favorable configuration for HAT and subsequent reactions with significantly higher barriers. The computational predictions substantiate the results obtained from the experiments, where two chloro-ferryl intermediates are observed, but only one can perform C–H activation and further lead to both a major chlorinated product and a minor hydroxylated product. Thus, overall, it is likely that the offline chloro-ferryl is favorable to catalysis by AdeV while the inline chloro-ferryl is inactive toward C–H activation, which could be the consequence of the flexibility of substrate binding in the case of the ferryl system, thus leading to unfavorable substrate-ferryl disposition and high reaction barriers as obtained from our computational studies.

### Calculation of Mössbauer Parameters of the Chloro-Ferryl Intermediates

To complement the experimental Mössbauer parameters and identify the nature of the first and second ferryl species, we implemented computational Mössbauer calculations on the two isomers of the chloro-ferryl systems we explored in our QM/MM calculations. (See the Mössbauer Calculations section in the SI for calculation details.) Initially, we performed multiple test calculations with different QM regions, and the results are tabulated in Tables S22 and S23. The best models required the inclusion of four water molecules surrounding the phosphate group of substrate **1** in the QM region. Based on this, we performed QM/MM calculations of the Mössbauer isomer shift (δ) and quadrupole splitting (Δ*E*_Q_) values for the Off1-RC (Table S22) and the In1-RC complexes (Table S23). The calculated values (δ_calc_ = 0.22 mm/s and Δ*E*_Qcalc_ = −1.00 mm/s) for the Off1-RC were in close agreement with the experimentally obtained parameters (δ = 0.23 mm/s and Δ*E*_Q_ = −0.94 mm/s) of the first ferryl species. Similarly, the calculated parameters of δ_calc_ = 0.21 mm/s and Δ*E*_Qcalc_ = −0.71 mm/s for the In1-RC were in agreement with the parameters of the second ferryl species (δ = 0.18 mm/s and Δ*E*_Q_ = −0.55 mm/s) observed experimentally. Hence, these calculated Mössbauer parameters lend further support to the assignment that the first-formed ferryl intermediate is the offline ferryl species and the second one is the inline ferryl species.

## Discussion

The reaction selectivity (chlorination vs hydroxylation) is a major mechanistic question in the studies of Fe/2OG halogenases. Despite intensive studies on the reaction mechanism of Fe/2OG halogenases,^17,23–40^ two fundamental mechanistic questions have not been addressed. First, in SyrB2 and CytC3, two chloro-ferryl intermediates have been observed,^21,22^ but the mechanistic implication of the coexistence of two chloro-ferryl species has not been elucidated. Second, the correlation between the stereochemistry of the targeted C–H bond and the installed C–Cl bond contains important mechanistic insights but has not been determined prior to our current study. Clearly, more experimental results are needed to derive a complete mechanistic understanding of these unique enzymes.

In this study, we showed that two chloro-ferryl species are generated in a sequential manner in AdeV (Figures 2 and 4). Thus, this provides an opportunity to elucidate the potential roles of these two intermediates in the AdeV reaction. Indeed, our experimental data strongly suggest that only the early chloro-ferryl intermediate (Fe^IV^=O^first^) performs the C–H activation and further leads to both chlorination and hydroxylation reactions with the former reaction as the dominant outcome (Figures 3 and 4). Our kinetics analysis suggests that the two chloro-ferryl intermediates interconvert, and the later chloro-ferryl intermediate (Fe^IV^=O^second^) does not participate in the chemical reactions but rather only converts back to Fe^IV^=O^first^ for C–H activation. In BesD, a recent study by Bollinger and co-workers have revealed that only a single chloro-ferryl intermediate was observed.^41^ Thus, the observation of two chloro-ferryl intermediates is not a defining feature for Fe/2OG halogenases. It is very likely that different enzyme active site architecture (e.g., the second coordination sphere composition, the substrate binding dynamics, and/or the substrate-ferryl disposition) may influence the structural dynamics of the ferryl species, thus leading to the observations of different ferryl intermediates with different kinetic behavior. Nevertheless, in both cases (the observation of two ferryl intermediates or of a single ferryl intermediate), the chlorination and hydroxylation outcomes are initiated from the same ferryl intermediate via the HAT step. In the case of AdeV, Fe^IV^=O^first^ is the only kinetically competent intermediate, while Fe^IV^=O^second^ does not participate in the chemistry.

In addition, AdeV also features a rapid equilibrium in primary substrate binding and dissociation, which is evidenced by the slow saturation kinetics observed in the substrate-dependent measurements of the formation rate of the chloro-ferryl intermediate (Figure 4). The estimated dissociation constant (*K*_d_) for substrate binding is ∼28 mM (in the presence of an excess amount of 2OG and Cl^–^), suggesting that AdeV forms only a weak ferrous reactant complex and the substrate binding most likely does not induce significant protein conformational change. However, such a weak association of substrate is still sufficient to enable binding and activation of O_2_ to form the chloro-ferryl intermediate and initiate the catalysis.

To better define the reaction mechanism of AdeV, we further determined the stereochemistry of the activated C–H bond and the installed C–Cl bond in the AdeV reaction. Specifically, by chemical synthesis of the substrate, substrate analogue, and isotopologues, coupled with LC–MS and NMR analyses, we have determined that the C–H activation occurs at pro-*R* H of the C2′ position of **3** with the subsequent retaining of the same stereochemistry of the installed C–Cl in the chlorinated product and most likely also of the installed C–OH in the hydroxylated product. This stereochemistry information provides a crucial experimental reference point for the subsequent computational studies described in this study.

All of these experimental observations provide important restraints for developing computational models to further elucidate the AdeV reaction mechanism. The MD and QM/MM studies show that irrespective of the initial structural configuration of the chloro-ferric-superoxo complex (offline vs inline), the O_2_ activation reaction leads to the same offline chloro-ferryl formation. The computational studies further suggest that the two experimentally observed chloro-ferryl intermediates may represent the offline and inline configurations, respectively, with only the offline chloro-ferryl exhibiting energetically favorable C–H activation and leading to both chlorination and hydroxylation. Specifically, the offline chloro-ferryl state shows less protein dynamics and flexibility and a more stable substrate–protein complex than those of the inline chloro-ferryl state. These dynamic behaviors also translate to an overall favorable C–H activation process for the offline state due to a lower transition state barrier and a less endothermic Fe^III^–OH–carbon–radical intermediate. In addition, the C–H activation step by the offline ferryl state features an offline-to-inline isomerization of the Fe^IV^=O moiety, which forms a metastable inline Fe^IV^=O en route to the C–H activation transition state. Thus, hydrogen atom abstraction is carried out exclusively via the σ-pathway with a clear α-spin transfer from the activated C–H bond to the Cl–Fe^IV^=O center. Subsequently, the Fe^III^–OH moiety reverts back to the offline configuration, setting up a shorter C2′···Cl distance (3.85 Å) than the C2′···OH distance (4.52 Å) to facilitate the chloride rebound, and the overall stereochemistry of the activated C–H bond and the installed C–Cl bond is consistent with the experimental observations (a pro-*R* HAT followed by an *R*-configuration C–Cl bond). Also, the computed Mössbauer parameters of the offline chloro-ferryl correlate well with the experimentally observed parameters of the first chloro-ferryl species. Thus, it is very likely that the computationally derived offline ferryl state represents the first chloro-ferryl intermediate (Fe^IV^=O^first^) observed experimentally. Regarding the inline chloro-ferryl state, the computational studies indicate that this state exhibits more protein dynamics and a less stable substrate–protein complex and thus cannot efficiently perform C–H activation, which is further supported by the reaction barrier calculations, showing much higher barriers for both the HAT and the rebound step than those from the offline ferryl state (≥10 kcal/mol in the case of HAT). These computational results are in accordance with the long-lived and unreactive second chloro-ferryl species (Fe^IV^=O^second^) observed experimentally. Similarly, the computed Mössbauer parameters of the inline chloro-ferryl species correlate with the experimental parameters obtained for the Fe^IV^=O^second^ species.

## Conclusions

Our biochemical, kinetics, spectroscopic, and computational study revealed that AdeV catalyzes the conversion of 2′-deoxyadenosine monophosphate (2′-dAMP) to 2′-Cl-dAMP in a regio- and stereoselective manner. We establish that C–H bond cleavage and C–Cl bond formation occur in a suprafacial manner. This is the first time that the stereochemical information on halogenation is revealed in Fe/2OG halogenases. Furthermore, spectroscopic analysis showed that two chloro-ferryl intermediates were accumulated in a sequential manner with only the early intermediate capable of C–H activation, leading to both chloride radical (Cl•) rebound and hydroxyl radical (OH•) rebound. However, the Cl• rebound clearly outcompetes the OH• rebound, resulting in chlorination as the dominant reaction outcome. The late chloro-ferryl intermediate interconverts with the early one but does not participate in the C–H activation. The computational study further indicated that the early chloro-ferryl intermediate responsible for the halogenation chemistry is most likely an offline ferryl intermediate with the Fe^IV^=O moiety *trans* to the iron-bound histidine close to the N-terminus of the protein and the substrate C2′-(pro-*R*)H bond positioned perpendicular to the Fe^IV^=O moiety. In addition, the C–H activation step by this offline ferryl state features a unique offline-to-inline isomerization of the Fe^IV^=O moiety, which forms a metastable inline Fe^IV^=O, finally enabling C–H activation exclusively via a σ-pathway. The subsequent formation of an offline hydroxy-ferric state facilitates the final C–Cl bond formation. Thus, our current study shows that by combining stereochemical information on the enzyme reaction, spectroscopic and kinetic characterizations of the reactive intermediates, and computational analysis (MD and QM/MM), a detailed reaction mechanism and stereoselective origin of halogenation catalyzed by Fe/2OG halogenases can be derived. Overall, our study provides key insights into the understanding of the chlorination mechanism of AdeV and sets up the foundation to elucidate governing factors that lead to nonhydroxylation outcomes in Fe/2OG enzymes.

## Supplementary Material



## Notes

Additional experimental and computational details including heterologous protein expression and purification protocols, synthetic methods, LC–MS methods, ^1^H NMR and ^13^C NMR spectroscopic characterization results, stopped-flow optical absorption spectroscopic results, Mössbauer data analysis, and computational methods and results (PDF)
