# Correction to “Regio- and Stereoselective Halogenation by an Iron(II)- and 2‑Oxoglutarate-Dependent Halogenase in the Biosynthesis of Halogenated Nucleosides”

**DOI:** 10.1021/jacs.5c23050

**Published:** 2026-01-24

**Authors:** Philip M. Palacios, Xiaojun Li, Simahudeen Bathir Jaber Sathik Rifayee, Haoyu Tang, Tatyana Karabencheva-Christova, Christo Christov, Wei-chen Chang, Yisong Guo

**Affiliations:** † Department of Chemistry, 6612 Carnegie Mellon University, Pittsburgh, Pennsylvania 15213, United States; ‡ Department of Chemistry, 6798 North Carolina State University, Raleigh, North Carolina 27695, United States; § Department of Chemistry, 3968 Michigan Technological University, Houghton, Michigan 49931, United States

The following typographic error on one coauthor’s name is hereby corrected. This change does not alter the conclusions of the original work.

One coauthor’s name is appeared as “Xiaoyun Li” in the original work. It should be “Xiaojun Li”. Now this typographic error is corrected in the authorship of this Correction. The same typographic error in the Supporting Information is also corrected now.

## Supplementary Material



## Notes

Additional experimental and computational details including heterologous protein expression and purification protocols, synthetic methods, LC–MS methods, ^1^H NMR and ^13^C NMR spectroscopic characterization results, stopped-flow optical absorption spectroscopic results, Mössbauer data analysis, and computational methods and results (PDF)

