# Supplementary material for: Correction to “Regio- and Stereoselective Halogenation by an Iron(II)- and 2‑Oxoglutarate-Dependent Halogenase in the Biosynthesis of Halogenated Nucleosides”
Source: J Am Chem Soc. 2026 Jan 24;148(4):4775. doi: 10.1021/jacs.5c23050 (PMC12879729; doi:10.1021/jacs.5c23050)
Supplement: Supplementary file 1 [file ja5c23050_si_001.pdf]

Supporting Information

Regio- and Stereo-Selective Halogenation by an Iron(II)- and 2-Oxoglutarate-Dependent Halogenase in the Biosynthesis of Halogenated Nucleosides

Philip M. Palacios,[1,#] Xiaojun Li,[2,#] Simahudeen Bathir Jaber Sathik Rifayee,[3#] Haoyu Tang,[2] Tatyana Karabencheva-Christova,[3,*] Christo Christov,[3,*] Wei-chen Chang,[2,*] Yisong Guo [1,*]

1. Department of Chemistry, Carnegie Mellon University, Pittsburgh, Pennsylvania, 15213, United States
2. Department of Chemistry, North Carolina State University, Raleigh, North Carolina, 27695, United States
3. Department of Chemistry, Michigan Technological University, Houghton, Michigan, 49931, United States

TABLE OF CONTENTS

SUPPLEMENTARY METHODS S5

General Information S5
Anaerobic techniques S5
Plasmid Construction of AdeV S5
Overexpression and Purification of AdeV S6
Invitro Assays for LC-MS Analysis S7
Preparative scale AdeV reaction and product isolation S7
Structure Determination of 4 .... S8
Preparation of Compound 2 and epi-2 .... S9
Preparation of Compound 3 and $^2H_2$-3 .... S16
Stopped-flow absorption spectroscopy of AdeV S20
Freeze-quench Mössbauer Sample Preparation S21
Mössbauer Analysis S21
Rapid Chemical Quench Sample Preparation S22
Computational Methods S22
System Preparation S22
Molecular Dynamics Simulations S23
QM/MM Calculations S24
Mössbauer Calculations S25
Alternate Substrate Binding Position in AdeV. S26

SUPPLEMENTARY FIGURES S27

Figure S1. Comassie-stained SDS-PAGE (12%) of His6-tagged purification of AdeV. S27
*Figure S2. Optical spectrum of anaerobic AdeV solution incubated with Fe^{2+}, 2OG, Cl^-, and **1**.* S27
Figure S3. Selected time dependent difference optical spectra of the reaction of the AdeV•Fe^{2+}•2OG•Cl^-•1 complex with O_2. .... S28
Figure S4. 4.2 K zero field Mössbauer spectra recorded on the freeze-quench AdeV samples at different reaction time points. S29
Figure S5. The kinetic simulations for the observed formation rates of the chloro-ferryl intermediate (310 nm) with varying substrate concentration. S30
Figure S6. ^1H, ^{13}C, DEPT135, HSQC, COSY, HMBC and NOESY NMR of (1'R, 2'R, 4'S)-2'-chloro-2',3'-dideoxyadenosine monophosphate. S33
Figure S7. ^1H, ^{13}C and ^{19}F NMR of (6aR,8R,9S,9aR)-8-(6-benzamido-9H-purin-9-yl)-2,2,4,4-tetraisopropyltetrahydro-6H-furo[3,2-f][1,3,5,2,4]trioxadisilocin-9-yl trifluoromethanesulfonate. S35
Figure S8. ^1H and ^{13}C NMR of N-(9-((6aR,8R,9R,9aR)-9-chloro-2,2,4,4-tetraisopropyltetrahydro-6H-furo[3,2-f][1,3,5,2,4]trioxadisilocin-8-yl)-9H-purin-6-yl)benzamide. S36
Figure S9. ^1H and ^{13}C NMR of N-(9-((2R,3R,4R,5R)-3-chloro-4-hydroxy-5-(hydroxymethyl)tetrahydrofuran-2-yl)-9H-purin-6-yl)benzamide. S37
Figure S10. ^1H and ^{13}C NMR of N-(9-((2R,3R,4R,5R)-4-((tert-butyldimethylsilyl)oxy)-5-(((tert-butyldimethylsilyl)oxy)methyl)-3-chlorotetrahydrofuran-2-yl)-9H-purin-6-yl)benzamide. S38
Figure S11. ^1H and ^{13}C NMR of 9-((2R,3R,4R,5R)-4-((tert-butyldimethylsilyl)oxy)-5-(((tert-butyldimethylsilyl)-oxy)methyl)-3-chlorotetrahydrofuran-2-yl)-9H-purin-6-amine. S39
Figure S12. ^1H and ^{13}C NMR of ((2R,3R,4R,5R)-5-(6-amino-9H-purin-9-yl)-3-((tert-butyldimethylsilyl)oxy)-4-chlorotetrahydrofuran-2-yl)methanol. S40
Figure S13. ^1H ^{13}C and ^{31}P NMR of ((2R,3R,4R,5R)-5-(6-amino-9H-purin-9-yl)-4-chloro-3-hydroxy-tetrahydrofuran-2-yl)methyl dibenzyl phosphate. S42
Figure S14. ^1H ^{13}C and ^{31}P NMR of ((2R,3R,4R,5R)-5-(6-amino-9H-purin-9-yl)-4-chloro-3-hydroxytetrahydrofuran-2-yl)methyl dihydrogen phosphate. S44
Figure S15. ^1H ^{13}C and ^{19}F NMR of (6aR,8R,9R,9aR)-8-(6-benzamido-9H-purin-9-yl)-2,2,4,4-tetraisopropyltetrahydro-6H-furo[3,2-f][1,3,5,2,4]trioxadisilocin-9-yl trifluoromethanesulfonate. S46

Figure S16. ^{1}H and ^{13}C NMR of N-(9-((2R,3S,4R,5R)-3-chloro-4-hydroxy-5-(hydroxymethyl)tetrahydrofuran-2-yl)-9H-purin-6-yl)benzamide. S47
Figure S17. ^{1}H and ^{13}C NMR of N-(9-((2R,3S,4R,5R)-4-((tert-butyldimethylsilyl)oxy)-5-(((tert-butyldimethylsilyl)oxy)methyl)-3-chlorotetrahydrofuran-2-yl)-9H-purin-6-yl)benzamide. S48
Figure S18. ^{1}H and ^{13}C NMR of 9-((2R,3S,4R,5R)-4-((tert-butyldimethylsilyl)oxy)-5-(((tert-butyldimethylsilyl)oxy)methyl)-3-chlorotetrahydrofuran-2-yl)-9H-purin-6-amine. S49
Figure S19. ^{1}H NMR of ((2R,3R,4S,5R)-5-(6-amino-9H-purin-9-yl)-3-((tert-butyldimethylsilyl)oxy)-4-chlorotetrahydrofuran-2-yl)methanol S50
Figure S20. ^{1}H and ^{31}P NMR of ((2R,3R,4S,5R)-5-(6-amino-9H-purin-9-yl)-3-((tert-butyldimethylsilyl)oxy)-4-chlorotetrahydrofuran-2-yl)methanol S51
Figure S21. ^{1}H ^{13}C and ^{31}P NMR of ((2R,3R,4S,5R)-5-(6-amino-9H-purin-9-yl)-4-chloro-3-hydroxy-tetrahydrofuran-2-yl)methyl dibenzyl phosphate. S53
Figure S22. ^{1}H, ^{13}C and ^{31}P NMR of ((2R,3R,4S,5R)-5-(6-amino-9H-purin-9-yl)-4-chloro-3-hydroxytetrahydro-furan-2-yl)methyl dihydrogen phosphate. S55
Figure S23. ^{1}H, ^{13}C and ^{31}P NMR of enzymatic product ((2R,3R,4R,5R)-5-(6-amino-9H-purin-9-yl)-4-chloro-3-hydroxytetra-hydrofuran-2-yl)methyl dihydrogen phosphate. S57
Figure S24. ^{1}H and ^{13}C NMR of enzymatic product and synthetic standard ((2R,3R,4R,5R)-5-(6-amino-9H-purin-9-yl)-4-chloro-3-hydroxytetra-hydrofuran-2-yl)methyl dihydrogen phosphate. S58
Figure S25. ^{1}H, ^{13}C, DEPT135, HSQC, COSY, NOESY and 31P NMR of enzymatic product ((2S,4R,5R)-5-(6-amino-9H-purin-9-yl)-4-chlorotetrahydrofuran-2-yl)methyl dihydrogen phosphate. S62
Figure S26. ^{1}H and ^{13}C NMR of (2R,3R,4S,5R)-2-(6-amino-9H-purin-9-yl)-5-(((tert-butyldimethylsilyl)oxy)methyl)tetrahydrofuran-3,4-diol. S63
Figure S27. ^{1}H and ^{13}C NMR of (3aR,4R,6R,6aR)-4-(6-amino-9H-purin-9-yl)-6-(((tert-butyldimethylsilyl)oxy)methyl) tetra-hydrofuro[3,4-d][1,3]dioxole-2-thione. S64
Figure S28. ^{1}H and ^{13}C NMR of 9-((2R,5S)-5-(((tert-butyldimethylsilyl)oxy)methyl)-2,5-dihydrofuran-2-yl)-9H-purin-6-amine. S65
Figure S29. ^{1}H and ^{13}C NMR of 9-((2R,5S)-5-(((tert-butyldimethylsilyl)oxy)methyl)-tetrahydrofuran-2-yl)-9H-purin-6-amine. S66
Figure S30. ^{1}H ^{13}C and ^{31}P NMR of ((2S,5R)-5-(6-amino-9H-purin-9-yl)tetrahydrofuran-2-yl)methyl dibenzyl phosphate. S68
Figure S31. ^{1}H,^{13}C and ^{31}P NMR of ((2S,5R)-5-(6-amino-9H-purin-9-yl)tetrahydrofuran-2-yl)methyl dihydrogen phosphate. S70
Figure S32. ^{1}H and ^{13}C NMR of 9-((2R,3R,4S,5S)-5-(((tert-butyldimethylsilyl)oxy)methyl)-tetrahydrofuran-2-yl-3,4-d2)-9H-purin-6-amine. S71
Figure S33. ^{1}H, ^{13}C and ^{31}P NMR of ((2S,3S,4R,5R)-5-(6-amino-9H-purin-9-yl)tetrahydrofuran-2-yl-3,4-d2)methyl dibenzyl phosphate. S73
Figure S34. ^{1}H, ^{13}C and ^{31}P NMR of ((2S,3S,4R,5R)-5-(6-amino-9H-purin-9-yl)tetrahydrofuran-2-yl-3,4-d2)methyl dibenzyl phosphate. S75
Figure S35. Overlaid RC and InTS1 QM/MM optimized structures obtained using QM1 (cyan) and QM2 region with Q201 (green) of the Off2-RC snapshot. S76
Figure S36. Molecular dynamics analysis of the Offline FeIII-superoxo complex. S77
Figure S37. Molecular dynamics analysis of the inline FeIII-superoxo complex. S78
Figure S38. Interactions stabilizing the 2OG in the offline Fe^{III}-superoxo dynamics. S79
Figure S39. Interactions stabilizing the 2OG in inline Fe^{III}-superoxo dynamics. S80
Figure S40. The overall protein dynamics of the offline and inline FeIII-superoxo systems. S81
Figure S41. The reaction profile and molecular structures of the iron center derived from QM/MM calculations. S82
Figure S42. The reaction profile and molecular structures of the iron center derived from QM/MM calculations. S83
Figure S43. Molecular dynamics analysis of the Offline ferryl complex. S84
Figure S44. Molecular dynamics analysis of the Inline ferryl complex. S85
Figure S45. Plots depicting the fluctuations of the distance between the ferryl oxygen and C2' of the substrate in a) offline and b) inline systems. S86

Figure S46. Reaction profile and representation of QM/MM optimized structures of halogenation and hydroxylation mechanism in the Inline ferryl system. S87
Figure S47. The change from offline to inline orientation during HAT S88
Figure S48. The spin density plots of a) Off1-IM1 and b) In1-IM1. ....S88
Figure S49. Reaction profile of Pro-R and Pro-S HAT from Off1-RC. Relative energies are given in kcal/mol calculated at zero-point corrected energies (QM(B3)/MM level). S89
Figure S50. Reaction profile of Pro-R and Pro-S HAT from In1-RC. Relative energies are given in kcal/mol calculated at zero-point corrected energies (QM(B3)/MM level). S89
Figure S51. QM/MM optimized structures of initial RCs obtained from the offline ferryl system. S90
Figure S52. QM/MM optimized structures of initial RCs obtained from the inline ferryl system. S91
Figure S54. Alternative substrate binding configuration. S92

SUPPLEMENTARY TABLES **S93**

Table S1. ....S93
Table S2. ....S93
Table S3. ....S93
Table S4 ....S94
Table S5 ....S94
Table S6. ....S95
Table S7. ....S95
Table S8. ....S96
Table S9. ....S96
Table S10. ....S97
Table S11. ....S97
Table S12. ....S97
Table S14. ....S98
Table S15. ....S99
Table S16. ....S99
Table S17. ....S100
Table S18. ....S100
Table S19. ....S101
Table S20. ....S101
Table S21. ....S102
Table S22. ....S102
Table S23. ....S102

REFERENCES: **S104**

COORDINATES OF QM/MM OPTIMIZED GEOMETRIES **S107**

Supplementary Methods

General Information

Unless specified otherwise, chemicals and solvents were purchased from suppliers and used without further purification. NMR spectra were recorded on Bruker NEO-500, Bruker NEO-600 or Bruker avance III 700 MHz spectrometers. The chemical shift (δ)of ^{1}H NMR and ^{13}C NMR is given in ppm relative to solvent residual peak. Column chromatography was carried out using 300–400 mesh silica gel. Thin layer chromatography (TLC) analysis was carried out on SilicycleTM aluminium backed TLC plates and visualized using alkaline potassium manganate solution, ninhydrin staining solution and/or UV light at 254 nm.

Anaerobic techniques

Samples for spectroscopic characterization, especially those that might be oxygen sensitive, were handled in an MBraun UNIlab glovebox circulated under a positive pressure of N_2(g) maintaining <0.5 ppm O_2(g). AdeV was rendered anoxic via the purification method outlined in the "Overexpression and Purification of AdeV" section. All other buffers and compounds were prepared within the glovebox to obtain uniform anaerobic conditions.

Plasmid Construction of AdeV

The DNA sequence presented below encodes the AdeV gene from Actinomadura sp. ATCC 39365 was codon-optimized for *E. coli* overexpression, synthesized, and inserted between the NdeI and BamHI restriction sites in the pET-28a vector by Genscript (Piscataway, NJ). This plasmid construct contains an N-terminal poly-histidine (His_6) affinity tag.

```
ATGGACGTCCCTCTCATGGAACTCAGCGGCCGCGCCCCCGTCGTCAGGCTGCATGACATCGAGGCGGACA
TGGCCGCCGCCACCGACGCCATCAGGTCGCAGCTGACCGGATGGGGCTTCATGGCCGCGGAGGTGCCCGG
CATCGGCGAGCGCGTCGAGGCCATGATGAACGAGTTCGCCGCGGCCTGCCGGGCGACCGGGCCGAGCCTG
TCCGACTACGCCTACGACGTCGTCCCGCAGCTCGCCGTCGGCGGCACGCACGGGTTCTTCCCGTACAACT
CGGAGATCCCGCGCCTGGCCAACGGCGTGCCCGACCCGAAGGAGTTCATCCACGTCAGCGGCGCCATGAT
CGGCGACCAGCCGCCCGGGGCGGGTGACGTGCTGCGGGCCTTCCCGGCGTTCGGCACCCGCGCCGCCGAG
GTGTTCGACATCGCCTTCCGGCTGATCTCGCTCTTCGGCGAGGTCGTCCGGGGCATGATGCCGCCCGGCA
CGCCGGAGCTGGACCTCTCGCACGACGCGACGAACCTGCGGGTGATCCACTACCGGGACGTCGGCGACCG
CGAGGTGCTGGCCCACGAGCACTCCGGCATCCAGATGCTCGGCCTCCAGCTGCCCCCGTCCGACCAGGGC
CTGCAGTACGTGCTGCACGACGGCACCTGGGTCGAGCCGGTGATCGCCGGGACCGACGTCGTGCTGTGCA
ACATCGGCCGGATGCTCACCAGCGCCTCCGACGGCGGTTCCGGCCGTCCACGCACCGGGTGCACACCAA
GCCGATGCCGGCCGGCTACGAGCGCCTGTCGTCGGTGCTCTTCGCCTACCCGCAGCACAAGGCCCGCCAG
TGGAAGATGGTGGACGGCGAGCTGATGTCGCTGAACGCCACCTGGGGCGACTTCATCGACAGCCGCTTCC
AGGGGCTCGGCAAGCAGTCCTGA
```

Overexpression and Purification of AdeV

The plasmid containing the AdeV gene was transformed into *E. coli* BL21(DE3) cells (New England Biolabs, MA) and grown overnight on a LB medium agar plate containing kanamycin (50 μg/ml). A single colony was transferred to 5 mL of LB growth medium containing kanamycin (50 μg/ml) and incubated 4 h (37 °C, 225 rpm) and then 100 uL was used to inoculate 100 mL of LB growth medium containing kanamycin (50 μg/ml) that was grown overnight (37 °C, 50 rpm). The overnight culture was used to inoculate 1 L of LB growth medium containing kanamycin (50 μg/mL) and 2 drops of Antifoam 204 (Sigma-Aldrich) at 37 °C and 225 rpm. At an OD_{600} of 0.4 – 0.6, the cultures were cooled in an ice-water bath for 15 minutes and 0.5 mM IPTG was added, and shaking was continued at 16 °C for 18 h. The cells were pelleted by centrifugation at 6500 x *g* and the cell paste was stored at -80 °C. The cell paste was thawed and resuspended in chilled buffer with 20 mM HEPES (pH 8.0) containing lysozyme and benzonase nuclease. The slurry mixture was mixed by gentle stirring followed by sonication. The lysate was centrifuged for 60 minutes at 30,000 x *g* at 4 °C and the supernatant was loaded onto a pre-equilibrated Ni-NTA agarose column. The column was washed with 5 column volumes of buffer containing 20 mM HEPES (pH 8.0), 20 mM imidazole followed by elution with 3 column volumes of 350 mM imidazole-containing buffer. Elution fractions were analyzed by UV-vis spectroscopy and SDS-PAGE. Fractions containing AdeV were pooled and concentrated to 10 mL using an Amicon centrifugal filter (30 kDa cutoff). The protein solution was dialyzed overnight against 1 L of buffer containing 20 mM HEPES (pH 8.0), 5 mM EDTA then buffer exchanged by centrifugation against EDTA-free buffer (20 mM HEPES, pH 8.0). The protein solution was concentrated to 80 – 100 mg/mL and was deoxygenated by flowing argon gas through the headspace with gentle stirring in an ice-water bath. The degassed protein solution was transferred into an anaerobic glovebox, aliquoted, and flash-frozen in liquid nitrogen. Protein concentration was determined by UV-vis absorption at 280 nm using the calculated molar extinction coefficient of 32,500 $M^{-1}cm^{-1}$ (http://web.expasy.org/compute_pi/). The purity of the protein purification for AdeV can be seen in the SDS-PAGE gel (12%) in Figure S1.

Invitro Assays for LC-MS Analysis

Enzymatic reactions were performed in 200 μL 50 mM HEPES (pH 7.5) with final concentrations of 0.11 mM apo-AdeV, 0.1 mM ferrous ammonium sulfate, 10 mM 2OG and 5 mM substrate and 50 mM of sodium chloride for 12 h at 4 °C. The reactions were then quenched with an equal volume of acetonitrile. After centrifugation (14,000 rpm, 30 mins), the supernatant was used for liquid chromatography coupled mass spectroscopy (LC-MS) analysis.

Products were characterized using an Agilent Technologies (Santa Clara, CA) 1290 Infinity II system with an Agilent Technologies 6530 quadrupole time of flight mass spectrometer. Chromatographic separation utilized an Agilent InfinityLab Poroshell 120 hilic column (4.6 x 50 mm, 4.0 μm) eluted isocratically with 70% solvent A (0.1% formic acid in H_2O) and 30% solvent B (acetonitrile) at a flow rate of 0.6 mL/min. Instrument parameters were 250 °C gas temperature, 11 L/min gas flow, and 250 °C sheath gas temperature. Mass spectra were acquired under electrospray ionization in positive ion mode (ESI^+) and analyzed using the Agilent MassHunter software.

Preparative scale AdeV reaction and product isolation

Reaction mixtures containing AdeV, ferrous ammonium sulfate, 2OG, *L*-ascorbic acid, NaCl and substrate (((*2R,3S,5R*)-5-(6-amino-9*H*-purin-9-yl)-3-hydroxytetrahydrofuran-2-yl)methyl dihydrogen phosphate) (**1**) with the final concentration of 22 μM AdeV, 20 μM iron, 8 mM 2OG, 8 mM *L*-ascorbic acid, 40 mM NaCl and 4 mM substrate with final volume of 14 mL in 100 mM Tris (pH = 7.54) were prepared. The reaction was conducted at 4 ˚C with stirring (50 rpm) for 24 h. The reactions were halted by adding acetonitrile (5 mL) and subjected to centrifugation (14,000 rpm, 30 mins) to precipitate the protein. The solvent was removed by lyophilization. The residual white solid was dissolved in 2 mL deionized water and purified by preparative HPLC (Waters Sunfire C18 column (10x250 mm, 5 μm, 100 Å) using isocratic elution conditions (15% acetonitrile + 85% water with 0.1% ammonia formate) to give ((*2R,3R,4R,5R*)-5-(6-amino-9*H*-purin-9-yl)-4-chloro-3-hydroxytetra-hydrofuran-2-yl)methyl dihydrogen phosphate (**2**) as a white solid (5 mg). The structures were elucidated by NMR and comparison with the synthetic standard. ^{1}H NMR (700 MHz, D_2O) δ 8.51 (s, 1H), 8.27 (s, 1H), 6.35 (d, *J* = 6.1 Hz, 1H), 4.98 (t, *J* = 5.4 Hz, 1H), 4.69 (t, *J* = 4.8 Hz, 1H), 4.47 (p, *J* = 2.6 Hz, 1H), 4.20 (ddd, *J* = 11.6, 4.6, 2.7 Hz, 1H), 4.15 (ddd, *J* = 11.8, 4.9,

2.8 Hz, 1H); ^{13}C NMR (175 MHz, D_2O) δ 155.1, 152.1, 148.9, 139.9, 118.7, 88.1, 84.0 (d, *J* = 8.8 Hz), 70.3, 63.9 (d, *J* = 4.8 Hz), 61.5; ^{31}P NMR (240 MHz, D_2O) δ 0.3.

Reaction mixtures containing AdeV, Fe(II), 2OG, *L*-ascorbic acid, NaCl and substrate ((2*S*,5*R*)-5-(6-amino-9*H*-purin-9-yl)tetrahydrofuran-2-yl)methyl dihydrogen phosphate (**3**) with the final concentration of 88 μM AdeV, 80 μM iron, 8 mM 2OG, 8 mM *L*-ascorbic acid, 40 mM NaCl and 4 mM substrate with final volume of 20 mL in 50 mM Tris (pH = 7.54) were prepared. The reaction was conducted at 4 ˚C with stirring (50 rpm) for 96 h. The reactions were halted by adding acetonitrile (5 mL) and centrifuged at 14,000 rpm for 30 mins to precipitate the protein. The solvent was removed by lyophilization. The residual white solid was dissolved in 2 mL deionized water and purified by preparative HPLC (Waters Sunfire C18 column (10x250 mm, 5 μm, 100 Å) using isocratic elution conditions (15% acetonitrile + 85% water with 0.1% ammonia formate) to give ((2*S*,4*R*,5*R*)-5-(6-amino-9*H*-purin-9-yl)-4-chlorotetrahydrofuran-2-yl)methyl dihydrogen phosphate (**4**) as a white solid (29 mg). ^{1}H NMR (700 MHz, Deuterium Oxide) δ 8.48 (s, 1H), 8.23 (s, 1H), 6.33 (d, *J* = 2.3 Hz, 1H), 4.99 – 4.89 (m, 1H), 4.85 (ddd, *J* = 9.3, 6.3, 3.0 Hz, 1H), 4.29 (ddd, *J* = 11.9, 4.8, 2.5 Hz, 1H), 4.09 (ddd, *J* = 11.8, 5.8, 3.5 Hz, 1H), 2.68 (ddd, *J* = 14.7, 9.1, 6.0 Hz, 1H), 2.46 (ddd, *J* = 14.4, 6.2, 3.0 Hz, 1H). ^{13}C NMR (175 MHz, D_2O) δ 154.4, 151.0, 148.0, 139.8, 118.6, 91.1, 80.2 (d, *J* = 8.8 Hz), 64.9 (d, *J* = 4.7 Hz), 60.25, 34.05; ^{31}P NMR (240 MHz, D_2O) δ 0.4.

Structure Determination of 4

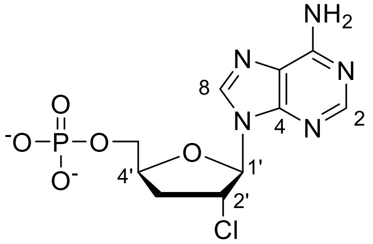

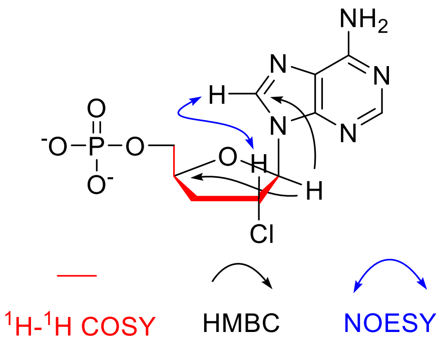

Compound **4** was isolated as amorphous solid, ^{1}H and ^{13}C NMR spectrums showed similar resonances of adenosine and compound **3.** The character signals of δ_H 8.53 (1H, s), 8.16 (1H, s) and δ_C 156.0, 152.8, 148.9, 138.7, 118.8 indicated an adenine moiety. Furthermore, ^{13}C, DEPT135 and HSQC displayed a hemiaminal methine (δ_H 6.21, δ_C 90.5), an oxygenated methine (δ_H 4.56, δ_C 79.7),

a chlorinated methine (δ_H 5.01, δ_C 61.2) and an oxygenated methylene (δ_H 4.01, 3.86, δ_C 64.2). The correlations of ^{1}H-^{1}H COSY spectrum suggested a spin-spin system of 1′-H/2′-H/3′-H/4′-H/5′-H and gave a 2′-chloro-(4*H*)-furan moiety confirmed by a key HMBC correlation of 1′-H/C-4′. The HMBC correlation from 1′-H to C-8 verified adenine attached to C-1′. The NOESY correlation of 2′-H/8-H indicated 2′-H faced to the same direction of adenine moiety and determined the stereochemistry of C-2′ as *R* configuration since the chirality of C-1′ is *R*. Consequently, compound **4** was characterized as (1′*R*, 2′*R*, 4′*S*)-2′-chloro-2′,3′-dideoxyadenosine monophosphate.

^{1}H NMR (500 MHz, DMSO-*d*$_6$) δ 8.53 (1H, s, 8-H), 8.16 (1H, s, 2-H), 6.21 (1H, d, 2.3, 1′-H), 5.01 (1H, ddd, 5.6, 2.3, 3.0, 2′-H), 4.56 (1H, m, 4′-H), 4.01 (1H, m, 5′-H), 3.86 (1H, m, 5′-H), 2.65 (1H, ddd, 14.3, 8.6, 6.0, 3′-H), 2.28 (1H, ddd, 14.2, 5.9, 3.0, 3′-H). ^{13}H NMR (125 MHz, DMSO-*d*$_6$) δ 156.0 (C), 152.8 (CH), 148.9 (C), 138.7 (CH), 118.8 (C), 90.5 (CH), 79.7 (CH), 64.2 (CH_2), 61.2 (CH), 34.8 (CH_2)

Preparation of Compound 2 and epi-2

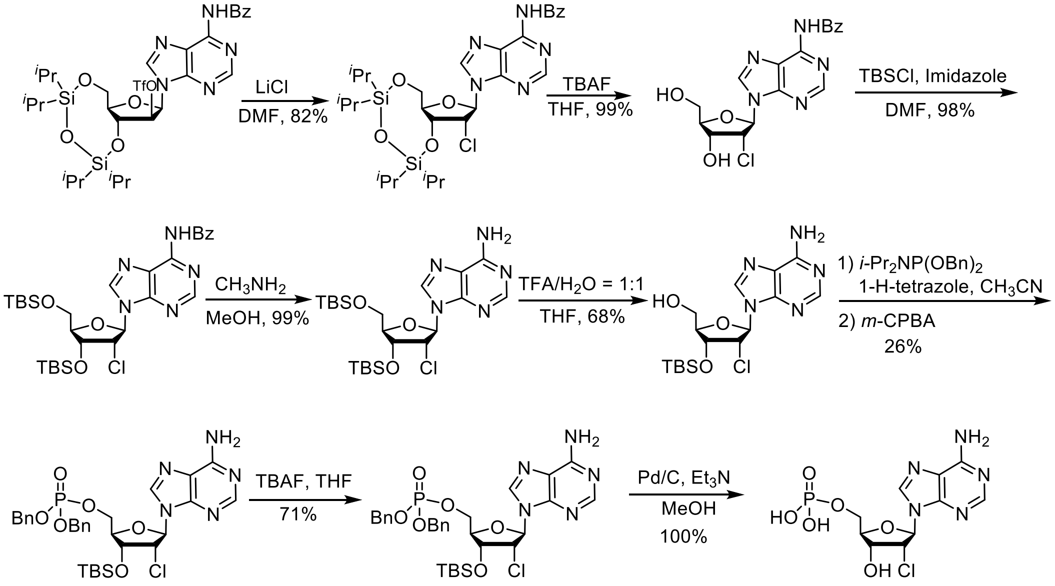

(*6aR,8R,9S,9aR*)-8-(6-benzamido-9*H*-purin-9-yl)-2,2,4,4-tetraisopropyl-tetrahydro-6*H*-furo[3,2-f][1,3,5,2,4]trioxadisilocin-9-yl trifluoromethane-sulfonate was prepared following the reported procedure (2.03 g, 66%, colorless oil).[1] ^{1}H NMR (500 MHz, $CDCl_3$) δ 9.49 (br, 1H), 8.69 (s, 1H), 8.11 (s, 1H), 7.95 (t, *d* = 7.6 Hz, 2H), 7.50 (t, *J* = 7.4 Hz, 1H), 7.41 (t, *J* = 7.6 Hz, 2H), 6.45 (d, *J* = 6.2 Hz, 1H), 5.48 (t, *J* = 6.6 Hz, 1H), 5.41 (t, *J* = 7.3 Hz, 1H), 4.18 (dd, *J* = 12.4, 5.8 Hz, 1H), 4.03 (dd, *J* = 12.4,

3.3 Hz, 1H), 3.93 (ddd, *J* = 8.6, 5.8, 3.2 Hz, 1H), 1.25 – 0.89 (m, 28H); ^{13}C NMR (125 MHz, $CDCl_3$) δ 164.7, 152.9, 151.2, 149.8, 142.0, 133.5, 132.5, 128.6, 127.7, 123.1, 117.98 (q, *J* = 320.1 Hz), 88.2, 80.8, 80.7, 73.8, 61.7, 17.2, 17.1, 17.09, 17.05, 16.6, 16.53, 16.52, 16.49, 13.0, 12.83, 12.79, 12.4; ^{19}F NMR (471 MHz, $CDCl_3$) δ -74.4.

To a solution of (*6aR,8R,9S,9aR*)-8-(6-benzamido-9*H*-purin-9-yl)-2,2,4,4-tetraisopropyl-tetrahydro-6*H*-furo[3,2-f][1,3,5,2,4]trioxadisilocin-9-yl trifluoromethane-sulfonate (2.03 g, 2.72 mmol) in DMF (54 mL) was added LiCl (1.15 g, 27.2 mmol, 10.0 equiv.) at room temperature. Then the reaction mixture was heated to 50 °C and stirred for 24 h. After cooling to room temperature, the mixture was diluted by water and extracted with ethyl acetate, the organic lawyer was washed with brine, dried over $MgSO_4$, and concentrated under reduced pressure. The residue was purified by a flash column chromatography on silica gel (20% to 40% Ethyl acetate /Hexane (v/v)) to give *N*-(9-((*6aR,8R,9R,9aR*)-9-chloro-2,2,4,4-tetraisopropyltetrahydro-6*H*-furo-[3,2-*f*][1,3,5,2,4]trioxadisilocin-8-yl)-9*H*-purin-6-yl)benzamide as a white solid (1.4 g, 82%). ^{1}H NMR (500 MHz, $CDCl_3$) δ 9.57 (br, 1H), 8.63 (s, 1H), 8.22 (s, 1H), 7.93 (d, *J* = 7.7 Hz, 2H), 7.47 (t, *J* = 7.4 Hz, 1H), 7.38 (t, *J* = 7.7 Hz, 2H), 6.23 (s, 1H), 4.98 (dd, *J* = 8.8, 5.6 Hz, 1H), 4.84 (d, *J* = 5.6 Hz, 1H), 4.15 (dd, *J* = 10.9, 3.0 Hz, 1H), 4.12 (t, *J* = 2.7 Hz, 1H), 3.99 (dd, *J* = 13.3, 2.9 Hz, 1H), 1.22 – 0.84 (m, 28H); ^{13}C NMR (125 MHz, $CDCl_3$) δ 164.8, 152.4, 150.6, 149.6, 141.3, 133.4, 132.4, 128.4, 127.8, 123.6, 90.4, 81.8, 68.3, 62.3, 59.8, 17.2, 17.1, 17.02, 17.00, 16.8, 16.72, 16.71, 16.6, 13.1, 12.7, 12.5, 12.4.

To a solution of *N*-(9-((*6aR,8R,9R,9aR*)-9-chloro-2,2,4,4-tetraisopropyltetrahydro-6*H*-furo-[3,2-*f*][1,3,5,2,4]trioxadisilocin-8-yl)-9*H*-purin-6-yl)benzamide (1.4 g, 2.21 mmol) in THF (44 mL) was added TBAF (1.0 M in THF, 2.2 mL, 2.21 mmol, 1.0 equiv.) at room temperature. Then the reaction mixture was stirred for 4 h at room temperature. The reaction mixture was concentrated under reduced pressure. The residue was purified by a flash column chromatography on silica gel (5% to 10% MeOH /DCM (v/v)) to give *N*-(9-((*2R,3R,4R,5R*)-3-chloro-4-hydroxy-5-(hydroxymethyl)tetrahydrofuran-2-yl)-9*H*-purin-6-yl)benzamide as a white solid (852 mg, 99%). ^{1}H NMR (500 MHz, DMSO-d_6) δ 10.35 (s, 1H), 7.91 (s, 1H), 7.88 (s, 1H), 7.19 (d, *J* = 7.2 Hz, 2H), 6.78 (t, *J* = 7.0 Hz, 1H), 6.69 (t, *J* = 7.7 Hz, 2H), 5.80 (d, *J* = 6.4 Hz, 1H), 5.30 (d, *J* = 5.7 Hz, 1H), 4.41 (t, *J* = 5.3 Hz, 1H), 4.01 (dd, *J* = 8.0, 6.4 Hz, 1H), 3.67 (td, *J* = 7.8, 5.7 Hz, 1H), 3.03 (ddd, *J* = 7.4, 4.2,

2.8 Hz, 1H), 2.95 (ddd, *J* = 12.3, 5.3, 2.8 Hz, 1H), 2.88 (dt, *J* = 12.3, 4.9 Hz, 1H); ^{13}C NMR (125 MHz, DMSO) δ 165.6, 152.1, 151.8, 150.4, 142.8, 133.3, 132.5, 128.5, 125.1, 83.7, 82.7, 73.9, 63.6, 59.8.

To a solution of *N*-(9-((*2R,3R,4R,5R*)-3-chloro-4-hydroxy-5-(hydroxymethyl)tetrahydrofuran-2-yl)-9*H*-purin-6-yl)benzamide (3.0 g, 11.9 mmol) in DMF (30 mL) was added Imidazole (5.35 g, 78.5 mmol, 6.6 equiv.) and TBSCl (5.73 g, 38 mmol, 3.2 equiv.) at room temperature. Then the reaction mixture was stirred for 24 h at room temperature. The mixture was concentrated under reduced pressure. The residue was purified by a flash column chromatography on silica gel (100% Ethyl acetate to 0.5% MeOH /DCM (v/v)) to give *N*-(9-((*2R,3R,4R,5R*)-4-((*tert*-butyldimethylsilyl)oxy)-5-(((*tert*-butyldimethylsilyl)oxy)methyl)-3-chlorotetrahydrofuran-2-yl)-9*H*-purin-6-yl)benzamide as a colorless oil (7.2 g, 98%). ^{1}H NMR (500 MHz, $CDCl_3$) δ 9.66 (br, 1H), 8.70 (s, 1H), 8.33 (s, 1H), 7.96 (d, *J* = 8.0 Hz, 2H), 7.46 (t, *J* = 7.5 Hz, 1H), 7.38 (t, *J* = 7.7 Hz, 2H), 6.59 (d, *J* = 5.4 Hz, 1H), 4.58 (t, *J* = 4.9 Hz, 1H), 4.46 (t, *J* = 5.1 Hz, 1H), 3.93 (dt, *J* = 8.3, 4.5 Hz, 2H), 3.88 – 3.83 (m, 1H), 0.88 (s, 9H), 0.87 (s, 9H), 0.12 (s, 3H), 0.10 (s, 3H), 0.07 (s, 6H); ^{13}C NMR (125 MHz, $CDCl_3$) δ 164.9, 152.4, 151.2, 149.5, 141.5, 133.6, 132.4, 128.5, 127.8, 122.6, 84.9, 83.6, 63.7, 61.5, 25.8, 25.5, 18.3, 17.7, -4.5, -5.0, -5.51, -5.52.

To a solution of *N*-(9-((*2R,3R,4R,5R*)-4-((*tert*-butyldimethylsilyl)oxy)-5-(((*tert*-butyldimethylsilyl)oxy)methyl)-3-chlorotetrahydrofuran-2-yl)-9*H*-purin-6-yl)benzamide (1.2 g, 1.94 mmol) in MeOH (30 mL) was added methylamine (40% aq., 15 mL) at room temperature. Then the reaction mixture was stirred for 4 h at room temperature. The reaction mixture was concentrated under reduced pressure. The residue was purified by a flash column chromatography on silica gel (3% to 5% MeOH /DCM (v/v)) to give 9-((*2R,3R,4R,5R*)-4-((*tert*-butyldimethylsilyl)oxy)-5-(((*tert*-butyldimethylsilyl)oxy)methyl)-3-chlorotetrahydrofuran-2-yl)-9*H*-purin-6-amine as a colorless oil (1.03 g, 99%). ^{1}H NMR (500 MHz, $CDCl_3$) δ 8.30 (s, 1H), 8.05 (s, 1H), 6.78 (s, 2H), 6.19 (d, *J* = 5.8 Hz, 1H), 4.98 (t, *J* = 5.3 Hz, 1H), 4.57 (t, *J* = 4.2 Hz, 1H), 4.14 (q, *J* = 3.7 Hz, 1H), 3.94 (dd, *J* = 11.4, 4.3 Hz, 1H), 3.73 (dd, *J* = 11.4, 3.1 Hz, 1H), 0.89 (s, 9H), 0.85 (s,9H), 0.11 (s, 3H), 0.09 (s, 3H), 0.03 (d, *J* = 1.6 Hz, 6H); ^{13}C NMR (125 MHz, $CDCl_3$) δ 155.9, 153.0, 149.6, 139.0, 120.0, 89.0, 85.7, 71.4, 62.0, 60.8, 25.8, 25.6, 18.2, 18.0, -4.8, -5.0, -5.5, -5.6.

To a solution of 9-((*2R,3R,4R,5R*)-4-((*tert*-butyldimethylsilyl)oxy)-5-(((*tert*-butyldimethylsilyl)oxy)methyl)-3-chlorotetrahydrofuran-2-yl)-9*H*-purin-6-amine (1.03 g, 2.0

mmol) in THF (25 mL) was added trifluoroacetic acid (50% aq., 10 mL) at 0 °C. Then the reaction mixture was warmed to room temperature and stirred for 4 h. Then $NH_3 \cdot H_2O$ was added to adjusted to pH = 10. The reaction mixture was concentrated under reduced pressure. The residue was purified by a flash column chromatography on silica gel (3% to 5% MeOH /DCM (v/v)) to give ((2*R*,3*R*,4*R*,5*R*)-5-(6-amino-9*H*-purin-9-yl)-3-((*tert*-butyldimethylsilyl)oxy)-4-chlorotetrahydro-furan-2-yl)methanol as a white solid (540 mg, 68%). ^{1}H NMR (500 MHz, $CDCl_3$) δ 8.31 (s, 1H), 7.88 (s, 1H), 6.15 (s, 2H), 5.95 (d, *J* = 8.7 Hz, 1H), 5.10 (dd, *J* = 8.7, 4.5 Hz, 1H), 4.53 (d, *J* = 4.5 Hz, 1H), 4.28 (d, *J* = 1.7 Hz, 1H), 3.98 (dd, *J* = 13.2, 1.6 Hz, 1H), 3.74 (dd, *J* = 13.2, 1.6 Hz, 1H), 0.95 (s, 9H), 0.17 (s, 3H), 0.15 (s, 3H); ^{13}C NMR (125 MHz, $CDCl_3$) δ 156.0, 152.3, 148.4, 140.8, 121.3, 91.9, 90.0, 74.0, 62.8, 59.7, 25.7, 18.3, -4.7, -4.8.

((2*R*,3*R*,4*R*,5*R*)-5-(6-amino-9*H*-purin-9-yl)-3-((*tert*-butyldimethylsilyl)oxy)-4-chlorotetrahydro-furan-2-yl)methanol (550 mg, 1.38 mmol) was dried by azeotrope with anhydrous pyridine three times and then dissolved in anhydrous MeCN (13.8 mL) and cooled to 0 °C. The dibenzyl *N*,*N*-diisopropylphosphoramidite (548 mg, 1.59 mmol, 1.15 equiv.) was added followed by 5-(ethylthio)-1*H*-tetrazole (3% in MeCN, 5.6 g, 2.39 mmol, 1.73 equiv.) and the reaction mixture was allowed to warm to room temperature. The reaction mixture was stirred at room temperature for 2 h. *m*-CPBA (70% wt., 392 mg, 1.59 mmol, 1.15 equiv.) was added at 0 °C and the reaction mixture was allowed to warm to room temperature. The reaction mixture was stirred at room temperature for additional 2 h. Then the reaction mixture was diluted by ethyl acetate and washed with 1 M aq. $Na_2S_2O_3$, sat. aq. $NaHCO_3$ and brine, then dried over $MgSO_4$ and concentrated under reduced pressure. The residue was purified by a flash column chromatography on silica gel (1% to 2% MeOH/DCM (v/v)) to give ((2*R*,3*R*,4*R*,5*R*)-5-(6-amino-9*H*-purin-9-yl)-3-((*tert*-butyldimethylsilyl)oxy)-4-chlorotetrahydrofuran-2-yl)methyl dibenzyl phosphate as a colorless oil (237 mg, 26%).

To a solution of ((2*R*,3*R*,4*R*,5*R*)-5-(6-amino-9*H*-purin-9-yl)-3-((*tert*-butyldimethylsilyl)oxy)-4-chlorotetrahydrofuran-2-yl)methyl dibenzyl phosphate (230 mg, 0.36 mmol) in THF (10 mL) was added TBAF (1.0 M in THF, 0.36 mL, 0.36 mmol, 1.0 equiv.) at room temperature. Then the reaction mixture was stirred for 16 h. The reaction mixture was concentrated under reduced pressure. The residue was purified by a flash column chromatography on silica gel (2% to 4%

MeOH/DCM (v/v)) to give ((*2R,3R,4R,5R*)-5-(6-amino-9*H*-purin-9-yl)-4-chloro-3-hydroxytetra-hydrofuran-2-yl)methyl dibenzyl phosphate as a yellow oil (140 mg, 71%). ^{1}H NMR (500 MHz, MeOD) δ 8.22 (s, 1H), 8.16 (s, 1H), 7.34 – 7.17 (m, 10H), 6.22 (d, *J* = 6.1 Hz, 1H), 5.11 (t, *J* = 5.6 Hz, 1H), 4.99 (dd, *J* = 8.8, 1.8 Hz, 4H), 4.52 (dd, *J* = 5.2, 3.6 Hz, 1H), 4.35 – 4.22 (m, 3H); ^{13}C NMR (125 MHz, MeOD) δ 157.3, 154.1, 150.6, 141.2, 136.9 (d, J_{C-P} = 6.2 Hz), 129.7 (d, J_{C-P} = 1.6 Hz), 129.6 (d, J_{C-P} = 0.9 Hz), 129.09 (d, J_{C-P} = 3.4 Hz), 120.6, 90.4, 84.4 (d, J_{C-P} = 7.8 Hz), 71.4, 70.9 (d, J_{C-P} = 6.4 Hz), 67.9 (d, J_{C-P} = 5.6 Hz), 61.9; ^{31}P NMR (200 MHz, MeOD) δ -1.4.

To a solution of ((*2R,3R,4R,5R*)-5-(6-amino-9*H*-purin-9-yl)-4-chloro-3-hydroxytetra-hydrofuran-2-yl)methyl dibenzyl phosphate (140 mg, 0.26 mmol) in 5% solution of triethyl amine in methanol (13 mL) was added Pd/C (28 mg, 20%) at room temperature. Then the reaction mixture was stirred for 17 h under H_2 atmosphere. The reaction mixture was filtered by celite, and the filtrate was concentrated under reduced pressure to give ((*2R,3R,4R,5R*)-5-(6-amino-*9H*-purin-9-yl)-4-chloro-3-hydroxytetra-hydrofuran-2-yl)methyl dihydrogen phosphate as a white solid (94 mg, 100%). ^{1}H NMR (500 MHz, D_2O) δ 8.48 (s, 1H), 7.99 (s, 1H), 6.17 (d, *J* = 5.9 Hz, 1H), 4.88 (t, *J* = 5.5 Hz, 1H), 4.63 (t, *J* = 4.6 Hz, 1H), 4.39 (d, *J* = 4.2 Hz, 1H), 4.04 (dt, *J* = 12.2, 3.6 Hz, 1H), 4.00 (dt, *J* = 7.4, 6.0 Hz, 1H); ^{13}C NMR (125 MHz, D_2O) δ 155.1, 152.5, 148.4, 139.6, 118.1, 87.9, 84.4 (d, J_{C-P} = 8.7 Hz), 70.4, 62.9 (d, J_{C-P} = 4.0 Hz), 61.8; ^{31}P NMR (200 MHz, D_2O) δ 3.7.

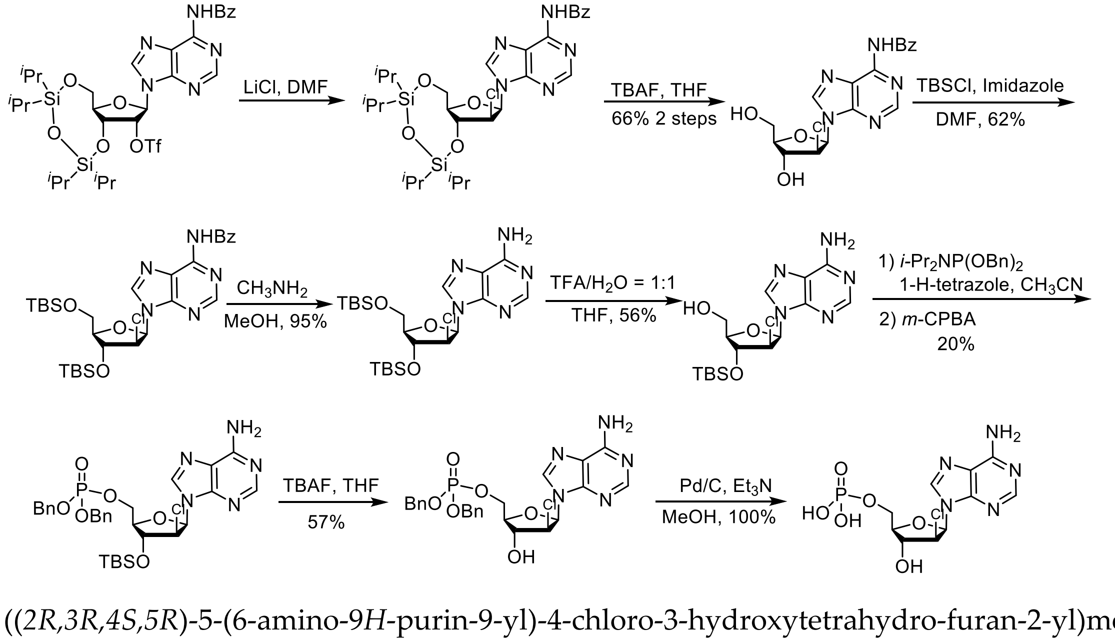

((*2R,3R,4S,5R*)-5-(6-amino-9*H*-purin-9-yl)-4-chloro-3-hydroxytetrahydro-furan-2-yl)methyl dihydrogen phosphate was prepared following the same procedure as the synthesis of

((*2R,3R,4R,5R*)-5-(6-amino-*9H*-purin-9-yl)-4-chloro-3-hydroxytetra-hydrofuran-2-yl)methyl dihydrogen phosphate starting from (*6aR,8R,9R,9aR*)-8-(6-benzamido-9*H*-purin-9-yl)-2,2,4,4-tetraisopropyltetrahydro-6*H*-furo[3,2-*f*][1,3,5,2,4]trioxadisilocin-9-yl trifluoromethanesulfonate.

(*6aR,8R,9R,9aR*)-8-(6-benzamido-9*H*-purin-9-yl)-2,2,4,4-tetraisopropyl-tetrahydro-6*H*-furo[3,2-*f*][1,3,5,2,4]trioxadisilocin-9-yl trifluoromethane-sulfonate (900 mg, 74%, colorless oil). ^{1}H NMR (500 MHz, $CDCl_3$) δ 9.36 (br, 1H), 8.66 (s, 1H), 8.19 (s, 1H), 7.98 (d, *J* = 7.7 Hz, 2H), 7.55 (t, *J* = 7.4 Hz, 1H), 7.46 (t, *J* = 7.7 Hz, 2H), 6.19 (s, 1H), 5.77 (d, *J* = 4.8 Hz, 1H), 5.24 (dd, *J* = 9.3, 4.8 Hz, 1H), 4.17 (dd, *J* = 13.5, 2.1 Hz, 1H), 4.09 (dt, *J* = 9.3, 2.4 Hz, 1H), 4.03 (dd, *J* = 13.4, 2.8 Hz, 1H), 1.27 – 0.70 (m, 28H); ^{13}C NMR (125 MHz, $CDCl_3$) δ 164.9, 152.8, 150.9, 150.0, 141.6, 133.5, 132.8, 128.8, 128.07, 123.7, 118.54 (q, *J* = 319.5 Hz), 88.1, 87.0, 81.6, 68.2, 59.5, 17.4, 17.3, 17.2, 16.8, 16.7, 16.6, 13.2, 12.9, 12.8, 12.7; ^{19}F NMR (471 MHz, $CDCl_3$) δ -74.9.

N-(9-((*2R,3S,4R,5R*)-3-chloro-4-hydroxy-5-(hydroxymethyl)tetrahydrofuran-2-yl)-9*H*-purin-6-yl)benzamide (670 mg, 66% for 2 steps, white solid). ^{1}H NMR (500 MHz, DMSO-d_6) δ 11.21 (s, 1H), 8.77 (s, 1H), 8.75 (s, 1H), 8.06 (d, *J* = 7.2 Hz, 2H), 7.65 (t, *J* = 7.2 Hz, 1H), 7.55 (t, *J* = 7.7 Hz, 2H), 6.66 (d, *J* = 6.4 Hz, 1H), 6.16 (d, *J* = 5.7 Hz, 1H), 5.27 (t, *J* = 5.3 Hz, 1H), 4.87 (dd, *J* = 8.0, 6.4 Hz, 1H), 4.53 (td, *J* = 7.8, 5.7 Hz, 1H), 3.89 (ddd, *J* = 7.4, 4.2, 2.8 Hz, 1H), 3.81 (ddd, *J* = 12.3, 5.3, 2.8 Hz, 1H), 3.74 (dt, *J* = 12.3, 4.9 Hz, 1H); ^{13}C NMR (125 MHz, DMSO) δ 165.6, 152.1, 151.8, 150.4, 142.8, 133.3, 132.5, 128.5, 125.1, 83.7, 82.7, 73.9, 63.6, 59.8.

N-(9-((*2R,3S,4R,5R*)-4-((*tert*-butyldimethylsilyl)oxy)-5-(((*tert*-butyldimethylsilyl)oxy)methyl)-3-chlorotetrahydrofuran-2-yl)-9*H*-purin-6-yl)benzamide (600 mg, 62%, white solid). ^{1}H NMR (500 MHz, $CDCl_3$) δ 9.66 (s, 1H), 8.70 (s, 1H), 8.33 (s, 1H), 7.96 (d, *J* = 7.2 Hz, 2H), 7.46 (t, *J* = 7.6 Hz, 1H), 7.38 (t, *J* = 7.7 Hz, 2H), 6.59 (d, *J* = 5.4 Hz, 1H), 4.58 (t, *J* = 4.9 Hz, 1H), 4.46 (t, *J* = 5.1 Hz, 1H), 3.93 (dd, *J* = 8.3, 4.5 Hz, 2H), 3.90 – 3.80 (m, 1H), 0.88 (s, 9H), 0.87 (s, 9H), 0.12 (s, 3H), 0.10 (s, 3H), 0.07 (s, 6H); ^{13}C NMR (125 MHz, $CDCl_3$) δ 164.9, 152.4, 151.2, 149.5, 141.5, 133.6, 132.4, 128.5, 127.8, 122.6, 84.9, 83.6, 76.7, 63.7, 61.5, 25.8, 25.5, 18.3, 17.7, -4.5, -5.0, -5.51, -5.52.

9-((*2R,3S,4R,5R*)-4-((*tert*-butyldimethylsilyl)oxy)-5-(((*tert*-butyldimethylsilyl)oxy)methyl)-3-chlorotetrahydrofuran-2-yl)-9*H*-purin-6-amine (500 mg, 95%, white solid). ^{1}H NMR (500 MHz, $CDCl_3$) δ 8.29 (s, 1H), 8.18 (s, 1H), 6.71 (s, 2H), 6.55 (d, *J* = 5.4 Hz, 1H), 4.59 (t, *J* = 5.3 Hz, 1H), 4.46 (t, *J* = 5.3 Hz, 1H), 3.94 (dd, *J* = 10.7, 4.1 Hz, 1H), 3.91 (dt, *J* = 5.6, 3.7 Hz, 1H), 3.86 (dd, *J* = 10.7, 3.2

Hz, 1H), 0.90 (s, 9H), 0.87 (s, 9H), 0.12 (s, 3H), 0.10 (s, 3H), 0.08 (s, 3H); ^{13}C NMR (125 MHz, $CDCl_3$) δ 155.8, 152.9, 149.4, 139.0, 119.1, 84.6, 83.5, 76.8, 63.9, 61.6, 25.9, 25.6, 18.4, 17.8, -4.4, -5.0, -5.4, -5.5.

((*2R,3R,4S,5R*)-5-(6-amino-9*H*-purin-9-yl)-3-((*tert*-butyldimethylsilyl)oxy)-4-chlorotetrahydrofuran-2-yl)methanol (230 mg, 56%, white solid). ^{1}H NMR (500 MHz, $CDCl_3$) δ 8.35 (s, 1H), 7.93 (s, 1H), 6.37 (d, *J* = 6.7 Hz, 1H), 5.76 (s, 2H), 4.94 (t, *J* = 7.3 Hz, 1H), 4.69 (s, 1H), 4.55 (t, *J* = 6.9 Hz, 1H), 4.09 (dd, *J* = 12.7, 2.3 Hz, 1H), 3.99 (dt, *J* = 7.4, 2.6 Hz, 1H), 3.88 (d, *J* = 12.6 Hz, 1H), 0.92 (s, 9H), 0.19 (s, 3H), 0.17 (s, 3H).

((2R,3R,4S,5R)-5-(6-amino-9H-purin-9-yl)-3-((tert-butyldimethylsilyl)oxy)-4-chlorotetrahydrofuran-2-yl)methyl dibenzyl phosphate (60 mg, 20%, white solid). ^{1}H NMR (500 MHz, $CDCl_3$) δ 8.34 (s, 1H), 8.15 (s, 1H), 7.39 – 7.28 (m, 10H), 6.54 (d, *J* = 5.0 Hz, 1H), 6.26 (br, 2H), 5.14 – 4.99 (m, 4H), 4.56 (t, *J* = 4.3 Hz, 1H), 4.46 (t, *J* = 4.5 Hz, 1H), 4.36 – 4.22 (m, 2H), 4.08 (q, *J* = 5.0 Hz, 1H), 0.90 (s, 9H), 0.14 (s, 3H), 0.10 (s, 3H); ^{31}P NMR (200 MHz, $CDCl_3$) δ -1.1.

((*2R,3R,4S,5R*)-5-(6-amino-9*H*-purin-9-yl)-4-chloro-3-hydroxytetrahydro-furan-2-yl)methyl dibenzyl phosphate (30 mg, 57%, white solid). ^{1}H NMR (500 MHz, MeOD) δ 8.18 (s, 2H), 7.33 – 7.25 (m, 10H), 6.53 (d, *J* = 5.9 Hz, 1H), 5.04 (dd, *J* = 8.6, 4.7 Hz, 4H), 4.68 (t, *J* = 6.0 Hz, 1H), 4.60 (t, *J* = 6.3 Hz, 1H), 4.46 (dt, *J* = 11.5, 6.5 Hz, 1H), 4.35 (ddd, *J* = 11.4, 6.7, 3.0 Hz, 1H), 4.13 (td, *J* = 6.3, 3.0 Hz, 1H); ^{13}C NMR (125 MHz, MeOD) δ 157.3, 154.0, 150.3, 141.2, 137.0 (dd, J_{C-P} = 6.4, 2.0 Hz), 129.7, 129.6, 129.1 (d, J_{C-P} = 2.0 Hz), 120.0, 85.6, 83.4 (d, J_{C-P} = 7.3 Hz), 77.2, 71.0 (dd, J_{C-P} = 7.6, 5.8 Hz), 68.0 (d, J_{C-P} = 5.7 Hz), 64.5; ^{31}P NMR (200 MHz, MeOD) δ -1.4.

((*2R,3R,4S,5R*)-5-(6-amino-9*H*-purin-9-yl)-4-chloro-3-hydroxytetrahydro-furan-2-yl)methyl dihydrogen phosphate(20 mg, 100%, white solid).^{1}H NMR (500 MHz, D_2O) δ 8.52 (s, 1H), 8.15 (s, 1H), 6.53 (d, *J* = 6.3 Hz, 1H), 4.63 (t, *J* = 7.5 Hz, 1H), 4.37 – 4.09 (m, 3H); ^{13}C NMR (125 MHz, D_2O) δ 155.4, 152.5, 148.5, 140.4, 118.0, 83.0, 82.0 (d, J_{C-P} = 8.2 Hz), 73.9, 62.3, 62.15 (d, J_{C-P} = 4.1 Hz); ^{31}P NMR (200 MHz, D_2O) δ 1.8.

Preparation of Compound 3 and $^2H_2$-3

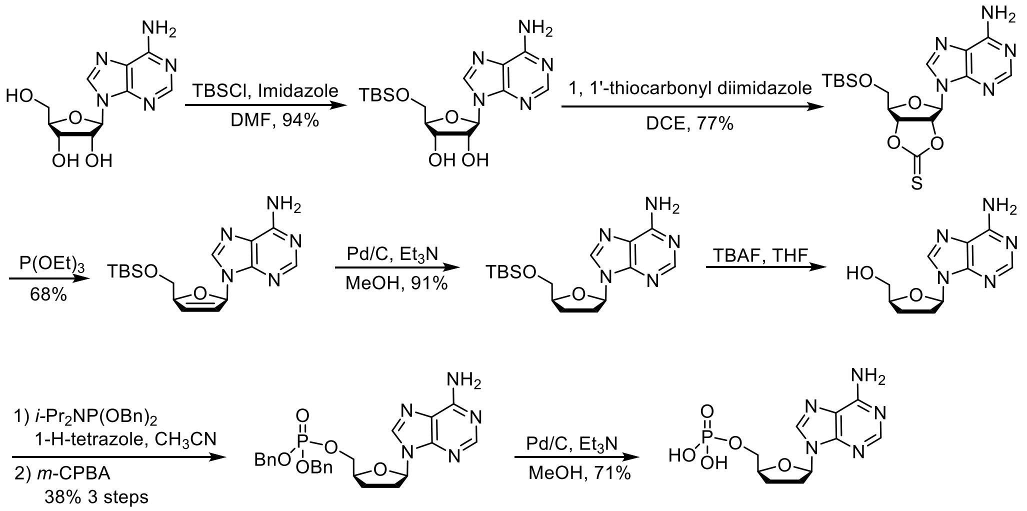

To a solution of adenosine (10 g, 37.4 mmol, 1.0 equiv.) in dimethylformamide (DMF) (300 mL) was added Imidazole (6.8 g, 45 mmol, 1.2 equiv.) and *tert*-butyldimethylsilyl chloride (5.3 g, 78.5 mmol, 2.1 equiv.) at 0 °C. After addition, the reaction mixture was warmed to room temperature and stirred for 24 h. Then the reaction mixture was concentrated under reduced pressure. The residue was purified by a flash column chromatography on silica gel (2% to 5% MeOH/DCM (v/v)) to give *(2R,3R,4S,5R)*-2-(6-amino-9*H*-purin-9-yl)-5-(((*tert*-butyldimethylsilyl)oxy)methyl)-tetrahydrofuran-3,4-diol as a colorless oil (13.2 g, 94%). ^{1}H NMR (500 MHz, MeOD) δ 8.39 (s, 1H), 8.20 (s, 1H), 7.05 (d, *J* = 1.1 Hz, 2H), 6.06 (d, *J* = 4.2 Hz, 1H), 4.56 (t, *J* = 4.6 Hz, 1H), 4.37 (t, *J* = 5.0 Hz, 1H), 4.17 – 4.11 (m, 1H), 4.01 (dd, *J* = 11.6, 3.0 Hz, 1H), 3.88 (dd, *J* = 11.6, 3.0 Hz, 1H), 0.92 (s, 9H), 0.11 (s, 3H), 0.11 (s, 3H); ^{13}C NMR (125 MHz, MeOD) δ 157.3, 153.9, 150.4, 140.7, 136.3, 122.6, 120.3, 90.0, 86.3, 76.4, 71.2, 63.8, 26.5, 19.3, -5.3, -5.4.

To a solution of *(2R,3R,4S,5R)*-2-(6-amino-9*H*-purin-9-yl)-5-(((*tert*-butyldimethylsilyl)oxy)-methyl)tetrahydrofuran-3,4-diol (12.2 g, 32 mmol, 1.0 equiv.) in dichloroethane (DCE) (300 mL) was added 1, 1'-thiocarbonyl diimidazole (8.5 g, 48 mmol, 1.5 equiv.) at room temperature. Then the reaction mixture was heated to reflux and stirred for 14 h. The reaction mixture was concentrated under reduced pressure. The residue was purified by a flash column chromatography on silica gel (2% to 4% MeOH/DCM (v/v)) to give (*3aR,4R,6R,6aR*)-4-(6-amino-9*H*-purin-9-yl)-6-(((*tert*-butyldimethylsilyl)oxy)methyl) tetrahydrofuro[3,4-*d*][1,3]dioxole-2-thione as a yellow solid (10.5 g, 77%). ^{1}H NMR (500 MHz, $CDCl_3$) δ 8.30 (s, 1H), 7.95 (s, 1H), 6.36

(dd, *J* = 7.3, 1.6 Hz, 1H), 6.30 (d, *J* = 1.7 Hz, 1H), 6.13 (br, 2H), 5.79 (dd, *J* = 7.2, 2.5 Hz, 1H), 4.60 (td, *J* = 5.9, 2.6 Hz, 1H), 3.69 (d, *J* = 6.0 Hz, 2H), 0.81 (s, 9H), -0.04 (s, 3H), -0.05 (s, 3H); ^{13}C NMR (125 MHz, $CDCl_3$) δ 189.3, 155.5, 152.7, 148.8, 140.0, 120.0, 90.0, 87.33, 87.30, 86.2, 62.0, 25.7, 18.1, -5.58, -5.60.

(*3aR,4R,6R,6aR*)-4-(6-amino-9*H*-purin-9-yl)-6-(((*tert*-butyldimethylsilyl)oxy)methyl) tetrahydrofuro[3,4-*d*][1,3]dioxole-2-thione (3.06 g, 7.2 mmol, 1.0 equiv.) was dissolved in triethyl phosphite (50 mL), then the reaction mixture was heated to reflux and stirred for 4 h. The reaction mixture was concentrated under reduced pressure. The residue was purified by a flash column chromatography on silica gel (2% to 4% MeOH/DCM (v/v)) to give 9-((*2R,5S*)-5-(((*tert*-butyldimethylsilyl)oxy)methyl)-2,5-dihydrofuran-2-yl)-9*H*-purin-6-amine as a yellow oil (1.7 g, 68%). ^{1}H NMR (500 MHz, MeOD) δ 8.27 (s, 1H), 8.22 (s, 1H), 7.04 (dt, *J* = 3.1, 1.6 Hz, 1H), 6.48 (dt, *J* = 6.1, 1.9 Hz, 1H), 6.20 – 6.12 (m, 1H), 5.03 – 4.97 (m, 1H), 4.01 – 3.78 (m, 2H), 0.87 (s, 9H), 0.02 (s, 6H); ^{13}C NMR (125 MHz, MeOD) δ 157.3, 153.9, 150.3, 141.0, 135.7, 126.6, 120.0, 89.8, 89.6, 66.0, 26.5, 19.4, -5.3.

To a solution of 9-((*2R,5S*)-5-(((*tert*-butyldimethylsilyl)oxy)methyl)-2,5-dihydrofuran-2-yl)-9*H*-purin-6-amine (1.70 g, 4.89 mmol) in 5% solution of triethyl amine in methanol (50 mL) was added Pd/C (170 mg, 10%) at room temperature. Then the reaction mixture was stirred for 46 h under H_2 atmosphere. The reaction mixture was filtered by celite and concentrated under reduced pressure. 9-((*2R,5S*)-5-(((*tert*-butyldimethylsilyl)oxy)methyl)tetrahydrofuran-2-yl)-9*H*-purin-6-amine was obtained as a white solid (1.39 g, 91%). ^{1}H NMR (500 MHz, MeOD) δ 8.46 (s, 1H), 8.19 (s, 1H), 6.29 (dd, *J* = 6.7, 2.4 Hz, 1H), 4.30 – 4.19 (m, 1H), 3.98 (dd, *J* = 11.4, 3.0 Hz, 1H), 3.76 (dd, *J* = 11.4, 3.3 Hz, 1H), 2.61 – 2.46 (m, 1H), 2.46 – 2.36 (m, 1H), 2.28 – 2.09 (m, 1H), 2.09 – 1.95 (m, 1H), 0.88 (s, 9H), 0.06 (s, 3H), 0.05 (s, 3H); ^{13}C NMR (125 MHz, MeOD) δ 157.2, 153.6, 149.8, 140.6, 120.3, 86.8, 83.8, 65.1, 34.1, 26.5, 25.7, 19.3, -5.29, -5.31.[2]

To a solution of 9-((*2R,5S*)-5-(((tert-butyldimethylsilyl)oxy)methyl)tetrahydrofuran-2-yl)-9H-purin-6-amine (1.13 g, 3.2 mmol) in THF (64 mL) was added TBAF (1.0 M in THF, 3.9 mL, 3.9 mmol, 1.2 equiv.) at room temperature. Then the reaction mixture was stirred for 2 h. The reaction mixture was concentrated under reduced pressure. The residue was purified by a flash column

chromatography on silica gel (2% to 4% MeOH/DCM (v/v)) to give ((2*S*,5*R*)-5-(6-amino-9*H*-purin-9-yl)tetrahydrofuran-2-yl)methanol as a yellow oil (800 mg).

((2*S*,5*R*)-5-(6-amino-9H-purin-9-yl)tetrahydrofuran-2-yl)methanol (800 mg) was dried by azeotrope with anhydrous pyridine three times and then dissolved in anhydrous MeCN (34 mL) and cooled to 0 °C. The dibenzyl *N*,*N*-diisopropylphosphoramidite (1.35 g, 3.9 mmol, 1.15 equiv.) was added followed by 5-(ethylthio)-1*H*-tetrazole (3% in MeCN, 13.7 g, 5.8 mmol, 1.73 equiv.) and the reaction mixture was allowed to warm to room temperature. The reaction mixture was stirred at room temperature for 22 h. *m*-CPBA (70% wt., 961 mg, 3.9 mmol, 1.15 equiv.) was added at 0 °C and the reaction mixture was allowed to warm to room temperature. The reaction mixture was stirred at room temperature for additional 2 h. Then the reaction mixture was diluted by ethyl acetate and washed with 1 M aq. $Na_2S_2O_3$, sat. aq. $NaHCO_3$ and brine, then dried over $MgSO_4$ and concentrated under reduced pressure. The residue was purified by a flash column chromatography on silica gel (1% to 5% MeOH/DCM (v/v)) to give ((2*S*,5*R*)-5-(6-amino-9*H*-purin-9-yl)tetrahydrofuran-2-yl)methyl dibenzyl phosphate as a colorless oil (600 mg, 38% for 3 steps). ^{1}H NMR (500 MHz, DMSO-d_6) δ 8.28 (s, 1H), 8.13 (s, 1H), 7.56 – 7.11 (m, 10H), 6.26 (dd, *J* = 6.8, 4.2 Hz, 1H), 5.03 – 4.88 (m, 4H), 4.35 – 4.22 (m, 1H), 4.22 – 4.13 (m, 1H), 4.13 – 4.05 (m, 1H), 2.50 – 2.38 (m, 2H), 2.10 (q, *J* = 7.5 Hz, 2H); ^{13}C NMR (125 MHz, DMSO-d_6) δ 156.1, 152.6, 149., 139.0, 136.0 (d, J_{C-P} = 6.8 Hz), 128.5 (d, J_{C-P} = 2.1 Hz), 128.4, 127.8, 119.1, 84.3, 78.8 (d, J_{C-P} = 7.7 Hz), 68.6 (d, J_{C-P} = 4.2 Hz), 68.5 (d, J_{C-P} = 5.1 Hz), 30.8, 25.9; ^{31}P NMR (200 MHz, DMSO) δ -1.0.

To a solution of ((2*S*,5*R*)-5-(6-amino-9H-purin-9-yl)tetrahydrofuran-2-yl)methyl dibenzyl phosphate (175 mg, 0.35 mmol) in 5% solution of triethyl amine in methanol (15 mL) was added Pd/C (35 mg, 20%) at room temperature. Then the reaction mixture was stirred for 24 h under H_2 atmosphere. The reaction mixture was filtered by celite and concentrated under reduced pressure. ((2*S*,5*R*)-5-(6-amino-9*H*-purin-9-yl)tetrahydrofuran-2-yl)methyl dihydrogen phosphate was obtained as a white solid (110 mg, 100%). ^{1}H NMR (500 MHz, D_2O) δ 8.26 (s, 1H), 7.87 (s, 1H), 6.08 (dd, *J* = 9.6, 2.8 Hz, 1H), 4.40 – 4.28 (m, 1H), 3.95 (ddd, *J* = 11.5, 5.2, 3.3 Hz, 1H), 3.80 (dt, *J* = 11.3, 5.5 Hz, 1H), 2.56 – 2.42 (m, 1H), 2.32 – 2.21 (m, 1H), 2.20 – 2.06 (m, 1H), 2.04 – 1.88 (m, 1H); ^{13}C NMR (125 MHz, D_2O) δ 154.8, 151.8, 147.5, 139.5, 118.0, 84.7, 81.4 (d, J_{C-P} = 8.2 Hz), 65.4 (d, J_{C-P} = 5.0 Hz), 31.8, 25.2; ^{31}P NMR (200 MHz, D_2O) δ 3.2.

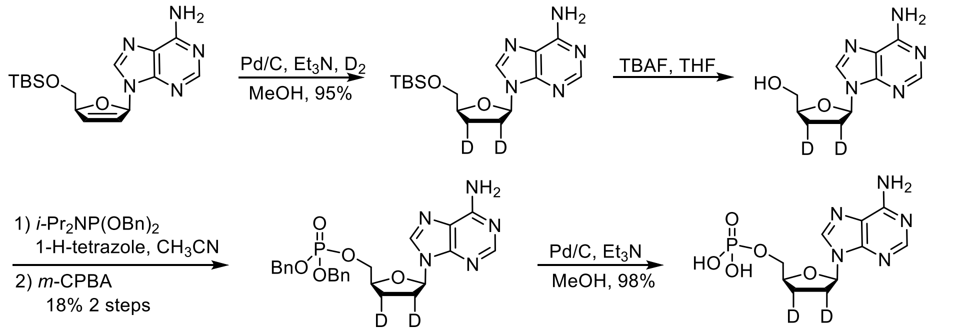

To a solution of 9-((*2R,5S*)-5-(((*tert*-butyldimethylsilyl)oxy)methyl)-2,5-dihydrofuran-2-yl)-9*H*-purin-6-amine (2.38 g, 6.8 mmol) in 5% solution of triethyl amine in methanol-d_1 (25 mL) was added Pd/C (238 mg, 10%) at room temperature. Then the reaction mixture was stirred for 26 h under D_2 atmosphere. The reaction mixture was filtered by celite and concentrated under reduced pressure. 9-((*2R,3R,4S,5S*)-5-(((*tert*-butyldimethylsilyl)oxy)methyl)tetrahydrofuran-2-yl-3,4-d_2)-9*H*-purin-6-amine was obtained as a white solid (2.28 g, 95%). ^{1}H NMR (500 MHz, MeOD) δ 8.48 (s, 1H), 8.19 (s, 1H), 6.30 (d, *J* = 2.4 Hz, 1H), 4.25 (dt, *J* = 9.2, 3.2 Hz, 1H), 4.00 (dd, *J* = 11.5, 3.0 Hz, 1H), 3.78 (dd, *J* = 11.5, 3.4 Hz, 1H), 2.41 (dd, *J* = 8.0, 2.4 Hz, 1H), 2.17 (t, *J* = 8.5 Hz, 1H), 0.91 (s, 9H), 0.08 (s, 3H), 0.07 (s, 3H); ^{13}C NMR (125 MHz, MeOD) δ 157.2, 153.7, 149.8, 140.7, 120.4, 86.8, 83.8, 65.2, δ 33.7 (t, $J_{C\text{-}D}$ = 24.6 Hz), 26.4, 25.4 (t, $J_{C\text{-}D}$ = 23.1 Hz), 19.3, -5.3.

To a solution of 9-((*2R,3R,4S,5S*)-5-(((*tert*-butyldimethylsilyl)oxy)methyl)tetrahydrofuran-2-yl-3,4-d_2)-9*H*-purin-6-amine (2.28 g, 6.5 mmol) in THF (65 mL) was added TBAF (1.0 M in THF, 6.5 mL, 6.5 mmol, 1.0 equiv.) at room temperature. Then the reaction mixture was stirred for 2 h. The reaction mixture was concentrated under reduced pressure. The residue was purified by a flash column chromatography on silica gel (2% to 4% MeOH/DCM (v/v)) to give ((*2S,3S,4R,5R*)-5-(6-amino-9*H*-purin-9-yl)tetrahydrofuran-2-yl-3,4-d_2)methanol as a yellow oil (1.08 g).

((*2S,3S,4R,5R*)-5-(6-amino-9*H*-purin-9-yl)tetrahydrofuran-2-yl-3,4-d_2)methanol (1.08 g, 4.55 mmol) was dried by azeotrope with anhydrous pyridine three times and then dissolved in anhydrous MeCN (40 mL) and cooled to 0 °C. The dibenzyl *N,N*-diisopropylphosphoramidite (1.81 g, 5.24 mmol, 1.15 equiv.) was added followed by 5-(ethylthio)-1*H*-tetrazole (3% in MeCN, 18.4 g, 7.87 mmol, 1.73 equiv.) and the reaction mixture was allowed to warm to room temperature. The reaction mixture was stirred at room temperature for 7 h. *m*-CPBA (70% wt., 1.3 g, 5.24 mmol, 1.15 equiv.) was added at 0 °C and the reaction mixture was allowed to warm to

room temperature. The reaction mixture was stirred at room temperature for additional 1 h. Then the reaction mixture was diluted by ethyl acetate and washed with 1 M aq. $Na_2S_2O_3$, sat. aq. $NaHCO_3$ and brine, dried over $MgSO_4$ and concentrated under reduced pressure. The residue was purified by a flash column chromatography on silica gel (1% to 5% MeOH/DCM (v/v)) to give ((*2S*,*3S*,*4R*,*5R*)-5-(6-amino-9*H*-purin-9-yl)tetrahydrofuran-2-yl-3,4-*d*$_2$)methyl dibenzyl phosphate as a colorless oil (600 mg, 18% for 2 steps). ^{1}H NMR (500 MHz, MeOD) δ 8.22 (s, 1H), 8.16 (s, 1H), 7.31 – 7.18 (m, 10H), 6.25 (d, *J* = 3.4 Hz, 1H), 5.00 – 4.90 (m, 4H), 4.37 – 4.27 (m, 1H), 4.20 (ddd, *J* = 11.2, 6.1, 3.1 Hz, 1H), 4.12 (ddd, *J* = 11.3, 6.4, 5.0 Hz, 1H), 2.49 (dd, *J* = 8.2, 3.4 Hz, 1H), 2.07 (t, *J* = 8.4 Hz, 1H); ^{13}C NMR (125 MHz, MeOD) δ 157.2, 153.7, 150.1, 140.5, 137.0 (dd, J_{C-P} = 6.2, 2.6 Hz), 129.7, 129.6, 129.1 (d, J_{C-P} = 2.6 Hz), 120.4, 86.6, 80.8 (d, J_{C-P} = 7.9 Hz), 70.8 (dd, J_{C-P} = 5.8, 3.1 Hz), 69.8 (d, J_{C-P} = 5.9 Hz), 32.3 (t, J_{C-D} = 22.5 Hz), 26.3 (t, J_{C-D} = 22.5 Hz); ^{31}P NMR (200 MHz, MeOD) δ -1.3.

To a solution of ((*2S*,*3S*,*4R*,*5R*)-5-(6-amino-9*H*-purin-9-yl)tetrahydrofuran-2-yl-3,4-*d*$_2$)methyl dibenzyl phosphate (320 mg, 0.64 mmol) in 5% solution of triethyl amine in methanol (35 mL) was added Pd/C (64 mg, 20%) at room temperature. Then the reaction mixture was stirred for 24 h under H_2 atmosphere. The reaction mixture was filtered by celite and concentrated under reduced pressure. ((*2S*,*3S*,*4R*,*5R*)-5-(6-amino-9*H*-purin-9-yl)tetrahydrofuran-2-yl-3,4-*d*$_2$)methyl dihydrogen phosphate was obtained as a white solid (200 mg, 98%). ^{1}H NMR (500 MHz, D_2O) δ 8.28 (s, 1H), 7.89 (s, 1H), 6.10 (d, *J* = 3.1 Hz, 1H), 4.46 – 4.25 (m, 1H), 4.06 – 3.91 (m, 1H), 3.81 (dt, *J* = 11.3, 5.5 Hz, 1H), 2.28 (dd, *J* = 7.9, 3.1 Hz, 1H), 1.96 (t, *J* = 8.4 Hz, 1H); ^{13}C NMR (125 MHz, D_2O) δ 154.8, 151.9, 147.6, 139.6, 118.1, 84.7, 81.4 (d, J_{C-P} = 8.1 Hz), 65.5 (d, J_{C-P} = 4.7 Hz), 31.5 (t, J_{C-D} = 19.1 Hz), 24.9 (t, J_{C-D} = 24.8 Hz); ^{31}P NMR (200 MHz, D_2O) δ 3.2.

Stopped-flow absorption spectroscopy of AdeV

Stopped-flow absorption spectroscopy (SF-Abs) experiments were performed with the Applied Photophysics SX20 stopped-flow spectrometer operating in an MBraun UNilab glove box circulated with nitrogen gas and maintained O_2 levels < 5 ppm. All solutions were prepared under anaerobic conditions in the glove box. An O_2-saturated buffer solution (20 mM HEPES, pH 8.0) was rapidly mixed in equal parts with an O_2-free protein solution containing AdeV (0.5 mM), Fe^{2+} (0.45 mM), 2OG (4.5 mM), NaCl (45 mM) and varying amount of 2′-dAMP (4.5 mM to 120 mM).

The absorbance scans were collected from 300 – 700 nm with a photo-diode array detect at 4 °C. The optical data was processed using the KaleidaGraph software (Synergy Software, Reading, PA).

Freeze-quench Mössbauer Sample Preparation

The rapid freeze-quench (RFQ) Mössbauer experiments were carried out using a KinTek quench-flow instrument (KinTek Co., Snow Shoe, PA). An oxygen-saturated buffer solution (20 mM HEPES, pH 8.0, ~ 0.9 mM O_2) was rapidly mixed with an equal volume oxygen-free solution containing AdeV (1.2 mM), Fe^{2+} (1.1 mM), 2OG (9.6 mM), NaCl (25 mM) and 2′-dAMP (6.7 mM) at 4 °C. The reaction was terminated by injection into a solution of liquid ethane maintained at 90 K at selected time points. Each sample was vacuum pumped to remove the residual liquid ethane. The dry, frozen powder was packed into freeze-quench Mössbauer cups for analysis. The reaction time of a freeze-quenched sample is the summation of the ageing time and the quench time. The ageing time corresponds to the time for the reaction mixture to traverse the ageing line into the mixing chamber. The quench time corresponded to the time required after injection into the cryosolvent for the reaction mixture to be cooled sufficiently to prevent further reaction and was estimated to be ~5 ms.

Mössbauer Analysis

Mössbauer spectra were recorded with home-built spectrometers using Janis Research Super-Varitemp dewars with a temperature range of 1.5 – 200 K and applied magnetic fields upwards to 8.0 T. The Mössbauer spectral simulations were performed using the WMOSS software package (SEE Co., Edina, MN) and the SpinCount software. Isomer shifts reported are quoted relative to Fe metal measured at 298 K. All subsequent Mössbauer figures were prepared using the SpinCount software.[3] In the Mössbauer analysis, the following spin Hamiltonian was used:

$$\hat{H} = \beta\vec{S}\cdot\tilde{g}\cdot\vec{B} + \vec{S}\cdot\tilde{D}\cdot\vec{S} + \vec{S}\cdot\tilde{A}\cdot\vec{I} - \beta_N g_N\vec{B}\cdot\vec{I} + \vec{I}\cdot\tilde{Q}\cdot\tilde{V}\cdot\vec{I} \quad \text{(Eq. 1)}$$

where the first term represents the electronic Zeeman interactions, the second term represents the zero-field splitting, the third term represents the electron-nuclear hyperfine interactions, the fourth term represents the nuclear Zeeman interactions, and the last term represents the nuclear quadrupole interactions. $\vec{S}$ is the electronic spin operator, $\vec{B}$ is the external magnetic field vector, $\vec{I}$ is the ^{57}Fe nuclear spin operator, $\tilde{D}$ is the zero-field splitting tensor, $\tilde{A}$ is

the ^{57}Fe nuclear hyperfine tensor, $\tilde{Q}$ is the ^{57}Fe nuclear quadrupole tensor, and $\tilde{V}$ is the electric field gradient tensor. β, β_N, and g_N are the electronic Bohr magneton, nuclear Bohr magneton and ^{57}Fe nuclear g value.

Specifically, for the zero-field splitting term with S = 2:

$$\vec{S}\cdot\tilde{D}\cdot\vec{S} = D\left[(S_z{}^2 - S(S+1)/3) + \frac{E}{D}\left(S_x{}^2 - S_y{}^2\right)\right] \qquad \text{(Eq. 2)}$$

where D is the axial zero field splitting parameter, and E is the rhombic zero field splitting parameter. For ^{57}Fe, the electric quadrupole interaction is described by the following equation:

$$\vec{I}\cdot\tilde{Q}\cdot\tilde{V}\cdot\vec{I} = \frac{eQV_{zz}}{12}[3I_z{}^2 - I(I+1) + \eta\left(I_x{}^2 - I_y{}^2\right)],\ \eta = \frac{V_{xx}-V_{yy}}{V_{zz}} \qquad \text{(Eq. 3)}$$

Rapid Chemical Quench Sample Preparation

Chemical quench experiments were carried out by using the samples generated by the freeze-quench Mössbauer analysis so the results of product analysis can be directly correlated with the Mössbauer results. Specifically, the frozen protein mixture from the Mössbauer samples was quenched by injecting into a solution of acetonitrile and acetic acid (80:20 v/v) in a 1:4 ratio at 25 °C at various time points. The final concentrations of the reactants in the solution were estimated to be 0.2 mM Fe(II)-AdeV, 0.64 mM 2OG, 5 mM NaCl, and 1.34 mM 2′-dAMP, respectively. The resulting solutions were centrifuged at 10,000 x *g* prior to analysis by liquid chromatography mass-spectrometry (LC-MS). The intensity ratio of the substrate and the product was used to quantify the amount of the product formed at different time points.

Computational Methods

System Preparation

The initial structure of Adev was obtained from the crystal structure (PDB ID: 7v57) [4] with 2OG in an inline fashion (C1 carboxylate of 2OG bound trans to H192), Cl^- ion, and a Fe atom from the RCSB protein data bank. The obtained structure is remodeled by modifying the water molecule attached trans to the H250 to dioxygen to represent the inline Fe(III)-superoxo intermediate. The inline Fe(III)-superoxo system thus obtained has Fe with bidentate coordination to 2OG and monodentate coordination to two histidines, H192, H250, and a chlorine iron Cl^-. Similarly, for the ferryl system, the water molecule trans to H250 was modified to oxygen, and the 2OG was modified to succinate with

monodentate coordination. The 2′-dAMP substrate is obtained from the crystal structure of the H194A variant of AdeV (PDB ID: H194A) by overlaying with the 7V57 structure. The parameters for 2′-dAMP and succinate or 2OG were obtained using the generalized Amber force field (GAFF)[5] implemented in the antechamber suite in AmberTools20.[6] The metal center parameters at high spin (HS, S=2) Fe(III)-superoxo and Fe(IV)=O intermediate level, including bond and angle force constants, were obtained using the Metal Center Parameter builder (MCPB.py v3.0)[7] of AmberTools20, and the remaining protein residues were considered using Amber ff14SB force field.[8] The protein is solvated using TIP3P[9] water molecules extending up to 10 Å from the protein surface, and the Na+ counter ions were added to the system for neutralization using a leap module in Amber20.[6] For the offline system, the superoxo oxygen and the ferryl oxygen molecule were oriented trans to the H192 residue in Fe(III)-superoxo and ferryl system, respectively, using Chimera tools.[10] The same procedure followed for the inline systems was repeated for the offline Fe(III)-superoxo and ferryl systems.

Molecular Dynamics Simulations

The solvated systems thus obtained initially undergo minimizations using the steepest descent (5000 steps) and then the conjugate gradient (5000 steps) method. The first minimization involves slightly restraining the solute molecules to relax the solvent molecules, while the second minimization relaxes the whole system. The systems were then subjected to controlled heating under NVT ensemble from 0 to 300k using a Langevin thermostat[11,12] with a collision frequency of 1 ps for 250ps with mild restraint using the harmonic potential of 50 kcal mol^{-1} $Å^{2}$ on the solute molecules. The periodic boundary conditions were implemented in all simulations. Long-range electrostatic interactions were calculated with the particle mesh Ewald method[13,14] with a direct space and van der Walls cutoff of 10 Å. The SHAKE[15] algorithm was used to constrain the bonds involving hydrogens. Following heating, the system undergoes MD simulation for one ns to achieve a uniform density with a weak restraint on the solute molecules using

periodic boundary conditions. Subsequently, the systems were equilibrated for 3ns at 300K in an NPT ensemble without restraint. During the simulations, the pressure was maintained at 1 bar using the Berendsen barostat.[16] The production simulations were performed for 1 μs each with a timestep of 2 fs in an NPT ensemble with pressure set at 1 bar and constant pressure coupling of 2ps. The GPU version of the Amber20 was used for the production simulations.[6] The hydrogen bond analysis was carried out using the CPPTRAJ[17] module of Amber20. The principal component and dynamic cross-correlation analyses were performed on the backbone atoms on the equilibrated portion of the production simulation using the Bio3D[18] module in R programming language.

QM/MM Calculations

Snapshots are chosen from the equilibrated regions of the 1 μs MD trajectory for QM/MM calculations. Water molecules beyond 12 Å from the protein were truncated. QM/MM simulations were conducted using the ChemShell program:[19] DL_POLY[20] for the MM implementation and Turbomole[21] for the QM region. The polarizing effect of the protein environment on the QM region was accounted for using an electrostatic embedding scheme.[22] Hydrogen link atoms were used to cap the QM/MM boundaries using a charge shift model.[23] The Fe, Superoxo, Cl^-, 2OG, H192, H250 and 2′-dAMP substrate defined the QM region for the offline and inline Fe(III)-superoxo complexes. The Fe, O, Cl^-, Succinate, H192, H250, and 2′-dAMP substrates defined the QM regions (QM1) for the offline and inline ferryl systems. The flexible MM region includes the protein residues and water within 8 Å from the QM region, and the remaining residues and solvent beyond 8 Å were fixed. The Amber ff14SB force field was used for the MM region.[8] The geometry optimization and frequency calculations used the def2-SVP basis set with the DFT-B3LYP (QM(B1)/MM) method. The reaction path scans (PES) with specific reaction coordinates for the reaction under study were implemented on the optimized reaction complexes (RC) with a step size of 0.1 to obtain transition states (TS), intermediate (IM), and product complexes (PC). Without any constraints, the highest energy points along the PES are

optimized using the dimer method[24] implemented in the DL-FIND[25] optimizer. Frequency calculations were performed to confirm the minima and transition states. To further refine the energies, the single point (SP) energy calculations were performed on the optimized geometries using a large all-electron def2-TZVP[23] basis set (QM(B2)/MM). The zero-point energies from frequency calculations were added to the B2 energy to obtain zero-point corrected QM (B3)/MM energies. The results are discussed at the QM(B3)/MM level of energies. We further performed additional calculations to test the accuracy of the calculations using (i) an extended QM region with SCS residue Q201(QM2) in the QM region in our QM/MM calculations for the Off2-RC snapshot (Figure S35, Table S5), (ii) SP calculations with Grimme's D3 dispersion correction (QM(B3(D3)/MM))(Table S2, S3), and (iii) optimization with a higher basis set def2-TZVP on the stationary points obtained during reaction path calculations (Table S8). QM2 calculations gave comparable barriers, and the optimized stationary points are superimposable with the stationary points obtained from QM1 region calculations (Figure S35). Similarly, optimization with the B2 basis set also gave similar reaction barriers and optimized structures (Table S8).

The primary kinetic isotope effects were calculated using the zero-curvature tunneling (ZCT) method as implemented in ChemShell. Using DL-FIND,[25] Hessian calculations were performed in ChemShell. The KIEs were calculated on both deuterated and nondeuterated systems using transition state theory (TST).

Mössbauer Calculations

We implemented computational Mössbauer calculations to corroborate the experimental Mössbauer parameters. We used the ORCA program (version 6.0.1)[26,27] for calculating Mössbauer isomer shifts (δ) and quadrupole splitting (ΔE_Q) parameters. Using the optimized RCs (Off1-RC and In1-RC) obtained from QM/MM calculations, we included four water molecules surrounding the 2′-dAMP substrate in the QM region and

optimized it again using QM/MM calculations in Chemshell. For Mössbauer calculations, the reoptimized geometries are included in the QM region in ORCA with the MM point charges. We implemented the B3LYP functional with the CP(PPP) basis set for the Fe atom and def2-TZVP basis for the remaining atoms. Mössbauer isomer shift was determined from the electron density at the Fe nucleus, using the calibration parameters obtained at the B3LYP/TZVP/Fe: CP(PPP) level of theory reported elsewhere: $\delta_{calc} = \alpha(\varrho - C) + \beta$, where α = -0.366 mm s-1 au^3, β = 2.852 mm s-1, and C = 11810 au.[28] We have utilized several QM model with different numbers of water molecules with and without MM charges, and the results are tabulated in Table S23. The inclusion of water molecules surrounding the phosphate group of the substrate reduces the significant spin density on the phosphate group, which is most likely an artifact from the QM calculations (see Table S23).

Alternate Substrate Binding Position in AdeV.

A recently published crystal structure of Pyrimidine Nucleoside 2′-Hydroxylase presented a very interesting substrate binding pose of the thymidine.[29] The thymine group of the thymidine structure was demonstrated to have a pi-pi stacking interaction with the phenyl ring of the F339 residue. Interestingly, such a phenylalanine residue was also conserved in the Adav system. The new binding pose was positioned favorably for C-H activation at the C2′ position and the subsequent chlorination. Hence, we reoriented the 2′-dAMP substrate to maintain a pi-pi stacking interaction with the F269 residue and conducted MD simulation on the inline ferryl system with the alternate binding pose. However, the MD simulations predicted that such a binding pose was not feasible as the substrate lost the pi-pi interactions; consequently, the substrate moved away after 150ns of the MD trajectory. The bulkier adenine group of the 2′-dAMP substrates does not favor the alternate binding pose in the AdeV system. Hence, the current calculations suggest that the alternate substrate binding pose of 2′-dAMP is not feasible in AdeV.

Supplementary Figures

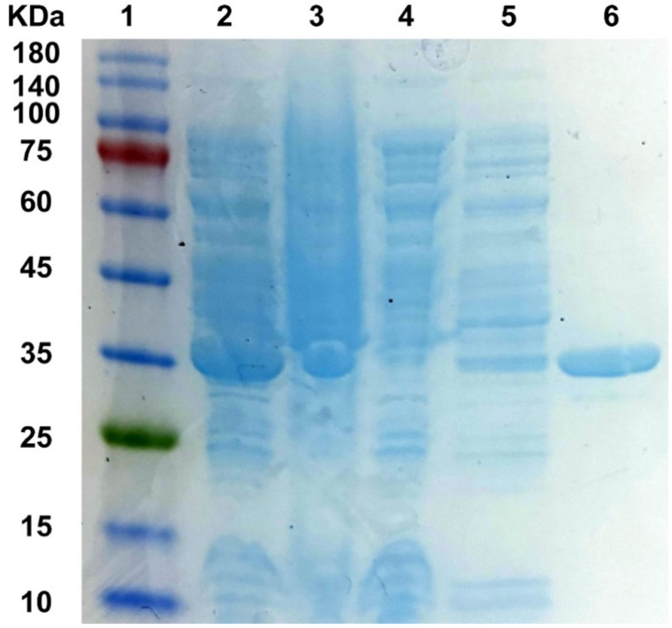

Figure S1. Comassie-stained SDS-PAGE (12%) of His6-tagged purification of AdeV. Lane **1**: protein ladder; Lane **2**: total cell extract; Lane **3**: soluble cell-free extract; Lane **4**: column flow-through; Lane **5**: column wash; Lane **6**: elution of isolated AdeV.

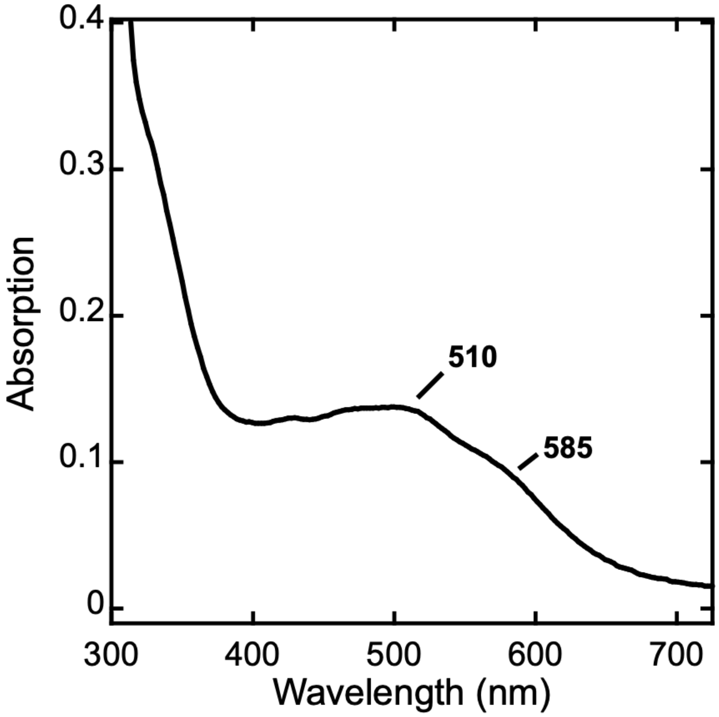

Figure S2. Optical spectrum of anaerobic AdeV solution incubated with Fe^{2+}, 2OG, Cl^-, and **1**. The concentrations used are: [AdeV] = 0.35 mM, [Fe^{2+}] = 0.32 mM, [2OG] = 3.2 mM, [Cl^-] = 63 mM, and [**1**] = 6.3 mM.

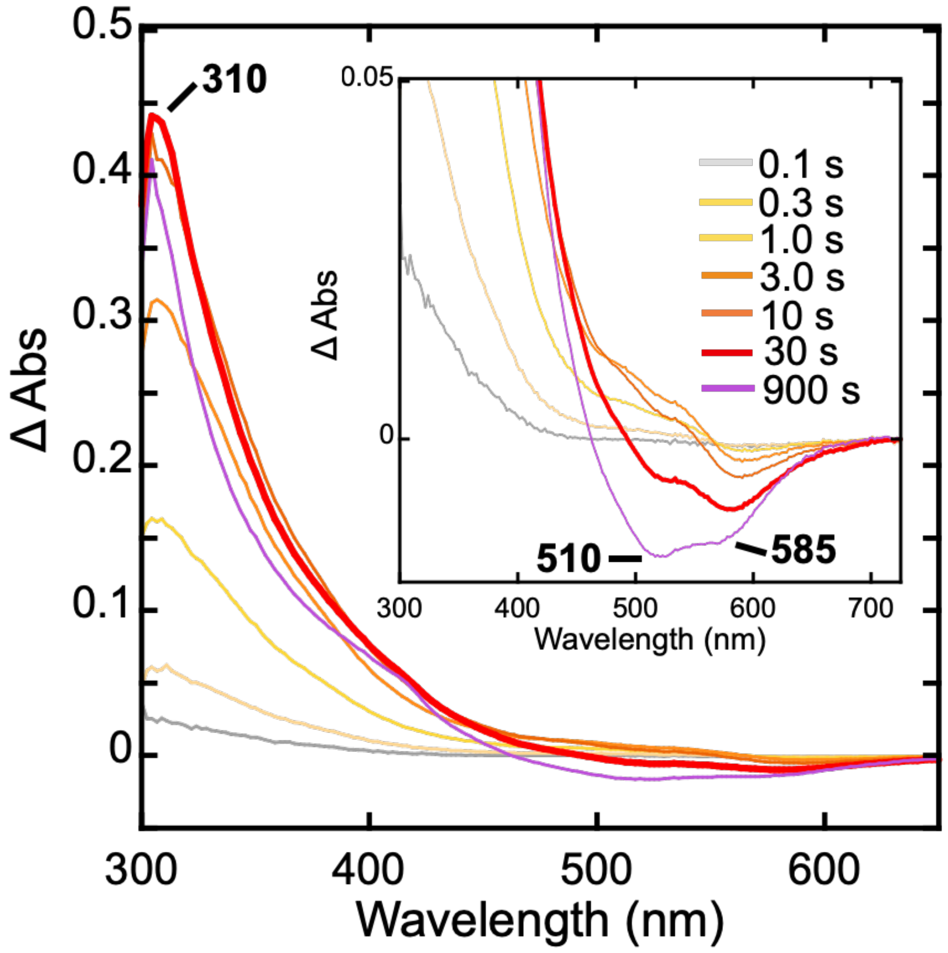

Figure S3. Selected time dependent difference optical spectra of the reaction of the AdeV•Fe^{2+}•2OG•Cl^-•1 complex with O_2. The difference spectra are generated by subtracting the earliest reaction time spectrum (0.006 s) from the spectra of the selected time points. The inset shows the optical features of the Fe(II)-2OG LMCT band.

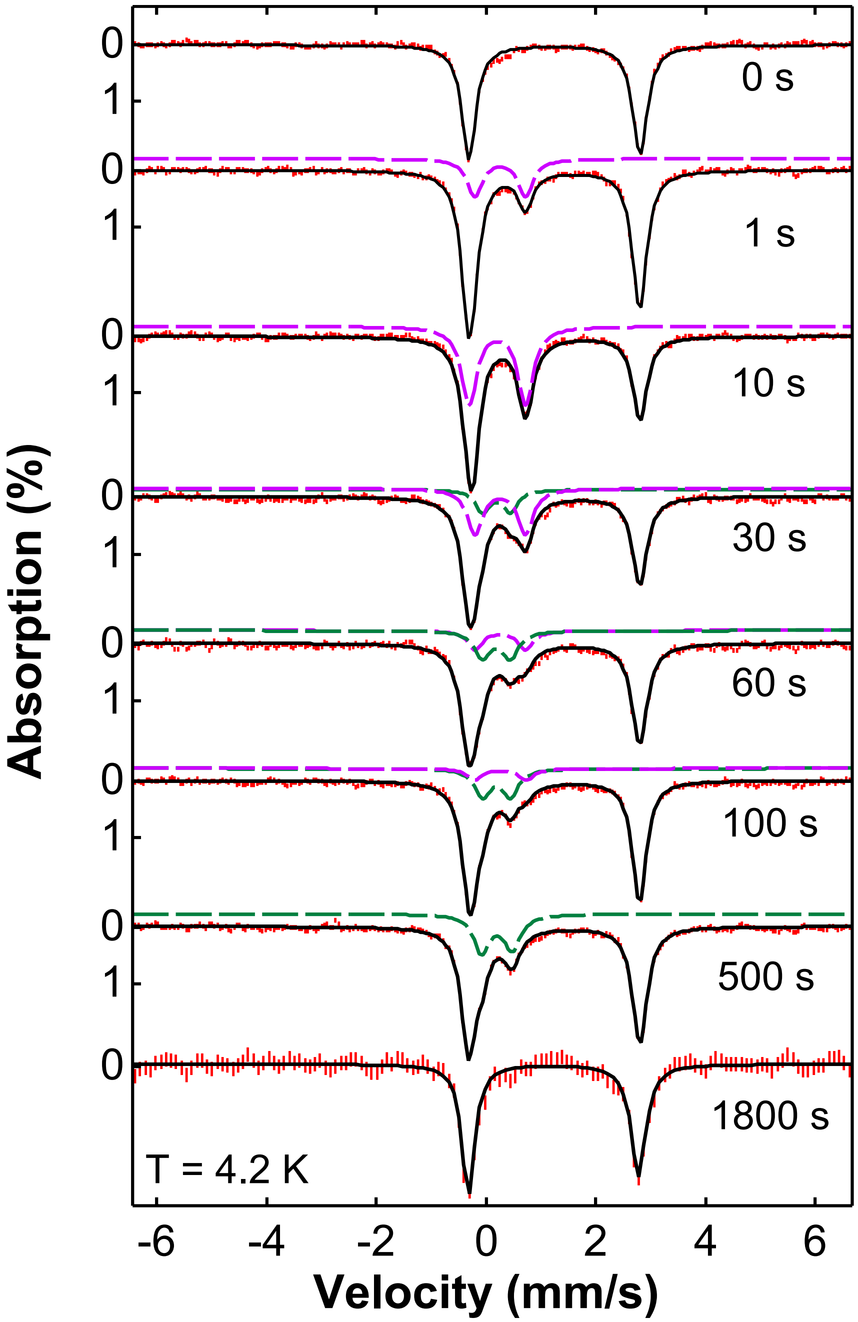

Figure S4. 4.2 K zero field Mössbauer spectra recorded on the freeze-quench AdeV samples at different reaction time points.

The samples were generated from the reaction between the AdeV•Fe(II)•2OG•Cl$^-$•**1** complex and O_2 (see the method section for details). The red vertical bars represent the experimental data, the black solid lines represent the overall spectral simulations, and the purple and the green dashed lines represent the spectral simulations of the Fe^{IV}=O^{1st} and the Fe^{IV}=O^{2nd} intermediates, respectively. The 1800s sample was generated by incubating the AdeV•Fe(II)•2OG•Cl$^-$•**1** complex with O_2-saturated buffer in a sealed container for 1800 s and then frozen in liquid nitrogen.

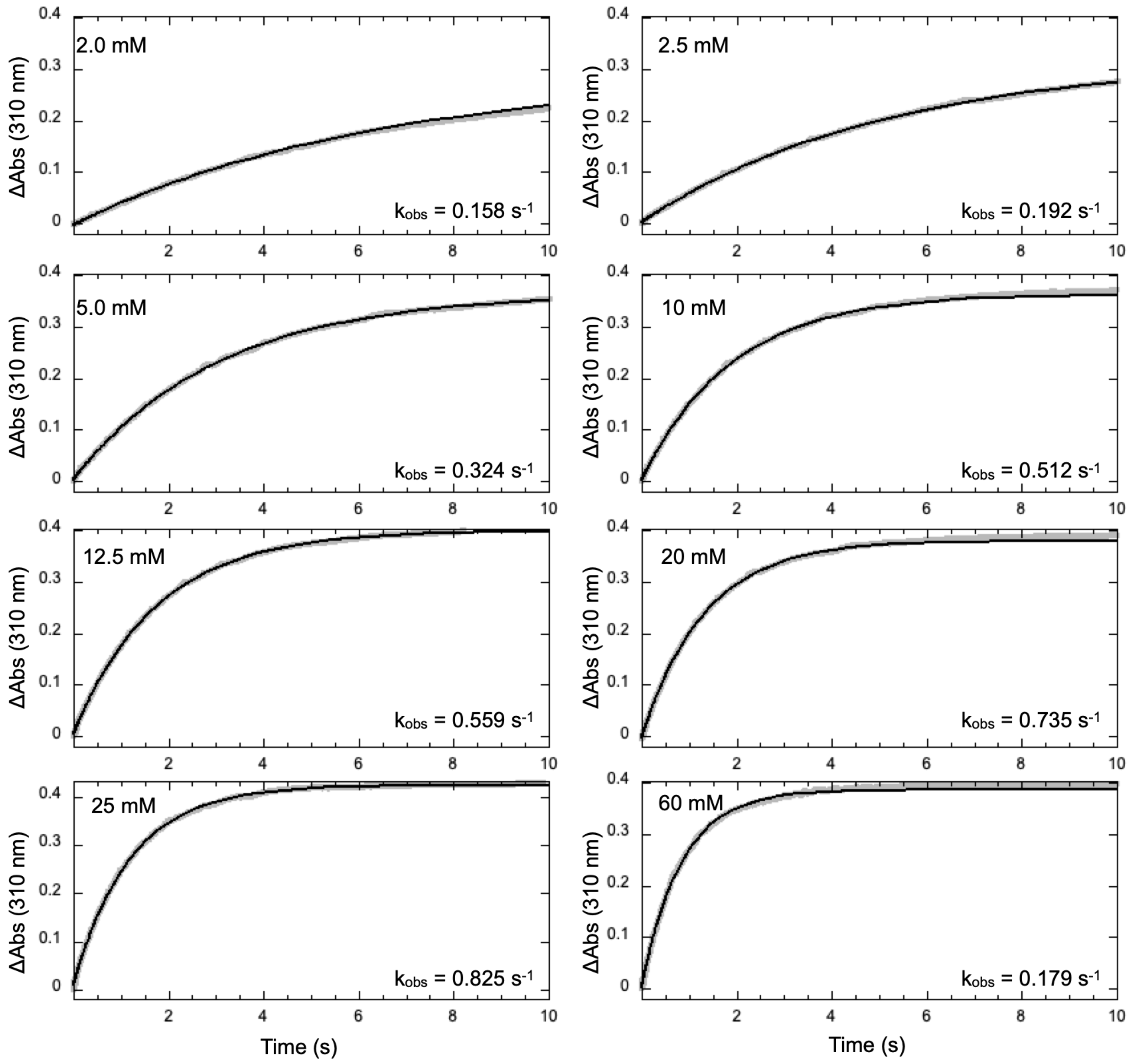

Figure S5. The kinetic simulations for the observed formation rates of the chloro-ferryl intermediate (310 nm) with varying substrate concentration. The substrate concentrations used are indicated in the figure. The following analytical expression is used for the fitting: $y = A\left(1 - e^{k_{obs}t}\right) + c$.

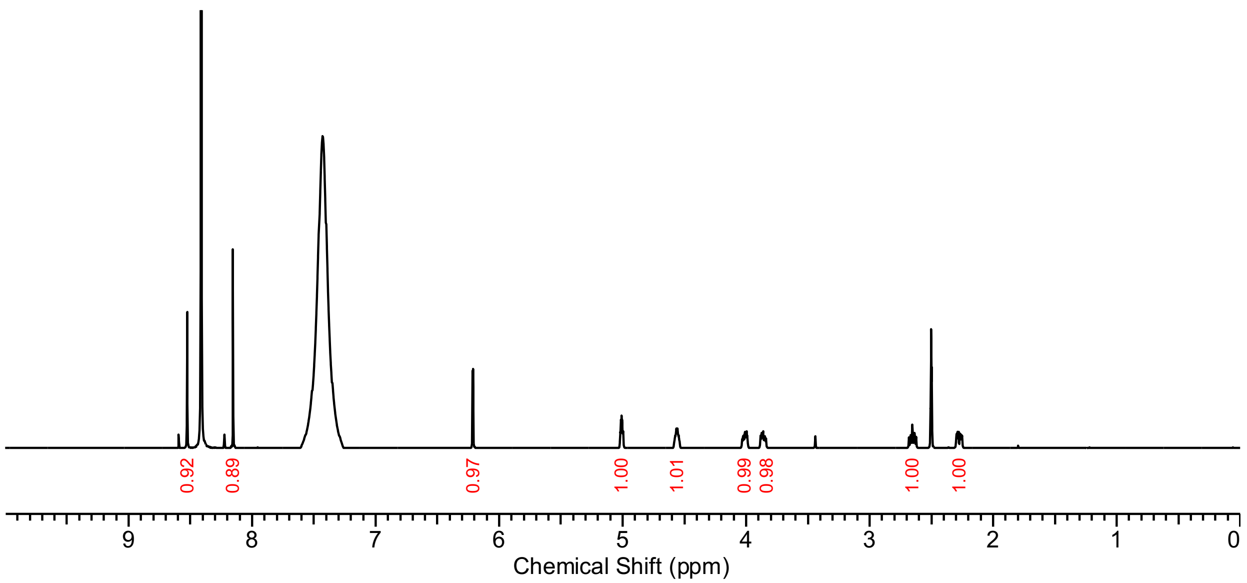
0.92
0.89
0.97
1.00
1.01
0.99
0.98
1.00
1.00
9 8 7 6 5 4 3 2 1 0
Chemical Shift (ppm)

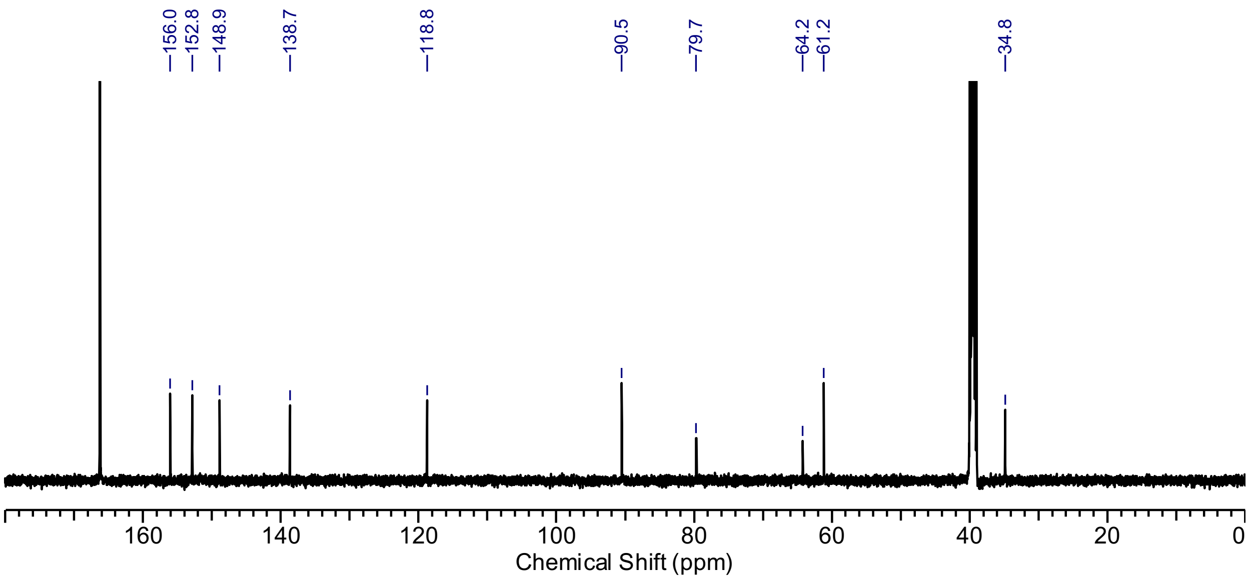
156.0
152.8
148.9
138.7
118.8
90.5
79.7
64.2
61.2
34.8
160 140 120 100 80 60 40 20 0
Chemical Shift (ppm)

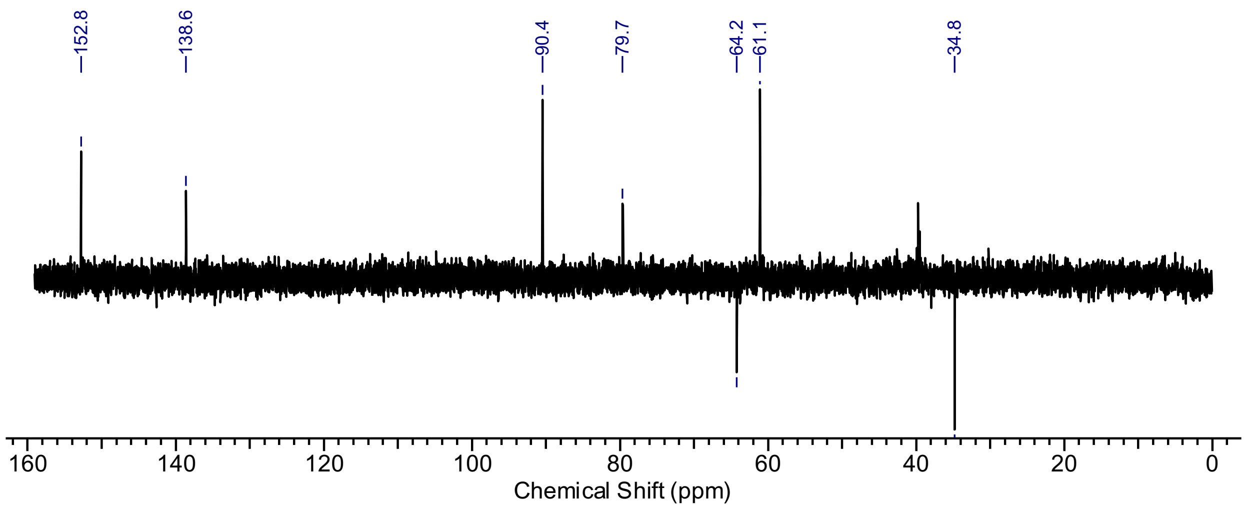
152.8
138.6
90.4
79.7
64.2
61.1
34.8
160 140 120 100 80 60 40 20 0
Chemical Shift (ppm)

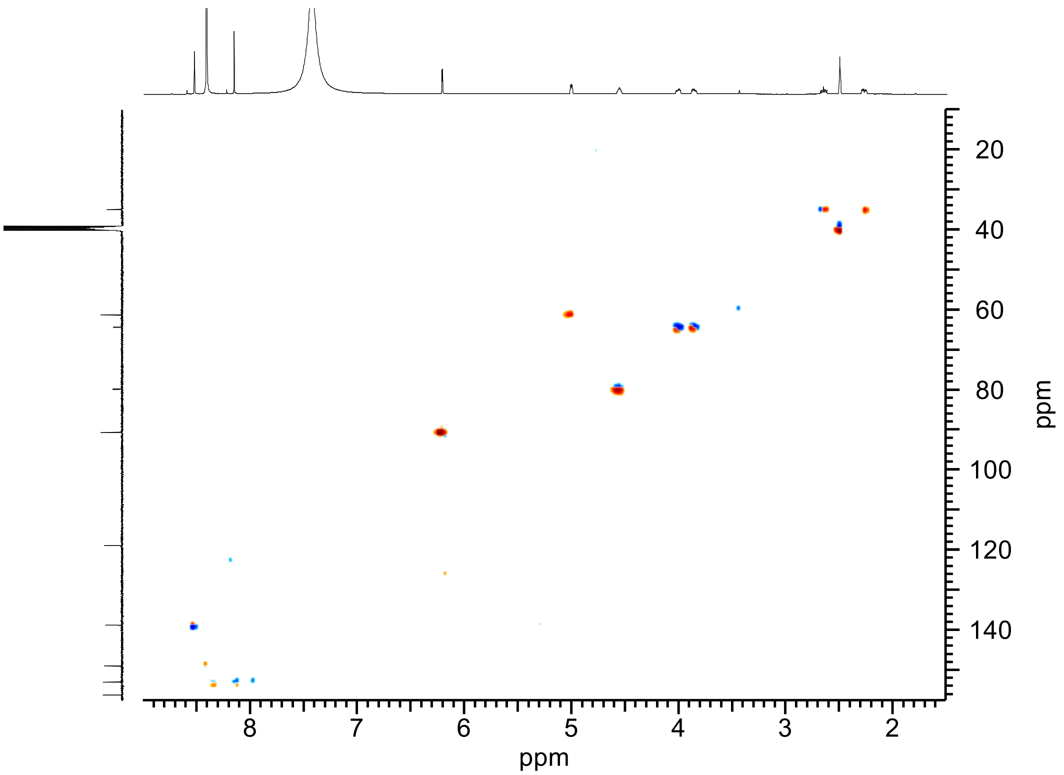
20
40
60
80
100
120
140
ppm
8
7
6
5
4
3
2
ppm

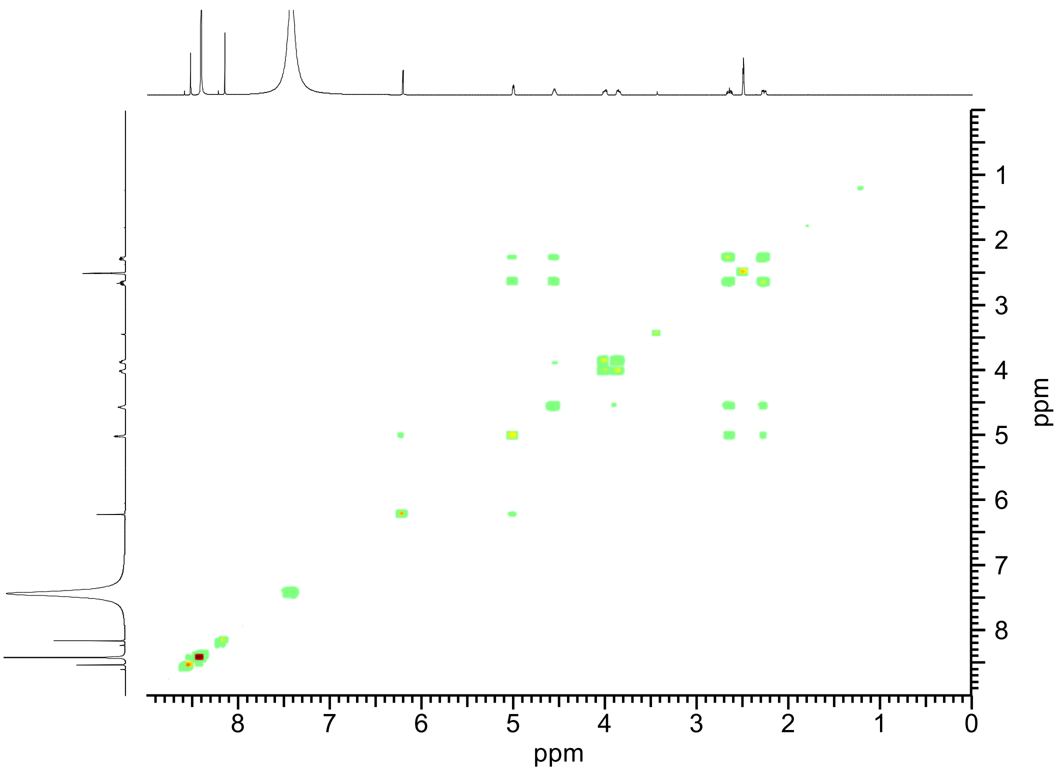
1
2
3
4
5
6
7
8
ppm
8
7
6
5
4
3
2
1
0
ppm

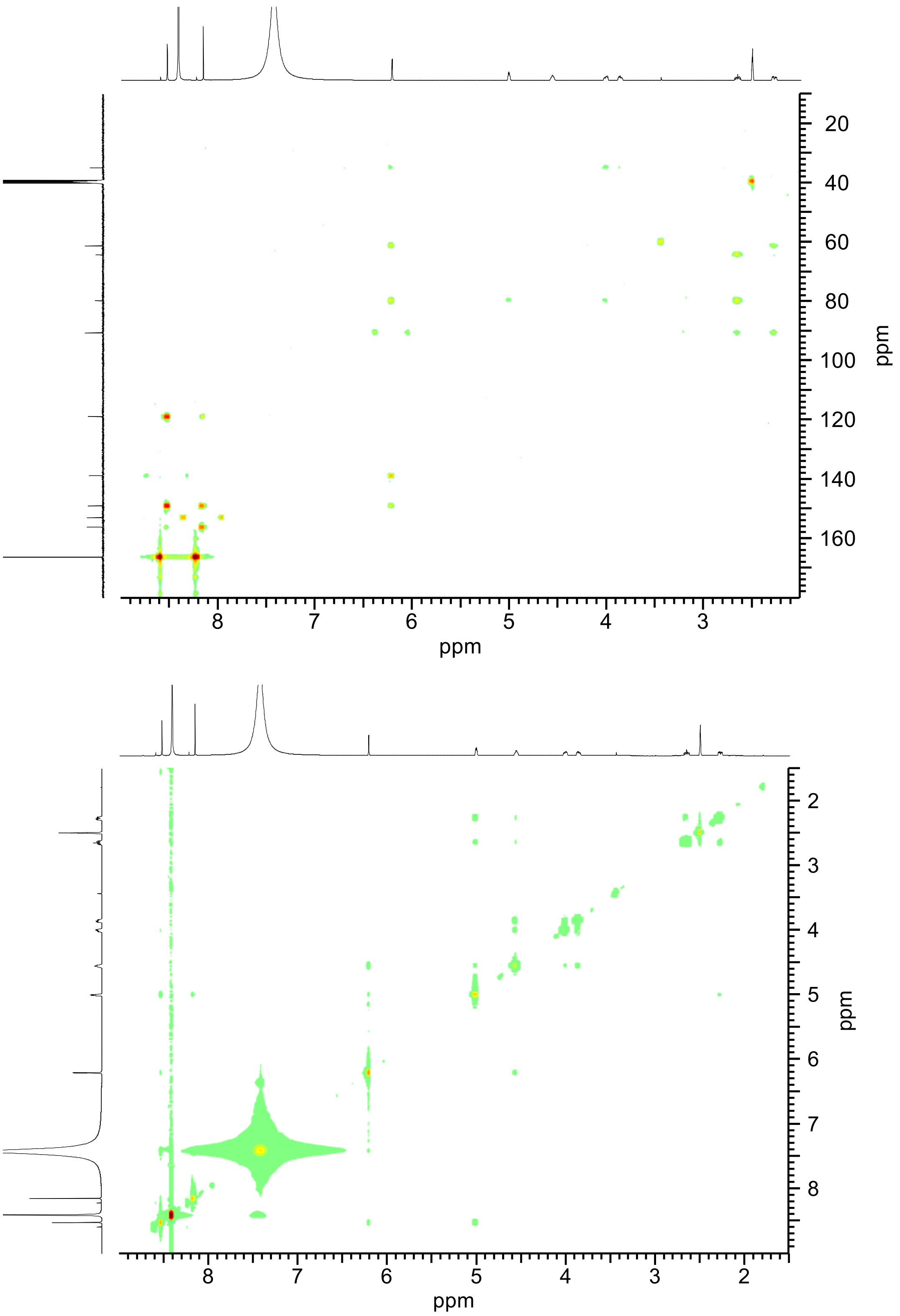

Figure S6. ^{1}H, ^{13}C, DEPT135, HSQC, COSY, HMBC and NOESY NMR of (1′*R*, 2′*R*, 4′*S*)-2′-chloro-2′,3′-dideoxyadenosine monophosphate.

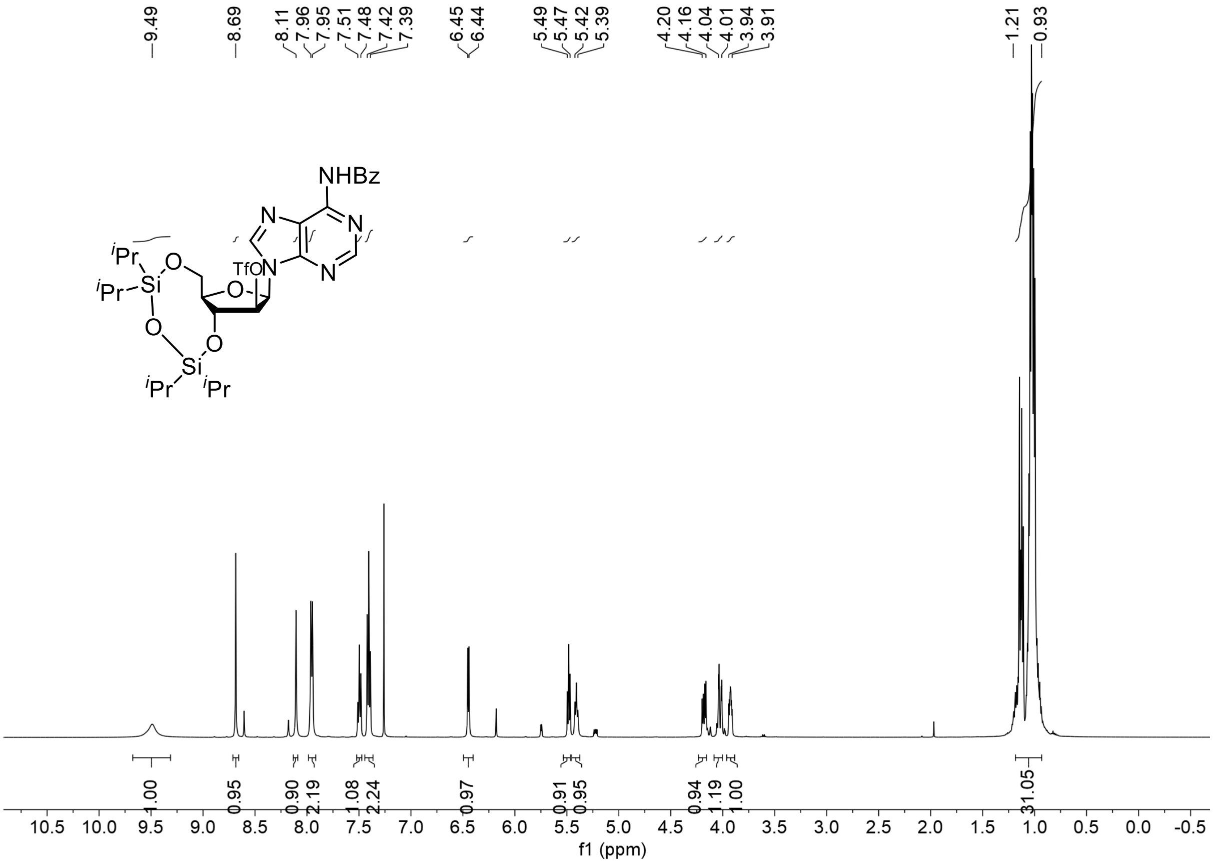
9.49
8.69
8.11
7.96
7.95
7.51
7.48
7.42
7.39
6.45
6.44
5.49
5.47
5.42
5.39
4.20
4.16
4.04
4.01
3.94
3.91
1.21
0.93
NHBz
N
N
N
N
TfO
O
O
O
O
Si
Si
iPr
iPr
iPr
iPr
1.00
0.95
0.90
2.19
1.08
2.24
0.97
0.91
0.95
0.94
1.19
1.00
31.05
f1 (ppm)

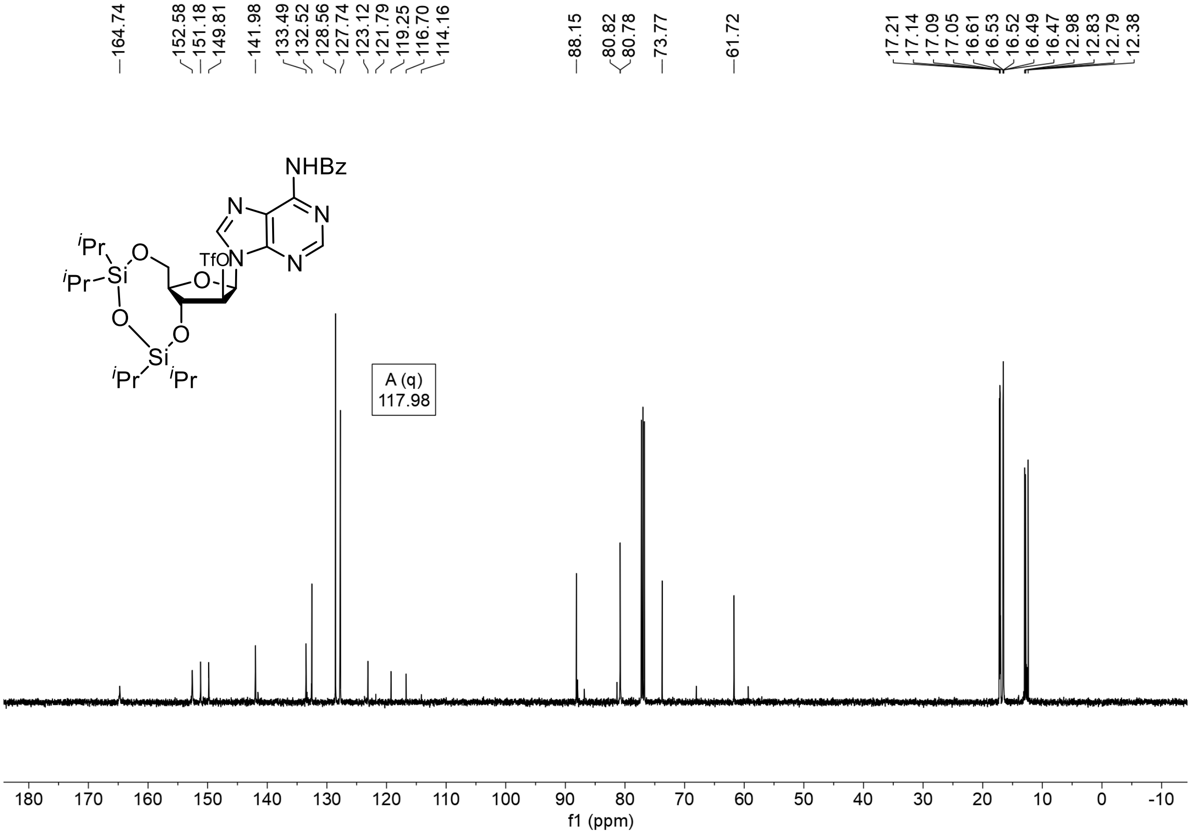
164.74
152.58
151.18
149.81
141.98
133.49
132.52
128.56
127.74
123.12
121.79
119.25
116.70
114.16
88.15
80.82
80.78
73.77
61.72
17.21
17.14
17.09
17.05
16.61
16.53
16.52
16.49
16.47
12.98
12.83
12.79
12.38
NHBz
N
N
N
N
TfO
O
O
O
O
Si
Si
iPr
iPr
iPr
iPr
A (q)
117.98
f1 (ppm)

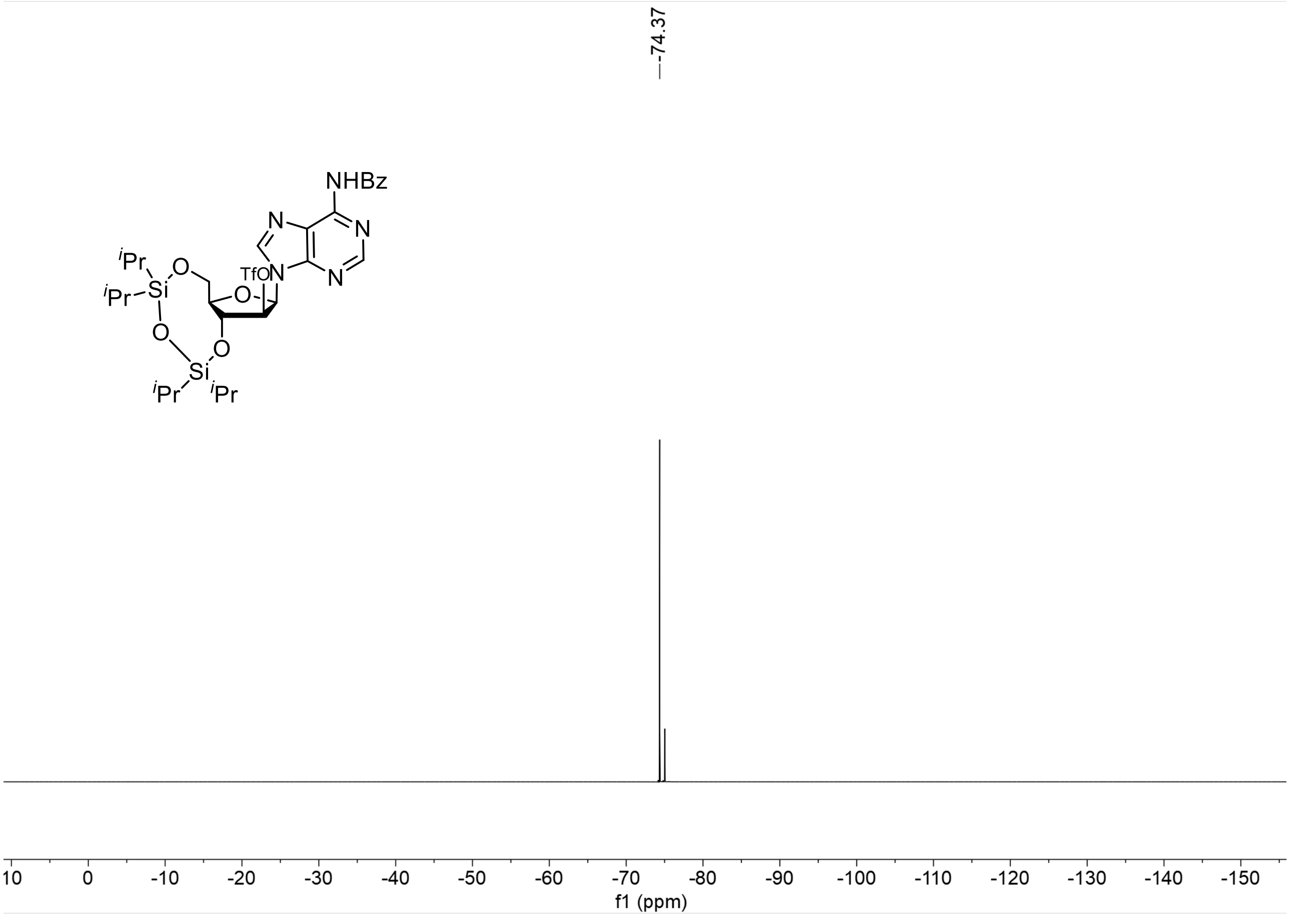

Figure S7. ^{1}H, ^{13}C and ^{19}F NMR of (*6aR,8R,9S,9aR*)-8-(6-benzamido-*9H*-purin-9-yl)-2,2,4,4-tetraisopropyltetrahydro-*6H*-furo[3,2-*f*][1,3,5,2,4]trioxadisilocin-9-yl trifluoromethanesulfonate.

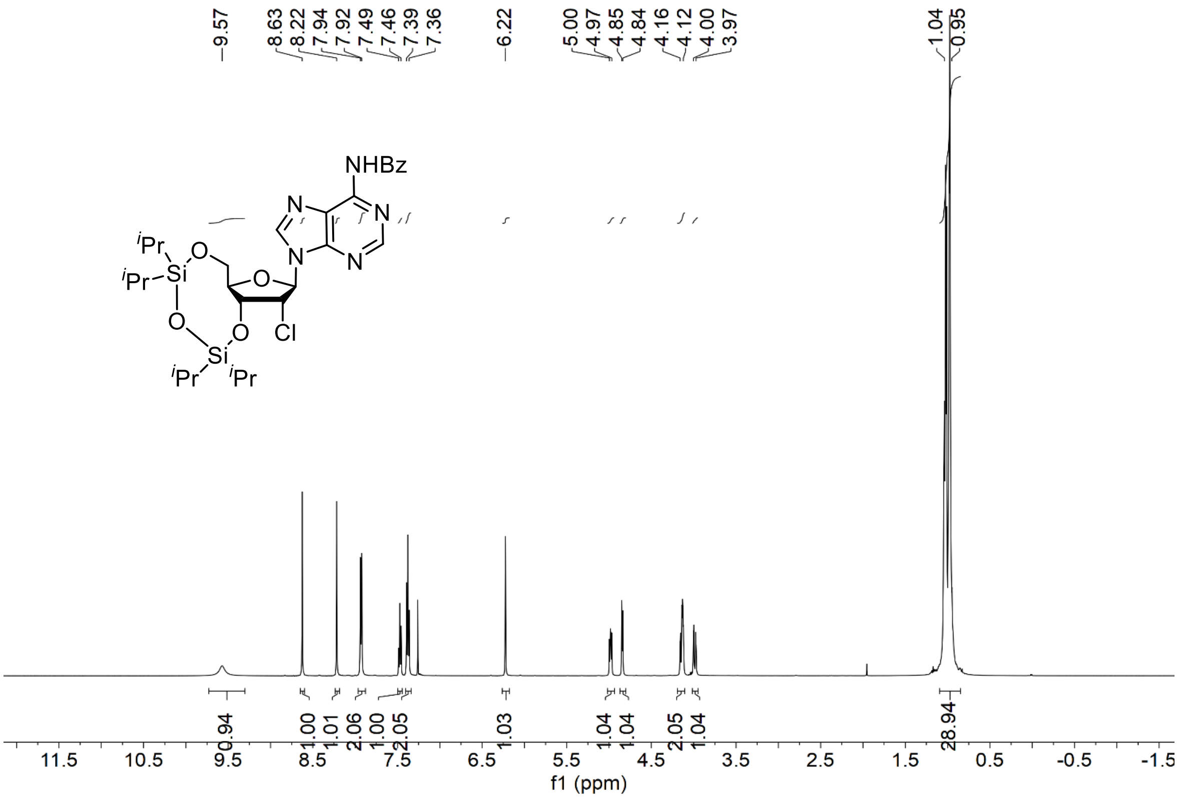

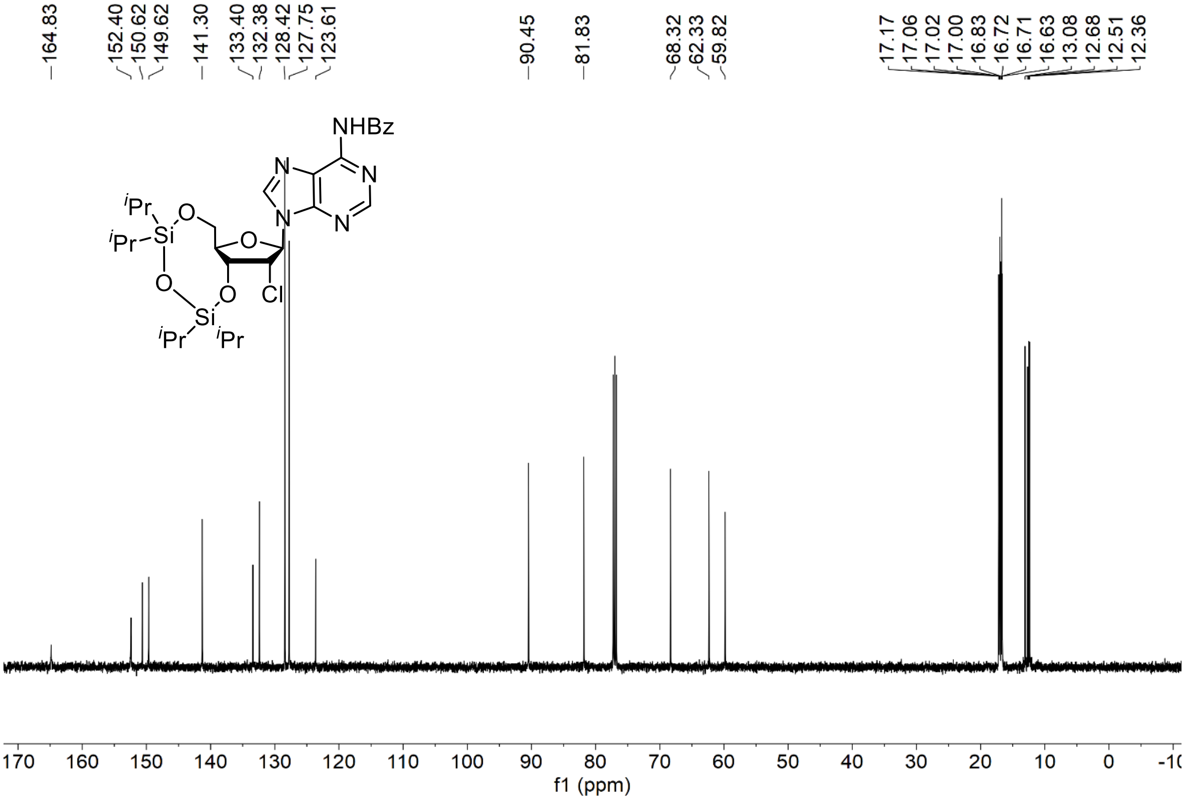

Figure S8. ^{1}H and ^{13}C NMR of *N*-(9-((*6aR,8R,9R,9aR*)-9-chloro-2,2,4,4-tetraisopropyltetrahydro-*6H*-furo[3,2-*f*][1,3,5,2,4]trioxadisilocin-8-yl)-*9H*-purin-6-yl)benzamide.

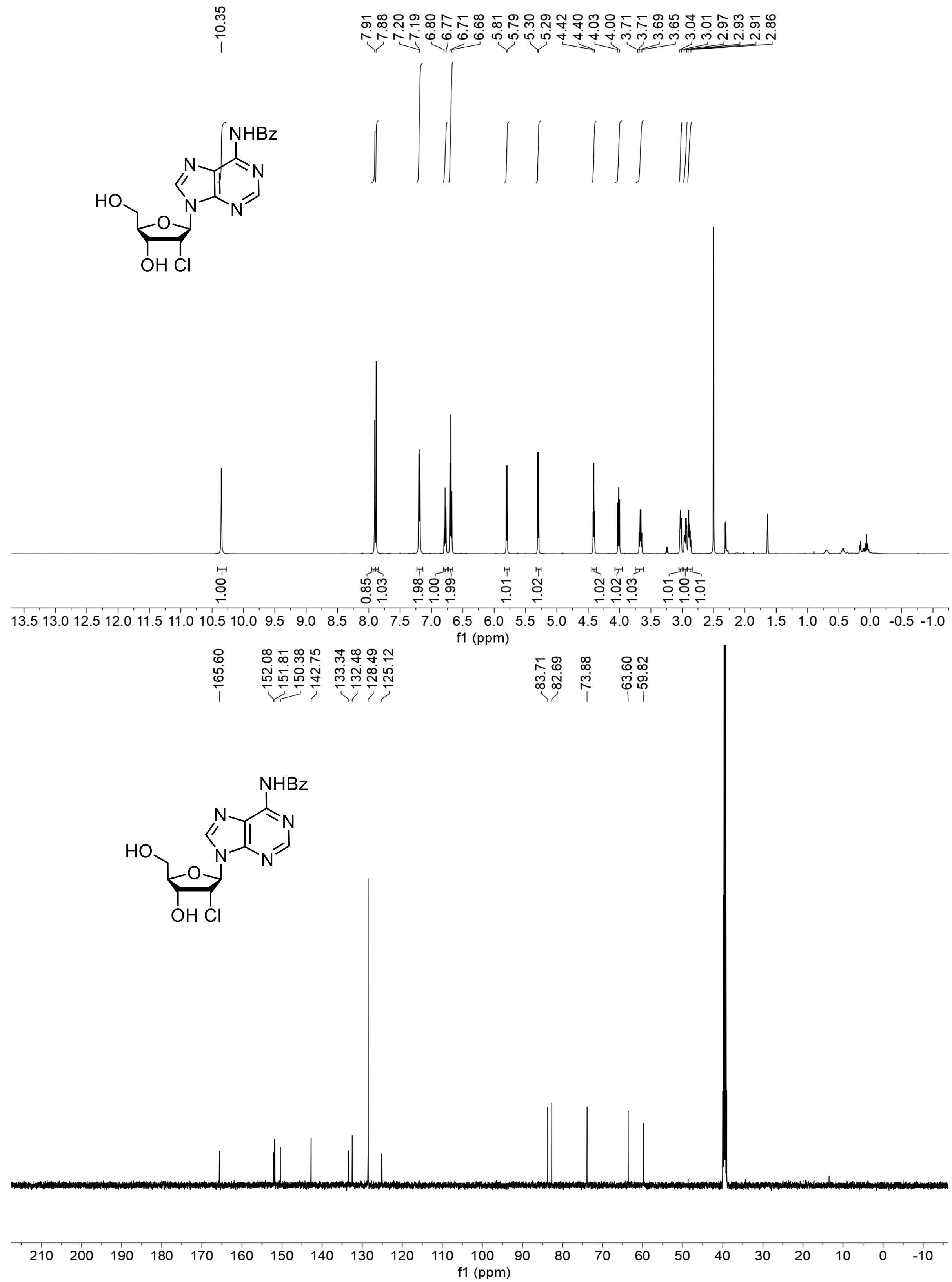

Figure S9. ^{1}H and ^{13}C NMR of N-(9-((2R,3R,4R,5R)-3-chloro-4-hydroxy-5-(hydroxymethyl)tetrahydrofuran-2-yl)-9H-purin-6-yl)benzamide.

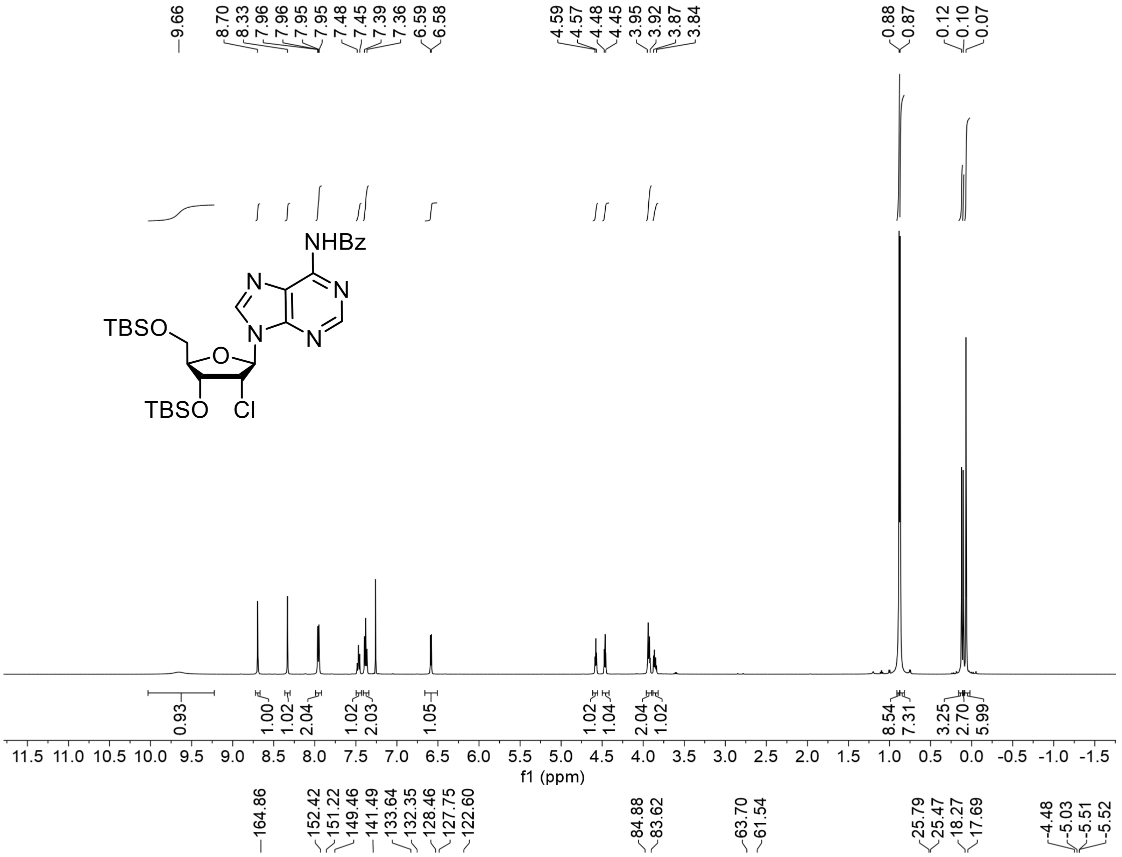

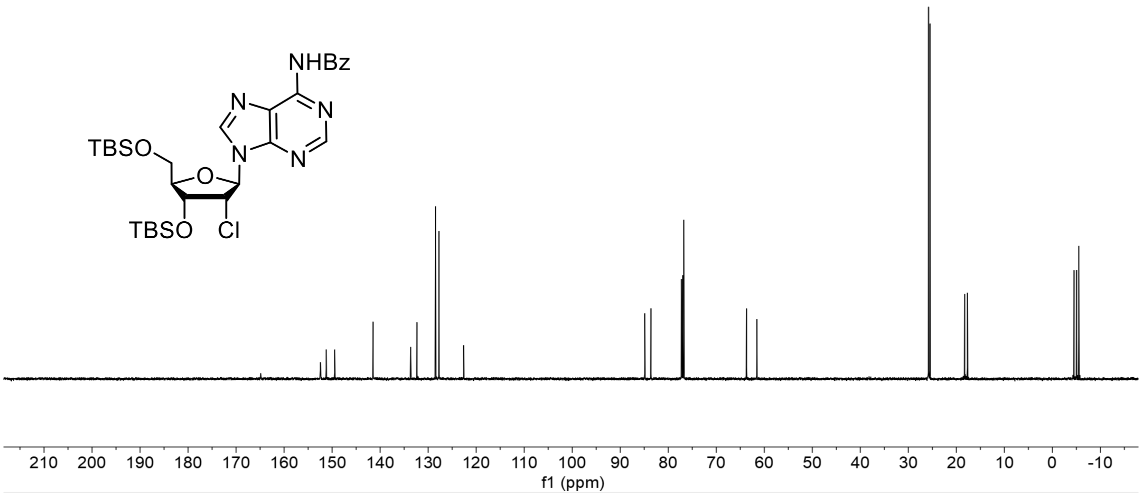

Figure S10. ^{1}H and ^{13}C NMR of *N-(9-((2R,3R,4R,5R)*-4-((*tert*-butyldimethylsilyl)oxy)-5-(((*tert*-butyldimethylsilyl)oxy)methyl)-3-chlorotetrahydrofuran-2-yl)-*9H*-purin-6-yl)benzamide.

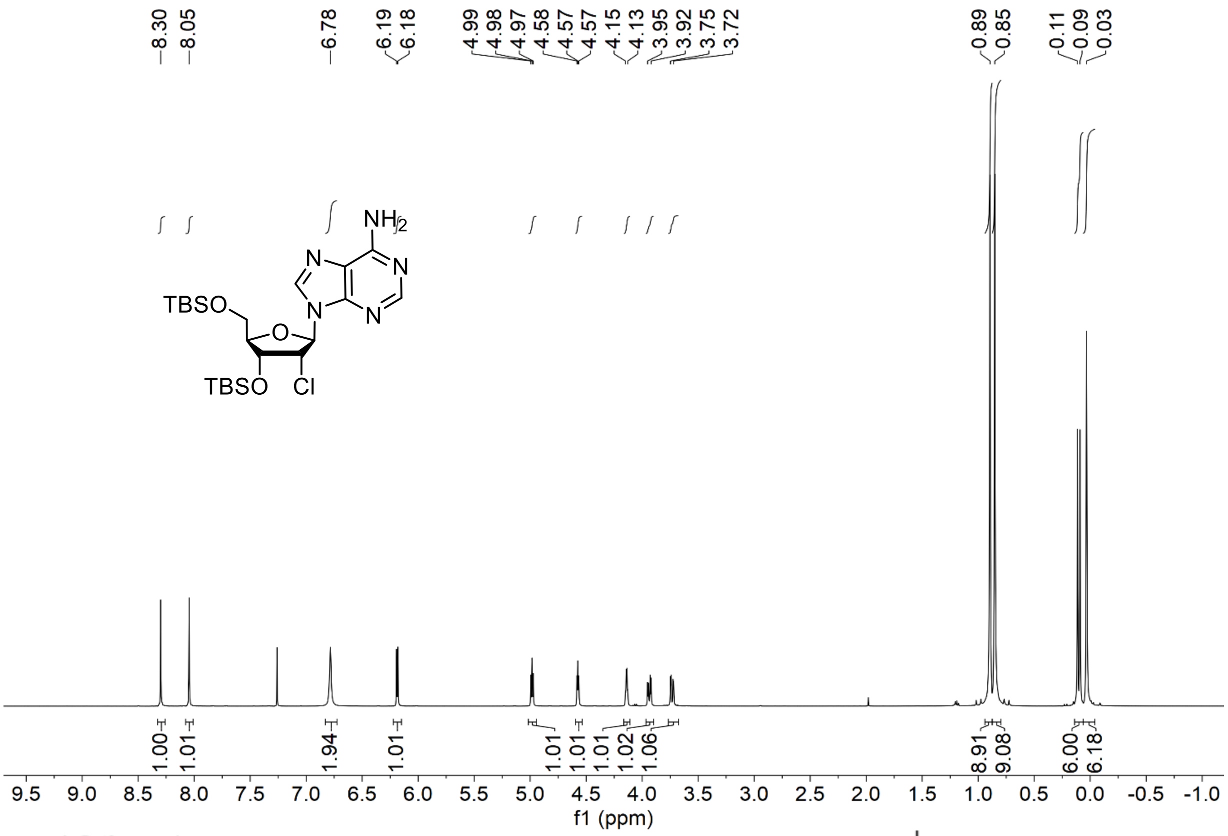

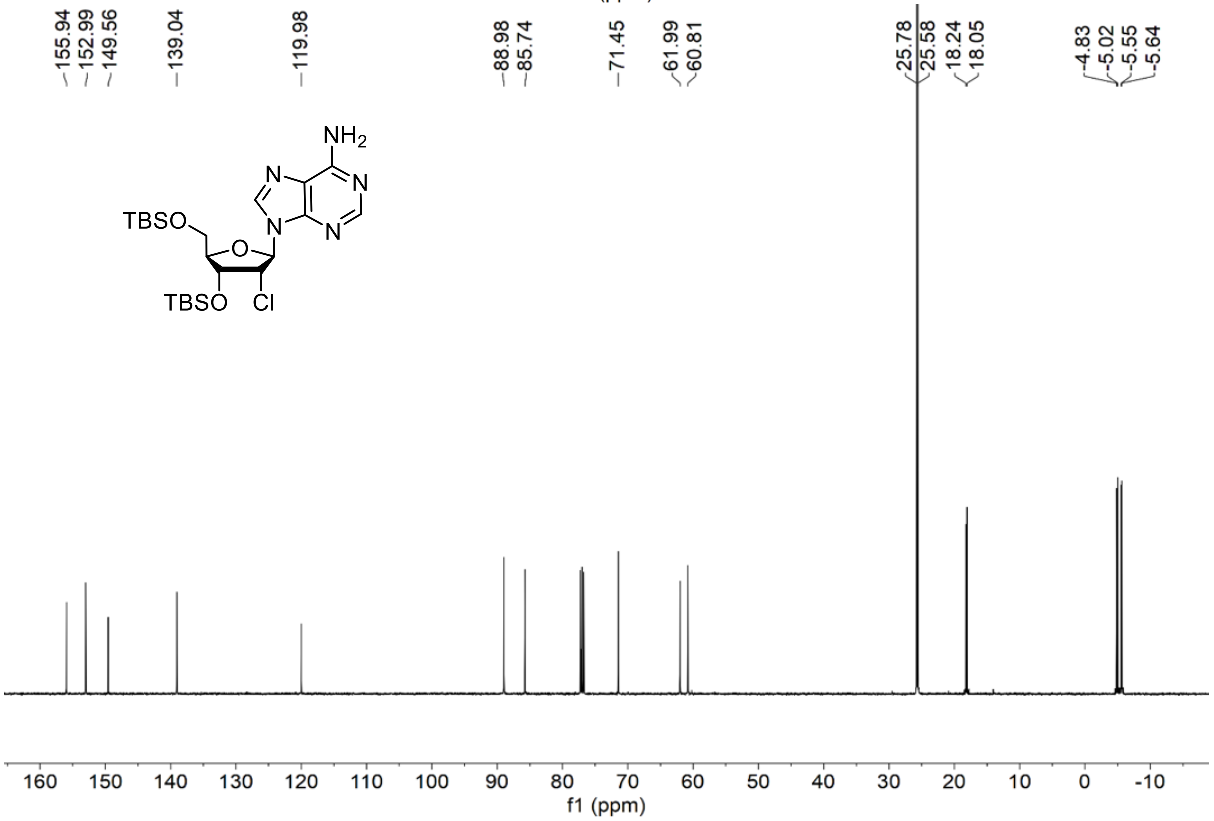

Figure S11. ^{1}H and ^{13}C NMR of 9-((2R,3R,4R,5R)-4-((tert-butyldimethylsilyl)oxy)-5-(((tert-butyldimethylsilyl)-oxy)methyl)-3-chlorotetrahydrofuran-2-yl)-9H-purin-6-amine.

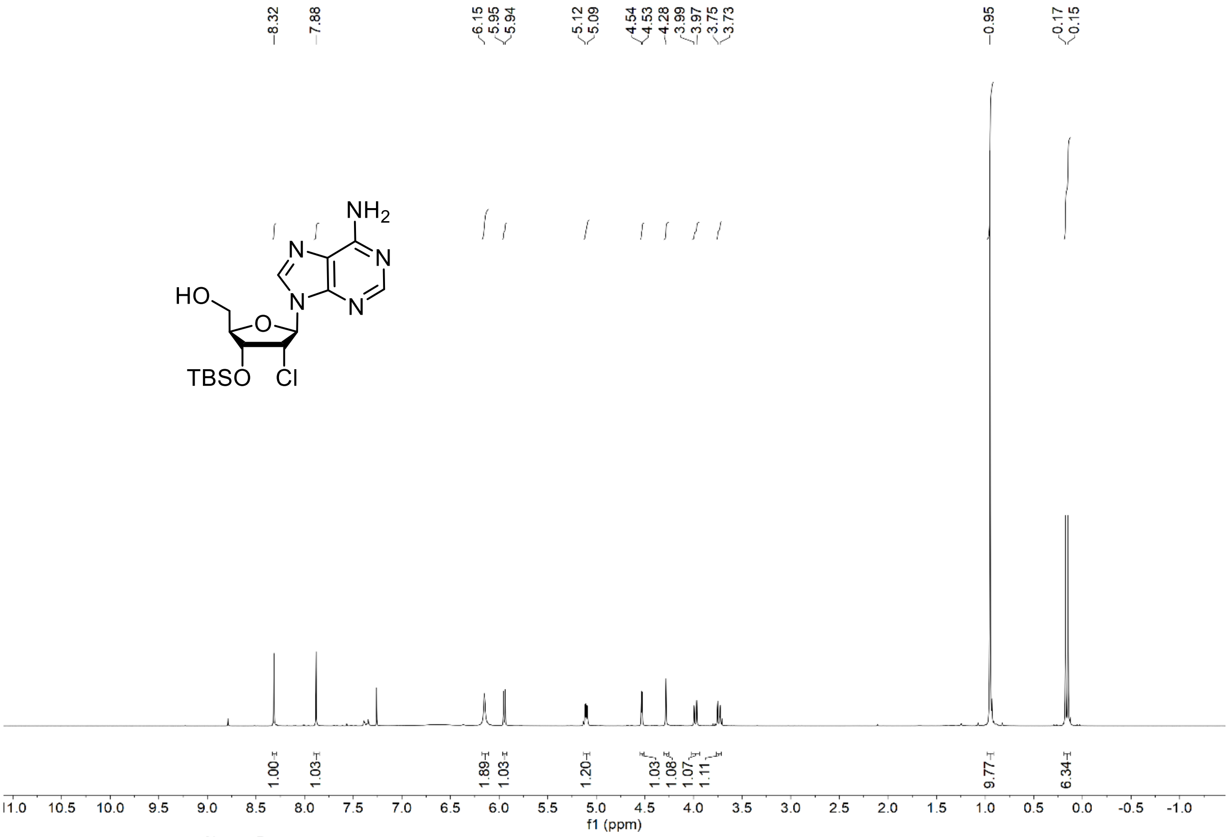

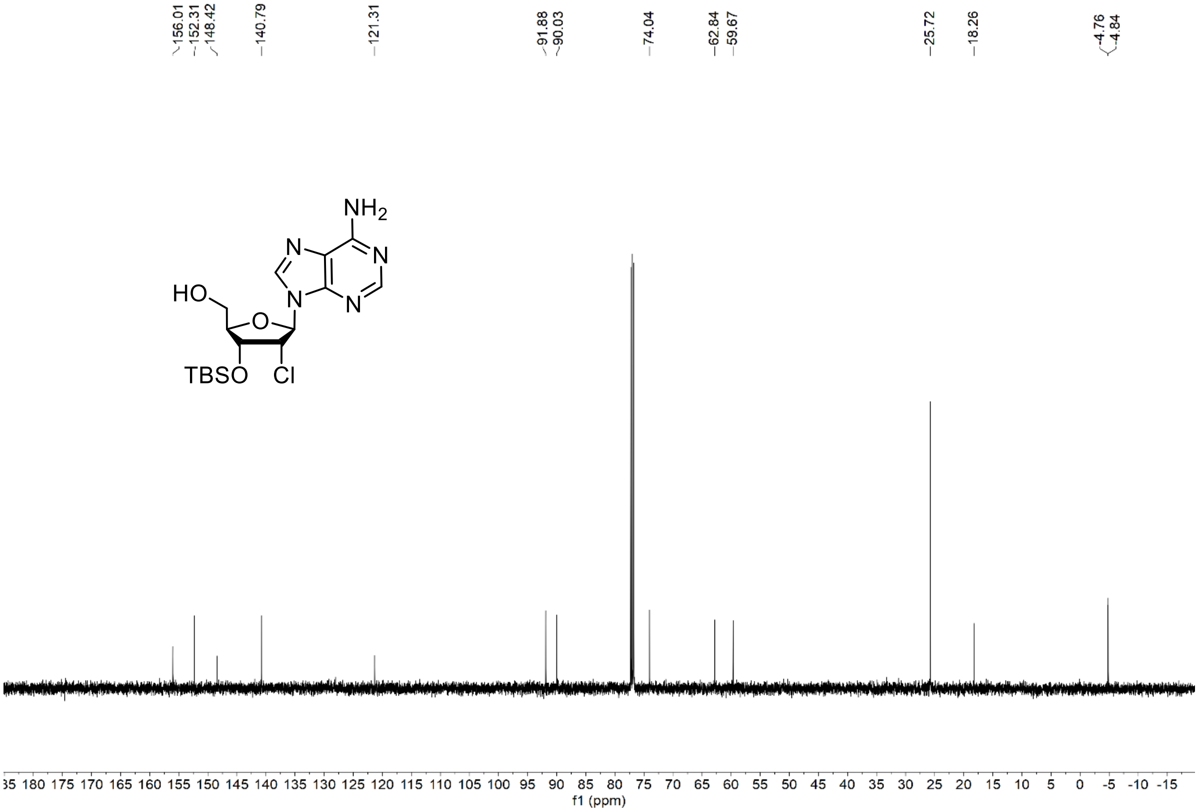

Figure S12. ^{1}H and ^{13}C NMR of ((2R,3R,4R,5R)-5-(6-amino-9H-purin-9-yl)-3-((tert-butyldimethylsilyl)oxy)-4-chlorotetrahydrofuran-2-yl)methanol.

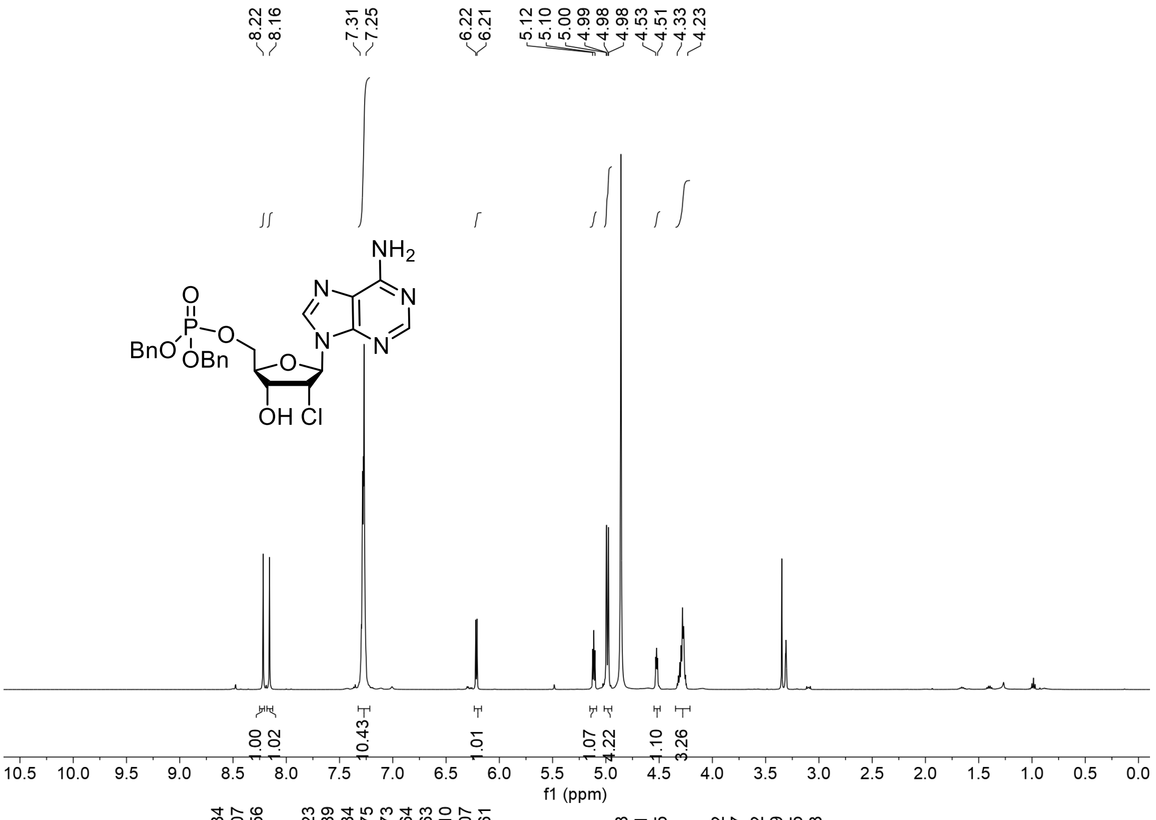

8.22
8.16
7.31
7.25
6.22
6.21
5.12
5.10
5.00
4.99
4.98
4.98
4.53
4.51
4.33
4.23
NH2
N
N
N
N
O
P
O
BnO
OBn
O
OH
Cl
1.00
1.02
10.43
1.01
1.07
4.22
1.10
3.26
f1 (ppm)

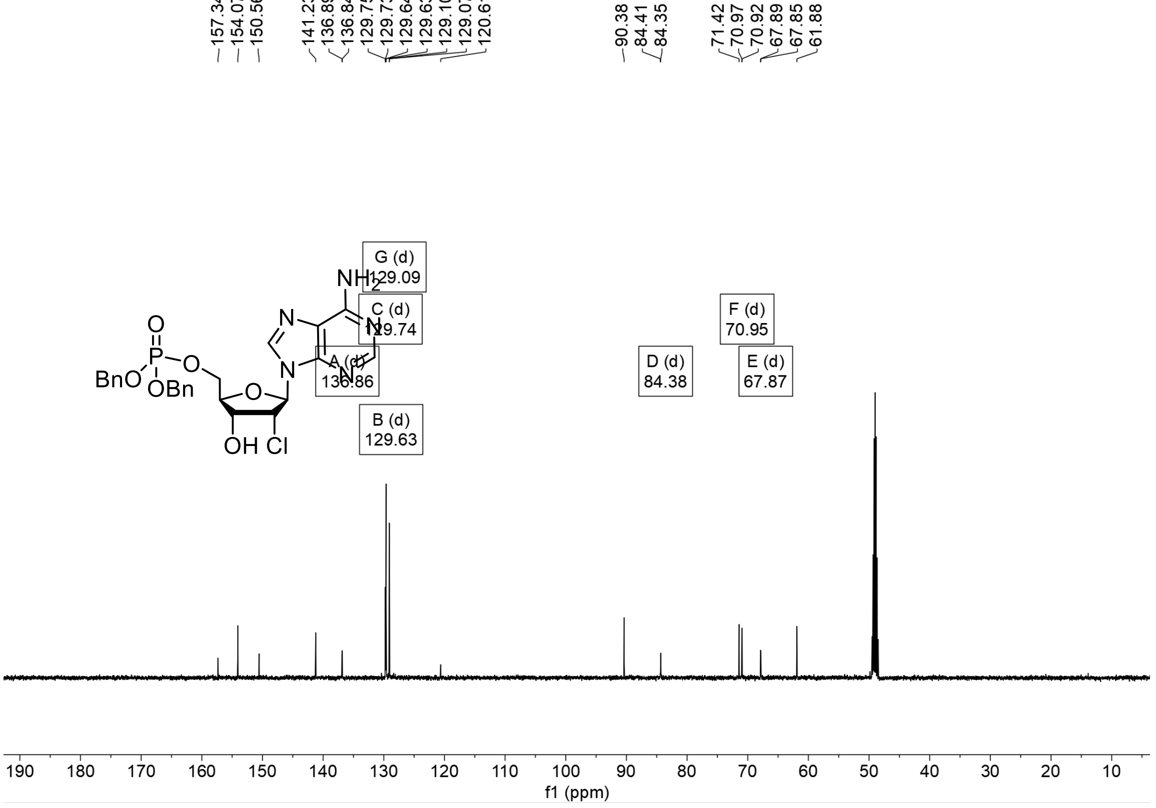

157.34
154.07
150.56
141.23
136.89
136.84
129.75
129.73
129.64
129.63
129.10
129.07
120.61
90.38
84.41
84.35
71.42
70.97
70.92
67.89
67.85
61.88
G (d)
129.09
C (d)
129.74
A (d)
136.86
B (d)
129.63
D (d)
84.38
F (d)
70.95
E (d)
67.87
NH2
BnO
OBn
OH
Cl
f1 (ppm)

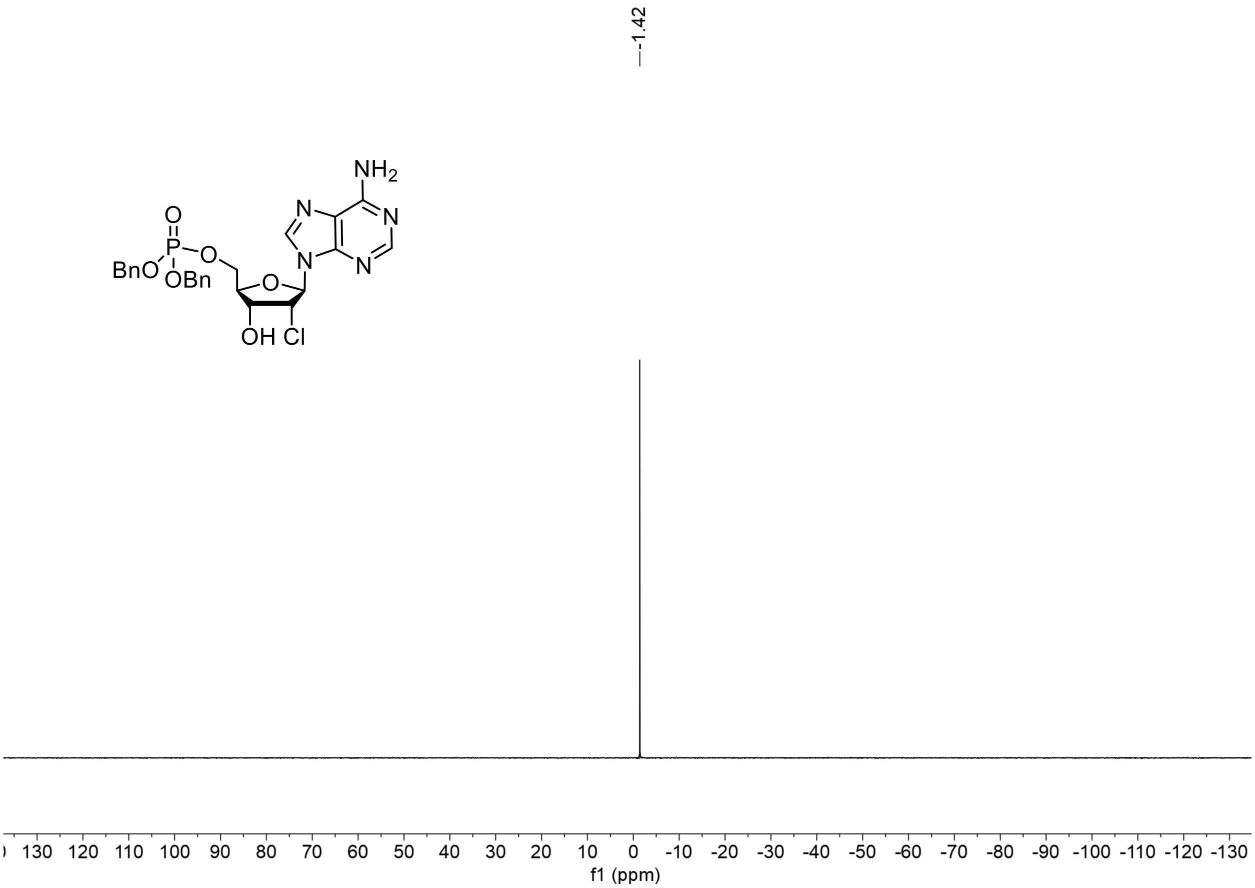

Figure S13. ^{1}H ^{13}C and ^{31}P NMR of ((2R,3R,4R,5R)-5-(6-amino-9H-purin-9-yl)-4-chloro-3-hydroxy-tetrahydrofuran-2-yl)methyl dibenzyl phosphate.

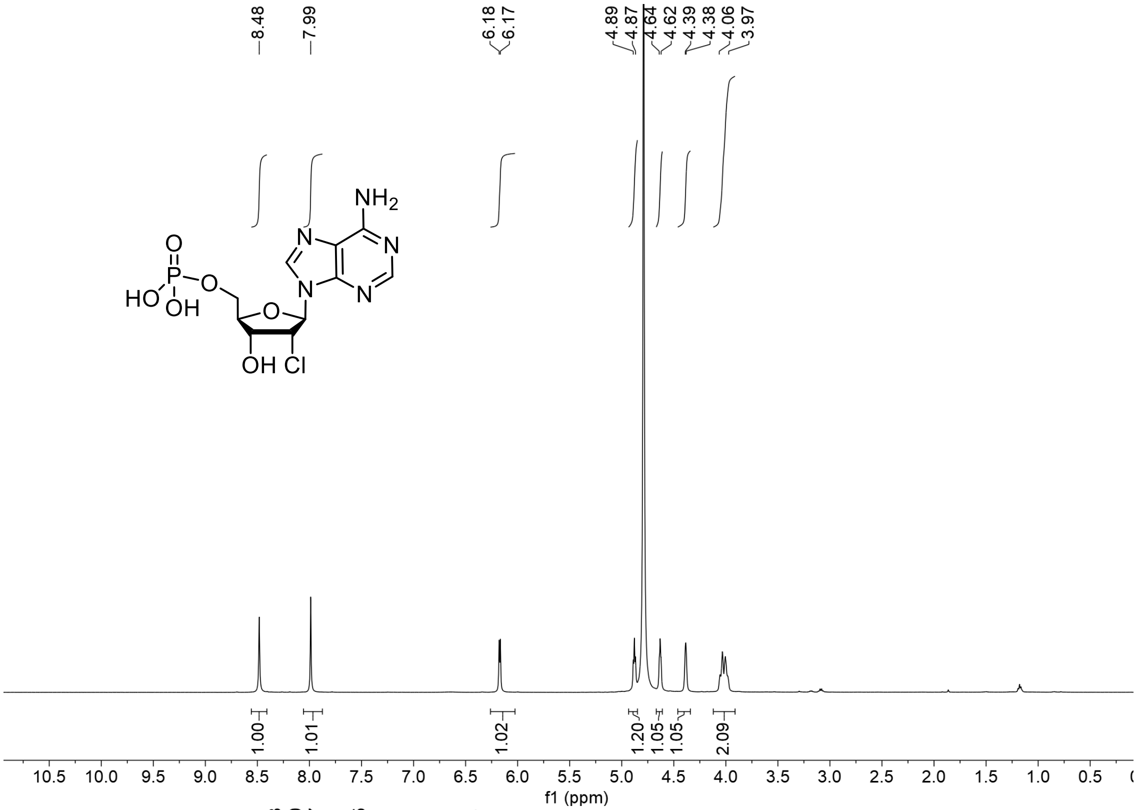
8.48
7.99
6.18
6.17
4.89
4.87
4.64
4.62
4.39
4.38
4.06
3.97
NH2
N
N
N
N
O
P
O
HO
OH
O
OH
Cl
1.00
1.01
1.02
1.20
1.05
1.05
2.09
10.5 10.0 9.5 9.0 8.5 8.0 7.5 7.0 6.5 6.0 5.5 5.0 4.5 4.0 3.5 3.0 2.5 2.0 1.5 1.0 0.5
f1 (ppm)

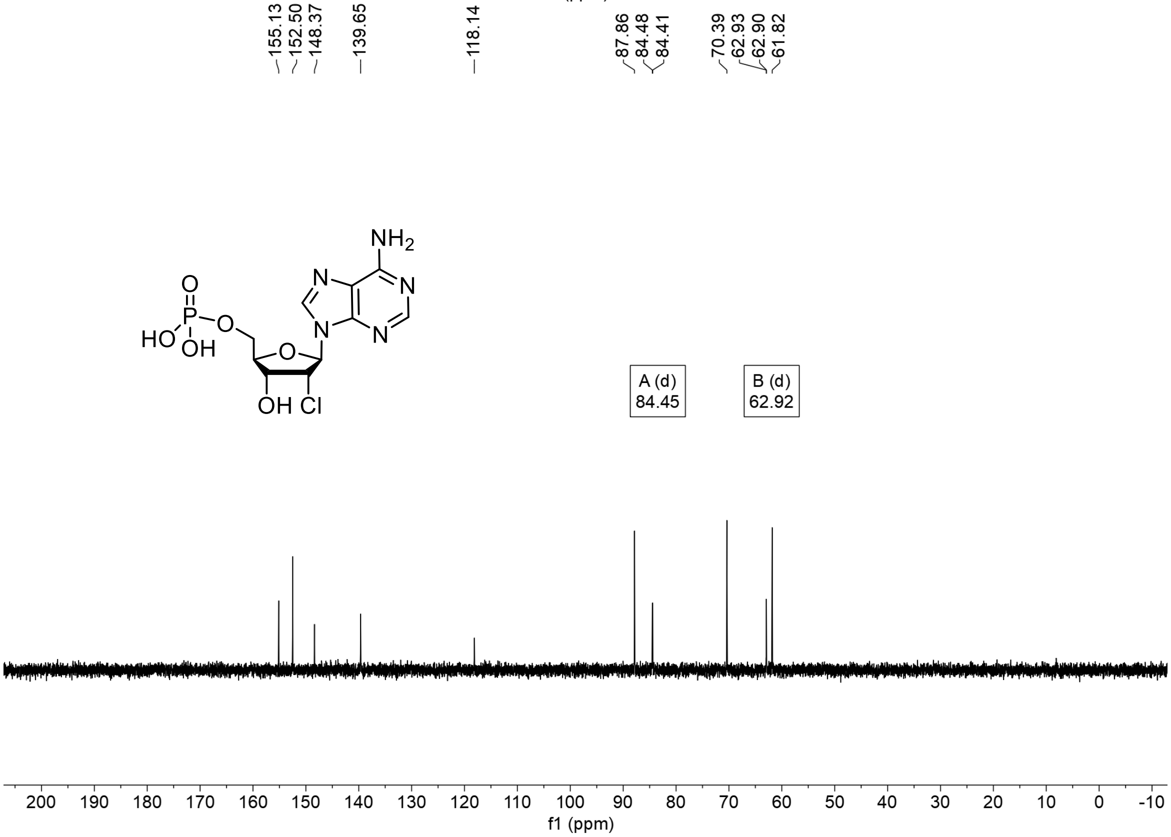
155.13
152.50
148.37
139.65
118.14
87.86
84.48
84.41
70.39
62.93
62.90
61.82
NH2
N
N
N
N
O
P
O
HO
OH
O
OH
Cl
A (d)
84.45
B (d)
62.92
200 190 180 170 160 150 140 130 120 110 100 90 80 70 60 50 40 30 20 10 0 -10
f1 (ppm)

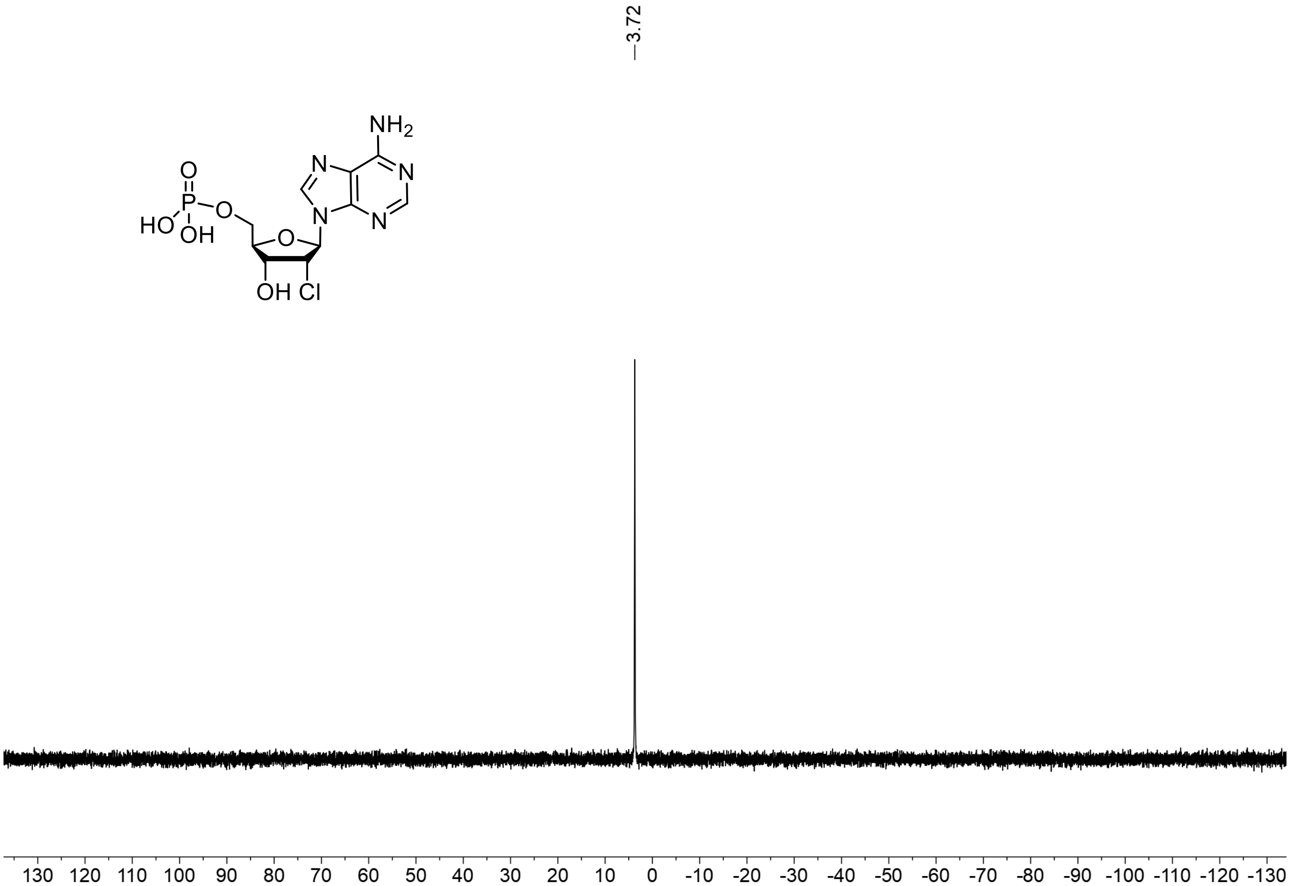

Figure S14. ^{1}H ^{13}C and ^{31}P NMR of ((2R,3R,4R,5R)-5-(6-amino-9H-purin-9-yl)-4-chloro-3-hydroxytetrahydrofuran-2-yl)methyl dihydrogen phosphate.

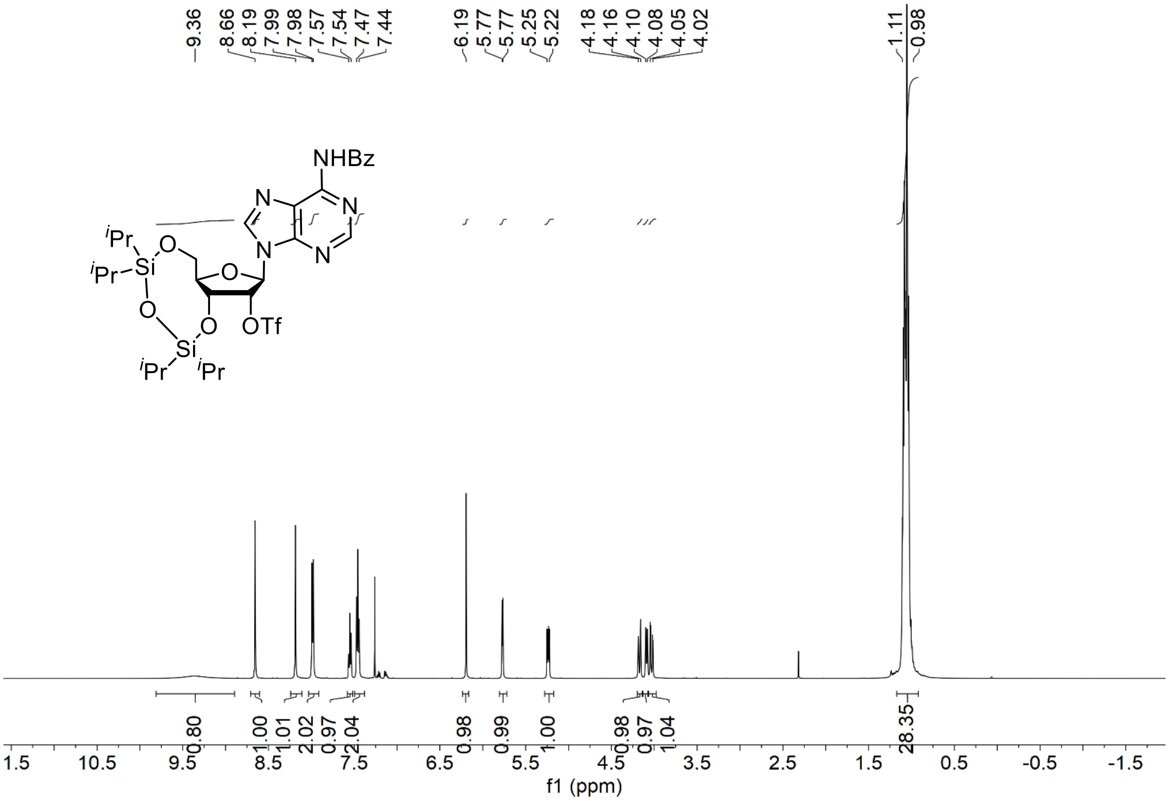
9.36 8.66 8.19 7.99 7.98 7.57 7.54 7.47 7.44 6.19 5.77 5.77 5.25 5.22 4.18 4.16 4.10 4.08 4.05 4.02 1.11 0.98
NHBz
N
N
N
N
O
O
O
O
OTf
Si
Si
iPr
iPr
iPr
iPr
0.80 1.00 1.01 2.02 0.97 2.04 0.98 0.99 1.00 0.98 0.97 1.04 28.35
1.5 10.5 9.5 8.5 7.5 6.5 5.5 4.5 3.5 2.5 1.5 0.5 -0.5 -1.5
f1 (ppm)
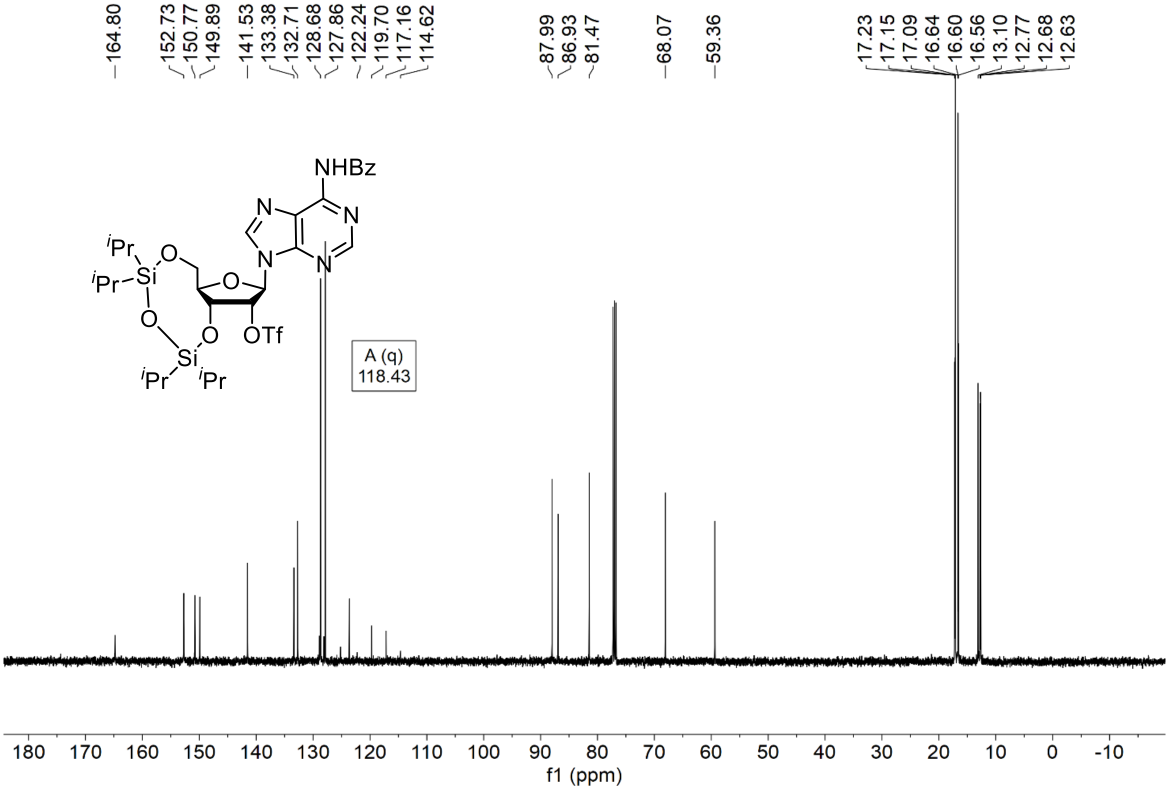
164.80 152.73 150.77 149.89 141.53 133.38 132.71 128.68 127.86 122.24 119.70 117.16 114.62 87.99 86.93 81.47 68.07 59.36 17.23 17.15 17.09 16.64 16.60 16.56 13.10 12.77 12.68 12.63
NHBz
N
N
N
N
O
O
O
O
OTf
Si
Si
iPr
iPr
iPr
iPr
A (q)
118.43
180 170 160 150 140 130 120 110 100 90 80 70 60 50 40 30 20 10 0 -10
f1 (ppm)

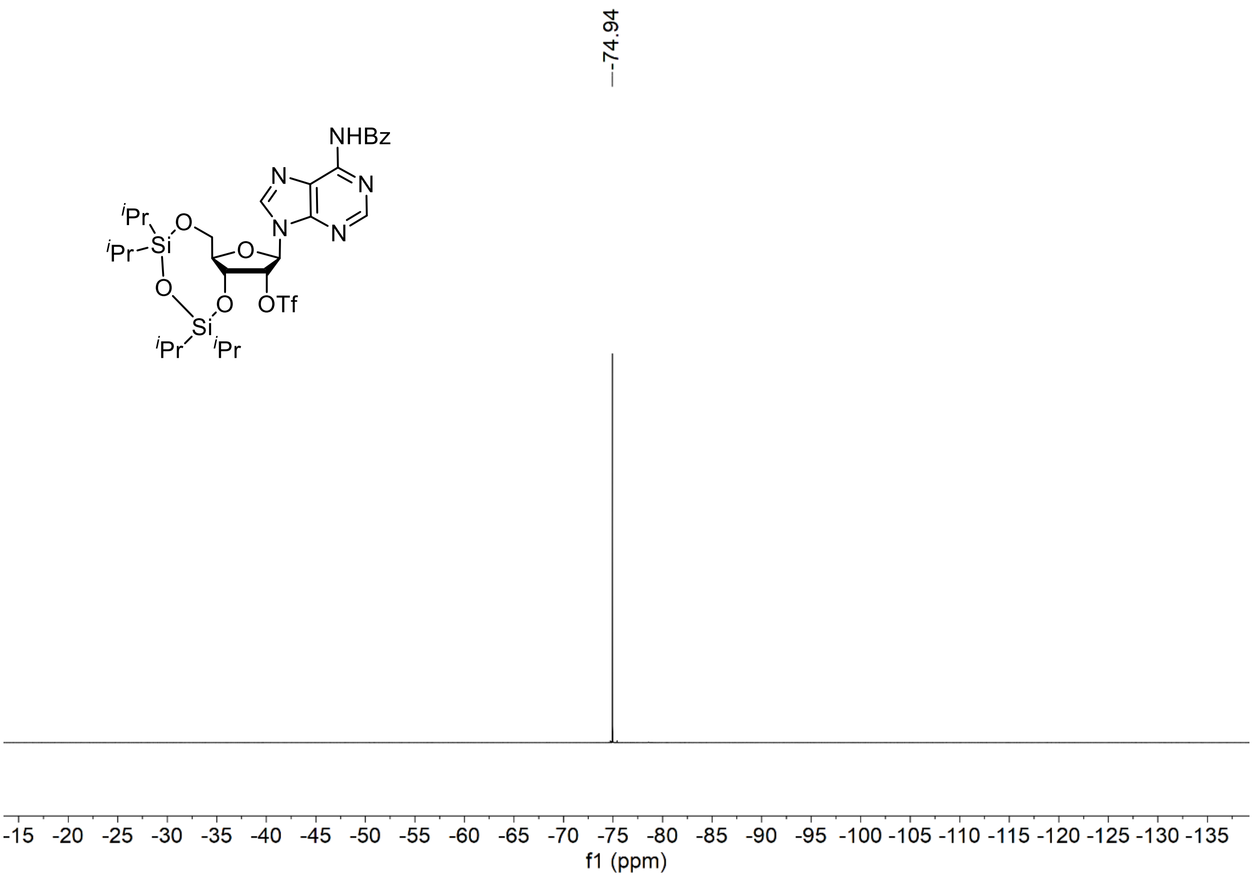

Figure S15. ^{1}H ^{13}C and ^{19}F NMR of (*6aR,8R,9R,9aR*)-8-(6-benzamido-9*H*-purin-9-yl)-2,2,4,4-tetraisopropyltetrahydro-6*H*-furo[3,2-*f*][1,3,5,2,4]trioxadisilocin-9-yl trifluoromethanesulfonate.

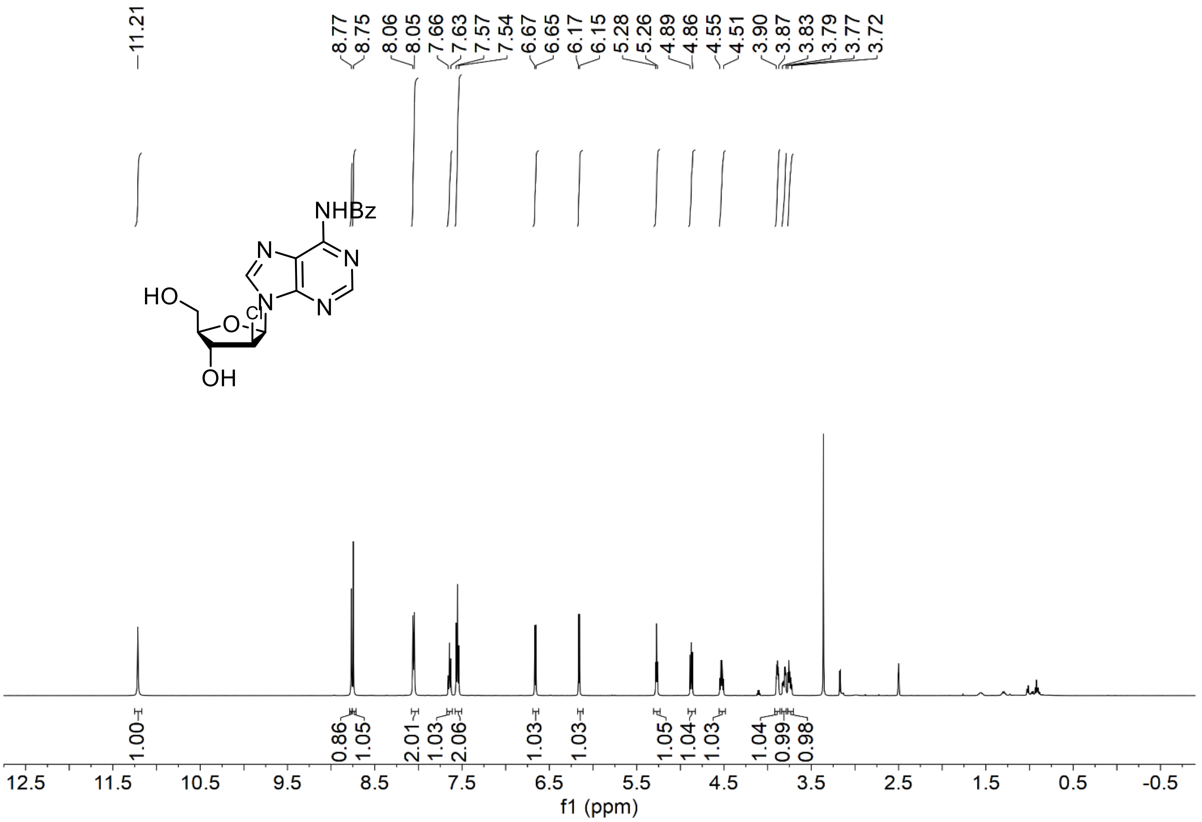

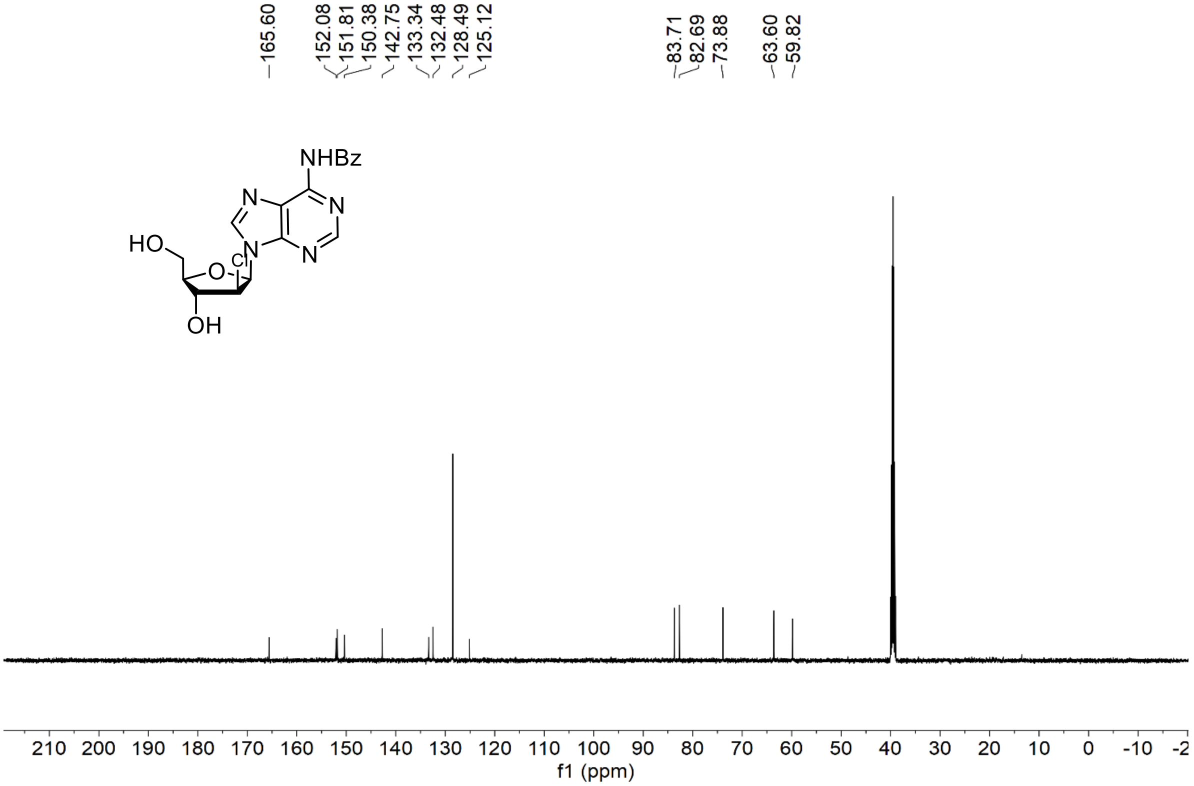

Figure S16. ^{1}H and ^{13}C NMR of N-(9-((2R,3S,4R,5R)-3-chloro-4-hydroxy-5-(hydroxymethyl)tetrahydrofuran-2-yl)-9H-purin-6-yl)benzamide.

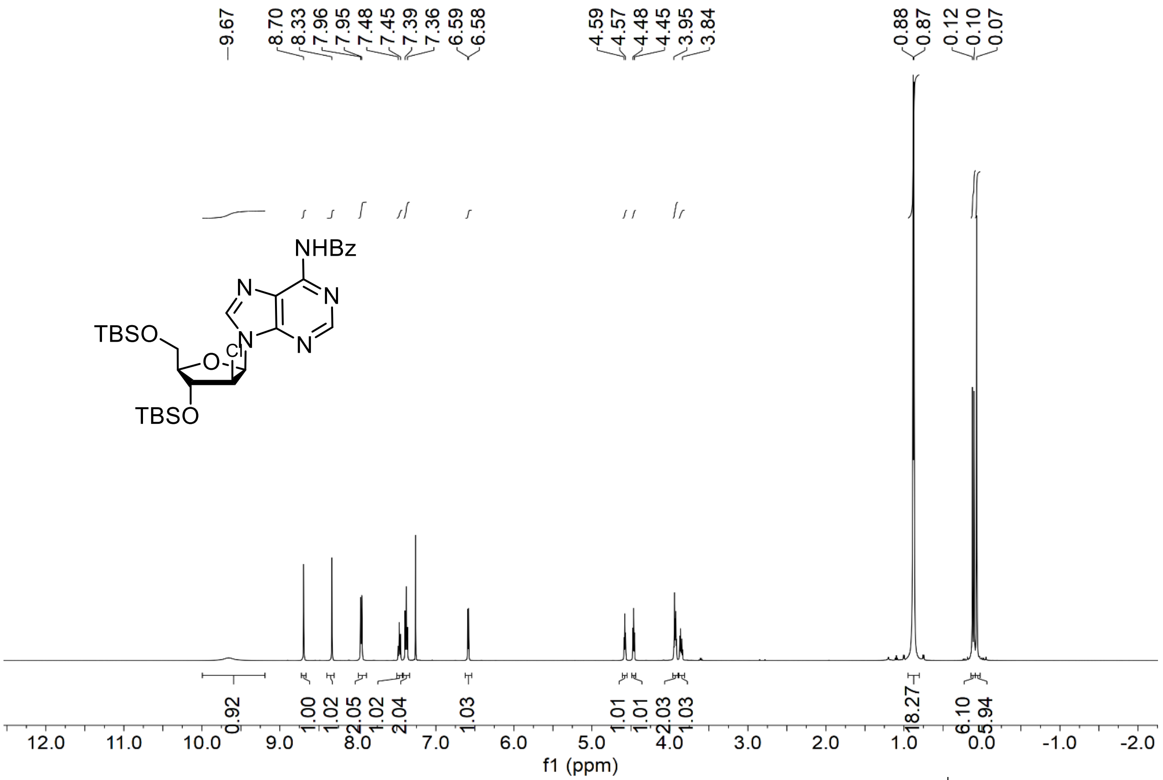

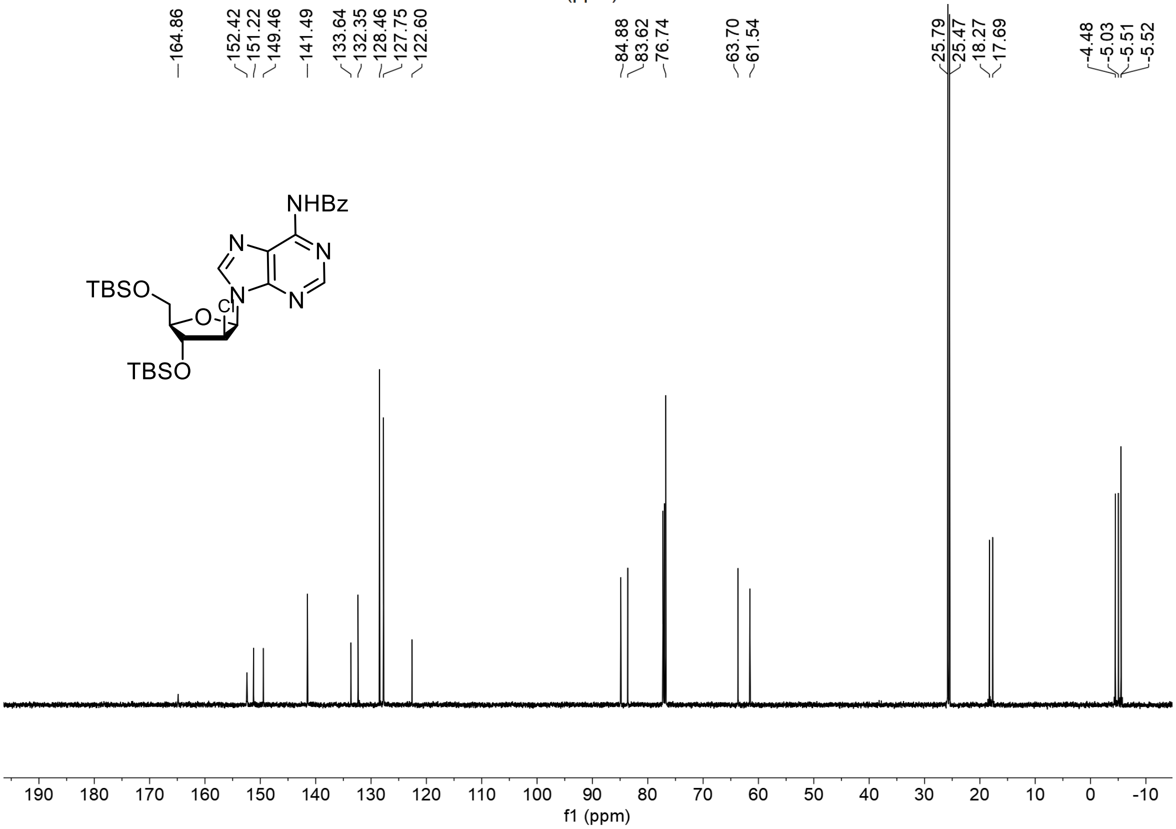

Figure S17. ^{1}H and ^{13}C NMR of N-(9-((2R,3S,4R,5R)-4-((tert-butyldimethylsilyl)oxy)-5-(((tert-butyldimethylsilyl)oxy)methyl)-3-chlorotetrahydrofuran-2-yl)-9H-purin-6-yl)benzamide.

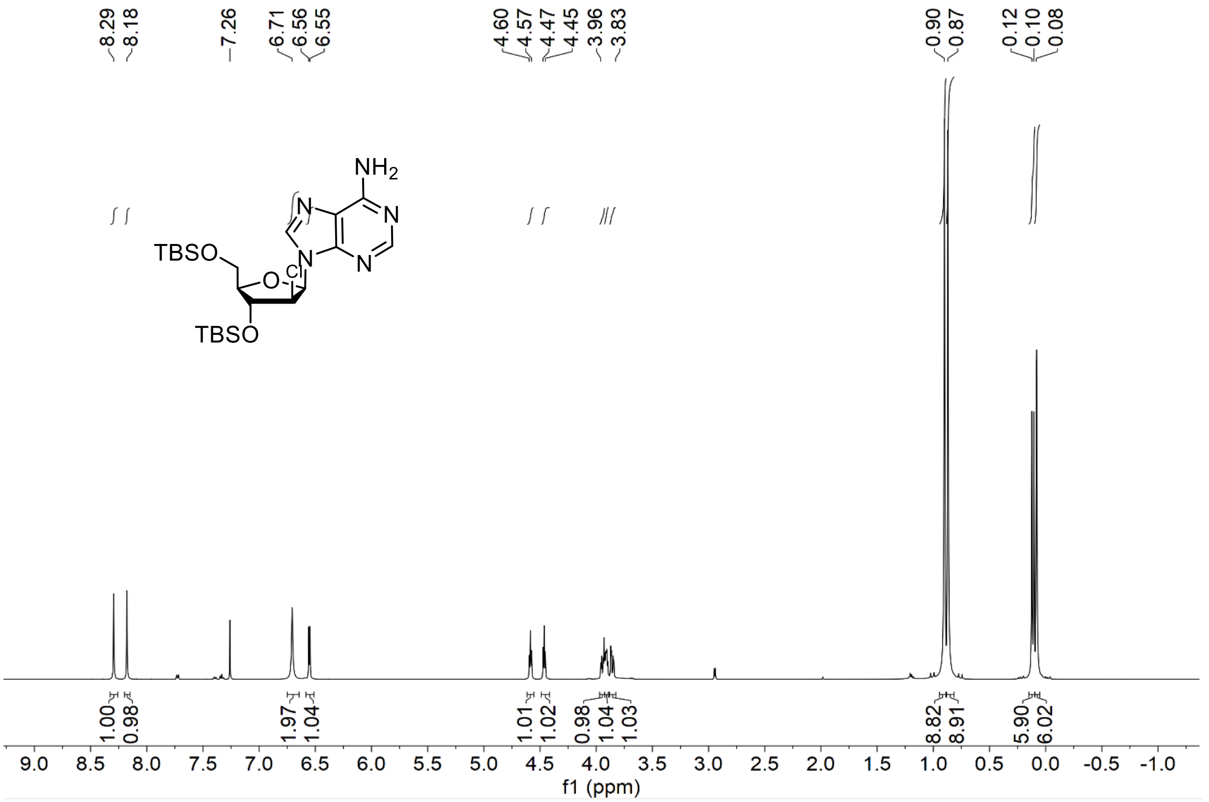

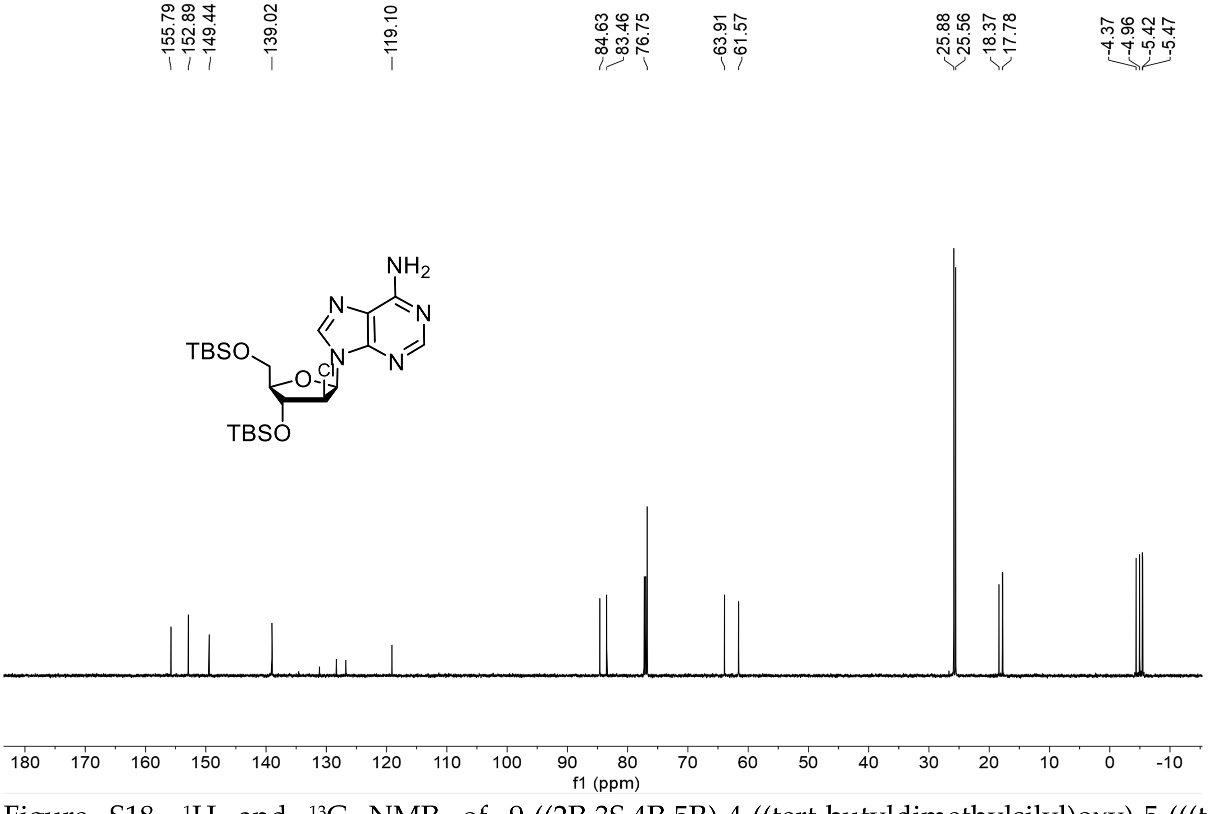

Figure S18. ^1H and ^{13}C NMR of 9-((2R,3S,4R,5R)-4-((tert-butyldimethylsilyl)oxy)-5-(((tert-butyldimethylsilyl)oxy)methyl)-3-chlorotetrahydrofuran-2-yl)-9H-purin-6-amine.

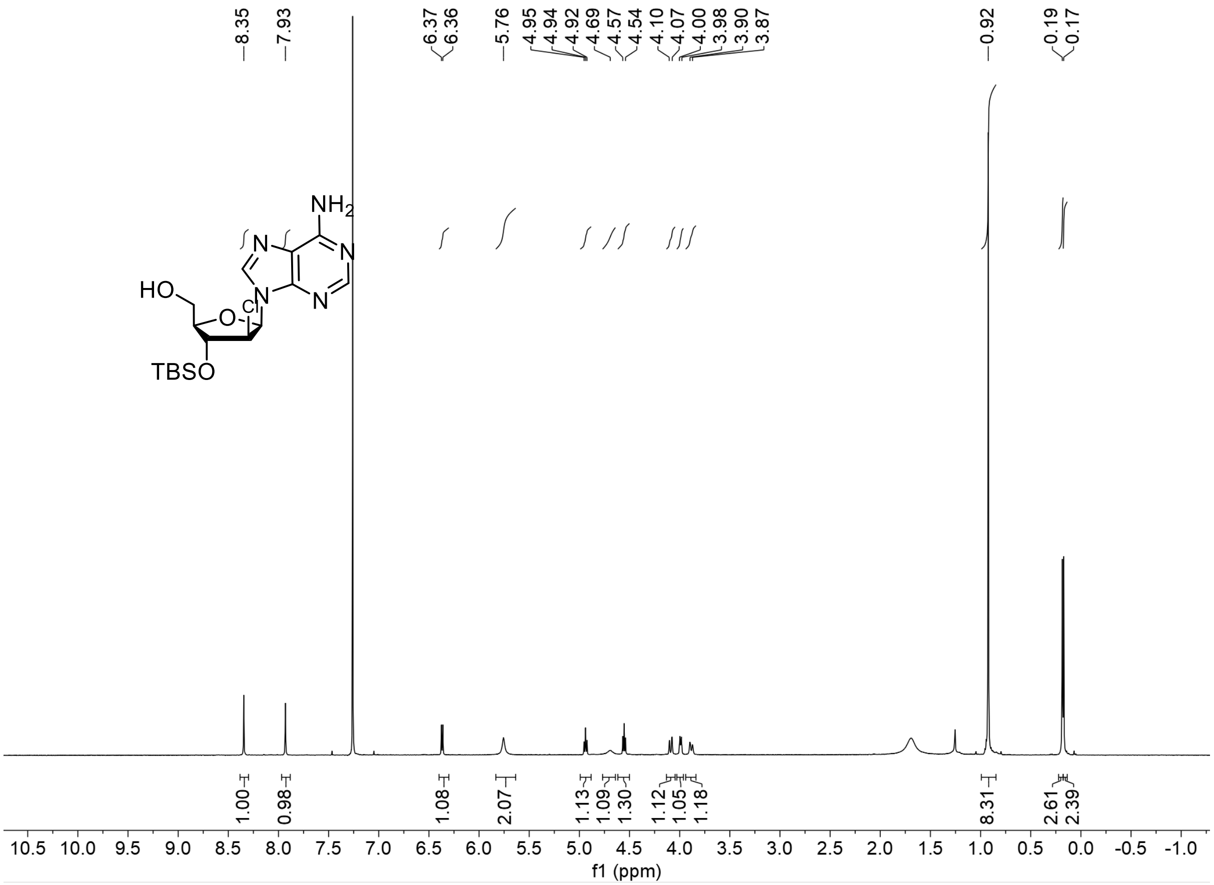

Figure S19. ^{1}H NMR of ((*2R,3R,4S,5R*)-5-(6-amino-9*H*-purin-9-yl)-3-((*tert*-butyldimethylsilyl)oxy)-4-chlorotetrahydrofuran-2-yl)methanol

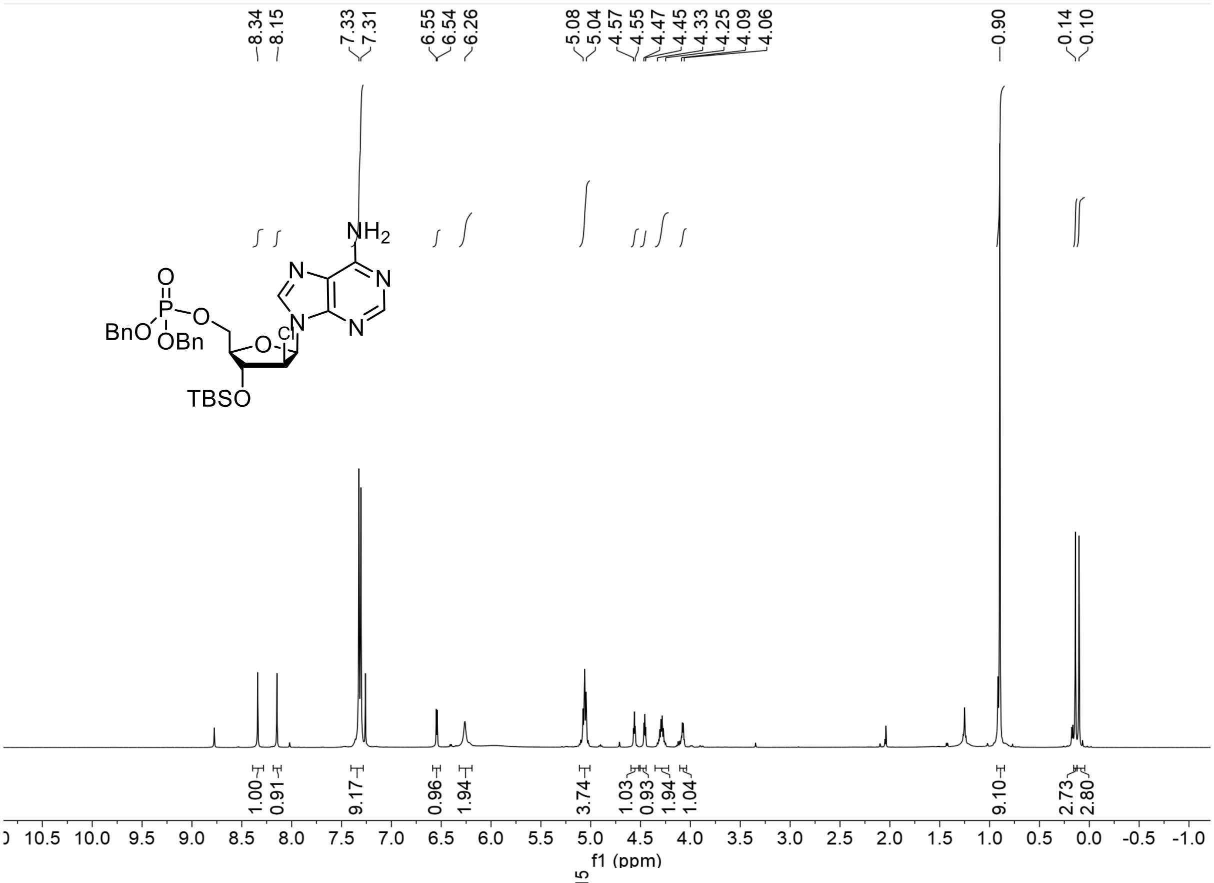

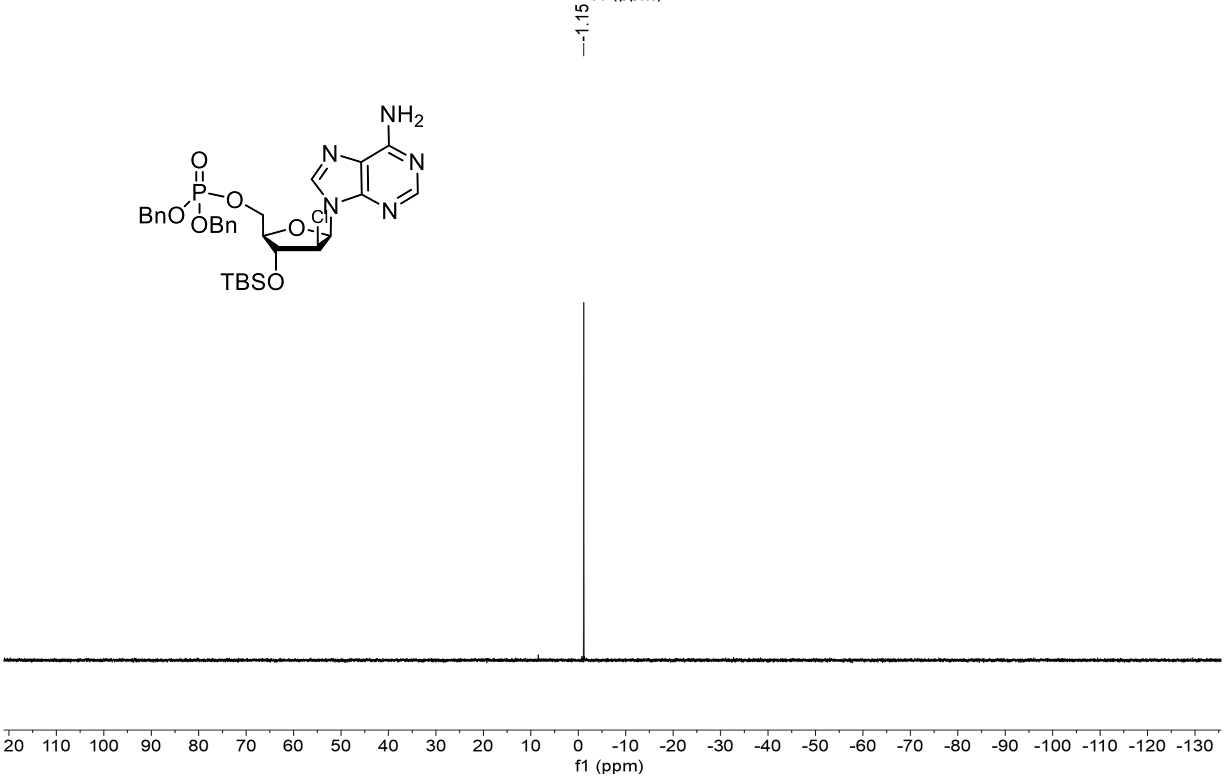

Figure S20. ^{1}H and ^{31}P NMR of ((2R,3R,4S,5R)-5-(6-amino-9H-purin-9-yl)-3-((tert-butyldimethylsilyl)oxy)-4-chlorotetrahydrofuran-2-yl)methanol

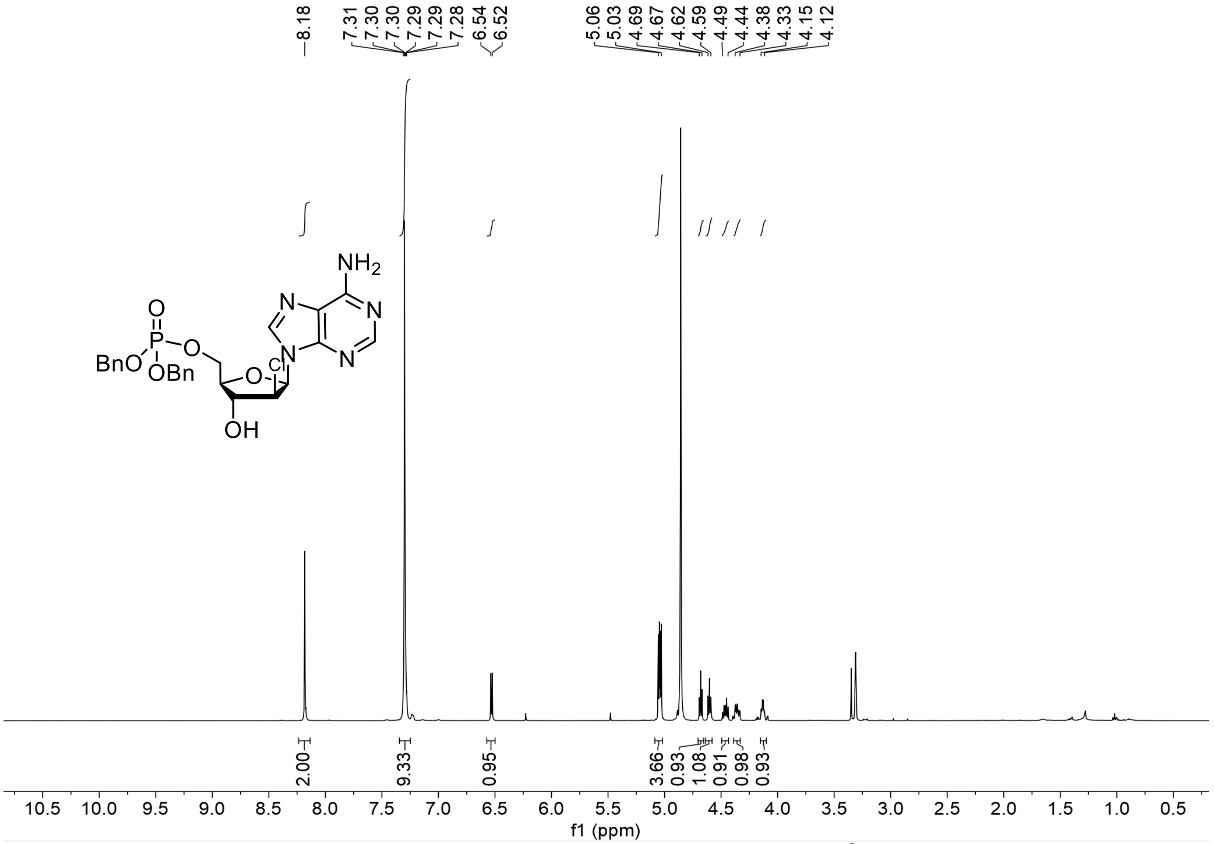
8.18
7.31
7.30
7.30
7.29
7.29
7.28
6.54
6.52
5.06
5.03
4.69
4.67
4.62
4.59
4.49
4.44
4.38
4.33
4.15
4.12
NH2
N
N
N
N
O
P
O
BnO
OBn
O
OH
2.00
9.33
0.95
3.66
0.93
1.08
0.91
0.98
0.93
10.5
10.0
9.5
9.0
8.5
8.0
7.5
7.0
6.5
6.0
5.5
5.0
4.5
4.0
3.5
3.0
2.5
2.0
1.5
1.0
0.5
f1 (ppm)

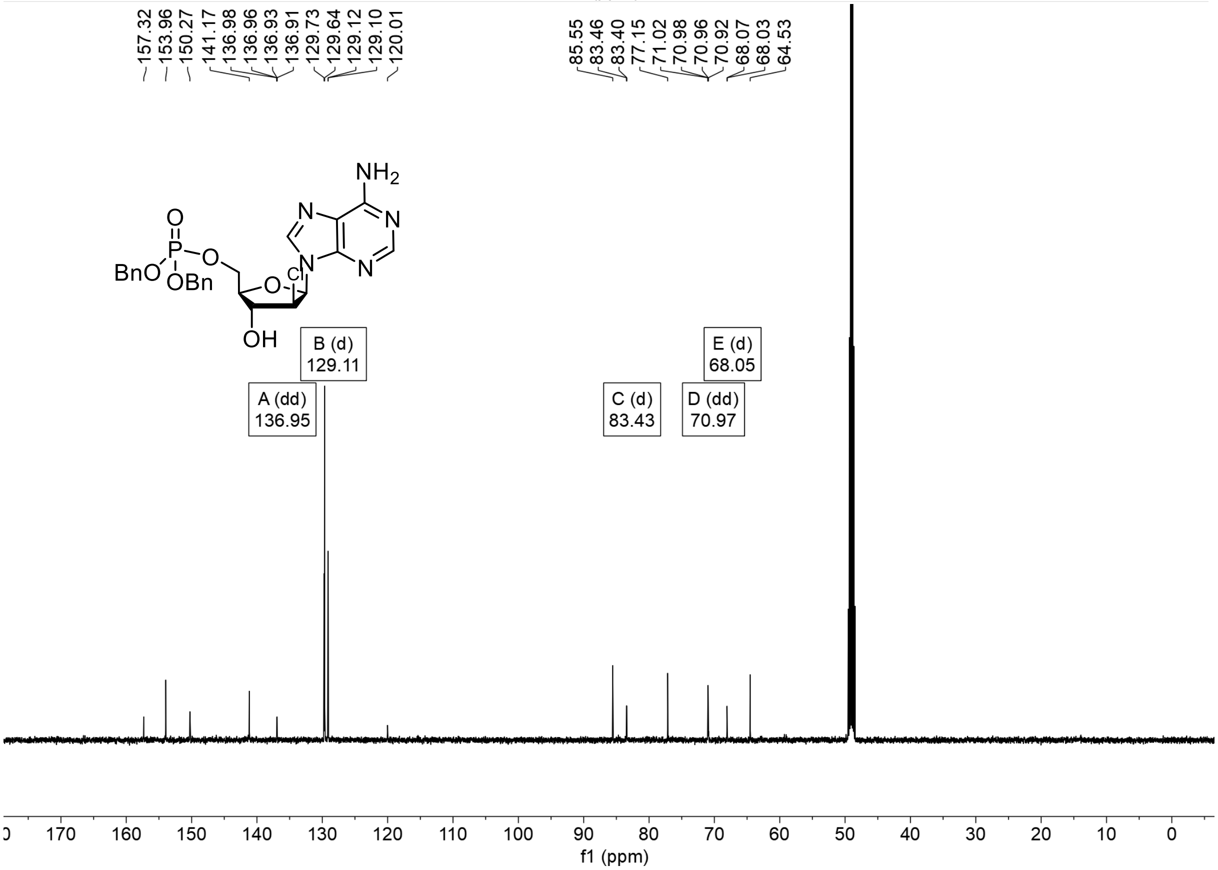
157.32
153.96
150.27
141.17
136.98
136.96
136.93
136.91
129.73
129.64
129.12
129.10
120.01
85.55
83.46
83.40
77.15
71.02
70.98
70.96
70.92
68.07
68.03
64.53
NH2
N
N
N
N
O
P
O
BnO
OBn
O
OH
B (d)
129.11
A (dd)
136.95
C (d)
83.43
D (dd)
70.97
E (d)
68.05
170
160
150
140
130
120
110
100
90
80
70
60
50
40
30
20
10
0
f1 (ppm)

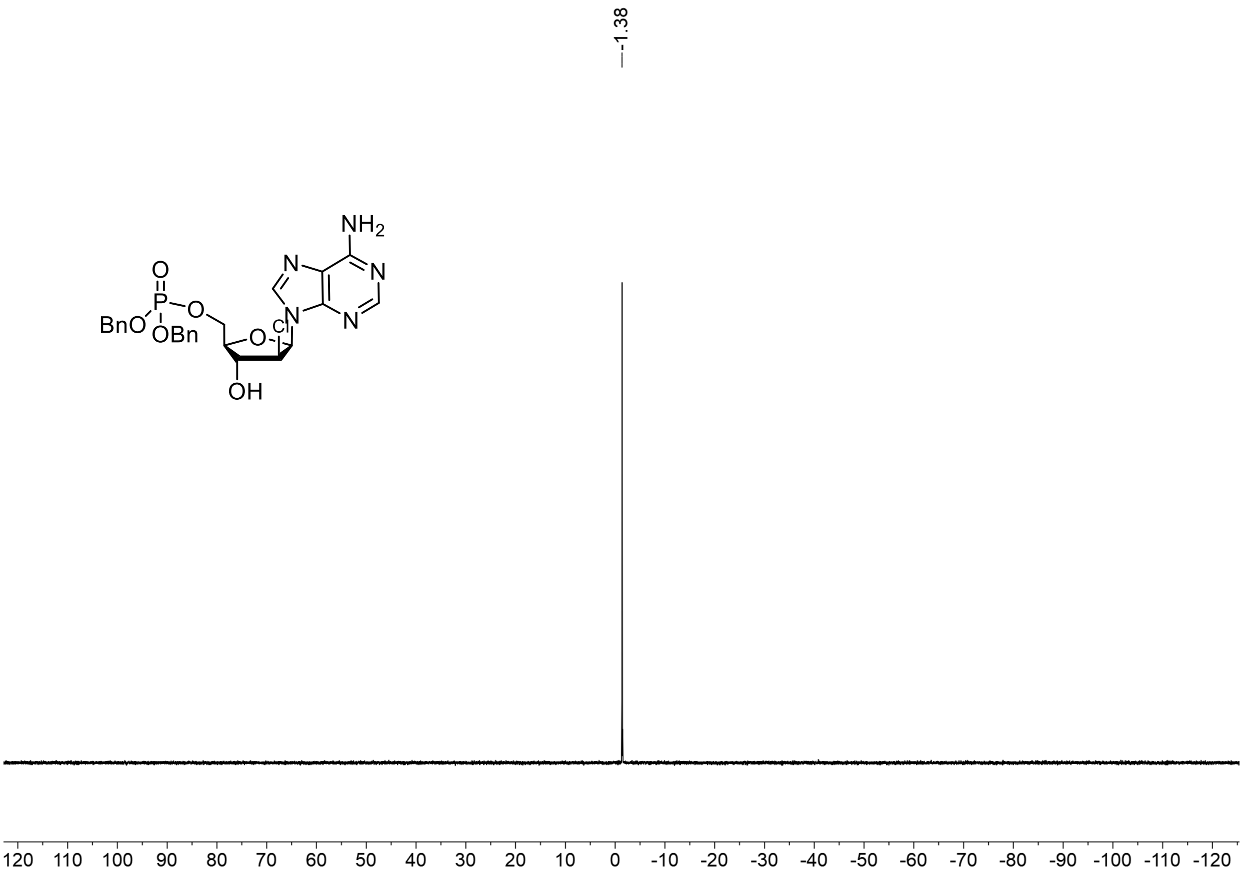

Figure S21. ^{1}H ^{13}C and ^{31}P NMR of ((*2R,3R,4S,5R*)-5-(6-amino-9*H*-purin-9-yl)-4-chloro-3-hydroxy-tetrahydrofuran-2-yl)methyl dibenzyl phosphate.

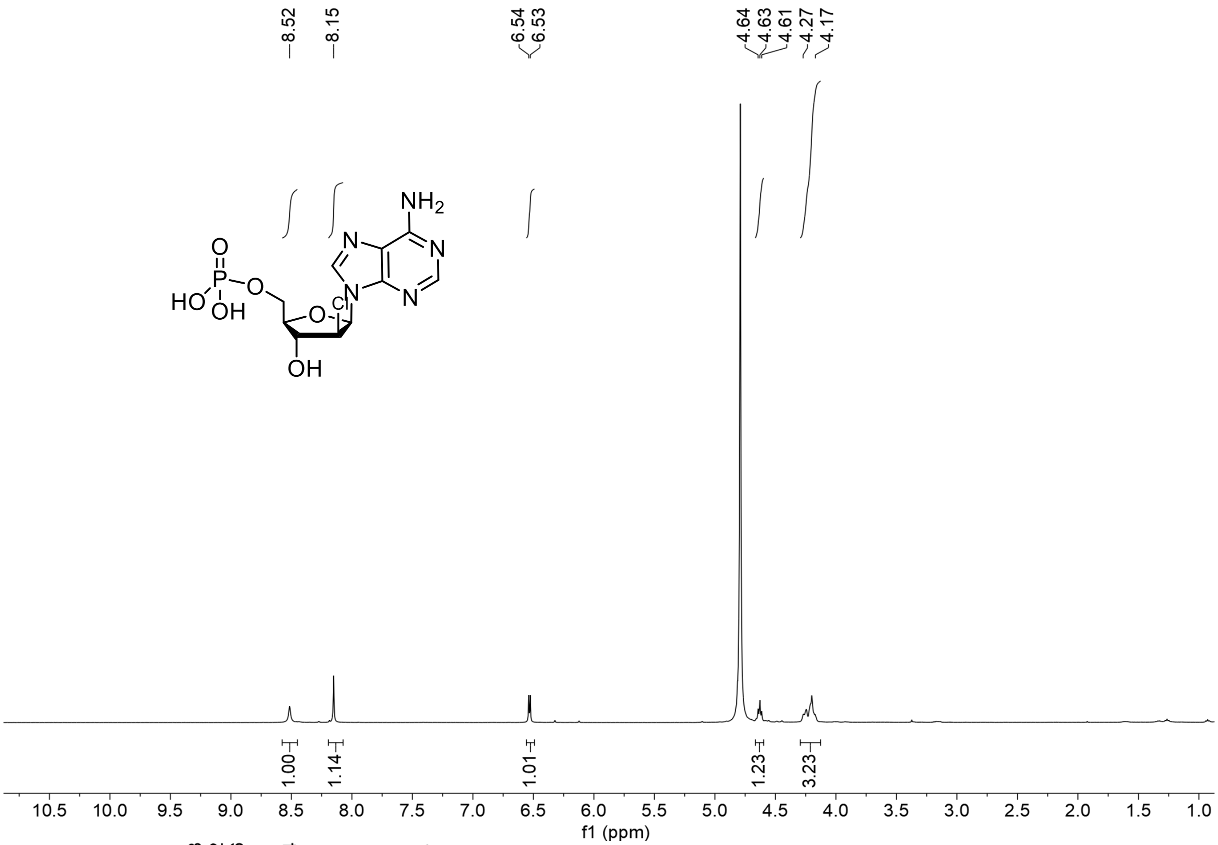
8.52
8.15
6.54
6.53
4.64
4.63
4.61
4.27
4.17
NH2
N
O
P
O
HO
OH
OH
Cl
1.00
1.14
1.01
1.23
3.23
f1 (ppm)

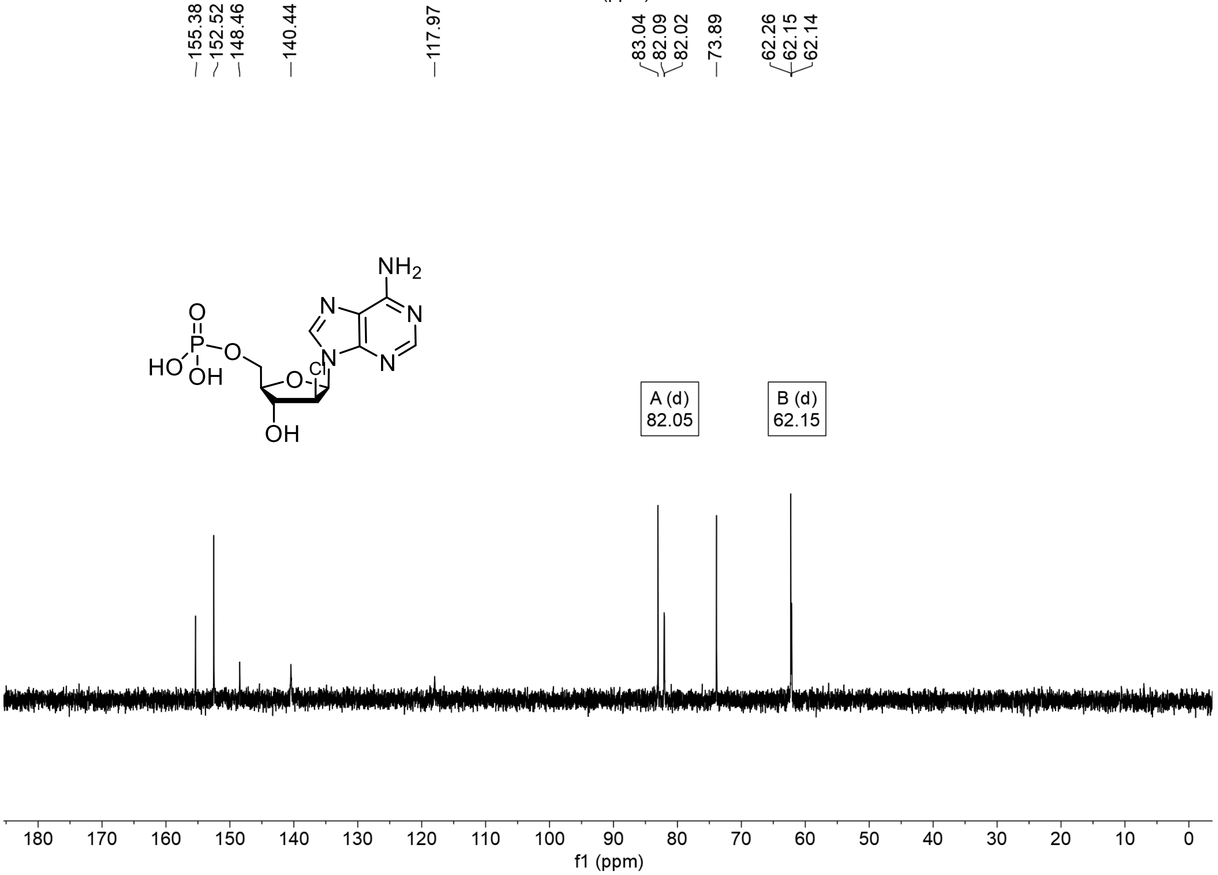
155.38
152.52
148.46
140.44
117.97
83.04
82.09
82.02
73.89
62.26
62.15
62.14
A (d)
82.05
B (d)
62.15
NH2
HO
OH
OH
Cl
f1 (ppm)

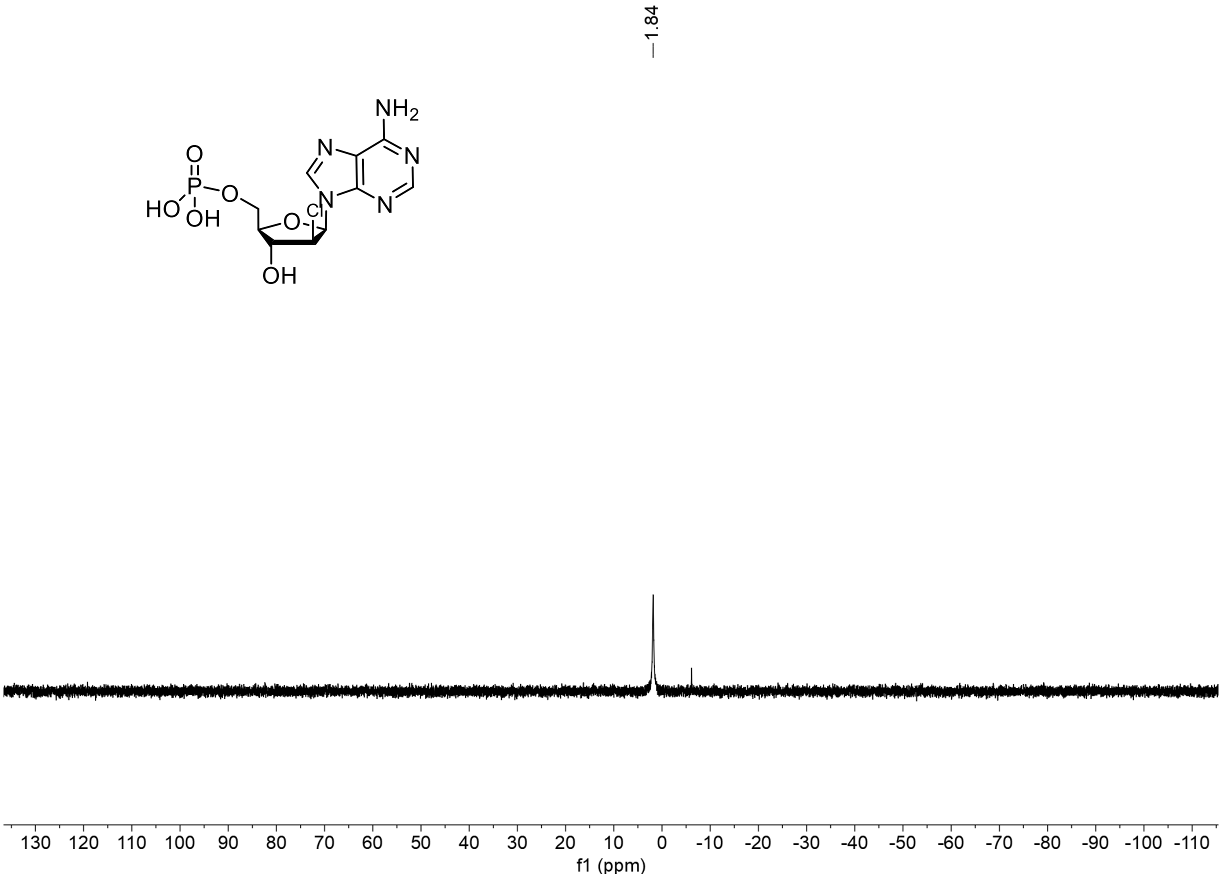

Figure S22. ^{1}H, ^{13}C and ^{31}P NMR of ((*2R,3R,4S,5R*)-5-(6-amino-9*H*-purin-9-yl)-4-chloro-3-hydroxytetrahydro-furan-2-yl)methyl dihydrogen phosphate.

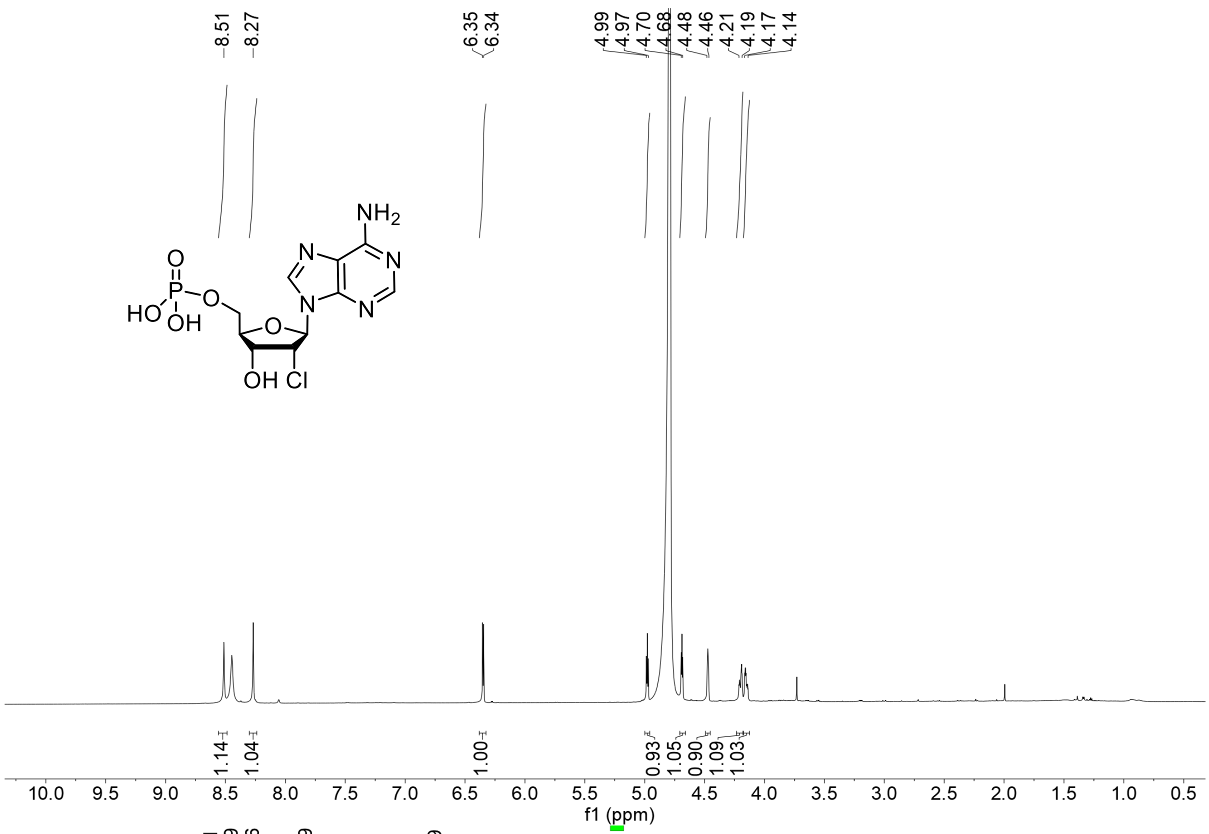

8.51
8.27
6.35
6.34
4.99
4.97
4.70
4.68
4.48
4.46
4.21
4.19
4.17
4.14
NH2
N
N
N
N
O
P
HO
OH
O
O
OH
Cl
1.14
1.04
1.00
0.93
1.05
0.90
1.09
1.03
10.0 9.5 9.0 8.5 8.0 7.5 7.0 6.5 6.0 5.5 5.0 4.5 4.0 3.5 3.0 2.5 2.0 1.5 1.0 0.5
f1 (ppm)

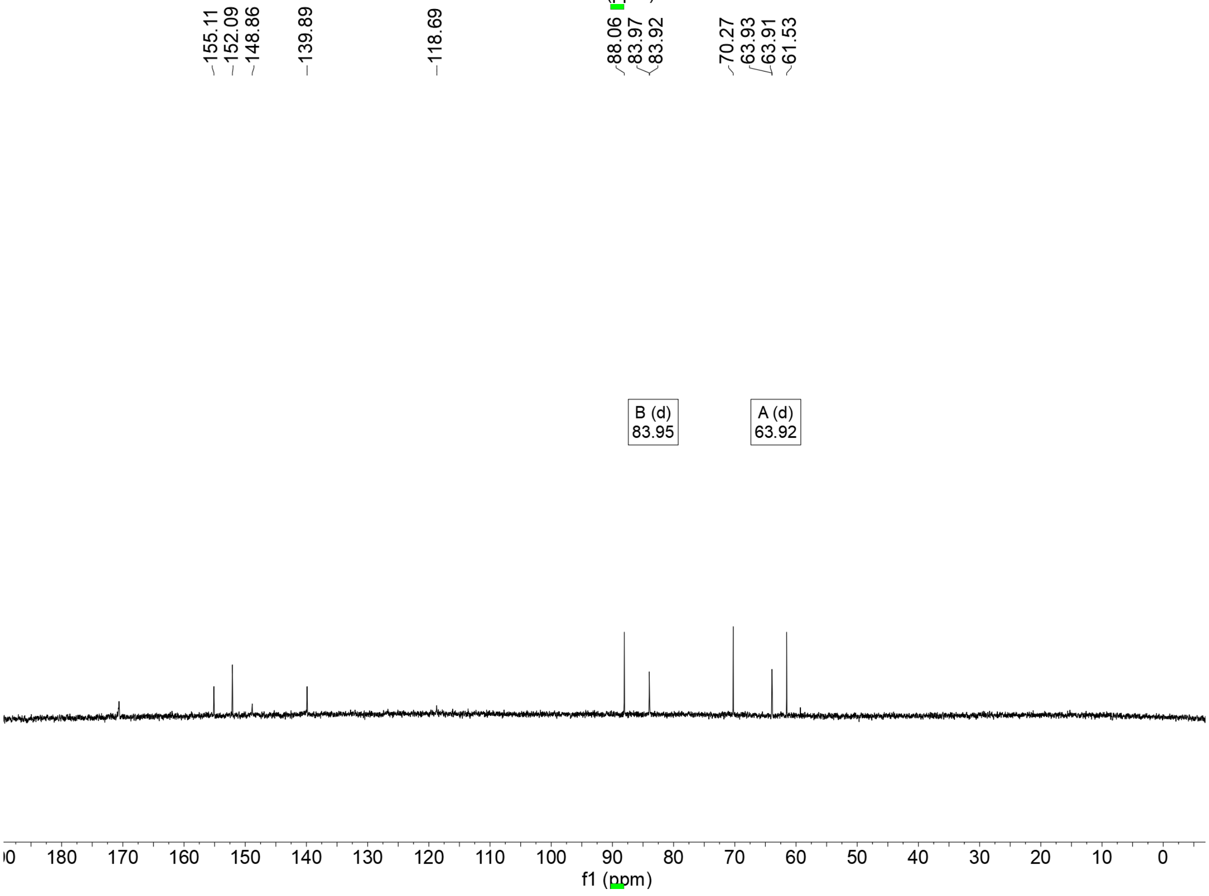

155.11
152.09
148.86
139.89
118.69
88.06
83.97
83.92
70.27
63.93
63.91
61.53
B (d)
83.95
A (d)
63.92
180 170 160 150 140 130 120 110 100 90 80 70 60 50 40 30 20 10 0
f1 (ppm)

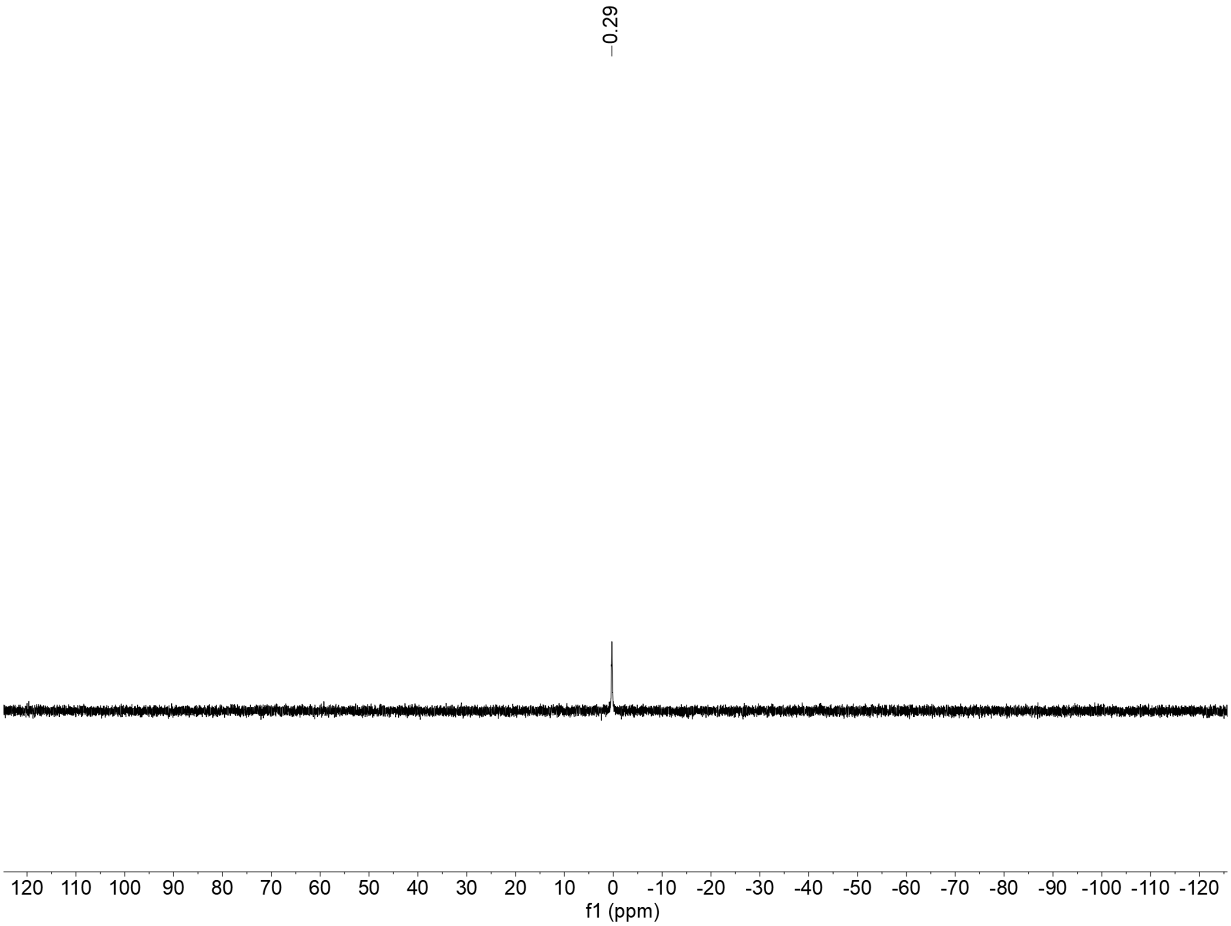

Figure S23. ^{1}H, ^{13}C and ^{31}P NMR of enzymatic product ((*2R,3R,4R,5R*)-5-(6-amino-9*H*-purin-9-yl)-4-chloro-3-hydroxytetra-hydrofuran-2-yl)methyl dihydrogen phosphate.

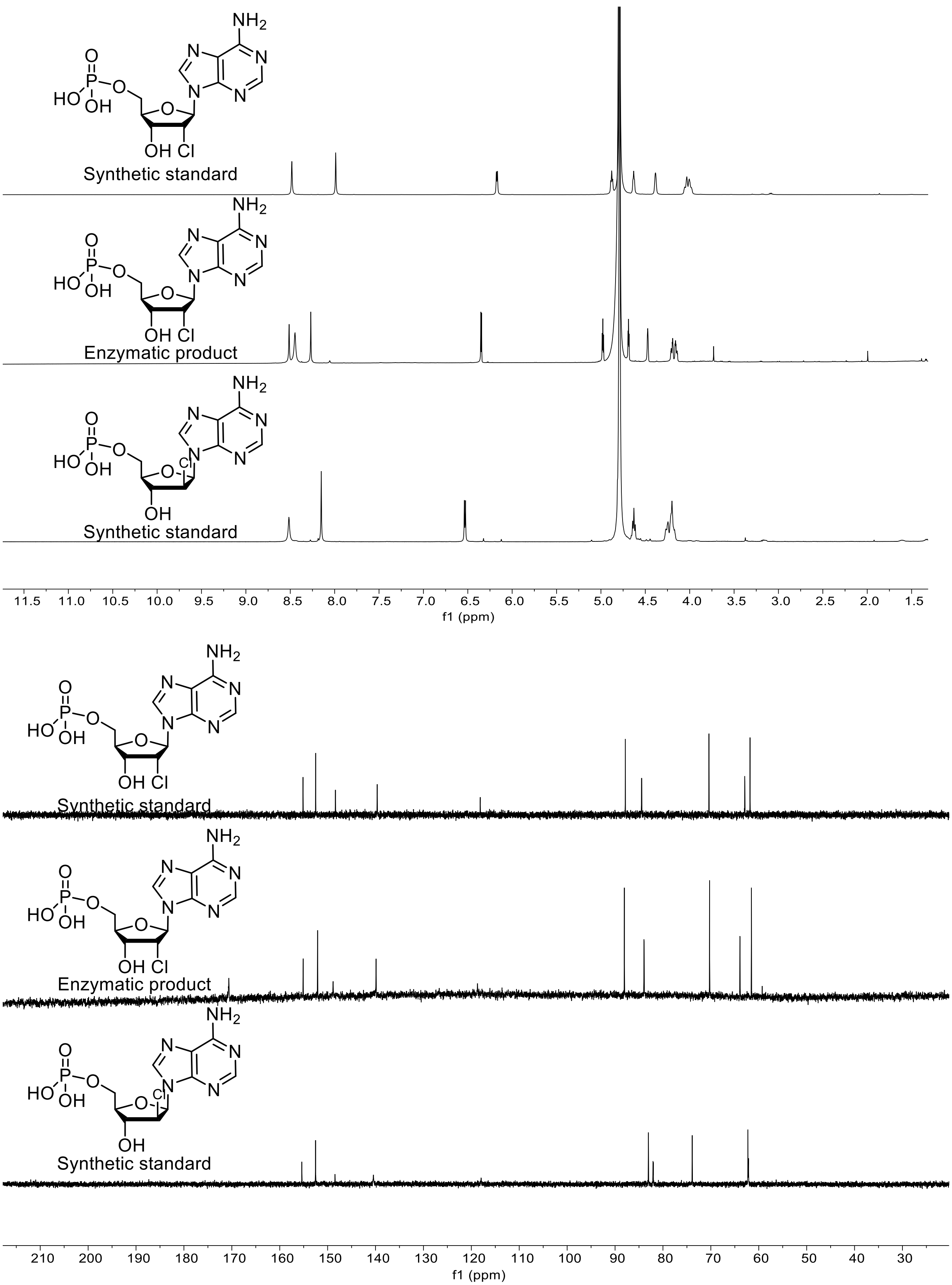

Figure S24. ^{1}H and ^{13}C NMR of enzymatic product and synthetic standard ((2R,3R,4R,5R)-5-(6-amino-9H-purin-9-yl)-4-chloro-3-hydroxytetra-hydrofuran-2-yl)methyl dihydrogen phosphate.

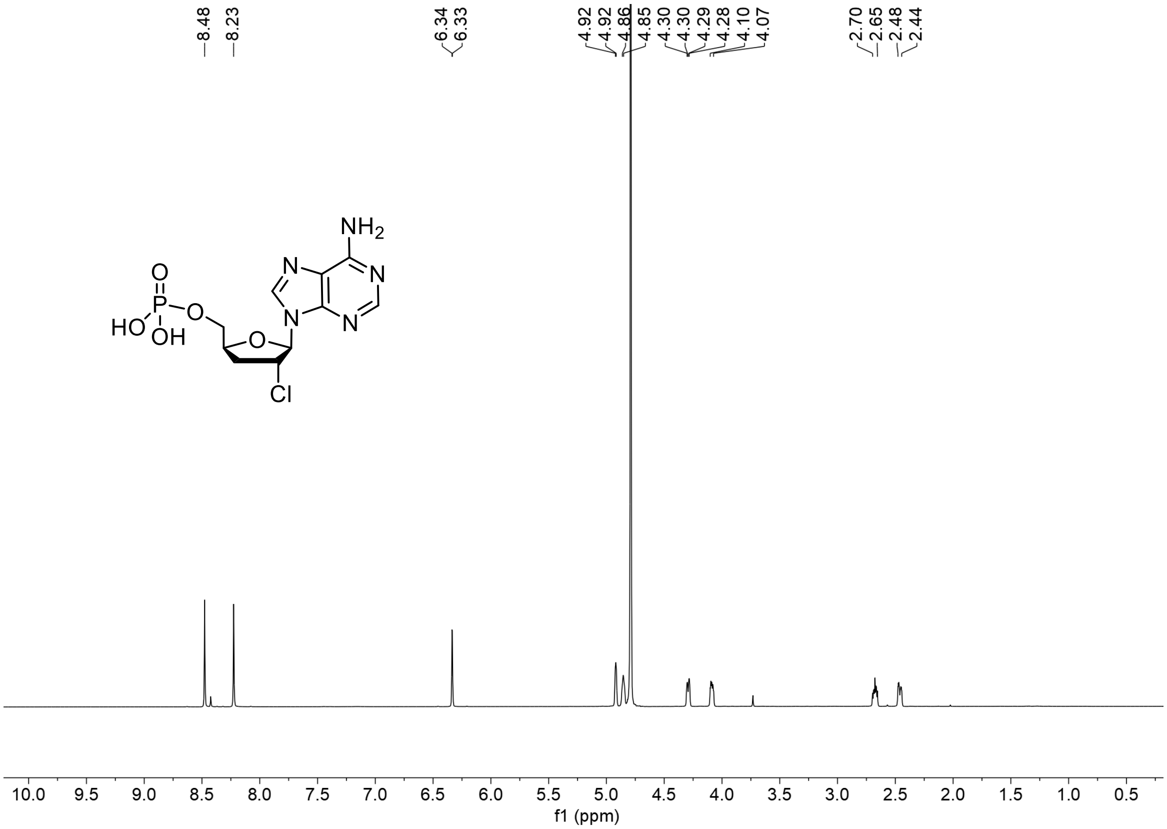
8.48
8.23
6.34
6.33
4.92
4.92
4.86
4.85
4.30
4.30
4.29
4.28
4.10
4.07
2.70
2.65
2.48
2.44
NH2
N
N
N
N
O
P
O
HO
OH
O
Cl
f1 (ppm)

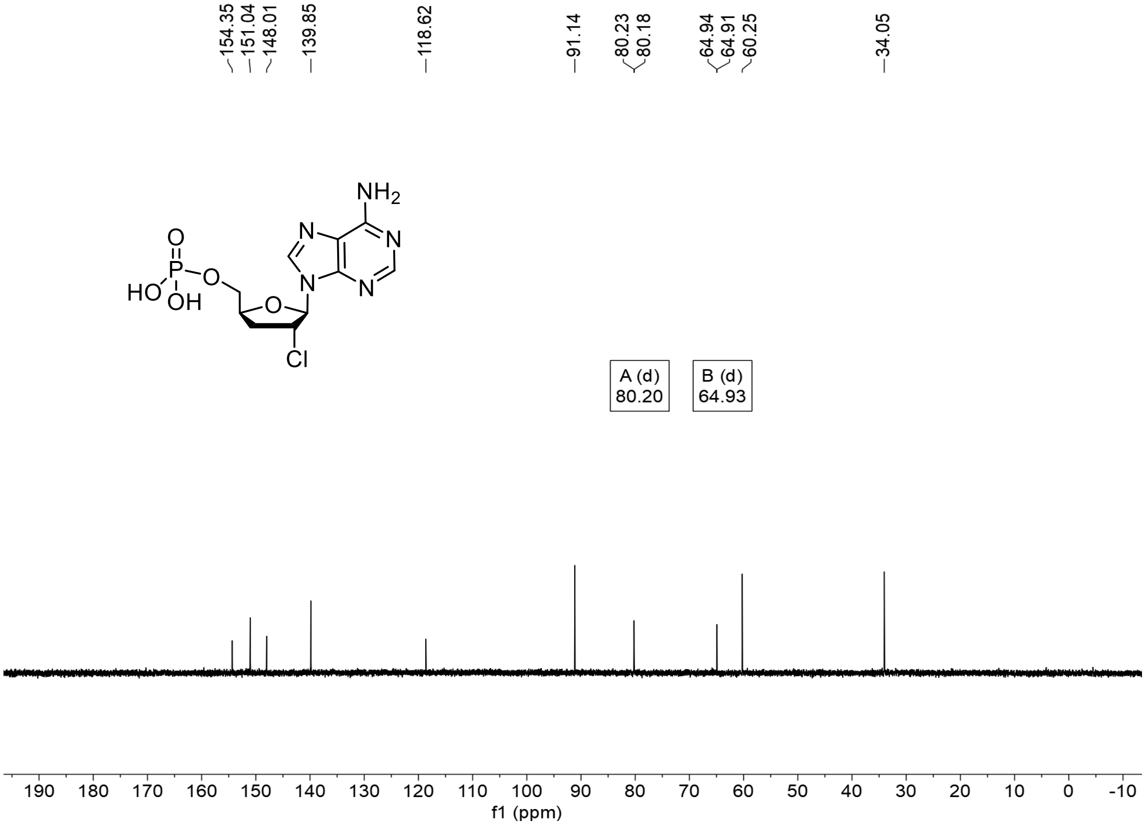
154.35
151.04
148.01
139.85
118.62
91.14
80.23
80.18
64.94
64.91
60.25
34.05
NH2
N
N
N
N
O
P
O
HO
OH
O
Cl
A (d)
80.20
B (d)
64.93
f1 (ppm)

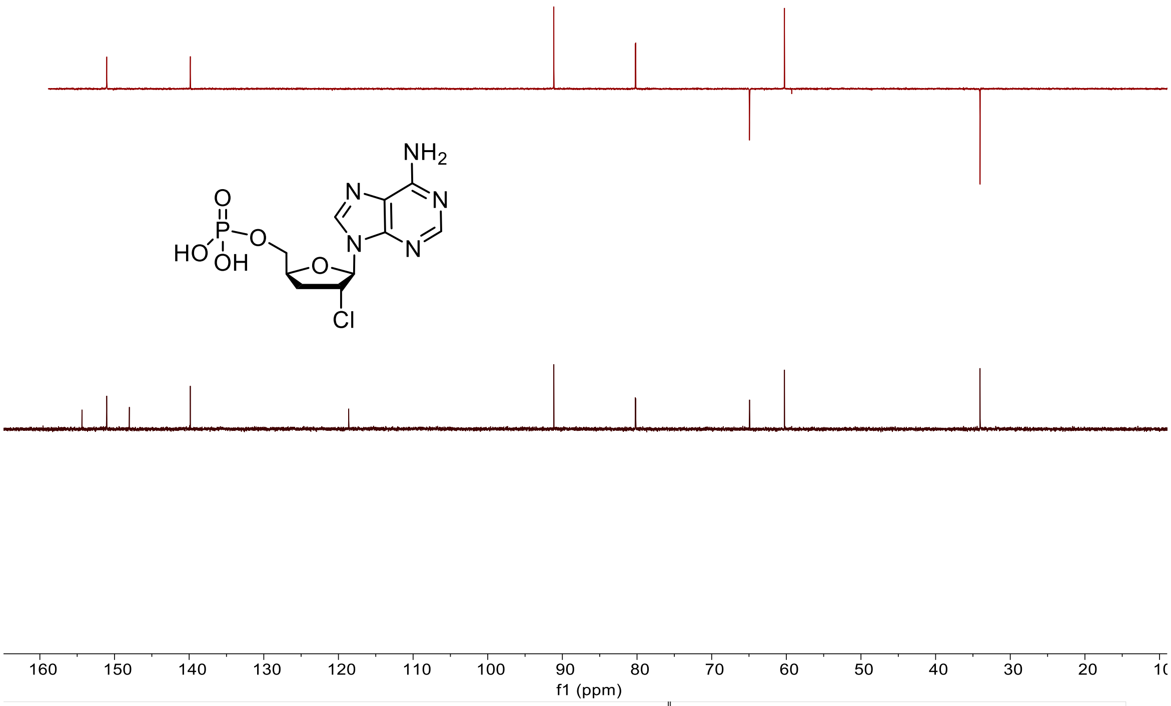
NH2
N
N
N
N
O
P
O
HO
OH
O
Cl
160
150
140
130
120
110
100
90
80
70
60
50
40
30
20
f1 (ppm)

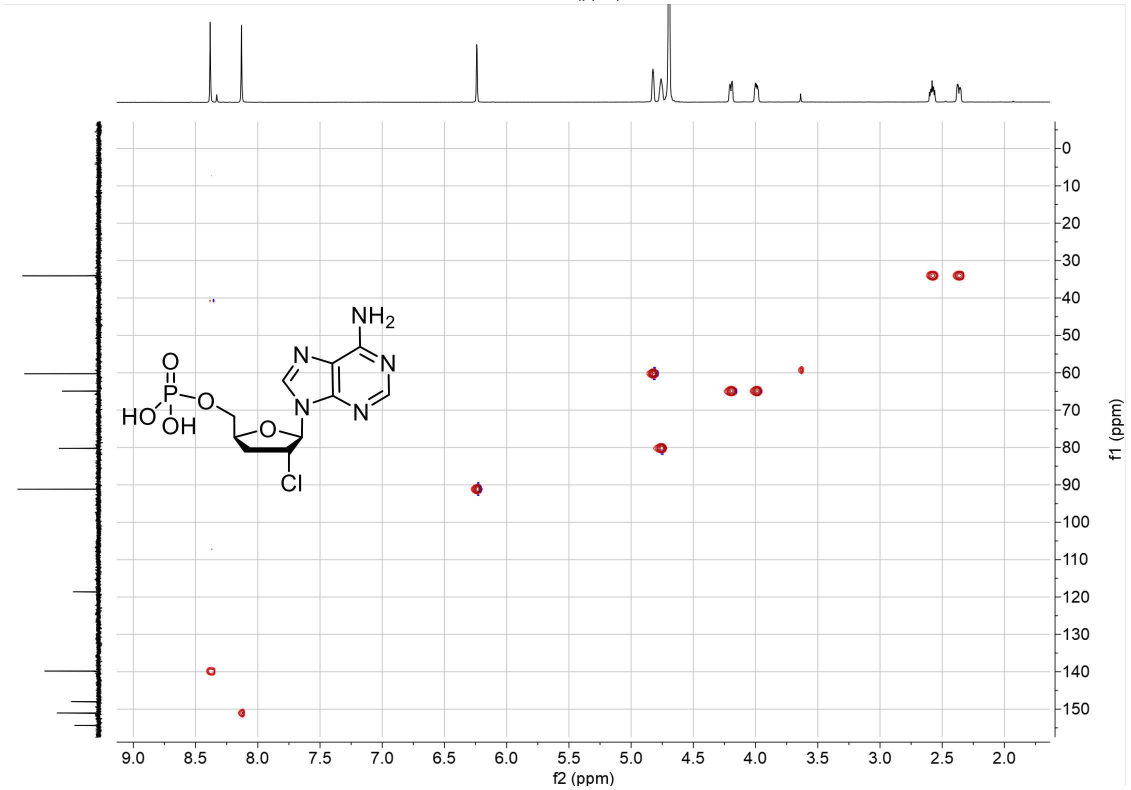
NH2
N
N
N
N
O
P
O
HO
OH
O
Cl
0
10
20
30
40
50
60
70
80
90
100
110
120
130
140
150
f1 (ppm)
9.0
8.5
8.0
7.5
7.0
6.5
6.0
5.5
5.0
4.5
4.0
3.5
3.0
2.5
2.0
f2 (ppm)

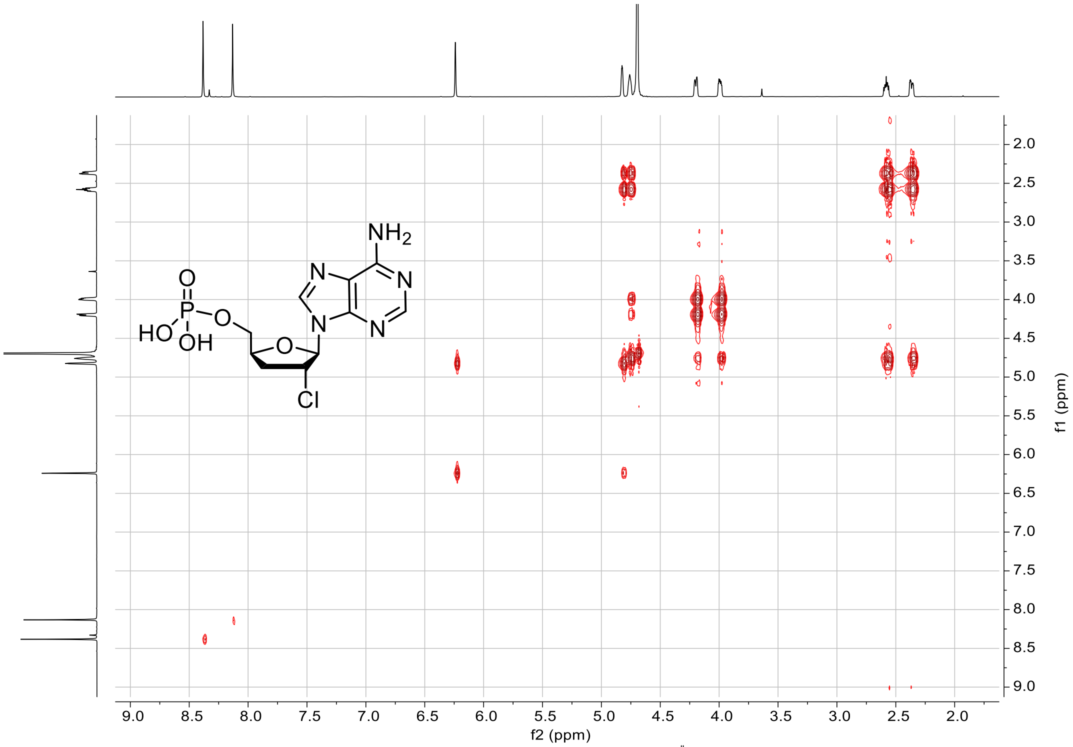

f2 (ppm)
f1 (ppm)
NH2
HO
OH
Cl

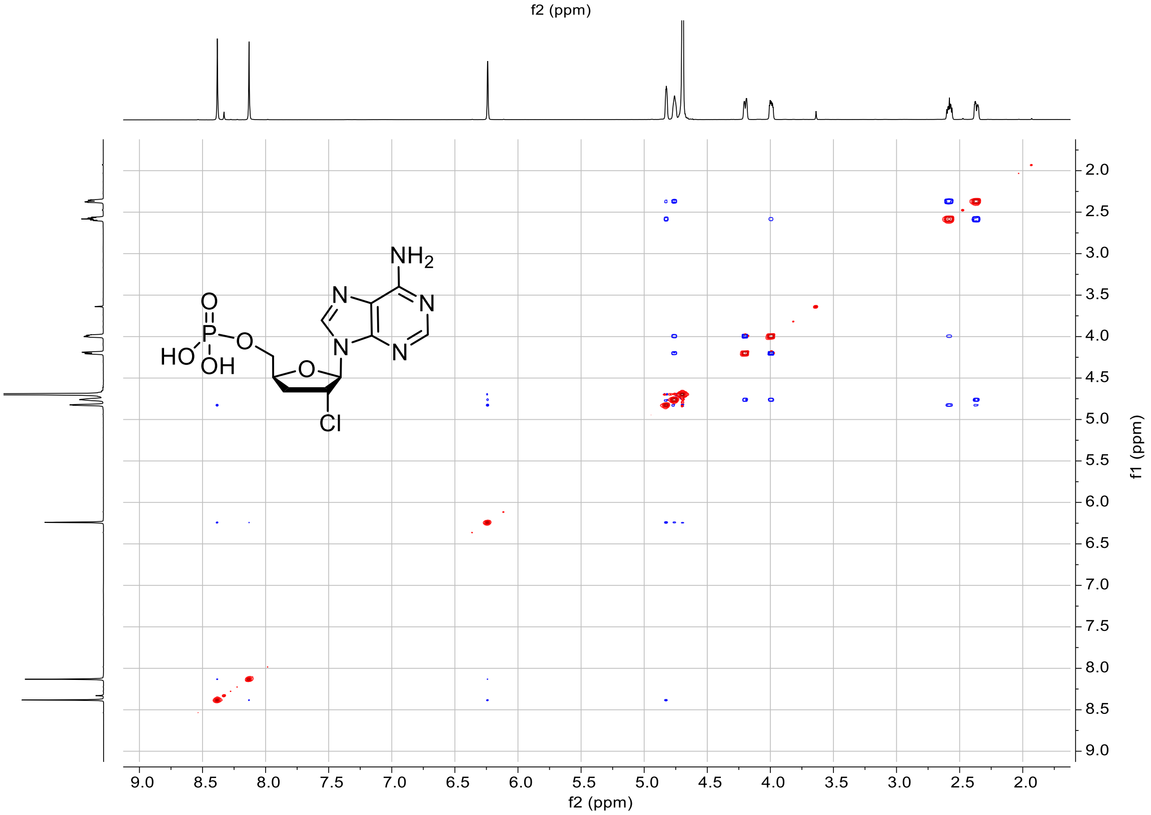

f2 (ppm)
f1 (ppm)
NH2
HO
OH
Cl

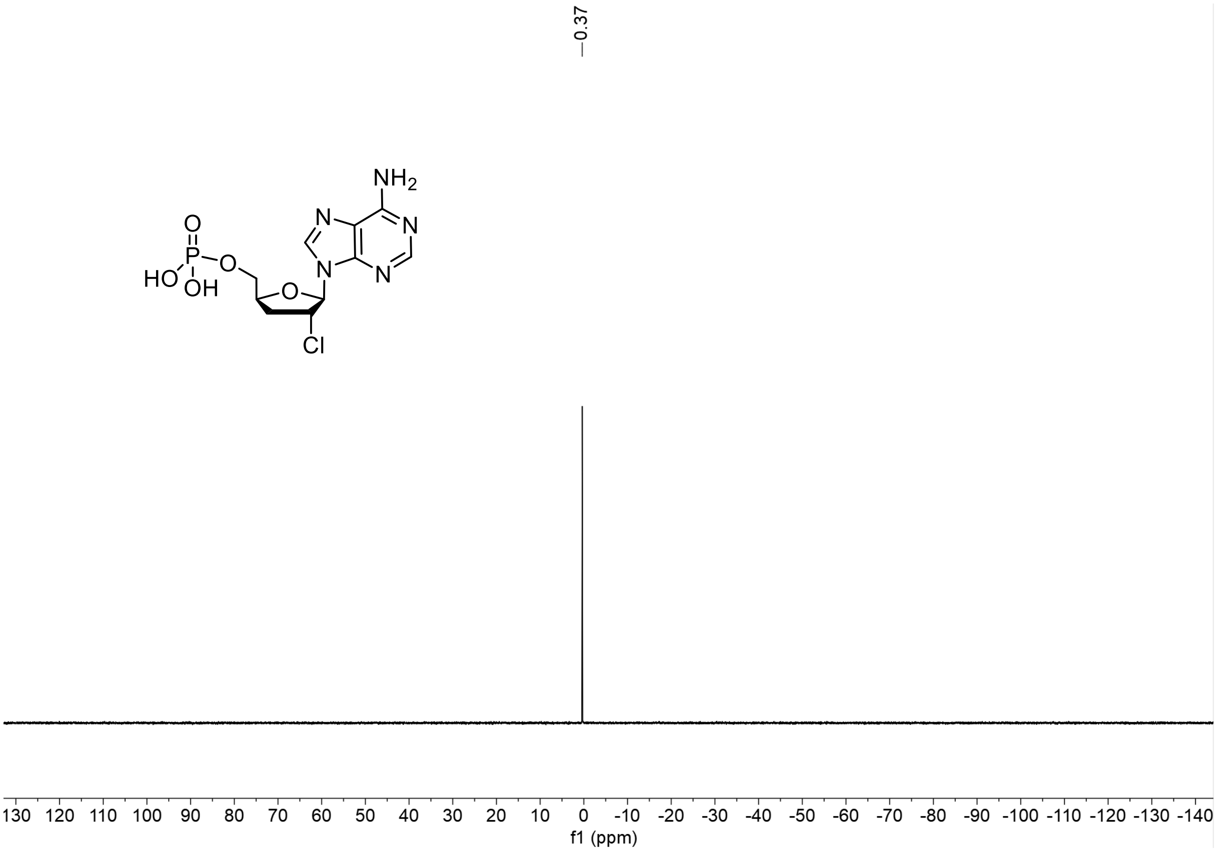

Figure S25. ^{1}H, ^{13}C, DEPT135, HSQC, COSY, NOESY and 31P NMR of enzymatic product ((2S,4R,5R)-5-(6-amino-9H-purin-9-yl)-4-chlorotetrahydrofuran-2-yl)methyl dihydrogen phosphate.

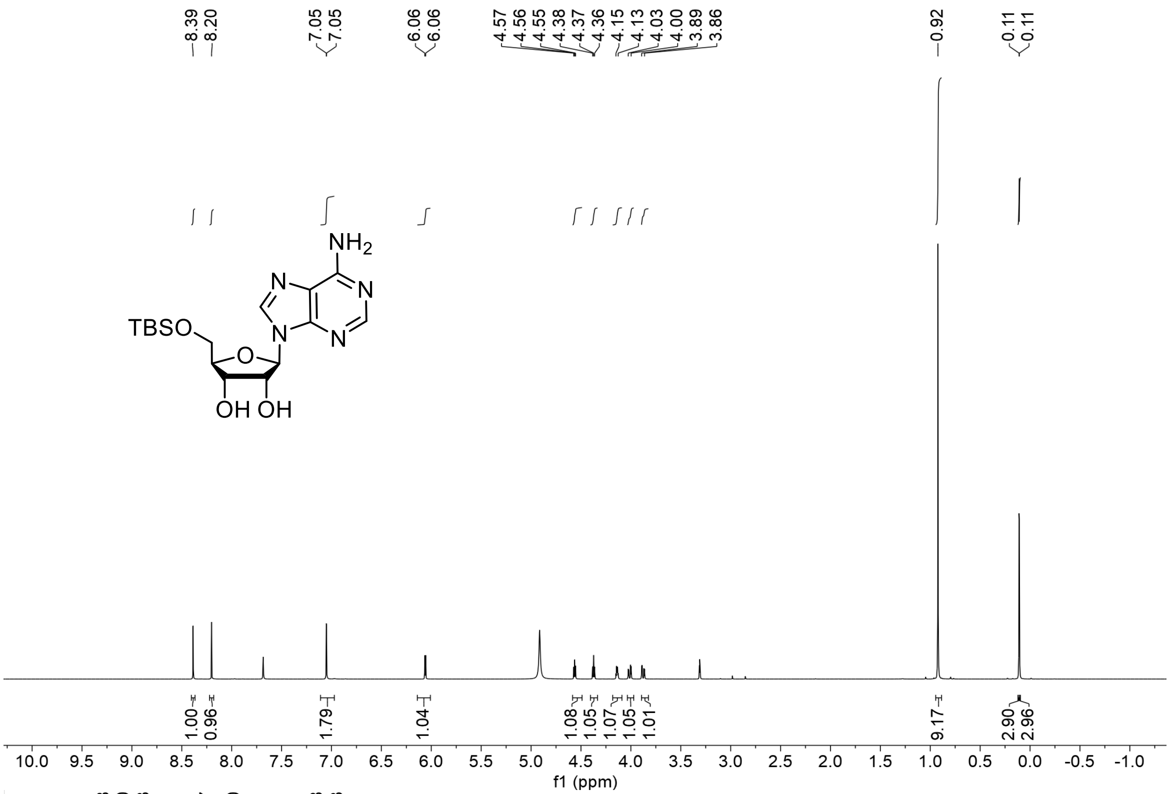

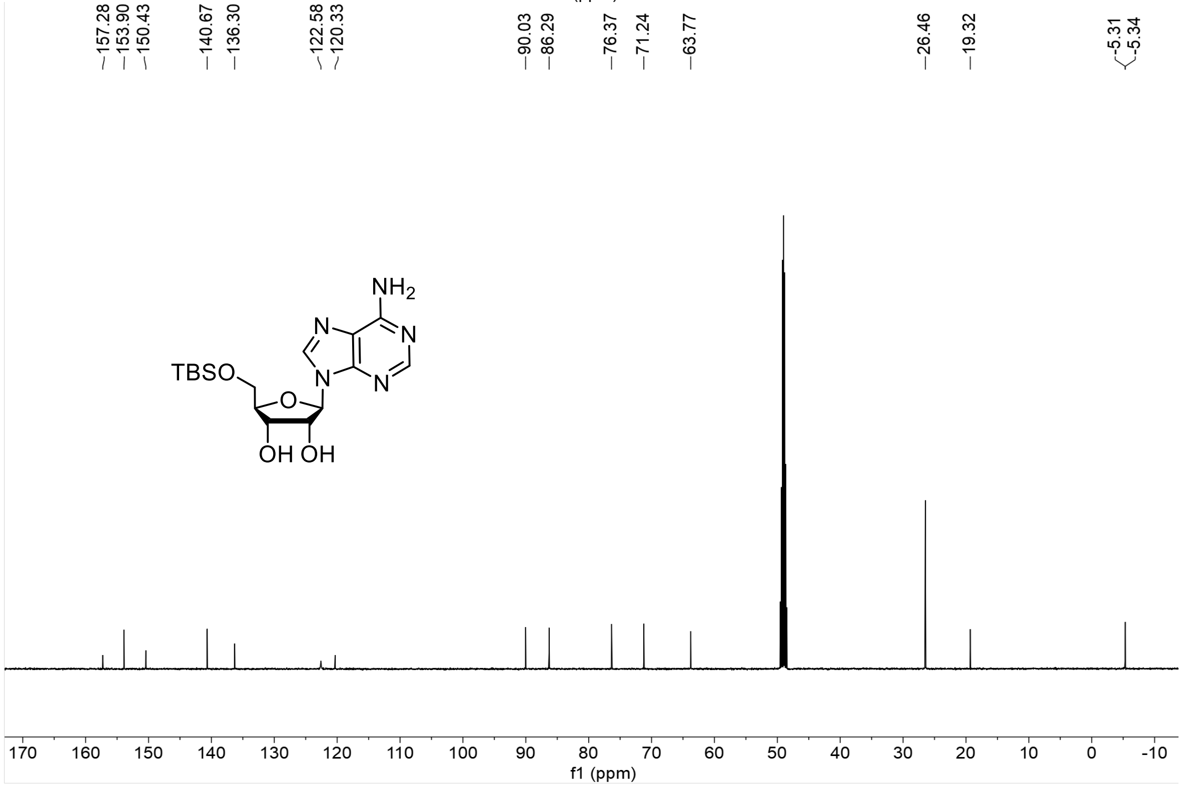

Figure S26. ^{1}H and ^{13}C NMR of (2R,3R,4S,5R)-2-(6-amino-9H-purin-9-yl)-5-(((tert-butyldimethylsilyl)oxy)methyl)tetrahydrofuran-3,4-diol.

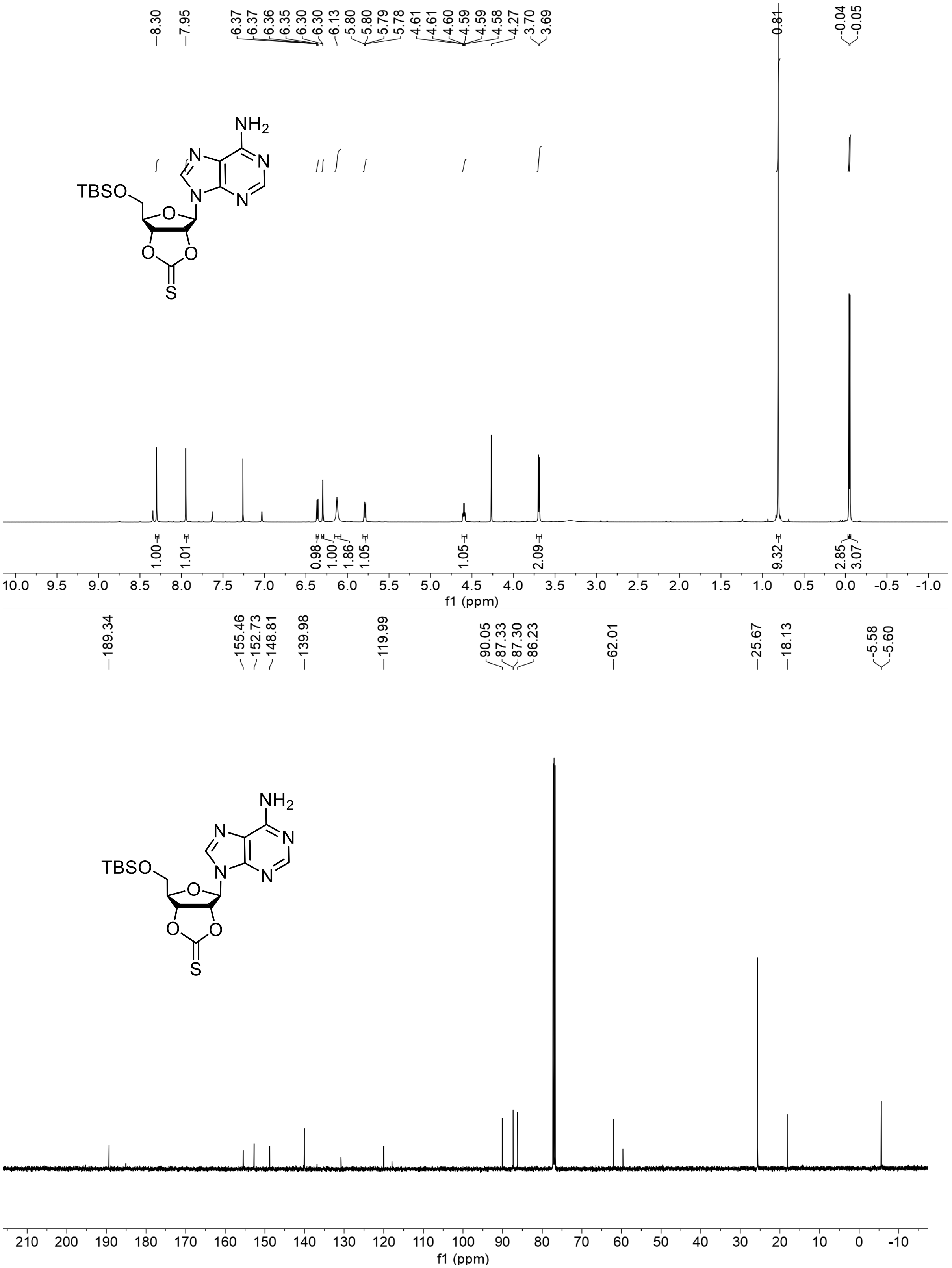

Figure S27. ¹H and ¹³C NMR of (3aR,4R,6R,6aR)-4-(6-amino-9H-purin-9-yl)-6-(((tert-butyldimethylsilyl)oxy)methyl) tetra-hydrofuro[3,4-d][1,3]dioxole-2-thione.

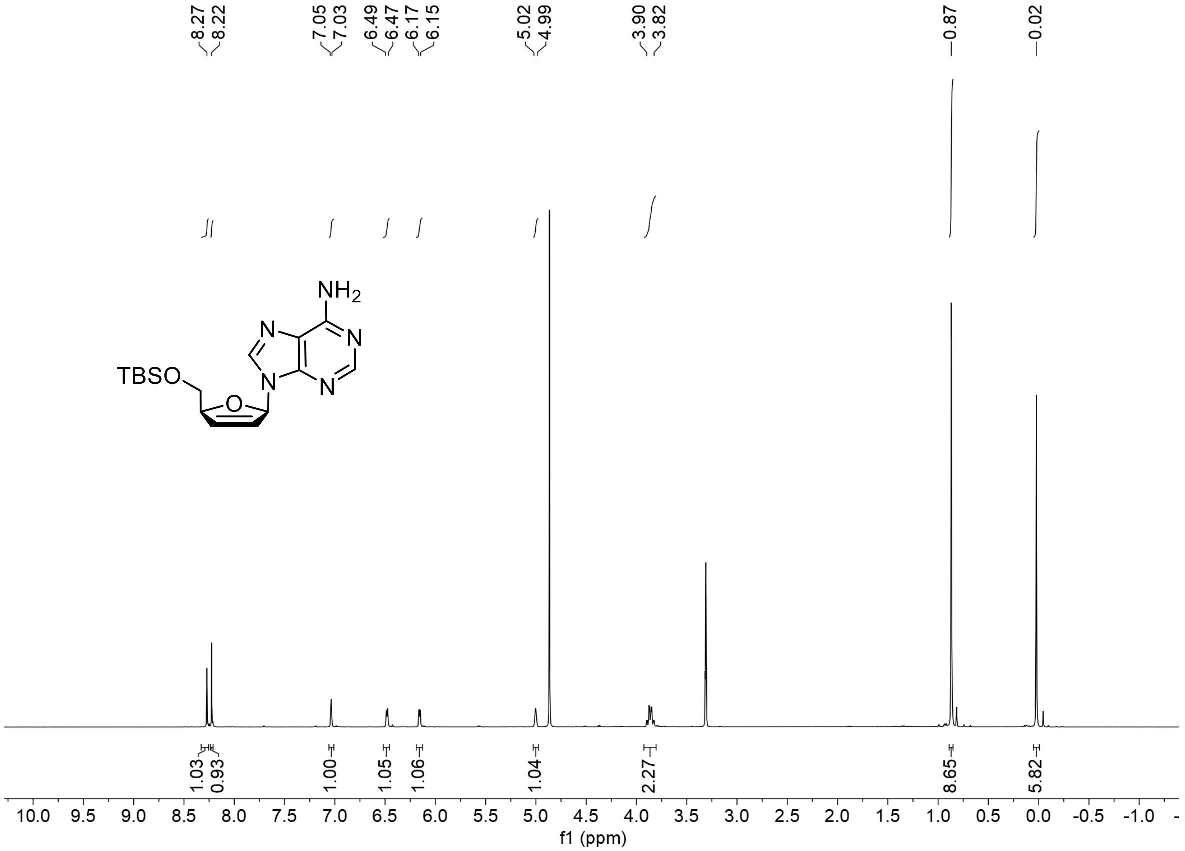

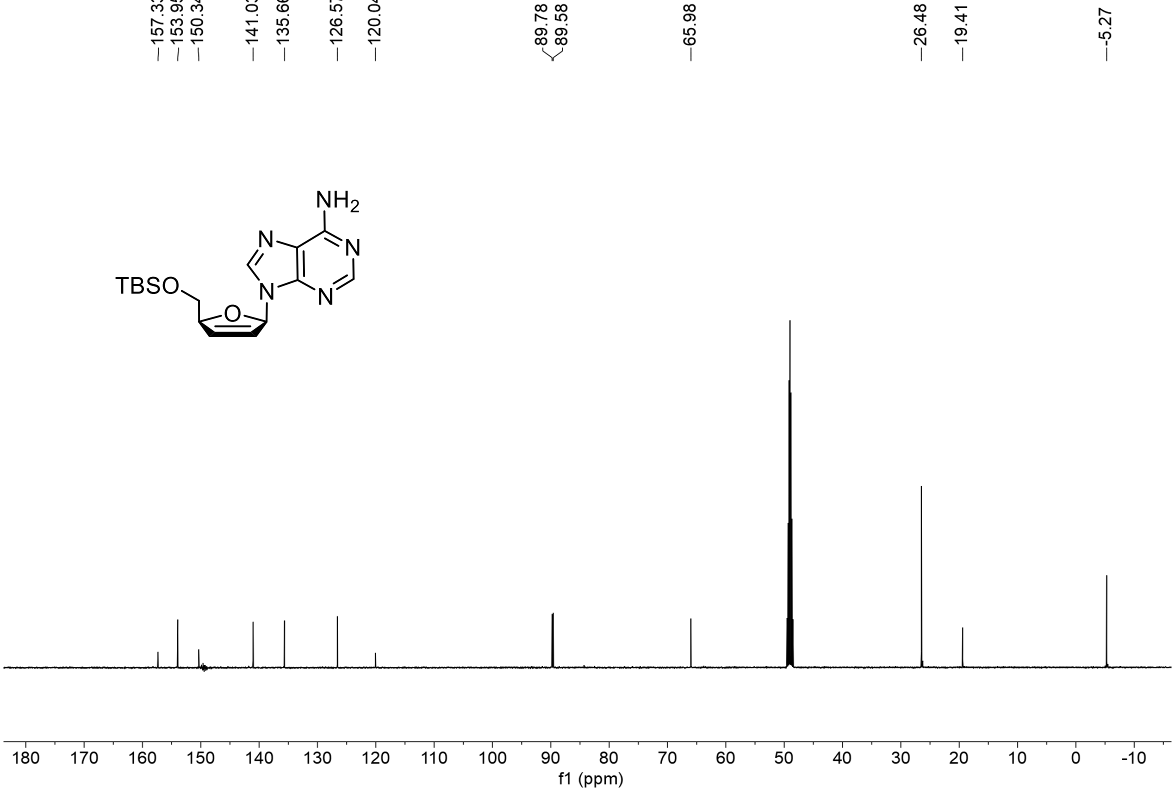

Figure S28. ^{1}H and ^{13}C NMR of 9-((2R,5S)-5-(((tert-butyldimethylsilyl)oxy)methyl)-2,5-dihydrofuran-2-yl)-9H-purin-6-amine.

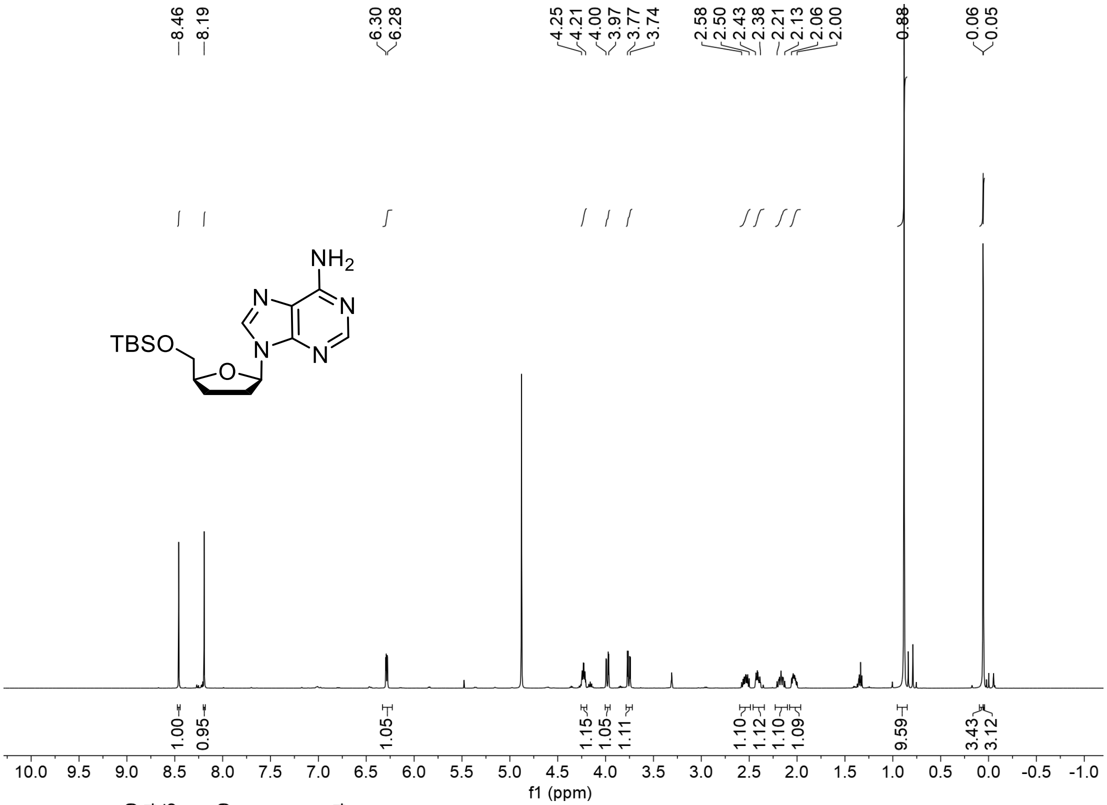

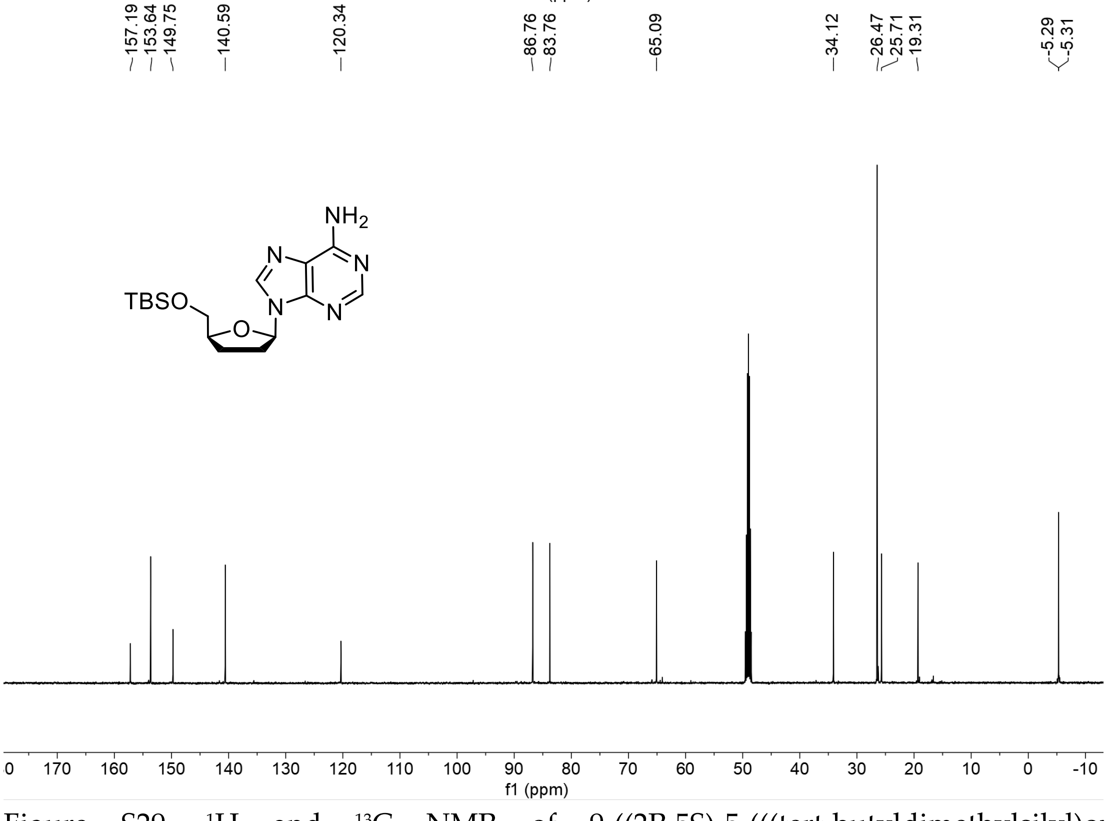

Figure S29. ^{1}H and ^{13}C NMR of 9-((2R,5S)-5-(((tert-butyldimethylsilyl)oxy)methyl)-tetrahydrofuran-2-yl)-9H-purin-6-amine.

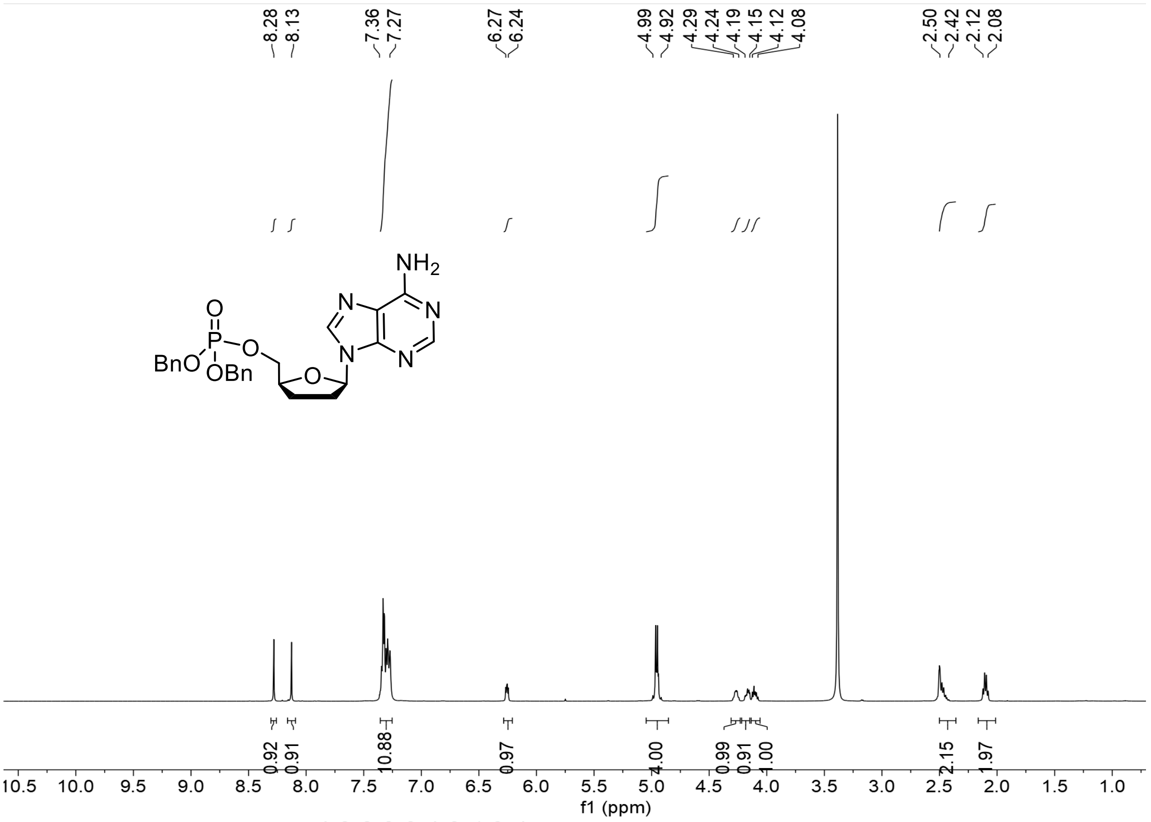
8.28
8.13
7.36
7.27
6.27
6.24
4.99
4.92
4.29
4.24
4.19
4.15
4.12
4.08
2.50
2.42
2.12
2.08
NH2
N
N
N
N
O
P
O
BnO
OBn
O
0.92
0.91
10.88
0.97
4.00
0.99
0.91
1.00
2.15
1.97
10.5 10.0 9.5 9.0 8.5 8.0 7.5 7.0 6.5 6.0 5.5 5.0 4.5 4.0 3.5 3.0 2.5 2.0 1.5 1.0
f1 (ppm)

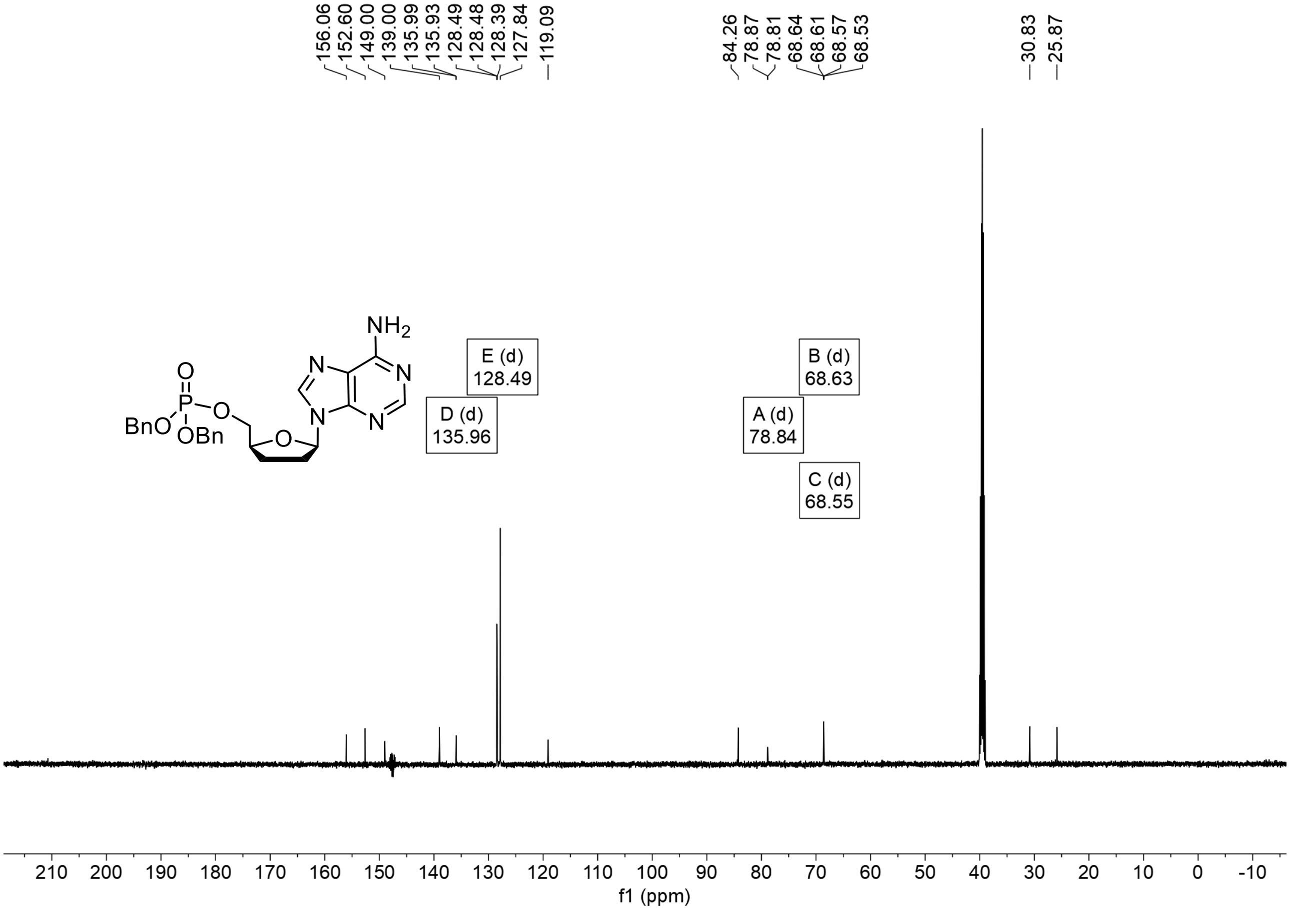
156.06
152.60
149.00
139.00
135.99
135.93
128.49
128.48
128.39
127.84
119.09
84.26
78.87
78.81
68.64
68.61
68.57
68.53
30.83
25.87
NH2
N
N
N
N
O
P
O
BnO
OBn
O
E (d)
128.49
D (d)
135.96
B (d)
68.63
A (d)
78.84
C (d)
68.55
210 200 190 180 170 160 150 140 130 120 110 100 90 80 70 60 50 40 30 20 10 0 -10
f1 (ppm)

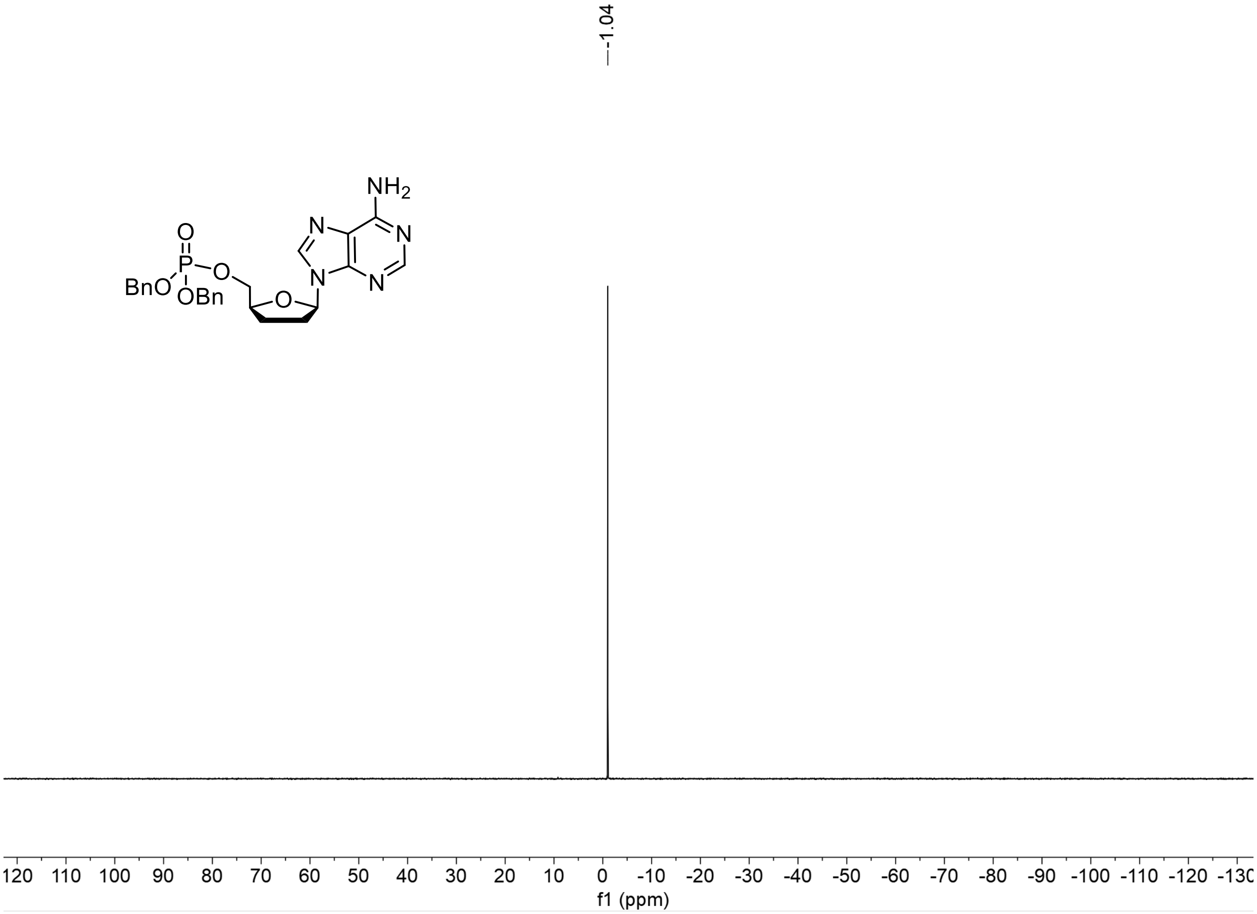

Figure S30. ^{1}H ^{13}C and ^{31}P NMR of ((2S,5R)-5-(6-amino-9H-purin-9-yl)tetrahydrofuran-2-yl)methyl dibenzyl phosphate.

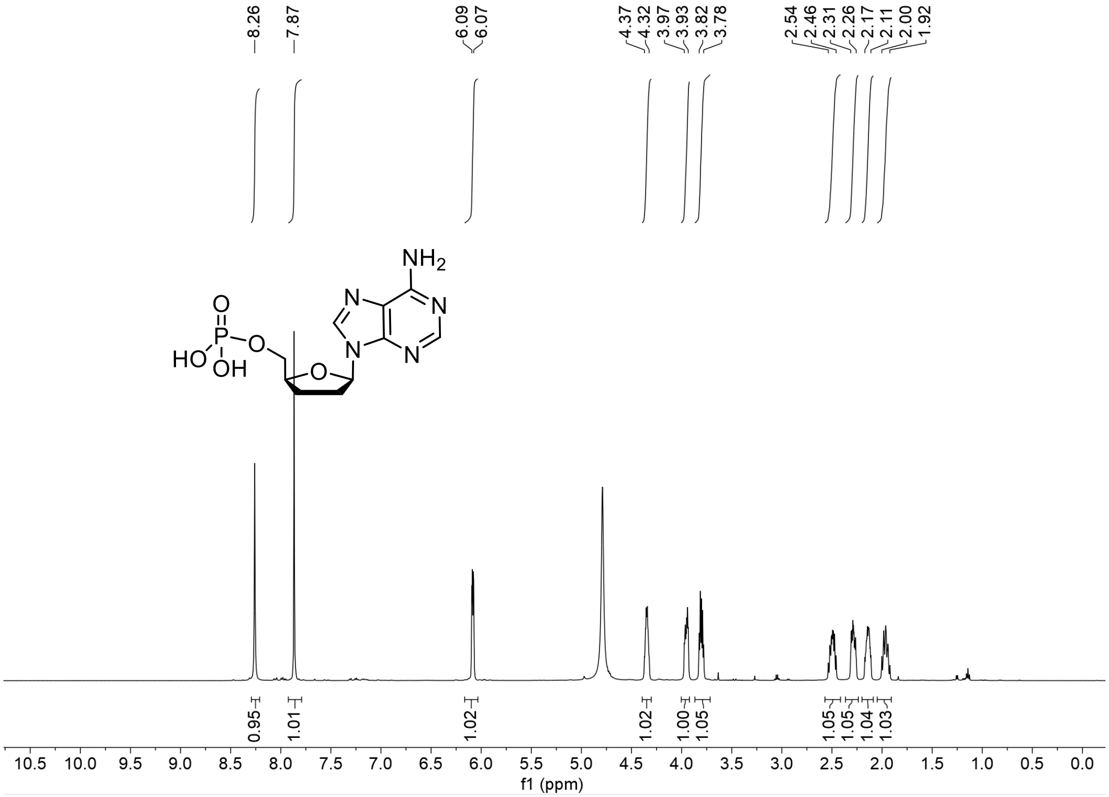
8.26
7.87
6.09
6.07
4.37
4.32
3.97
3.93
3.82
3.78
2.54
2.46
2.31
2.26
2.17
2.11
2.00
1.92
NH2
N
N
N
N
O
P
O
HO
OH
O
0.95
1.01
1.02
1.02
1.00
1.05
1.05
1.05
1.04
1.03
f1 (ppm)

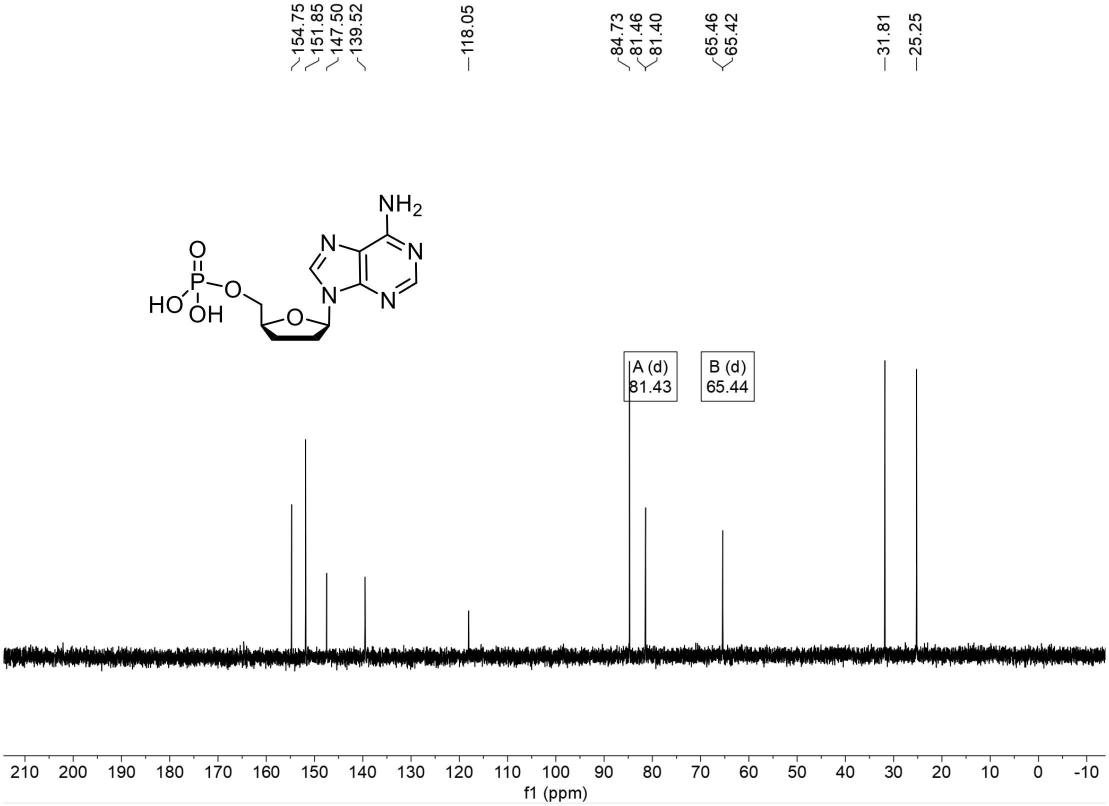
154.75
151.85
147.50
139.52
118.05
84.73
81.46
81.40
65.46
65.42
31.81
25.25
NH2
N
N
N
N
O
P
O
HO
OH
O
A (d)
81.43
B (d)
65.44
f1 (ppm)

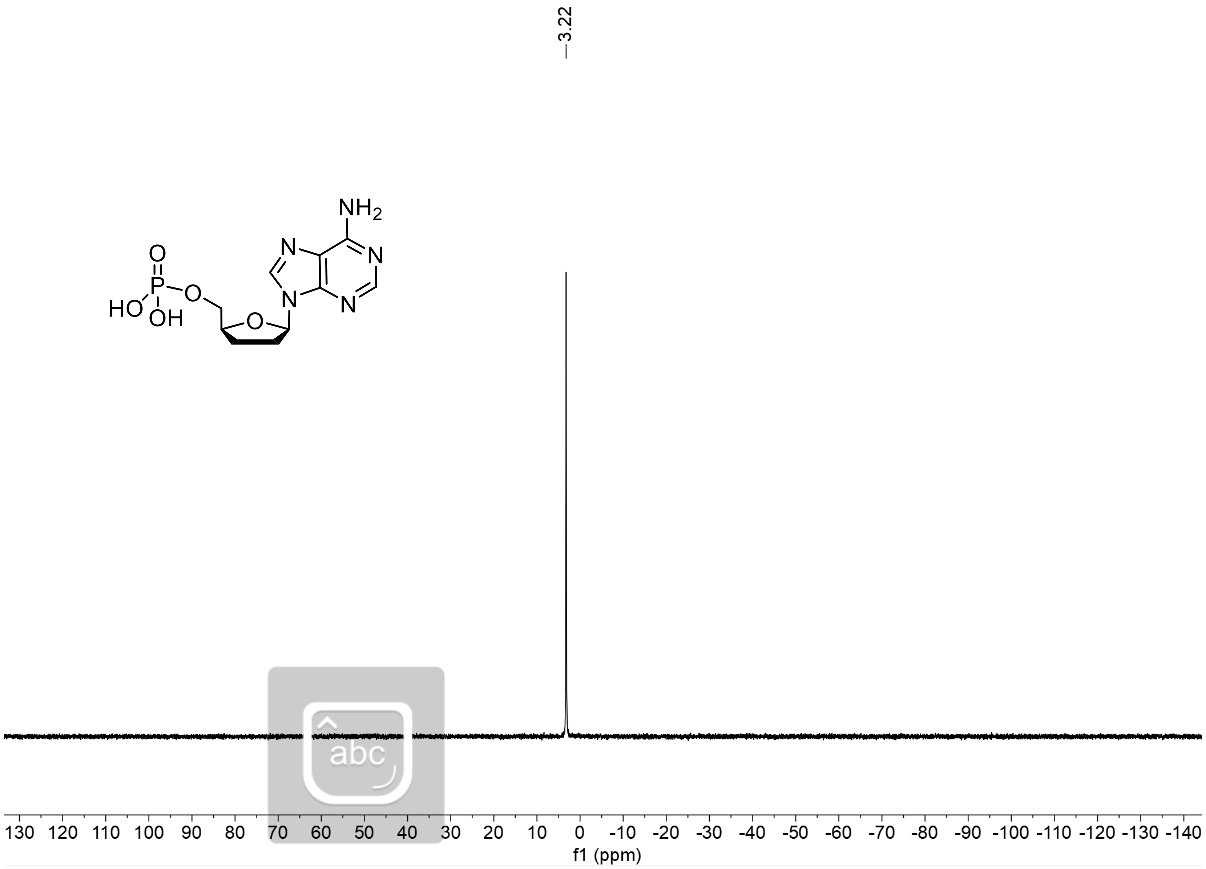

Figure S31. ^{1}H,^{13}C and ^{31}P NMR of ((2S,5R)-5-(6-amino-9H-purin-9-yl)tetrahydrofuran-2-yl)methyl dihydrogen phosphate.

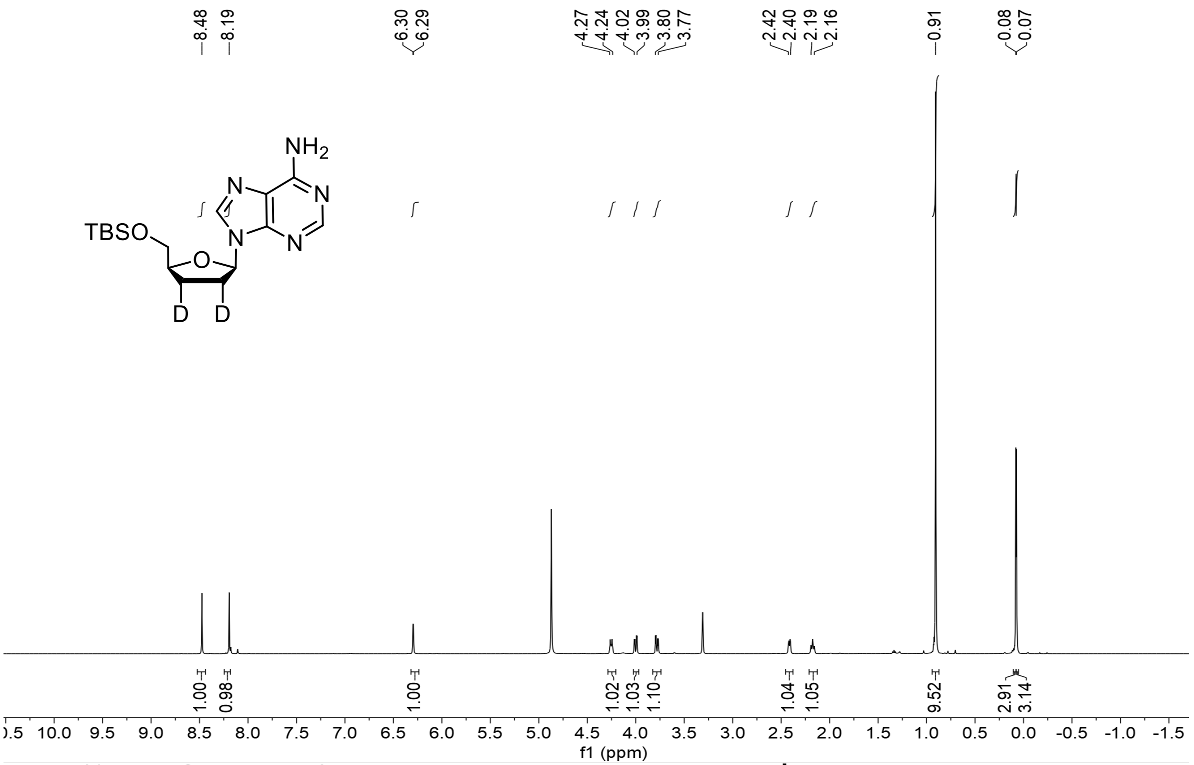

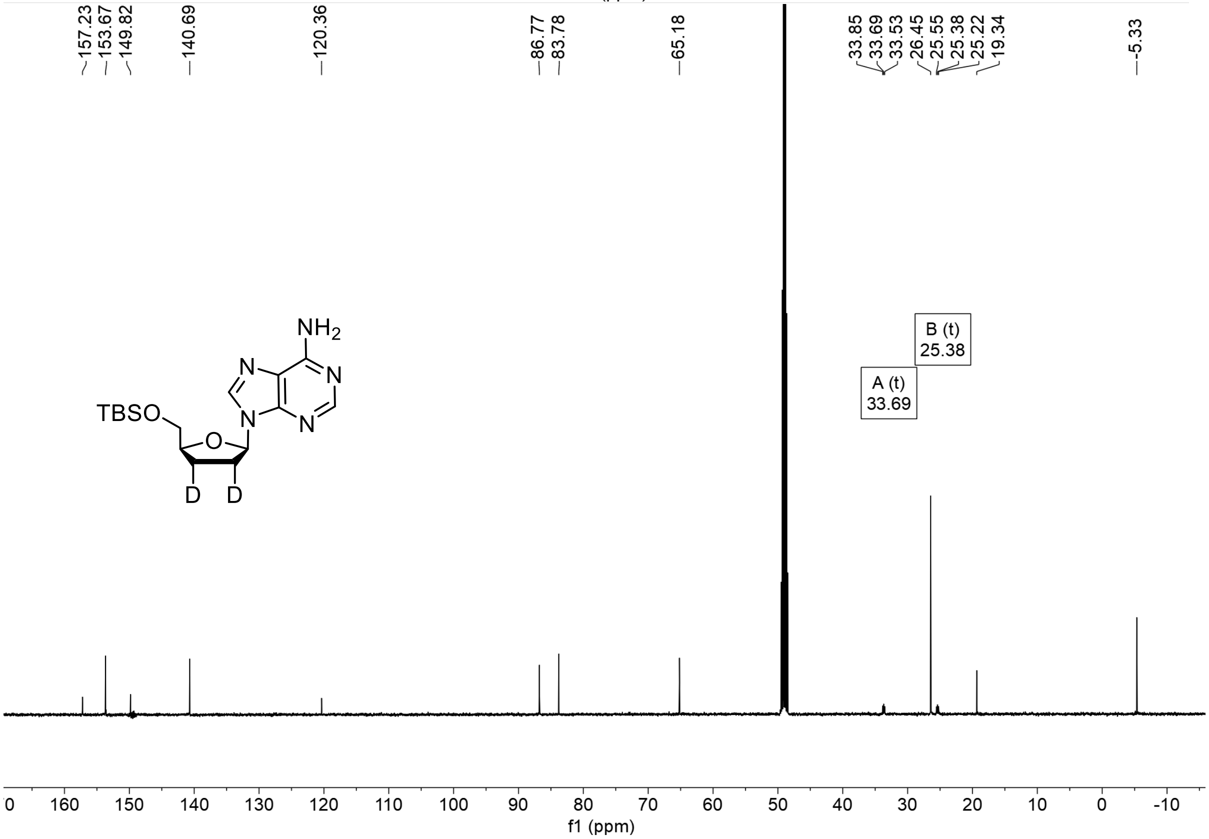

Figure S32. ^{1}H and ^{13}C NMR of 9-((2R,3R,4S,5S)-5-(((tert-butyldimethylsilyl)oxy)methyl)-tetrahydrofuran-2-yl-3,4-d2)-9H-purin-6-amine.

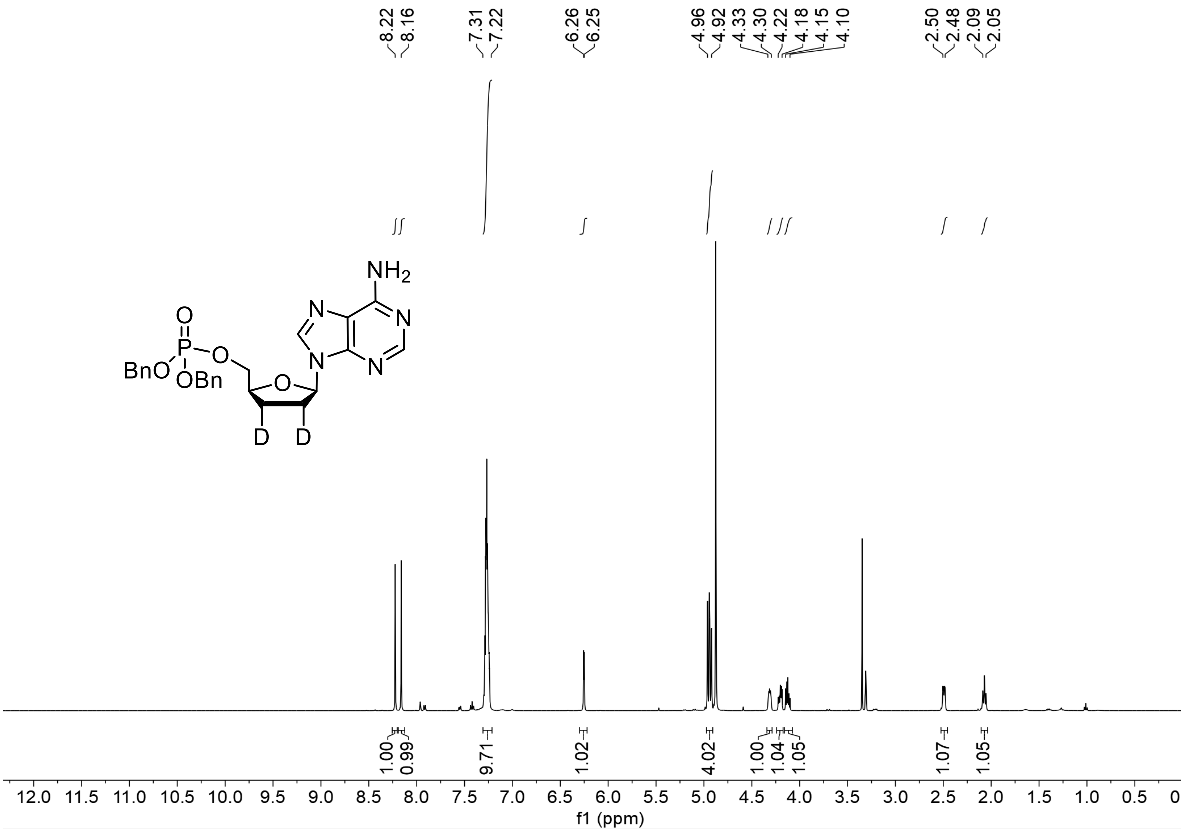
8.22
8.16
7.31
7.22
6.26
6.25
4.96
4.92
4.33
4.30
4.22
4.18
4.15
4.10
2.50
2.48
2.09
2.05
NH2
N
N
N
N
O
P
O
BnO
OBn
O
D
D
1.00
0.99
9.71
1.02
4.02
1.00
1.04
1.05
1.07
1.05
12.0 11.5 11.0 10.5 10.0 9.5 9.0 8.5 8.0 7.5 7.0 6.5 6.0 5.5 5.0 4.5 4.0 3.5 3.0 2.5 2.0 1.5 1.0 0.5 0
f1 (ppm)

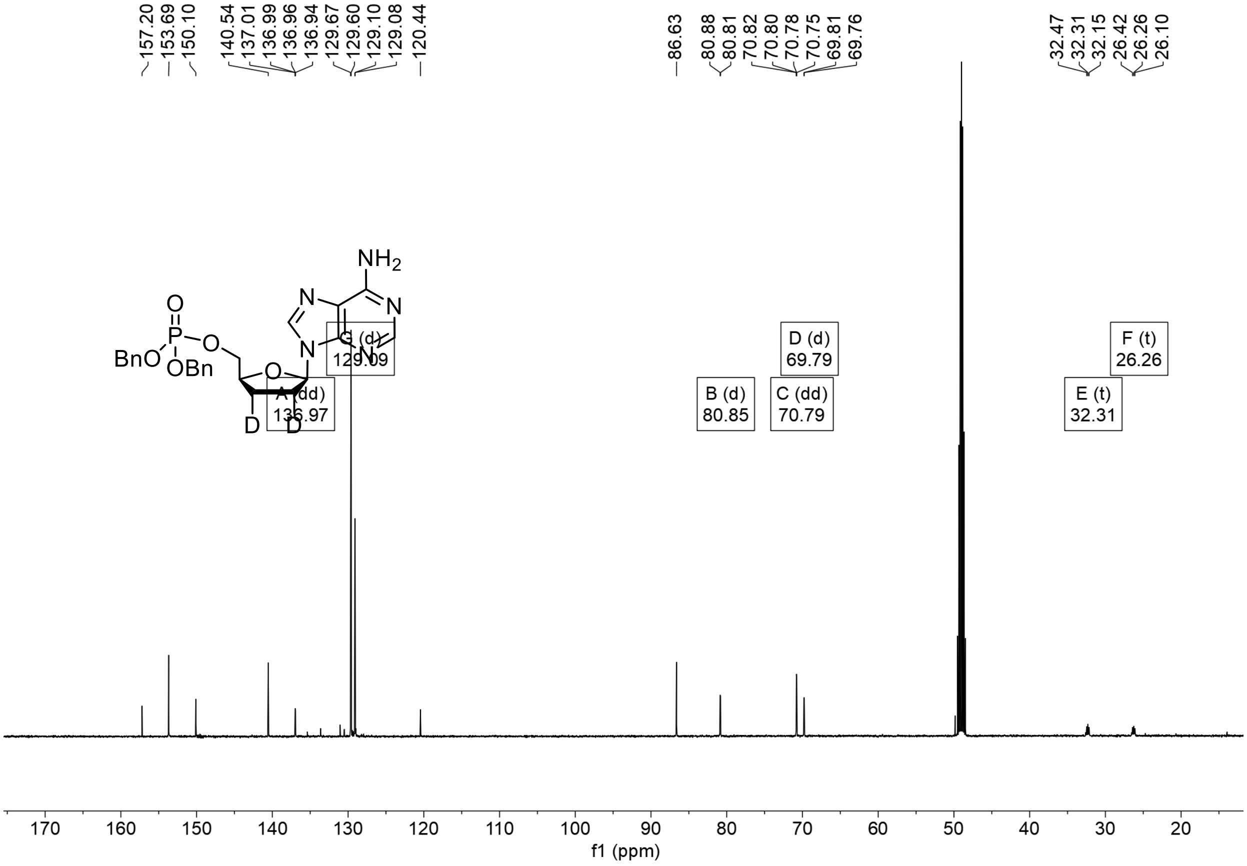
157.20
153.69
150.10
140.54
137.01
136.99
136.96
136.94
129.67
129.60
129.10
129.08
120.44
86.63
80.88
80.81
70.82
70.80
70.78
70.75
69.81
69.76
32.47
32.31
32.15
26.42
26.26
26.10
G (d)
129.09
A (dd)
136.97
B (d)
80.85
C (dd)
70.79
D (d)
69.79
E (t)
32.31
F (t)
26.26
170 160 150 140 130 120 110 100 90 80 70 60 50 40 30 20
f1 (ppm)

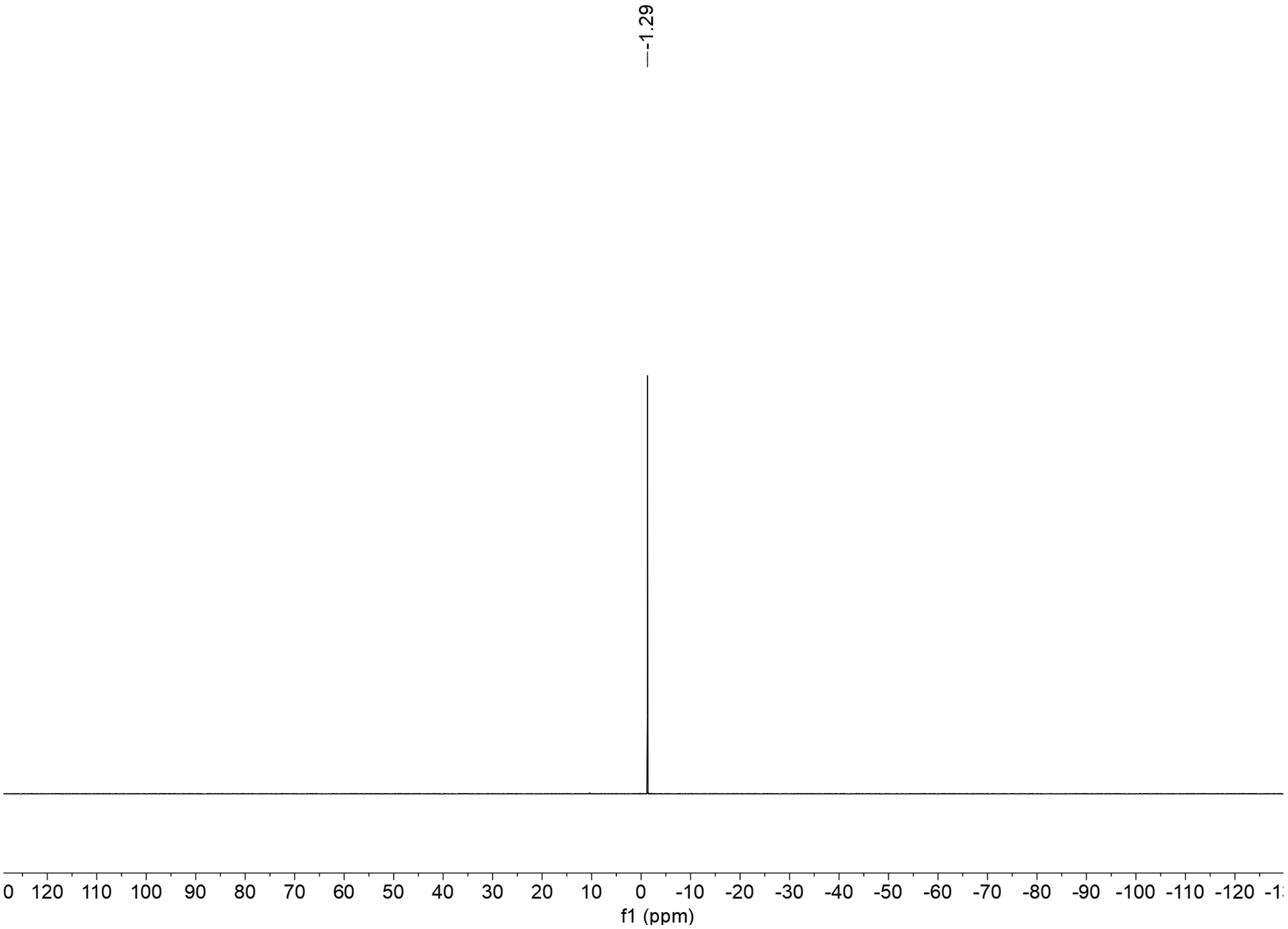

Figure S33. ^{1}H, ^{13}C and ^{31}P NMR of ((2S,3S,4R,5R)-5-(6-amino-9H-purin-9-yl)tetrahydrofuran-2-yl-3,4-d2)methyl dibenzyl phosphate.

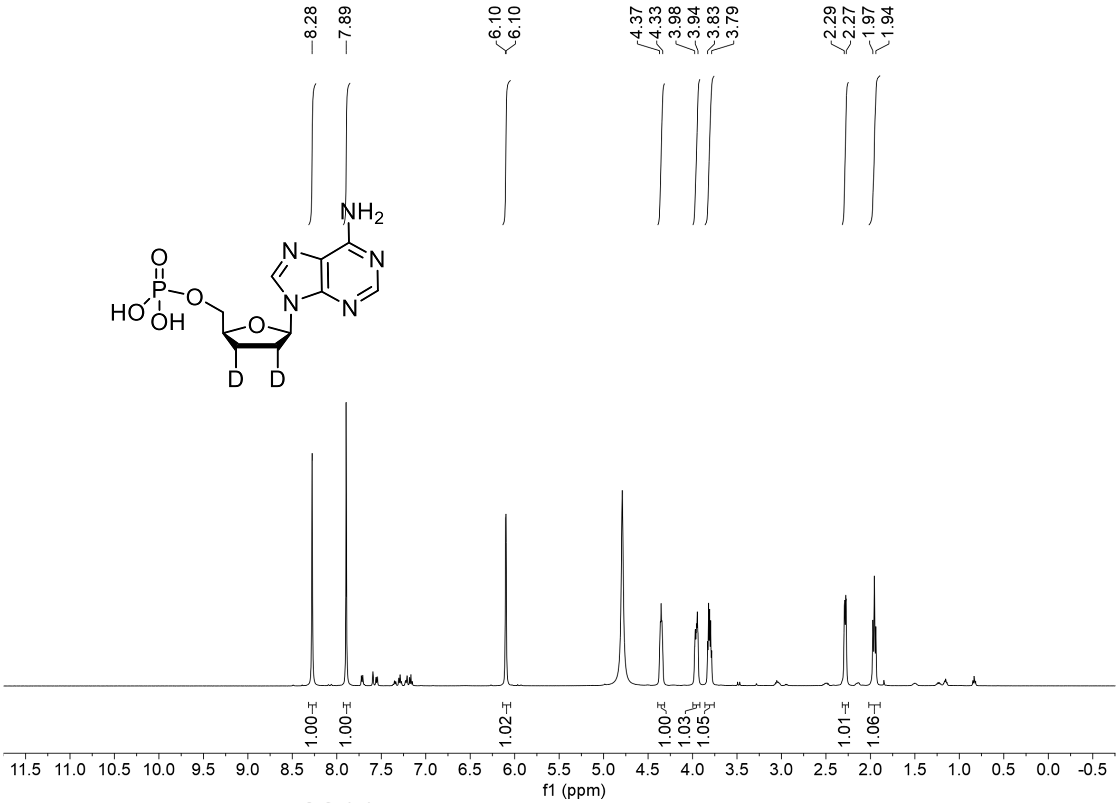
8.28
7.89
6.10
6.10
4.37
4.33
3.98
3.94
3.83
3.79
2.29
2.27
1.97
1.94
1.00
1.00
1.02
1.00
1.03
1.05
1.01
1.06
f1 (ppm)

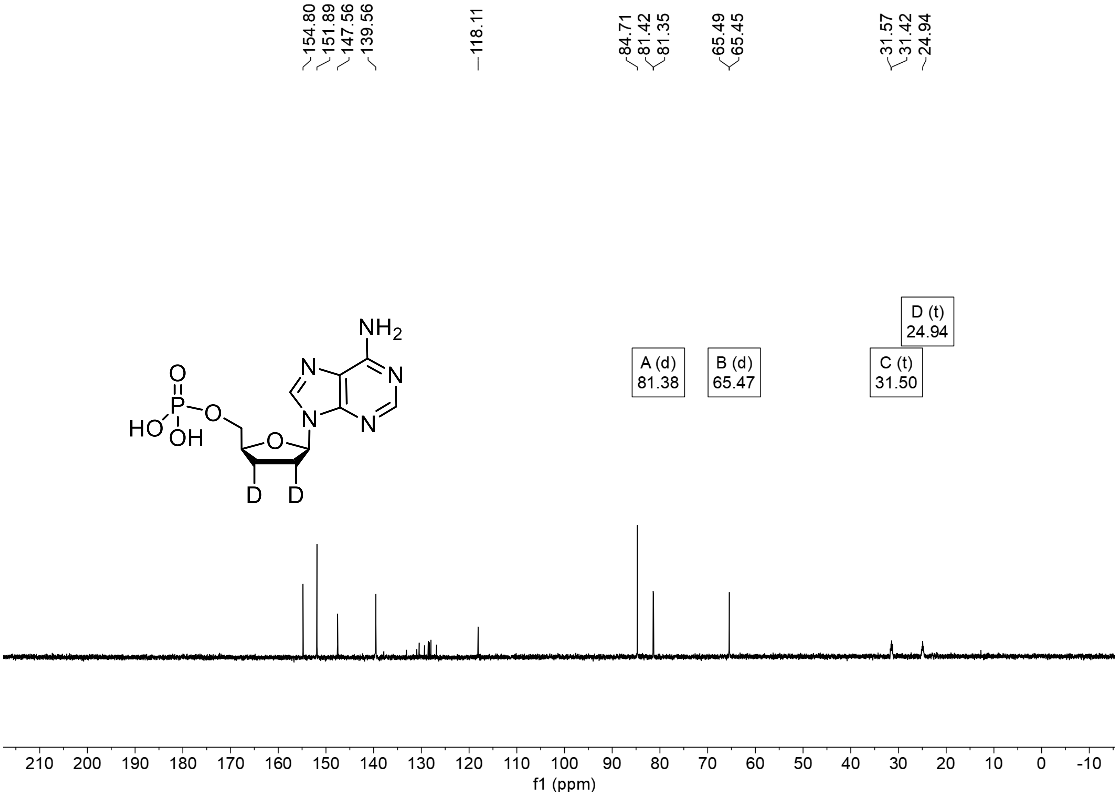
154.80
151.89
147.56
139.56
118.11
84.71
81.42
81.35
65.49
65.45
31.57
31.42
24.94
A (d)
81.38
B (d)
65.47
C (t)
31.50
D (t)
24.94
f1 (ppm)

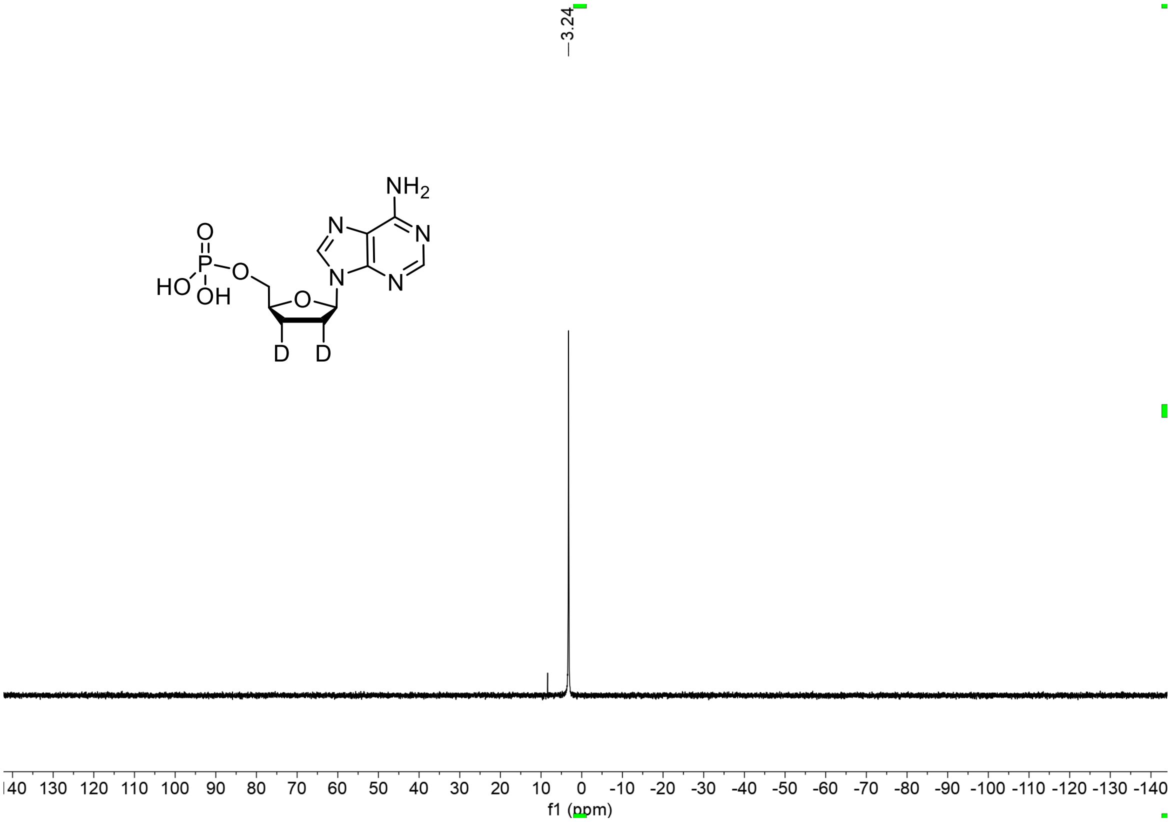

Figure S34. ^{1}H, ^{13}C and ^{31}P NMR of ((2S,3S,4R,5R)-5-(6-amino-9H-purin-9-yl)tetrahydrofuran-2-yl-3,4-d2)methyl dibenzyl phosphate.

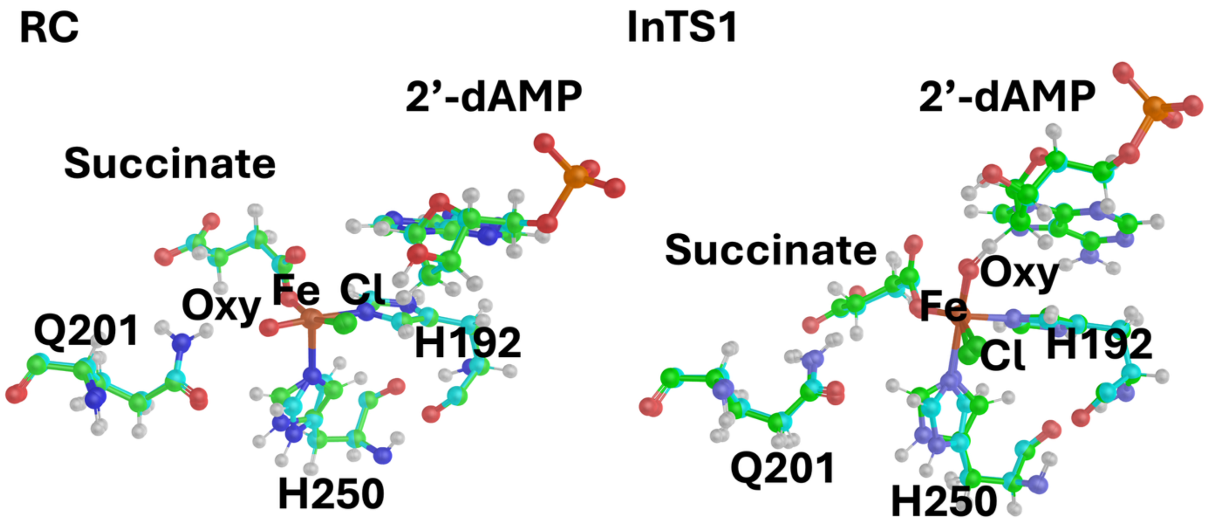

Figure S35. Overlaid RC and InTS1 QM/MM optimized structures obtained using QM1 (cyan) and QM2 region with Q201 (green) of the Off2-RC snapshot.

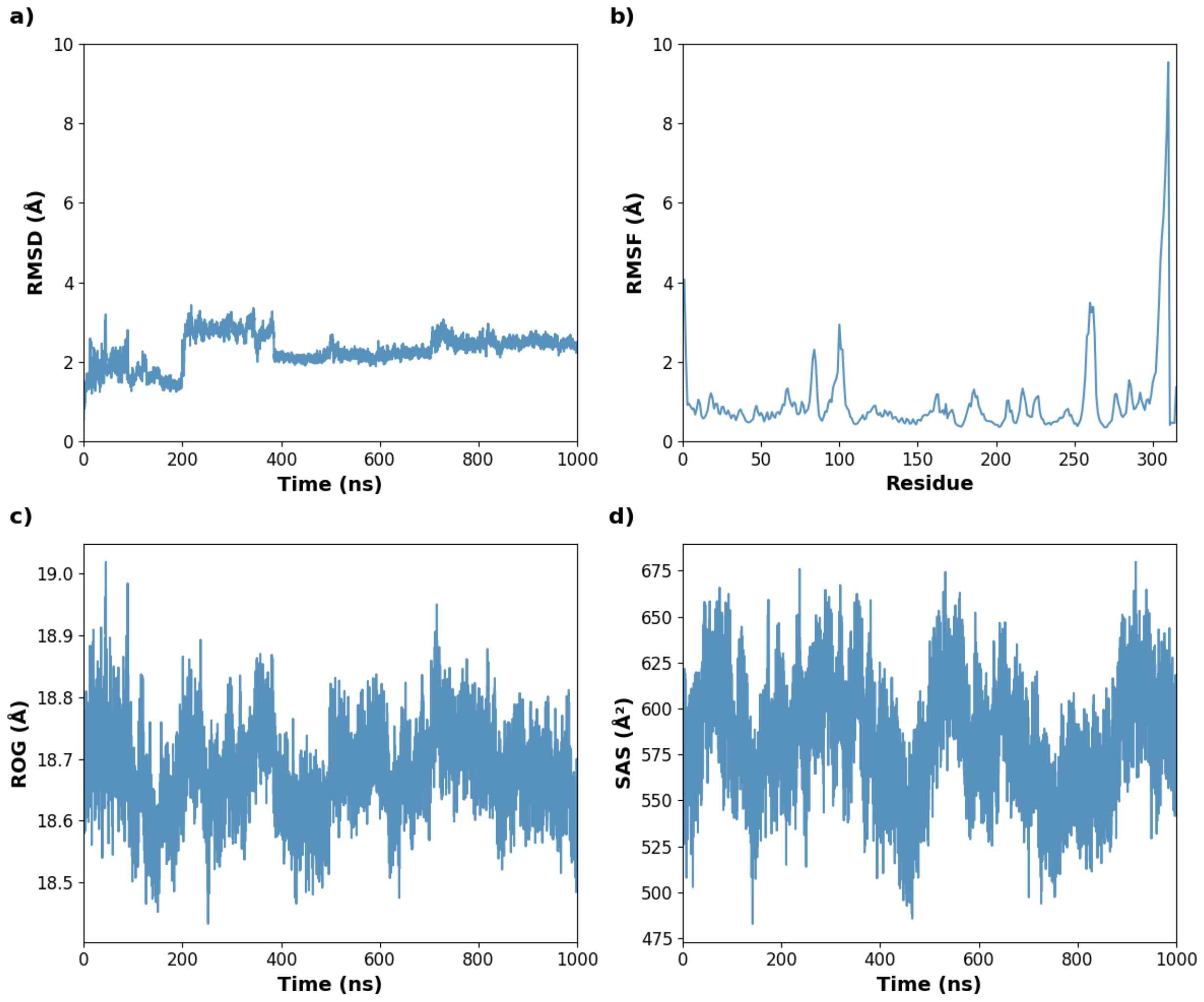

Figure S36. Molecular dynamics analysis of the Offline FeIII-superoxo complex. a) The root mean square deviation (RMSD) of the dynamics suggests the system is equilibrated, b) the root mean square fluctuation (RMSF) of the system identifies flexible regions, c) Radius of gyration (ROG) shows the stability of the overall protein fold, and d) Solvent accessible surface area (SAS) implies that the system is equilibrated.

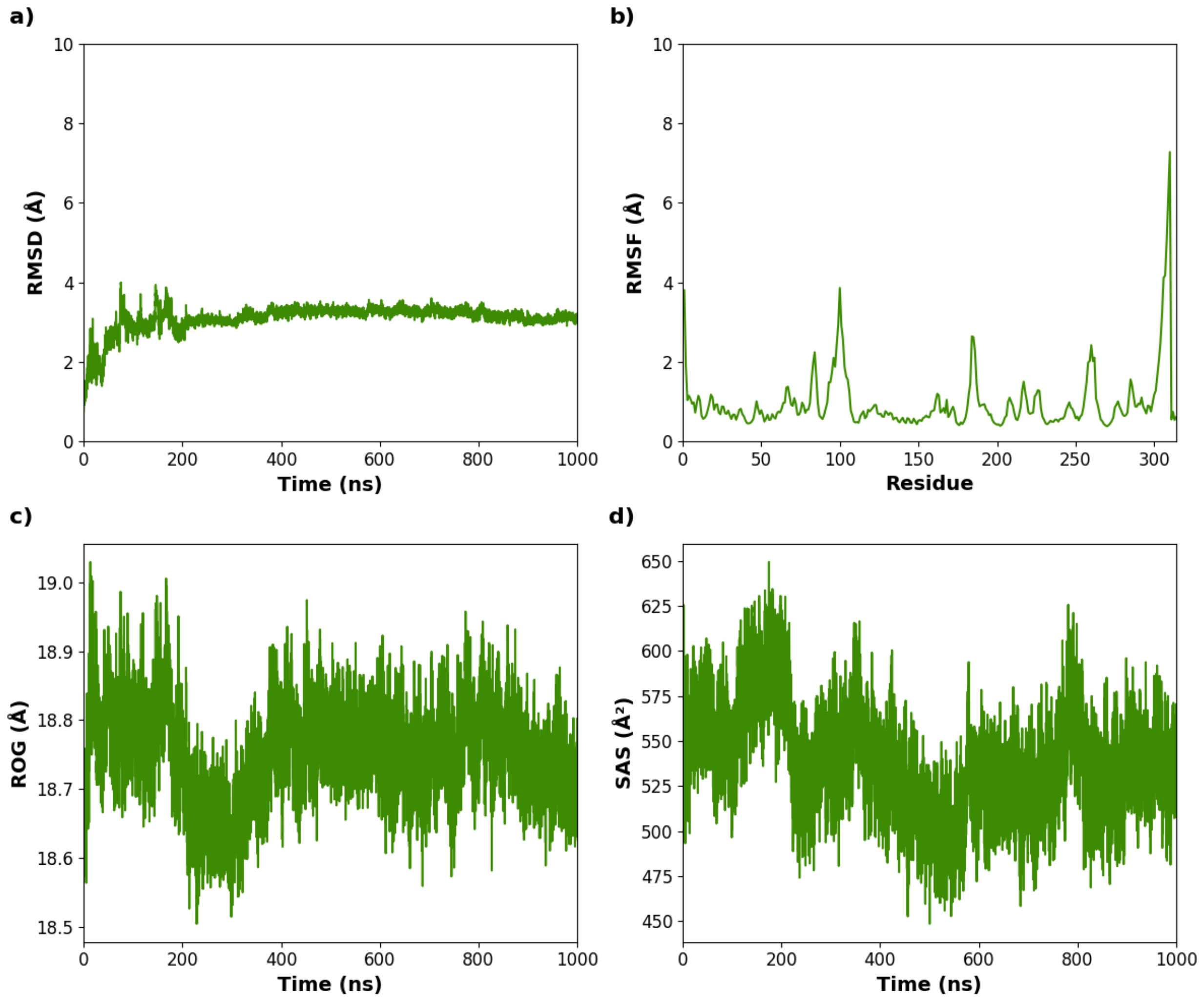

Figure S37. Molecular dynamics analysis of the inline FeIII-superoxo complex. a) RMSD of the dynamics suggests the system is equilibrated, b) RMSF of the system identifies flexible regions, c) ROG shows the stability of the overall protein fold, and d) SAS implies that the system is equilibrated.

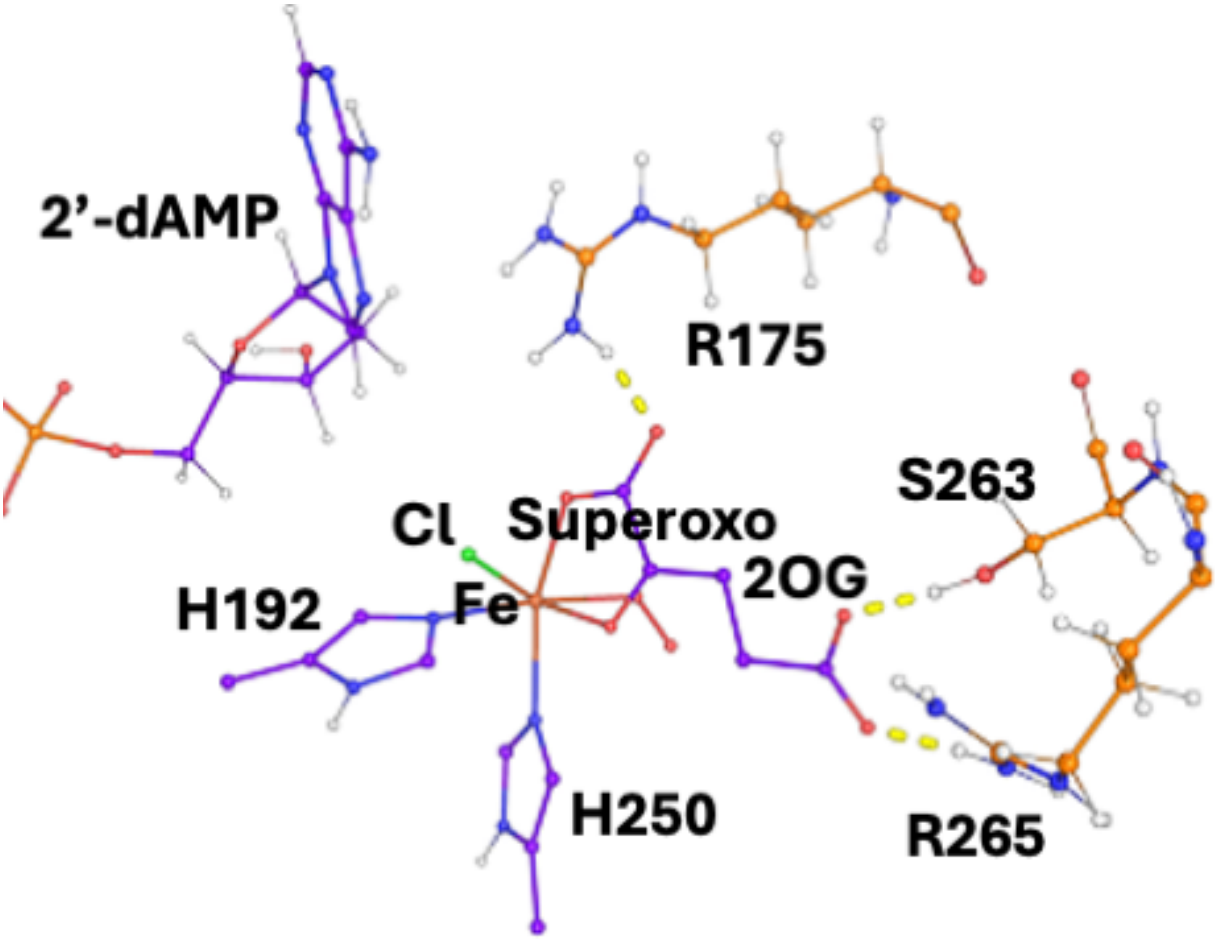

Figure S38. Interactions stabilizing the 2OG in the offline Fe^{III}-superoxo dynamics.

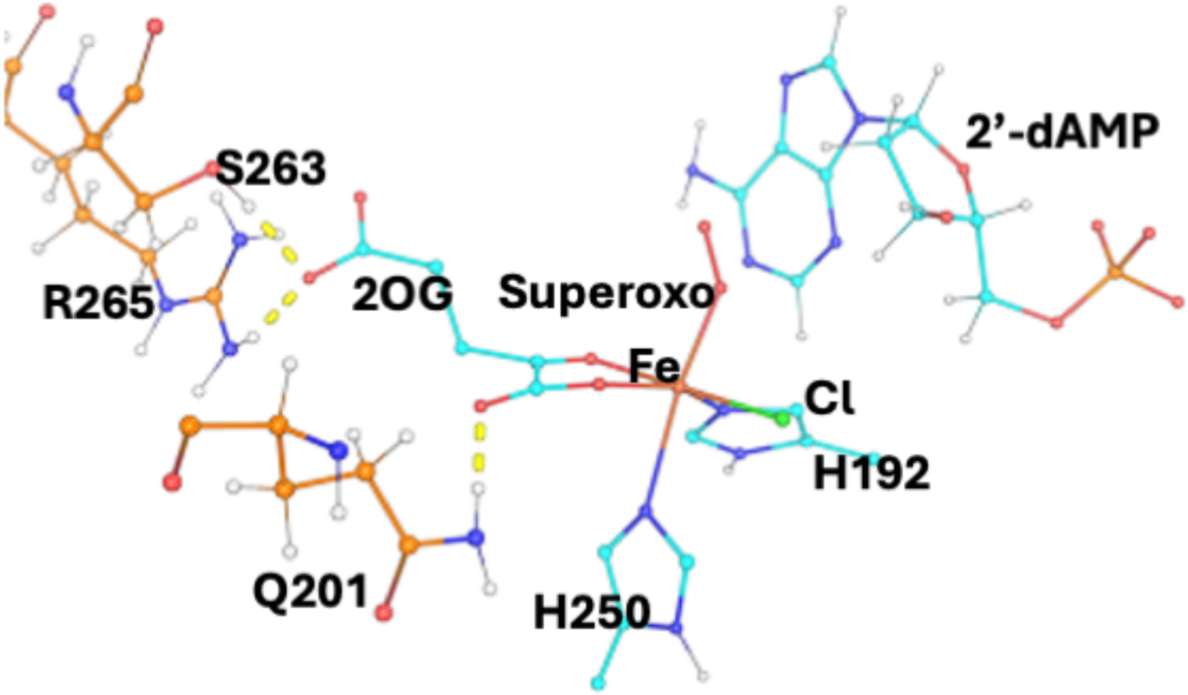

Figure S39. Interactions stabilizing the 2OG in inline Fe^{III}-superoxo dynamics.

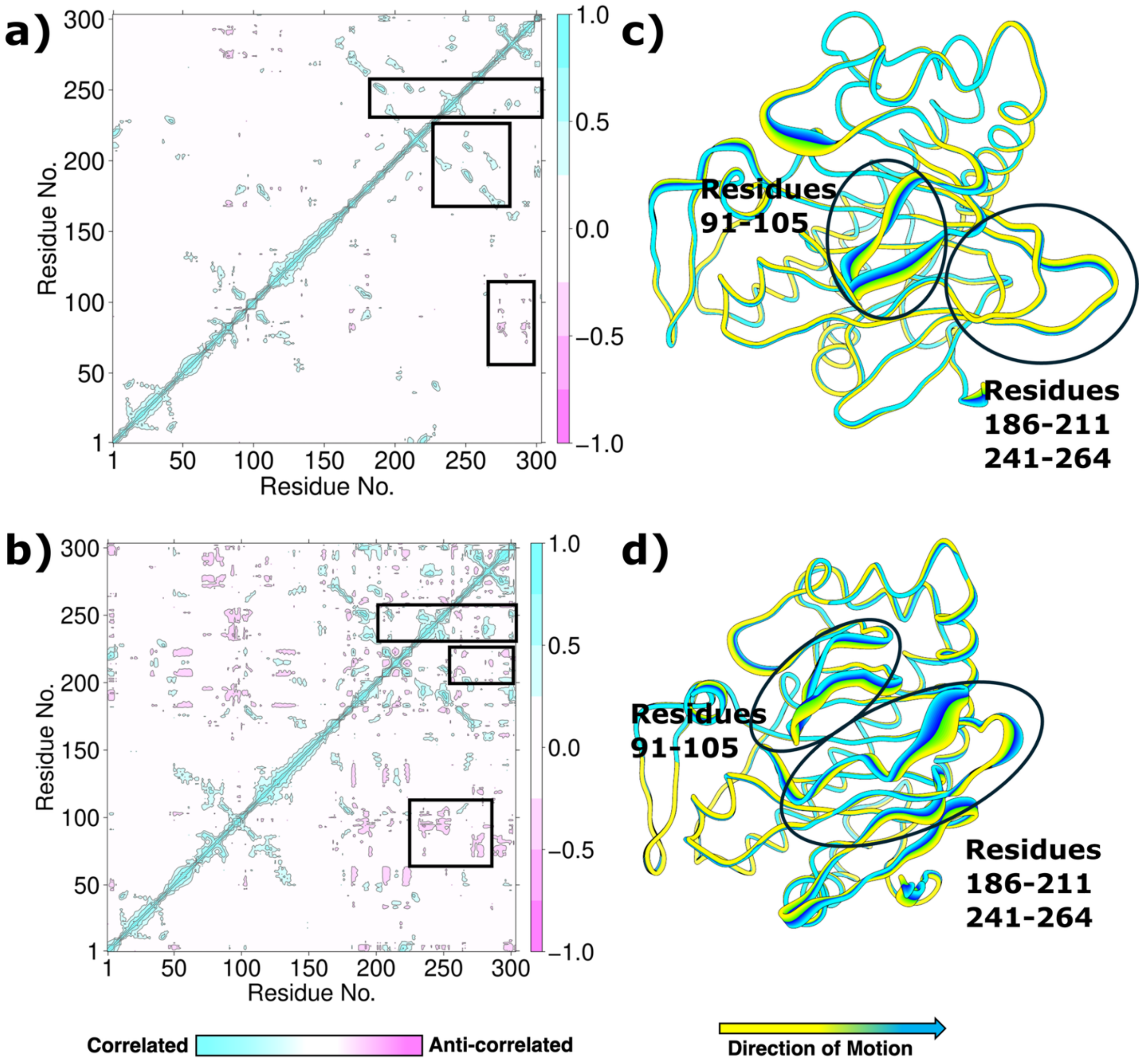

Figure S40. The overall protein dynamics of the offline and inline FeIII-superoxo systems. The dynamic cross-correlation matrix shows the regions of correlated and anti-correlated motions in a) offline and b) inline Fe^{III}-superoxo systems, and Principal component analysis shows the flexible regions of the c) offline and d) inline Fe^{III}-superoxo systems. The boxed regions show correlated/anti-correlated motion of residues *91-105*, *186*-211, and 241-*264* with the active site residues (a and b), and circled regions show the flexibility of the corresponding residues (c and d).

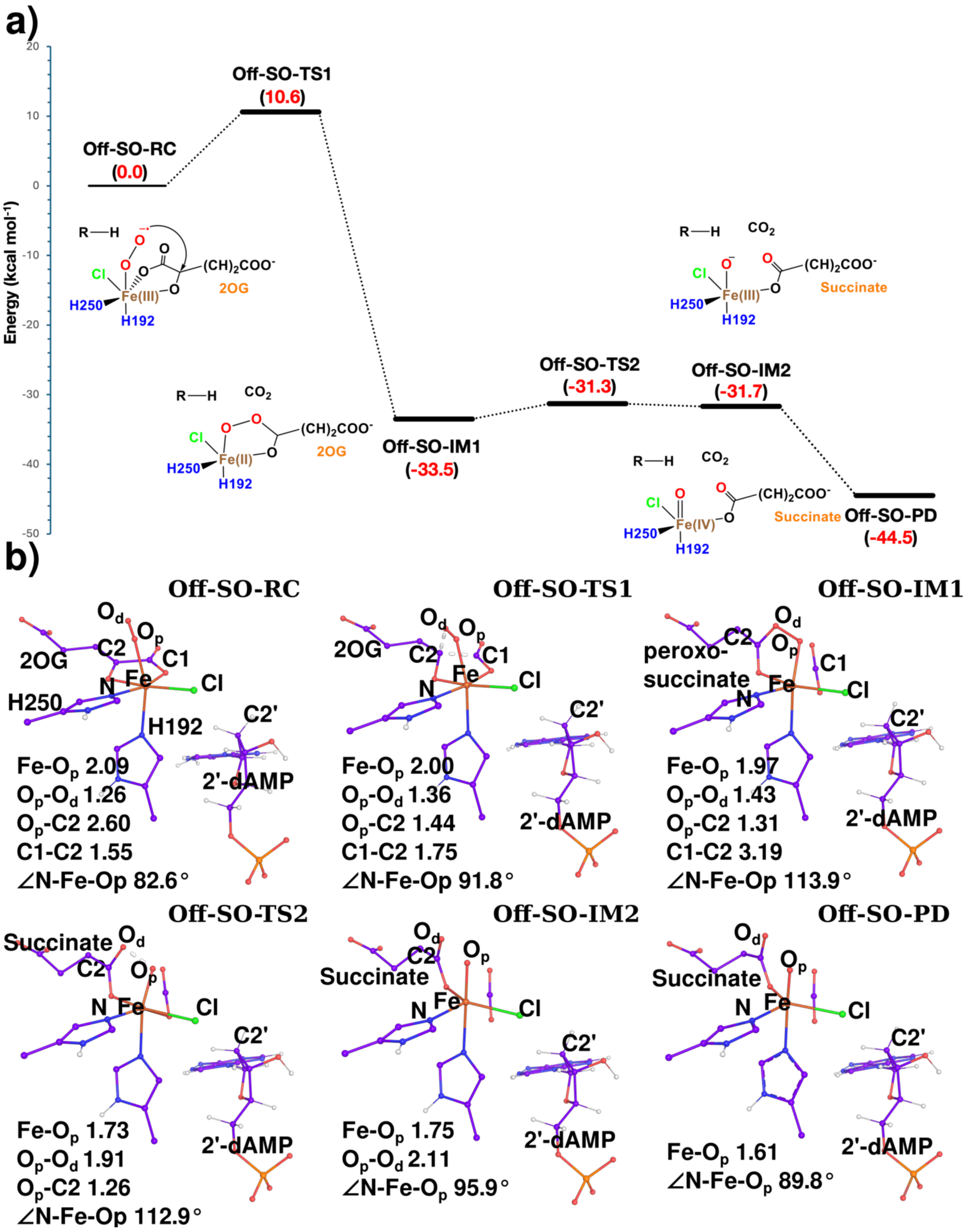

Figure S41. The reaction profile and molecular structures of the iron center derived from QM/MM calculations. a) Reaction profile of the O_2 activation reaction from the offline Fe^{III}-superoxo system. Relative energies are given in kcal/mol calculated at zero-point corrected energies (QM(B3)/MM level). b) Representations of QM/MM optimized structures obtained during the reaction path. Non-polar hydrogens are hidden except for the substrate for clarity. Distances are mentioned in Å.

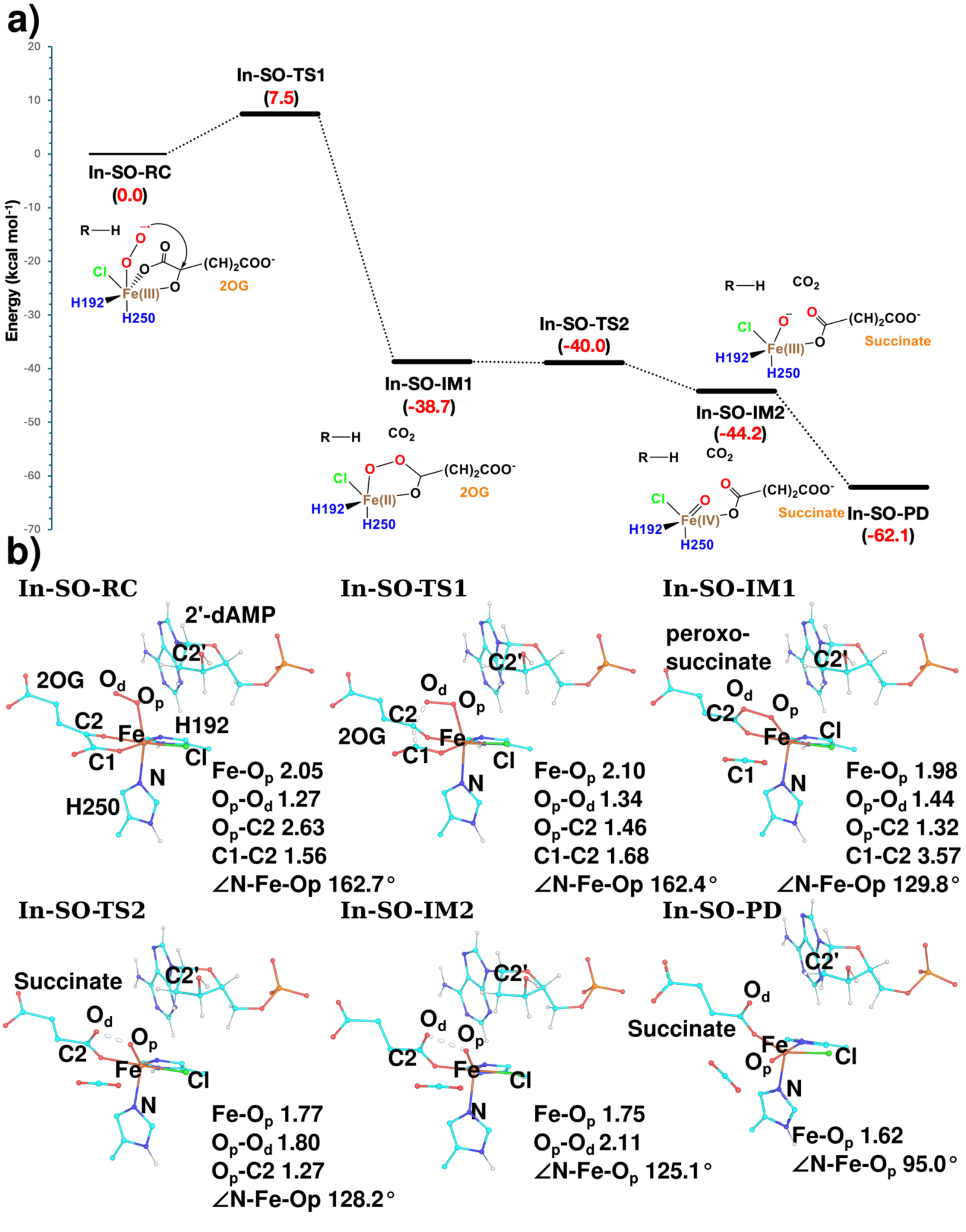

Figure S42. The reaction profile and molecular structures of the iron center derived from QM/MM calculations. a) Reaction profile of the O_2 activation reaction from the inline Fe^{III}-superoxo system. Relative energies are given in kcal/mol calculated at zero-point corrected energies (QM(B3)/MM level). b) Representations of QM/MM optimized structures obtained during the reaction path. Non-polar hydrogens are hidden except for the substrate for clarity. Distances are mentioned in Å.

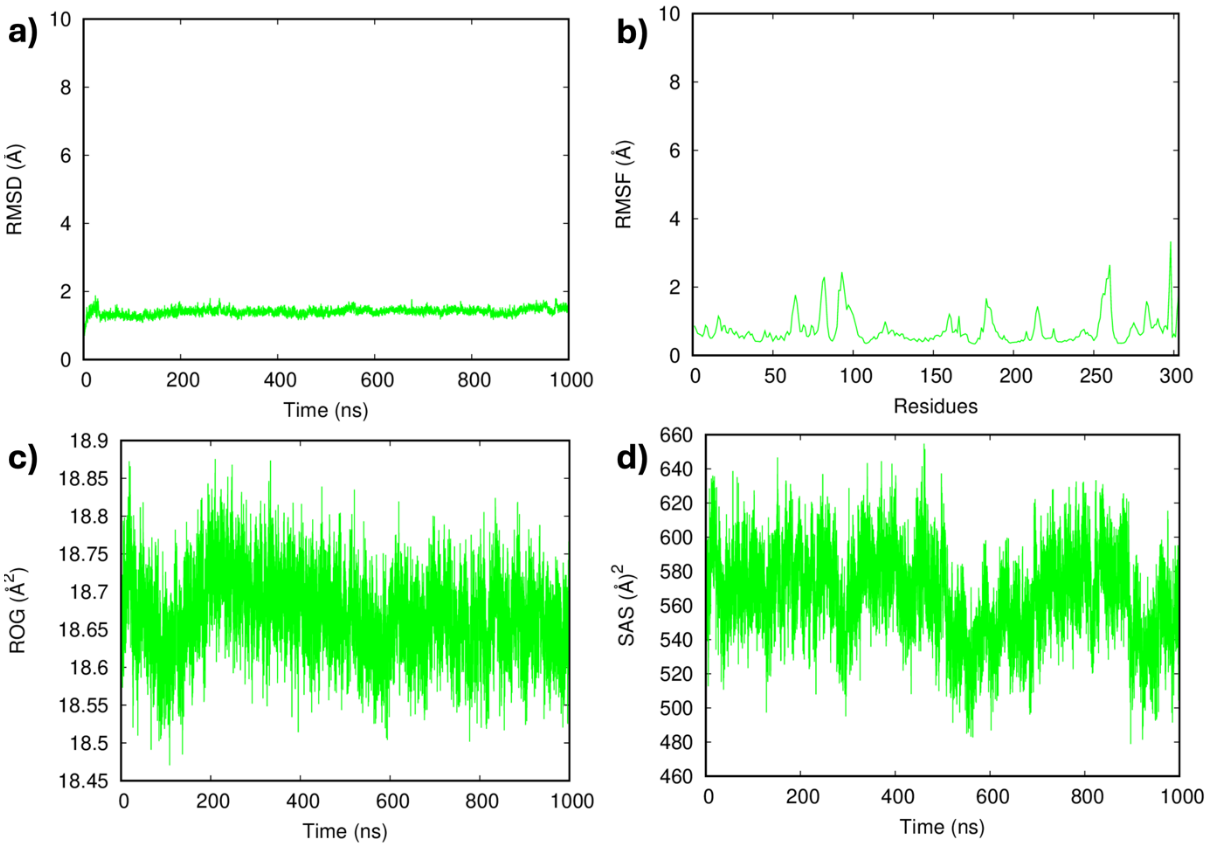

Figure S43. Molecular dynamics analysis of the Offline ferryl complex. a) RMSD of the dynamics suggests the system is equilibrated, b) RMSF of the system identifies flexible regions, c) ROG shows the stability of the overall protein fold, and d) SAS implies that the system is equilibrated.

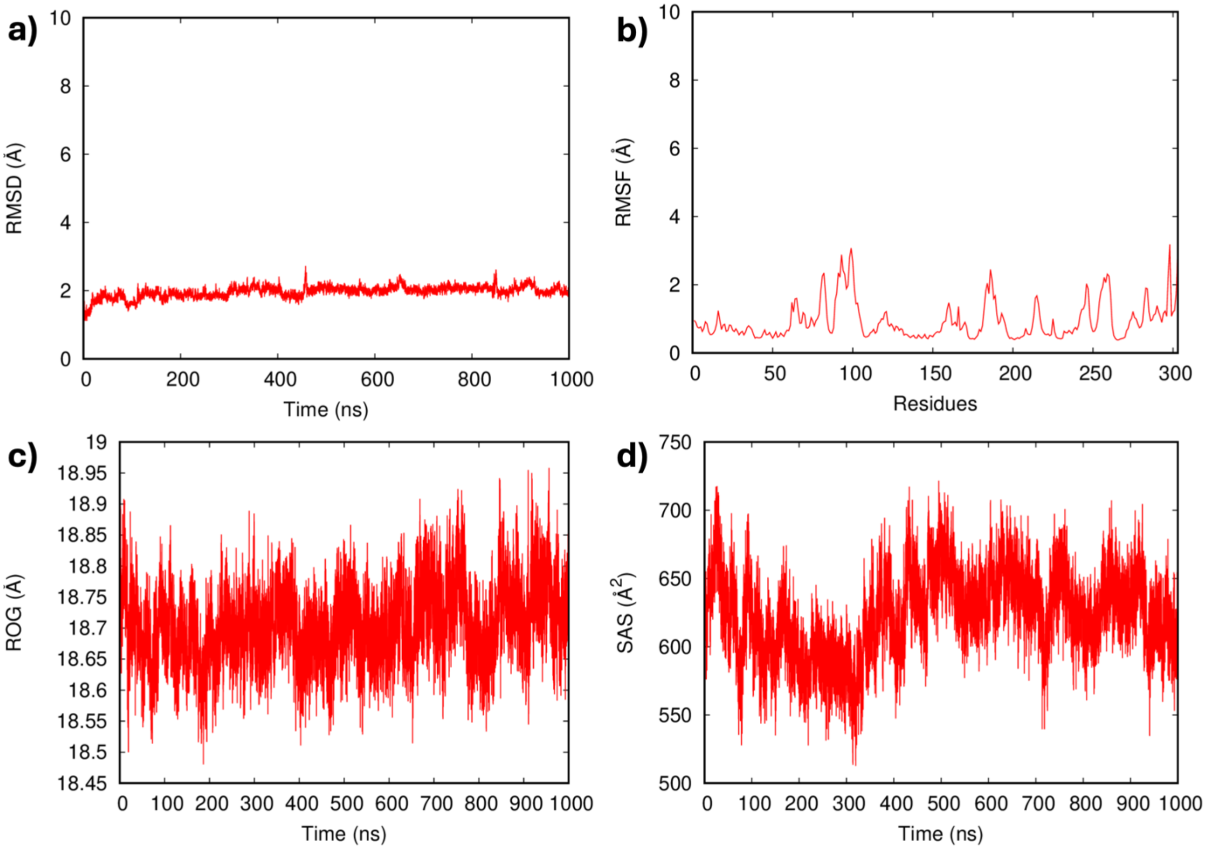

Figure S44. Molecular dynamics analysis of the Inline ferryl complex. a) RMSD of the dynamics suggests the system is equilibrated, b) RMSF of the system identifies flexible regions, c) ROG shows the stability of the overall protein fold, and d) SAS implies that the system is equilibrated.

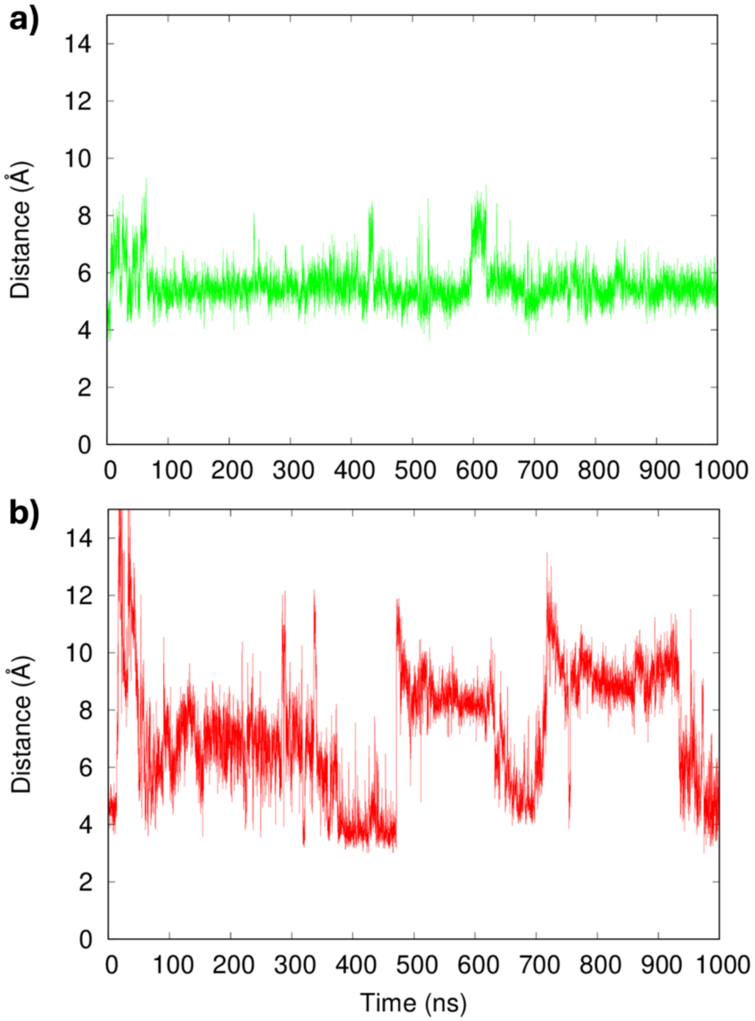

Figure S45. Plots depicting the fluctuations of the distance between the ferryl oxygen and C2′ of the substrate in a) offline and b) inline systems.

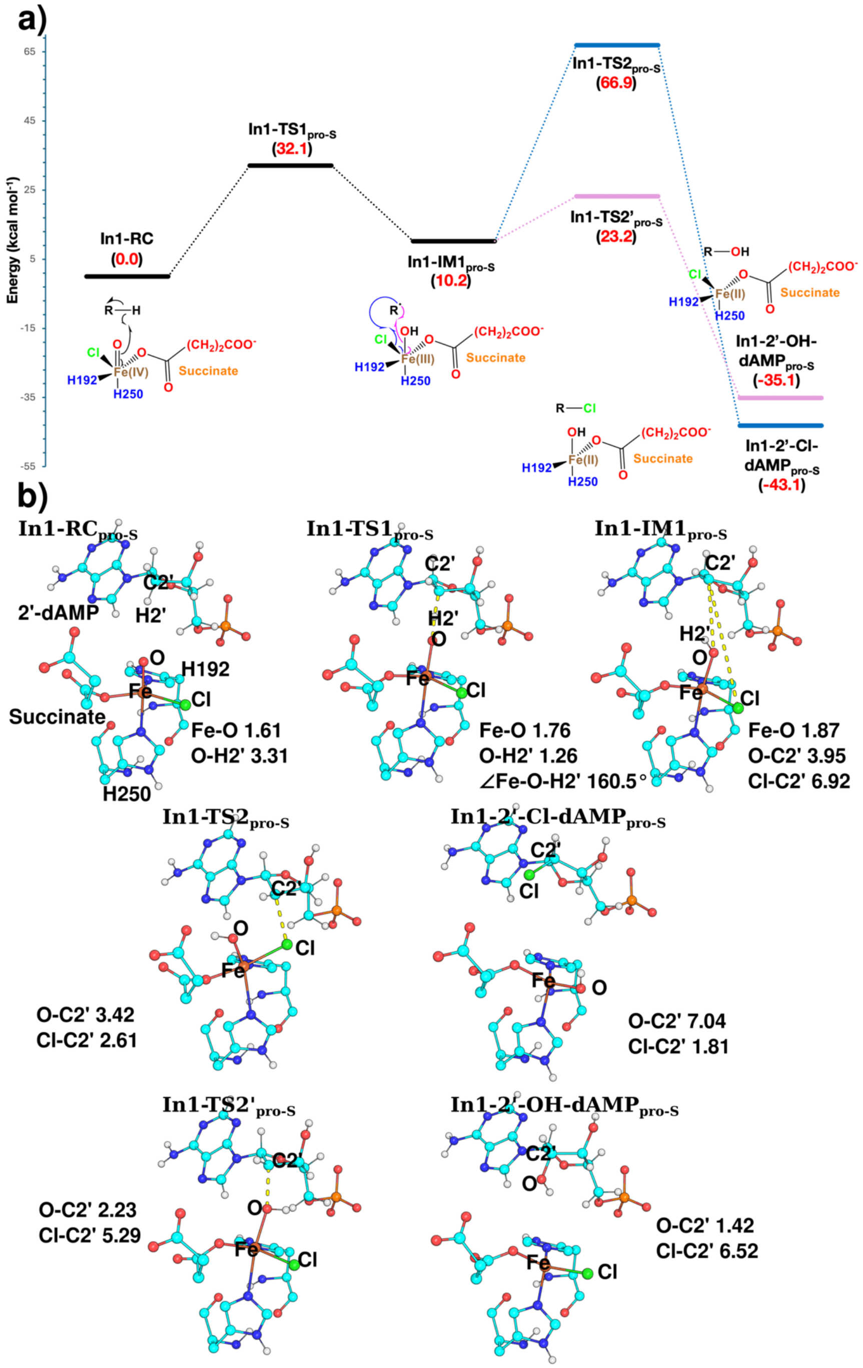

Figure S46. Reaction profile and representation of QM/MM optimized structures of halogenation and hydroxylation mechanism in the Inline ferryl system. a) Reaction profile of halogenation and hydroxylation mechanism in the Inline ferryl system. Relative energies are given in kcal/mol calculated at zero-point corrected energies (QM(B3)/MM level). b) Representations of QM/MM optimized structures obtained during the halogenation and hydroxylation in Inline ferryl. Non-polar hydrogens are hidden except for the substrate for clarity. Distances are mentioned in Å.

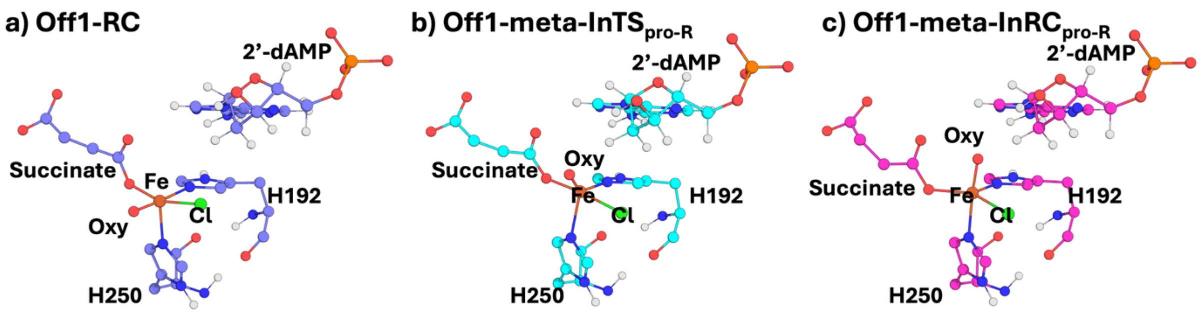

Figure S47. The change from offline to inline orientation during HAT a) Off1-RC, b) Off1-InTS, c) Off1-InRC.

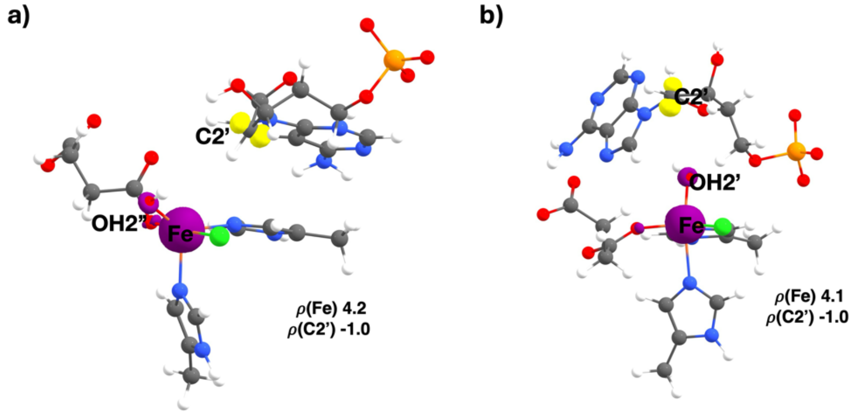

Figure S48. The spin density plots of a) Off1-IM1 and b) In1-IM1. Purple isosurface (Isovalue = 0.05) indicates positive spin density, and yellow indicates negative spin density.

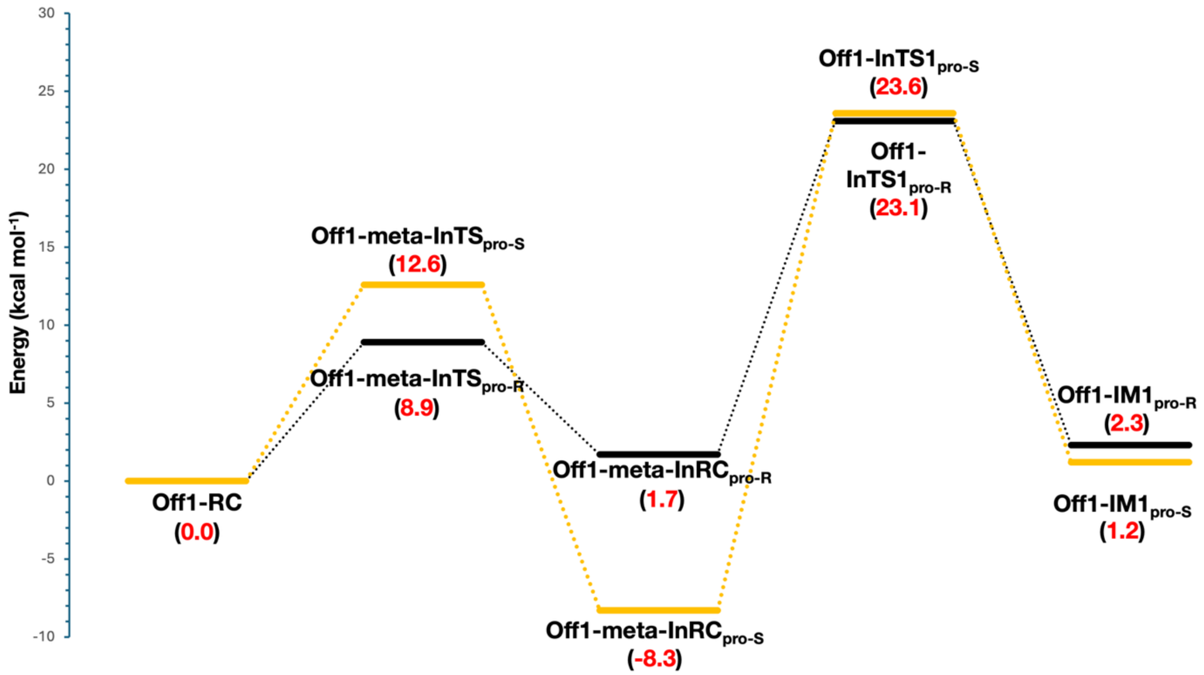

Figure S49. Reaction profile of Pro-R and Pro-S HAT from Off1-RC. Relative energies are given in kcal/mol calculated at zero-point corrected energies (QM(B3)/MM level).

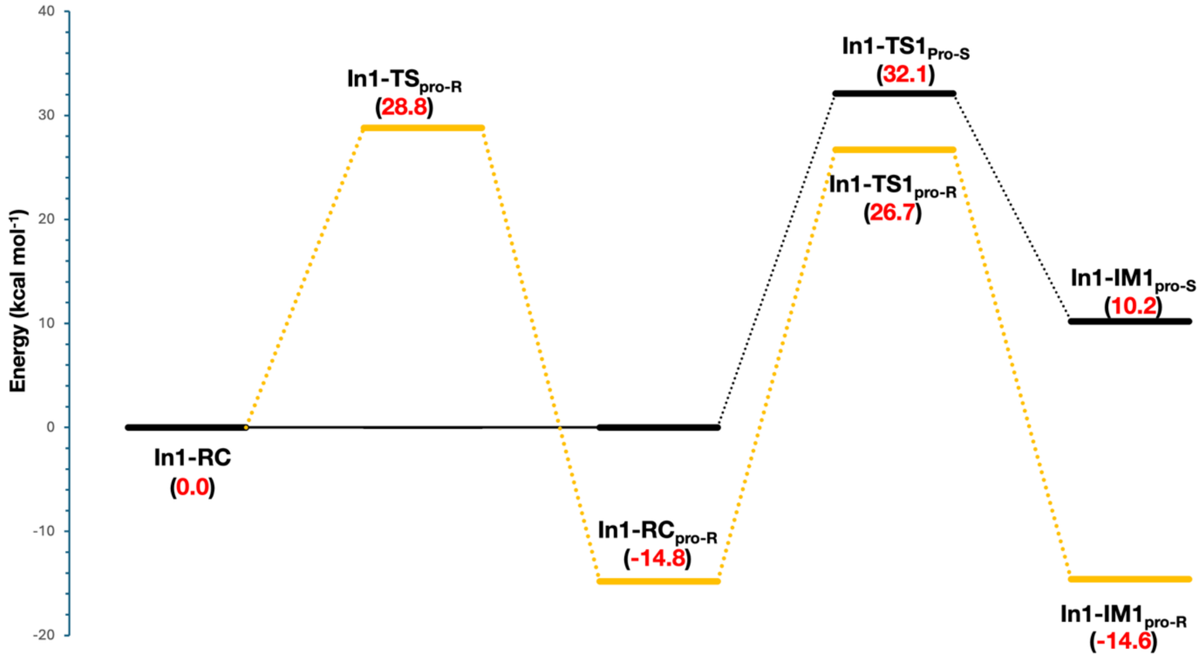

Figure S50. Reaction profile of Pro-R and Pro-S HAT from In1-RC. Relative energies are given in kcal/mol calculated at zero-point corrected energies (QM(B3)/MM level).

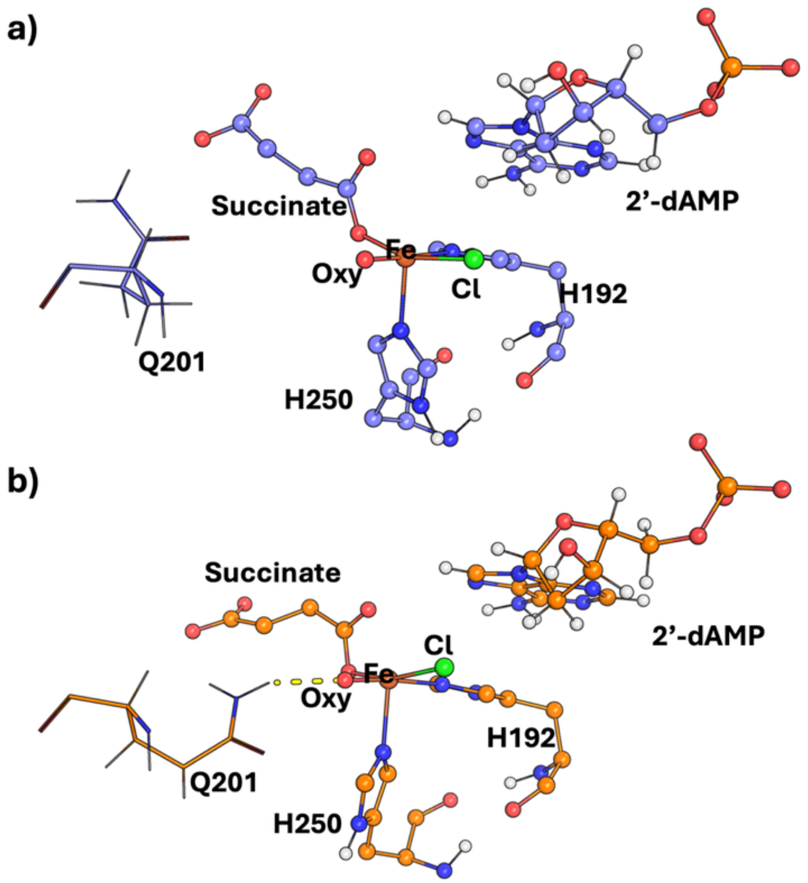

Figure S51. QM/MM optimized structures of initial RCs obtained from the offline ferryl system. a) Off1-RC, b) Off2-RC. Yellow dotted lines denote hydrogen bonds.

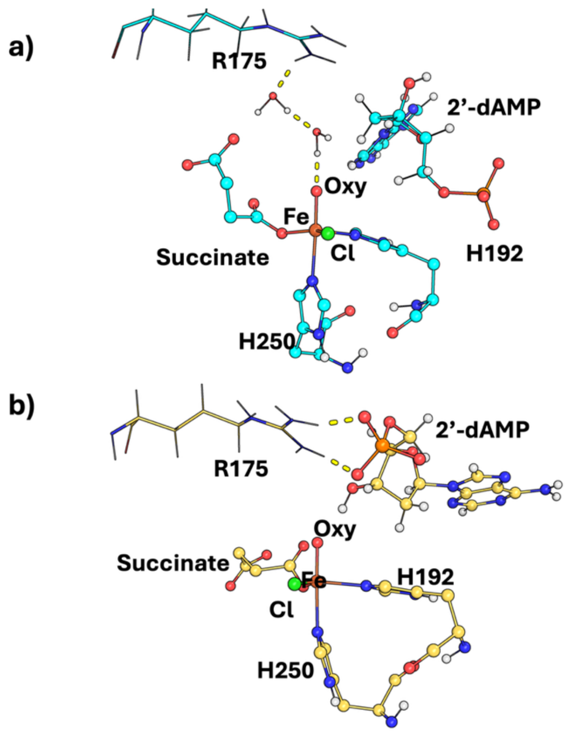

Figure S52. QM/MM optimized structures of initial RCs obtained from the inline ferryl system. a) In1-RC, b) In2-RC. Yellow dotted lines denote hydrogen bonds.

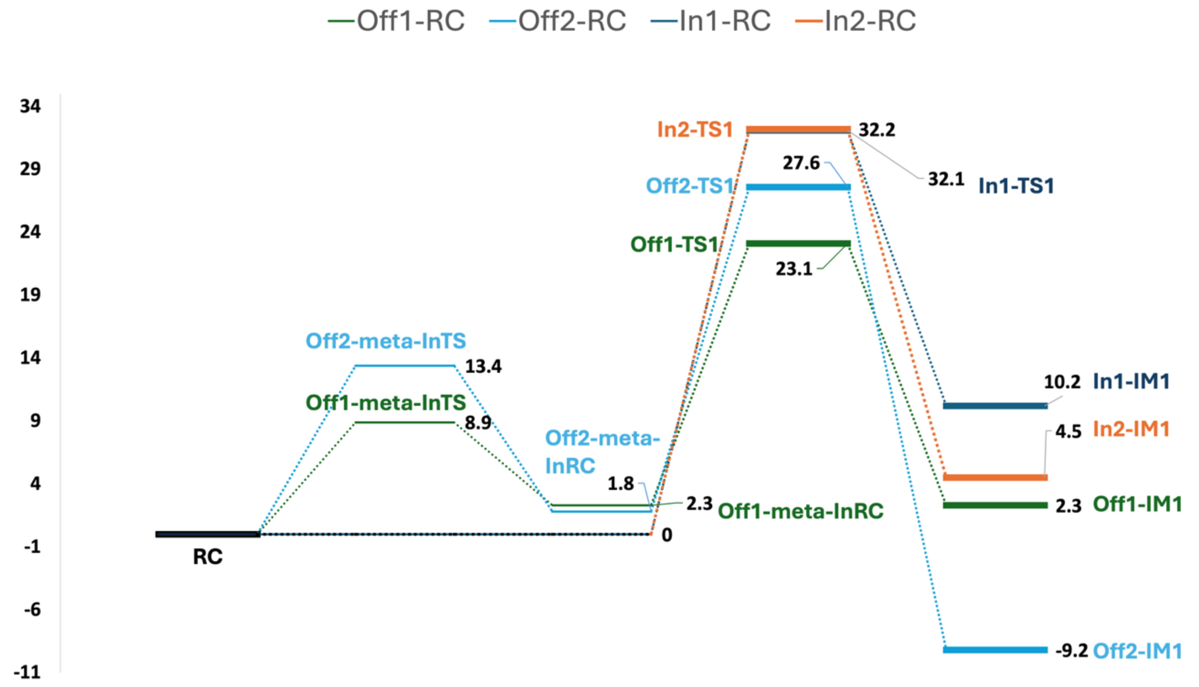

Figure S53. Potential Energy Surface of the HAT reaction obtained from QM/MM simulations on offline and inline ferryl systems. Relative energies are given in kcal/mol calculated at zero-point corrected energies (QM(B3)/MM level).

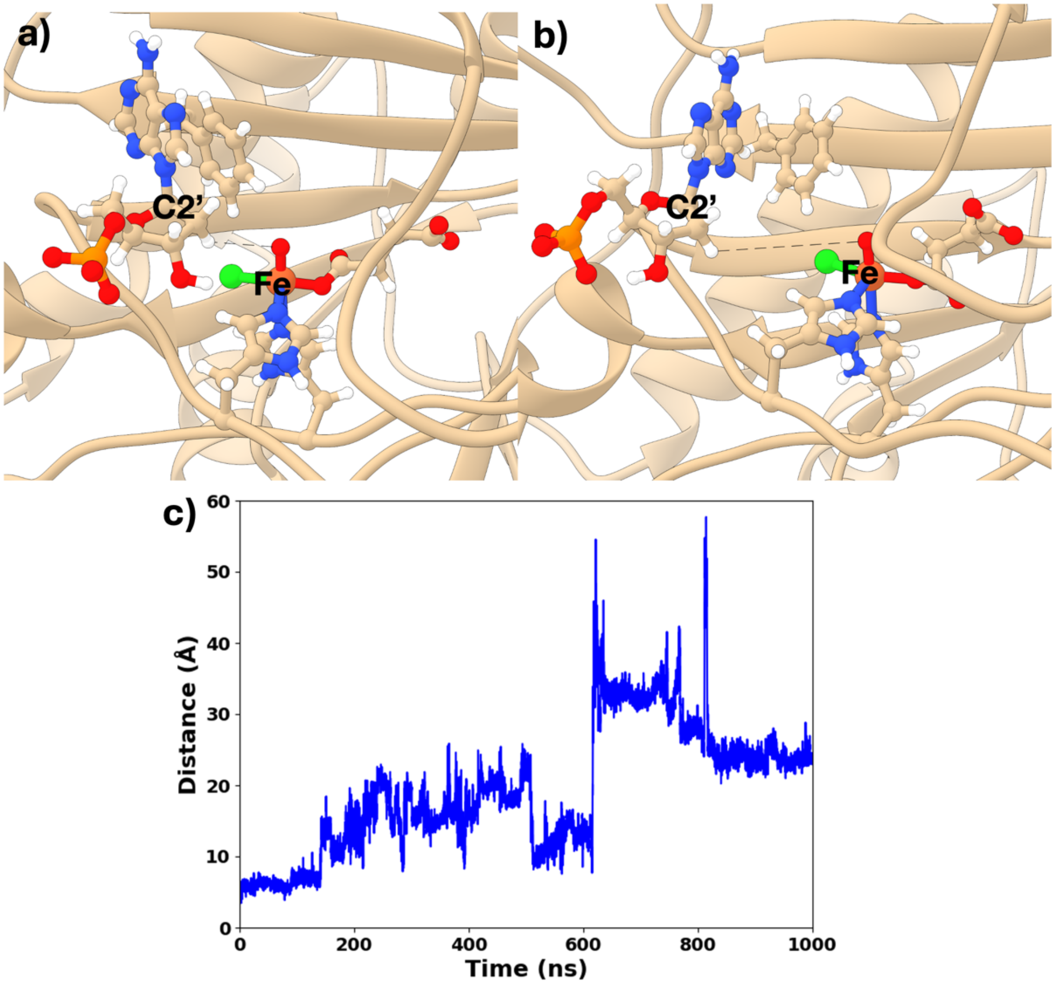

Figure S54. Alternative substrate binding configuration. (a) The initial structure of the production MD trajectory, (b) the snapshot obtained after 150ns of the MD trajectory, (c) Time evolution of the distance between Ferryl oxygen and C2' carbon of 2'-dAMP.

Supplementary Tables

Table S1.
Mössbauer parameters of the S = 2 ferryl intermediates.

**Species**	**D (cm-1)***	**E/D***	**A (T)**	**ΔE_Q (mm/s)**	**δ (mm/s)**
Fe(IV)=O^{1st}	10	0	-18, -18, -30	-0.94	0.23
Fe(IV)=O^{2nd}	10	0	-17.3, -17.3, -30	-0.55	0.18

* the parameter is fixed during the simulation.

Table S2.
The time-dependent iron speciation and the chlorinated product **2** in the AdeV reaction in the presence of Cl^- and 1.*

**Reaction Time (s)**	**ES complex (%)**	**Fe^{IV}=O^{1st} (%)**	**Fe^{IV}=O^{2nd} (%)**	**Fe(III) (%)**	**Product 2 (%)**
0	100	0	0	0	0
1	77	20	0	≤ 3	4
10	43	45	0	11	25
30	50	26	13	10	50
60	65	12	16	10	66
100	70	5	15	12	66
500	74	0	15	12	66

*The percentage values listed here are relative to the concentration of the Fe(II) loaded AdeV.

Table S3.
Kinetic parameters used in the kinetic model shown in the main text.

K_d (mM)	k_2 (mM^{-1} s^{-1})	k_3 (s^{-1})	k_4 (s^{-1})	k_5 (s^{-1})	k_{-5} (s^{-1})	k_x (mM^{-1} s^{-1})
28	~1.8	0.05	fast	0.015	≤ 0.001	0.25

Table S4 Comparison of ^{1}H NMR spectroscopic data of enzymatic and synthetic product ((*2R,3R,4R,5R*)-5-(6-amino-9*H*-purin-9-yl)-4-chloro-3-hydroxytetra-hydrofuran-2-yl)methyl dihydrogen phosphate

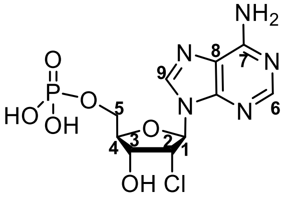

Position	**Enzymatic product**<br>δ ^{1}H [ppm; mult; J (Hz)]<br>700 MHz	**Synthetic standard**<br>δ ^{1}H [ppm; mult; J (Hz)]<br>500 MHz	**deviation**<br>(Enzymatic–synthetic) Δδ (ppm)
9	8.51, s	8.48, s	0.03
6	8.27, s	7.99, s	0.28
1	6.35, d, *J* = 6.1 Hz	6.17, d, *J* = 5.9 Hz	0.18
2	4.98 t, *J* = 5.4 Hz	4.88 (t, *J* = 5.5 Hz	0.1
3	4.47 p, *J* = 2.6 Hz	4.39 (d, *J* = 4.2 Hz	0.08
4	4.69 t, *J* = 4.8 Hz,	4.63 (t, *J* = 4.6 Hz,	0.06
5	4.20, ddd, *J* = 11.6, 4.6, 2.7 Hz<br>4.15, ddd, *J* = 11.8, 4.9, 2.8 Hz	4.04, dt, *J* = 12.2, 3.6 Hz<br>4.00, dt, *J* = 7.4, 6.0 Hz	0.16<br>0.15

Table S5 Comparison of ^{13}C NMR spectroscopic data of enzymatic and synthetic product ((*2R,3R,4R,5R*)-5-(6-amino-*9H*-purin-9-yl)-4-chloro-3-hydroxytetra-hydrofuran-2-yl)methyl dihydrogen phosphate

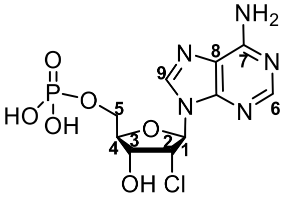

Position	**Enzymatic product**<br>δ ^{1}H [ppm; mult; J (Hz)]<br>125 MHz	**Synthetic standard**<br>δ ^{1}H [ppm; mult; J (Hz)]<br>175 MHz	**deviation**<br>(Enzymatic–synthetic) Δδ (ppm)
10	148.9	148.4	0.5
9	139.9	139.6	0.3
8	118.7	118.1	0.6
7	155.1	155.1	0
6	152.1	152.5	-0.4
1	88.1	87.9	0.2
2	61.5	61.8	-0.3
3	70.3	70.4	-0.1
4	84.0	84.4	-0.4
5	63.9	62.9	1

Table S6. Tabulated the B3 energies obtained from normal QM (QM1) and extended QM (QM2) regions in Off2-RC.

Reaction States	Absolute Energies (QM(B3)/MM) a.u.	Relative Energies (QM(B3)/MM) Kcal/mol	Extended QM Absolute Energies (QM(B3(D3))/MM) a.u.	Extended QM Relative Energies (QM(B3(D3))/MM) Kcal/mol
**Off2-RC**	-4322.92432671152	0.0	-4571.62138505181	0.0
**Off2-meta-InTS**	**-4322.90294996493**	**13.4**	**-4571.60335920455**	**11.3**
**Off2-meta-InRC**	-4322.92144812357	1.8	-4571.62045316849	0.6
**Off2-InTS1**	**-4322.88022998958**	**27.7**	**-4571.58195544695**	**24.7**
**Off2-IM1**	-4322.93902298275	-9.2	-4571.65402223124	-20.5

Table S7. Tabulated the B3 energies and also the B3 energies with D3 dispersion correction in the Off1-RC snapshot.

Reaction States	Absolute Energies (QM(B3)/MM) a.u.	Relative Energies (QM(B3)/MM) Kcal/mol	Absolute Energies (QM(B3(D3))/MM) a.u.	Relative Energies (QM(B3(D3))/MM) Kcal/mol
**Off1-RC**	-4322.170333	0.0	-4322.34938	0.0
**Off1-meta-InTS**	-4322.156171	8.9	-4322.339549	6.2
**Off1-meta-InRC**	-4322.167662	1.7	-4322.346826	1.6
**Off1-InTS1**	-4322.133585	23.1	-4322.314465	21.9
**Off1-IM1**	-4322.166632	2.3	-4322.343986	3.4
**Off1-TS2**	-4322.154842	9.7	-4322.334744	9.2
**Off1-2'-Cl-dAMP**	-4322.205824	-22.3	-4322.382635	-20.9
**Off1-TS2'**	-4322.147928	14.1	-4322.328785	12.9
**Off1-2'-OH-dAMP**	-4322.228748	-36.7	-4322.409046	-37.4

Table S8. Tabulated the B3 energies and also the B3 energies with D3 dispersion correction in the In1-RC snapshot.

Reaction States	Absolute Energies (QM(B3)/MM) a.u.	Relative Energies (QM(B3)/MM) Kcal/mol	Absolute Energies (QM(B3(D3))/MM) a.u.	Relative Energies (QM(B3(D3))/MM) Kcal/mol
**In1-RC**	-4322.024596	0.0	-4322.77	0.0
**In1-TS1**	-4321.97336	32.1	-4290.58	28.2
**In1-IM1**	-4322.008256	10.2	-4312.51	10.0
**In1-TS2**	-4321.917946	66.9	-4255.76	58.5
**In1-2'-Cl-dAMP**	-4322.093388	-43.1	-4366.01	-39.8
**In1-TS2'**	-4321.987591	23.2	-4299.52	18.8
**In1-2'-OH-dAMP**	-4322.080609	-35.1	-4357.99	-34.5

Table S9. Tabulated the B2 SP energies and the B2 Optimized energies from the Off1-RC snapshot

Reaction States	Absolute Energies (SP at def2-TZVP) (QM(B2)/MM) a.u.	Relative Energies SP at def2-TZVP (QM(B2)/MM) Kcal/mol	Absolute Energies Optimized at def2-TZVP (QM(B2-Opt)/MM) a.u.	Relative Energies Optimized at def2-TZVP (QM(B2-Opt)/MM) Kcal/mol
**Off1-RC**	-4322.717226	0.0	-4322.72507529332	0.0
**Off1-meta-InTS**	-4322.702462	9.3	-4322.70246217829	14.2
**Off1-meta-InRC**	-4322.713531	2.3	-4322.72165421559	2.1
**Off1-InTS1**	-4322.673607	27.4	-4322.68054114299	27.9
**Off1-IM1**	-4322.714699	1.6	-4322.72301025357	1.3

Table S10. Spin Densities for stationary points obtained from the O2 activation reaction from In-SO-RC.

**Spin Density**	**In-SO-RC**	**In-SO-TS1**	**In-SO-IM1**	**In-SO-TS2**	**In-SO-IM2**	**In-SO-PD**
FE1	4.12	4.21	3.79	3.94	4.11	3.13
Op	-0.14	-0.27	0.07	0.07	-0.16	0.67
Od	-0.44	-0.30	0.00	-0.22	0.27	0.01
2OG	0.20	0.06	0.03	0.05	0.09	0.07
Cl	0.14	0.15	0.05	0.07	0.10	0.06
H192-N	0.05	0.06	0.01	0.03	0.04	-0.03
H250-N	0.06	0.07	0.02	0.03	0.05	0.07
2'-dAMP	0.00	0.00	0.00	0.00	0.00	0.00

Table S11. Charges for stationary points obtained from the O2 activation reaction from In-SO-RC.

**Mulliken Charges**	**In-SO-RC**	**In-SO-TS1**	**In-SO-IM1**	**In-SO-TS2**	**In-SO-IM2**	**In-SO-PD**
FE1	0.85	0.86	0.76	0.82	0.86	0.81
Op	-0.18	-0.22	-0.40	-0.39	-0.40	-0.39
Od	-0.12	-0.09	-0.12	-0.19	-0.21	-0.27
2OG	-1.50	-1.55	-0.97	-1.05	-1.15	-1.18
Cl	-0.46	-0.46	-0.56	-0.54	-0.51	-0.43
H192-N	0.27	0.29	0.22	0.24	0.27	0.29
H250-N	0.27	0.31	0.21	0.24	0.27	0.31
2'-dAMP	-2.05	-2.04	-2.05	-2.04	-2.04	-2.05

Table S12. Spin Densities for stationary points obtained from the O2 activation reaction from Off-SO-RC.

**Spin Density**	**Off-SO-RC**	**Off-SO-TS1**	**Off-SO-IM1**	**Off-SO-TS2**	**Off-SO-IM2**	**Off-SO-PD**
FE1	4.11	4.18	3.78	3.99	4.06	3.04

Op	-0.14	0.00	0.08	0.07	-0.08	0.71
Od	-0.40	-0.23	0.00	-0.29	-0.27	0.05
2OG	0.20	-0.20	0.03	0.07	0.11	0.09
Cl	0.11	0.12	0.04	0.06	0.09	0.06
H192-N	0.04	0.05	0.01	0.02	0.02	-0.02
H250-N	0.04	0.07	0.02	0.05	0.07	0.05
2'-dAMP	0.00	0.00	0.00	0.00	0.00	0.00

Table S13. Charges for stationary points obtained from the O2 activation reaction from Off-SO-RC.

**Mulliken Charges**	**Off-SO-RC**	**Off-SO-TS1**	**Off-SO-IM1**	**Off-SO-TS2**	**Off-SO-IM2**	**Off-SO-PD**
FE1	0.85	0.86	0.77	0.84	0.86	0.79
Op	-0.17	-0.28	-0.41	-0.40	-0.41	-0.33
Od	-0.11	-0.10	-0.11	-0.18	-0.18	-0.22
2OG	-1.47	-1.42	-0.93	-1.04	-1.10	-1.18
Cl	-0.53	-0.53	-0.64	-0.60	-0.57	-0.54
H192-N	0.22	0.23	0.18	0.19	0.16	0.22
H250-N	0.29	0.30	0.24	0.29	0.32	0.36
2'-dAMP	-2.01	-2.01	-2.03	-2.03	-2.03	-2.03

Table S14. Spin Densities for stationary points obtained from the reaction path calculations from Off1-RC.

**Spin Density**	**Off1-RC**	**Off1-meta-InTS**	**Off1-meta-InRC**	**Off1-InTS1**	**Off1-IM1**	**Off1-TS2**	**Off1-2-Cl-dAMP**	**Off1-TS2'**	**Off1-2-OH-dAMP**
**Fe**	3.16	3.16	3.03	3.98	4.21	4.10	3.82	4.08	3.83
**O**	0.65	0.69	0.74	0.09	0.28	0.18	0.08	0.36	0.01
**Succinate**	0.04	0.03	0.11	0.11	0.21	0.12	0.03	0.12	0.06

**Cl**	0.07	0.00	0.07	0.13	0.12	0.15	0.00	0.10	0.04
**H192-N**	-0.02	0.04	0.06	0.07	0.08	0.05	0.01	0.05	0.02
**H250-N**	0.09	0.06	-0.02	0.03	0.08	0.05	0.03	0.03	0.03
**2'-dAMP-C2'**	0.00	0.00	0.00	-0.42	-0.97	-0.68	0.00	-0.77	0.00

Table S15. Charges for stationary points obtained from the reaction path calculations from Off1-RC.

**Mulliken Charges**	**Off1-RC**	**Off1-meta-InTS**	**Off1-meta-InRC**	**Off1-InTS1**	**Off1-IM1**	**Off1-TS2**	**Off1-2-Cl-dAMP**	**Off1-TS2'**	**Off1-2-OH-dAMP**
**Fe**	0.78	0.77	0.77	0.87	0.88	0.84	0.77	0.86	0.81
**O**	-0.52	-0.41	-0.32	-0.44	-0.55	-0.58	-0.72	-0.45	-0.31
**Succinate**	-1.43	-1.40	-1.49	-1.52	-1.47	-1.53	-1.63	-1.57	-1.61
**Cl**	-0.37	-0.40	-0.45	-0.50	-0.52	-0.40	-0.12	-0.57	-0.62
**H192**	0.28	0.28	0.28	0.26	0.27	0.23	0.19	0.22	0.20
**H250**	0.32	0.26	0.26	0.20	0.30	0.26	0.21	0.19	0.19
**2'-dAMP**	-2.01	-2.05	-2.02	-1.82	-1.86	-1.76	-1.64	-1.61	-1.61

Table S16. Spin Densities for stationary points obtained from the reaction path calculations from In1-RC.

**Spin Density**	**In1-RC**	**In1-TS1**	**In1-IM1**	**In1-TS2**	**In1-2'-Cl-dAMP**	**In1-TS2'**	**In1-2'-OH-dAMP**
**Fe**	3.09	3.97	4.18	4.12	3.79	4.04	3.79
**O**	0.65	0.03	0.30	0.35	0.09	0.20	0.00
**Succinate**	0.12	0.19	0.21	0.14	0.06	0.17	0.08
**Cl**	0.07	0.11	0.14	0.13	0.00	0.09	0.06
**H192-N**	0.07	0.08	0.09	0.06	0.01	0.05	0.02

**H250-N**	-0.03	0.03	0.04	0.03	0.02	0.03	0.02
**2'-dAMP-C2'**	0.00	-0.42	-0.97	-0.84	0.00	-0.60	0.00

Table S17. Charges for stationary points obtained from the reaction path calculations from In1-RC.

**Mulliken Charges**	**In1-RC**	**In1-TS1**	**In1-IM1**	**In1-TS2**	**In1-2'-Cl-dAMP**	**In1-TS2'**	**In1-2'-OH-dAMP**
**Fe**	0.81	0.92	0.91	0.84	0.87	0.77	0.78
**O**	-0.43	-0.53	-0.54	-0.53	-0.47	-0.69	-0.36
**Succinate**	-1.48	-1.50	-1.49	-1.48	-1.52	-1.61	-1.57
**Cl**	-0.49	-0.50	-0.52	-0.40	-0.56	-0.12	-0.59
**H192**	0.27	0.27	0.24	0.24	0.23	0.18	0.16
**H250**	0.29	0.20	0.21	0.19	0.16	0.21	0.21
**2'-dAMP**	-1.92	-1.78	-1.75	-1.80	-1.65	-1.68	-1.57

Table S18. Spin Densities for stationary points obtained from the reaction path calculations from Off2-RC.

**Spin Density**	**Off2-RC**	**Off2-meta-InTS**	**Off2-meta-InRC**	**Off2-InTS1**	**Off2-IM1**	**Off2-TS2**	**Off2-2-Cl-dAMP**	**Off2-TS2'**	**Off2-2-OH-dAMP**
**Fe**	3.15	3.18	3.07	3.98	4.20	4.01	3.81	4.04	3.82
**O**	0.61	0.66	0.74	0.10	0.33	0.18	0.09	0.34	0.00
**Succinate**	0.12	0.03	0.08	0.12	0.17	0.10	0.05	0.10	0.06
**Cl**	0.08	0.00	0.04	0.12	0.12	0.10	0.00	0.09	0.05
**H192-N**	-0.03	0.05	0.08	0.07	0.06	0.02	0.02	0.05	0.02
**H250-N**	0.05	0.06	-0.02	0.03	0.07	0.04	0.02	0.03	0.02
**2'-dAMP-C2'**	0.00	0.00	0.00	-0.42	-0.98	-0.44	0.00	-0.63	0.00

Table S19. Charges for stationary points obtained from the reaction path calculations from Off2-RC.

Mulliken Charges	Off2-RC	Off2-meta-InTS	Off2-meta-InRC	Off2-InTS1	Off2-IM1	Off2-TS2	Off2-2-Cl-dAMP	Off2-TS2'	Off2-2-OH-dAMP
**Fe**	0.75918	0.78567	0.90371	0.90305	0.80288	0.826	0.8712	0.7808	0.7823
**O**	-0.45536	-0.39491	-0.45477	-0.51777	-0.34144	-0.61842	-0.41423	-0.69872	-0.31692
**Succinate**	-1.36473	-1.39423	-1.49039	-1.48204	-1.43086	-1.52058	-1.55129	-1.57586	-1.52624
**Cl**	-0.39563	-0.42359	-0.516	-0.56343	-0.457	-0.36032	-0.56912	-0.12611	-0.51538
**H192**	0.25572	0.27717	0.2622	0.25262	0.25895	0.19428	0.23406	0.16686	0.19441
**H250**	0.29235	0.25847	0.20082	0.29141	0.26093	0.22818	0.2005	0.19659	0.21338
**2'-dAMP**	-2.02847	-2.04474	-1.83943	-1.82649	-2.03258	-1.67959	-1.70435	-1.67942	-1.7686

Table S20. Spin Densities for stationary points obtained from the reaction path calculations from In2-RC.

Spin Density	In2-RC	In2-TS1	In2-IM1	In2-TS2	In2-2'-Cl-dAMP	In2-TS2'	In2-2'-OH-dAMP
**Fe**	3.11371	4.01609	4.1848	4.02398	3.79122	4.0736	3.79411
**O**	0.61879	-0.0845	0.26071	0.22922	0.0763	0.21297	0
**Succinate**	0.13988	0.15986	0.24531	0.17164	0.05878	0.17165	0.07993
**Cl**	0.0735	0.17236	0.13311	0.08153	0	0.11498	0.05725
**H192-N**	0.06868	0.07417	0.08351	0.05735	0.03035	0.06042	0.02728
**H250-N**	-0.02922	0.04084	0.04573	0.04393	0.01774	0.03204	0.02045
**2'-dAMP-C2'**	0	-0.39955	-0.96746	-0.64079	0	-0.68611	0

Table S21. Charges for stationary points obtained from the reaction path calculations from In2-RC.

**Mulliken Charges**	**In2-RC**	**In2-TS1**	**In2-IM1**	**In2-TS2**	**In2-2'-Cl-dAMP**	**In2-TS2'**	**In2-2'-OH-dAMP**
**Fe**	0.78715	0.86562	0.87959	0.83906	0.78714	0.83212	0.75716
**O**	-0.42201	-0.51297	-0.49515	-0.56617	-0.67162	-0.43836	-0.31124
**Succinate**	-1.4178	-1.44303	-1.45574	-1.50449	-1.6235	-1.50127	-1.58835
**Cl**	-0.42538	-0.43794	-0.49514	-0.34563	-0.10904	-0.49089	-0.52435
**H192**	0.30001	0.2757	0.29447	0.25872	0.1856	0.27496	0.19364
**H250**	0.27868	0.22725	0.24115	0.25023	0.19947	0.19651	0.21805
**2'-dAMP**	-2.06851	-1.94455	-1.93618	-1.90012	-1.72988	-1.84205	-1.71175

Table S22. Tabulated the calculated Mössbauer parameters and associated spin densities of the different models of Off1-RC snapshot.

**Off1-RC**	**QM**	**QM/MM**	**QM with 3 H_2O added**	**QM/MM with 3 H_2O added**	**QM with 4 H_2O added**	**QM/MM with 4 H_2O added**	**Optimized with 4 H_2O added QM**	**Optimized with 4 H_2O added QM/MM**
**ρ**	11816.75039968	11816.78720616	11817.06660533	11817.13638574	11817.15019906	11817.23012865	11817.11044976	11817.19248787
**Isomer Shift (δ)**	0.38	0.37	0.27	0.24	0.24	0.21	0.25	0.22
**Quadrupole Splitting (ΔE_Q)**	1.78	1.74	0.58	-0.72	-0.97	-1.25	-0.75	-1.01
**Spin Densities**								
**Fe**	2.72	2.73	3.00	3.00	3.07	3.1	3.04	3.08
**O**	0.49	0.5	0.60	0.62	0.63	0.66	0.61	0.64
**PO3**	0.74	0.71	0.29	0.21	0.17	0.07	0.23	0.13

Table S23. Tabulated the Mössbauer parameters and associated spin densities of the different models of In1-RC snapshot.

**In1-RC**	**QM**	**QM/MM**	**QM with 3 H_2O added**	**QM/MM with 3 H_2O added**	**QM with 4 H_2O added**	**QM/MM with 4 H_2O added**	**Optimized with 4 H_2O added QM**	**Optimized with 4 H_2O added QM/MM**
**ρ**	11816.96	11816.94	11817.208	11817.2292	11817.213	11817.23521	11817.2109	11817.229

	9605915	1144165	792601	74428	492877	6125	28189	007047
**Isomer Shift (δ)**	0.30	0.31	0.21	0.21	0.21	0.20	0.21	0.21
**Quadrupole Splitting (ΔE_Q)**	0.83	1.26	-0.86	-0.73	-0.86	-0.73	-0.85	-0.71
**Spin Densities**								
**Fe**	2.94	2.79	3.11	3.02	3.11	3.02	3.11	3.03
**O**	0.54	0.51	0.63	0.63	0.63	0.63	0.63	0.62
**PO3**	0.34	0.5	0	0	0	0	0	0

References:

(1) Marriott, J. H.; Mottahedeh, M.; Reese, C. B. Synthesis of 2′-Thioadenosine. *Carbohydr. Res.* **1992**, *216*, 257–269. https://doi.org/10.1016/0008-6215(92)84167-Q.

(2) Kai, K.; Fujii, H.; Ikenaka, R.; Akagawa, M.; Hayashi, H. An Acyl-SAM Analog as an Affinity Ligand for Identifying Quorum Sensing Signal Synthases. *Chem. Commun.* **2014**, *50* (62), 8586–8589. https://doi.org/10.1039/C4CC03094J.

(3) Petasis, D. T.; Hendrich, M. P. *Methods Enzymol.* **2015**, 563, 171.

(4) Zhai, G.; Gong, R.; Lin, Y.; Zhang, M.; Li, J.; Deng, Z.; Sun, J.; Chen, W.; Zhang, Z. Structural Insight into the Catalytic Mechanism of Non-Heme Iron Halogenase AdaV in 2′-Chloropentostatin Biosynthesis. *ACS Catal.* **2022**, *12* (22), 13910–13920. https://doi.org/10.1021/acscatal.2c04608.

(5) Wang, J.; Wolf, R. M.; Caldwell, J. W.; Kollman, P. A.; Case, D. A. Development and Testing of a General Amber Force Field. *J. Comput. Chem.* **2004**, *25* (9), 1157–1174. https://doi.org/10.1002/jcc.20035.

(6) Case, D. A.; Belfon, K.; Ben-Shalom, I. Y.; Brozell, S. R.; Cerutti, D. S.; Cheatham, T. E.; Cruzeiro, V. W. D.; Darden, T. A.; Duke, R. E.; Giambasu, G.; Gilson, M. K.; Gohlke, H.; Goetz, A. W.; Harris, R.; Izadi, S.; Izmailov, S. A.; Kasavajhala, K.; Kovalenko, A.; Krasny, R.; Kurtzman, T.; Lee, T. S.; LeGrand, S.; Li, P.; Lin, C.; Liu, J.; Luchko, T.; Luo, R.; Man, V.; Merz, K. M.; Miao, Y.; Mikhailovskii, O.; Monard, G.; Nguyen, H.; Onufriev, A.; Pan, F.; Pantano, S.; Qi, R.; Roe, D. R.; Roitberg, A.; Sagui, C.; Schott-Verdugo, S.; Shen, J.; Simmerling, C. L.; Skrynnikov, N. R.; Smith, J.; Swails, J.; Walker, R. C.; Wang, J.; Wilson, L.; Wolf, R. M.; Wu, X.; Xiong, Y.; Xue, Y.; York, D. M.; Kollman, P. A. AMBER 2020, 2020.

(7) Li, P.; Merz, K. M. MCPB.Py: A Python Based Metal Center Parameter Builder. *J. Chem. Inf. Model.* **2016**, *56* (4), 599–604. https://doi.org/10.1021/acs.jcim.5b00674.

(8) Maier, J. A.; Martinez, C.; Kasavajhala, K.; Wickstrom, L.; Hauser, K. E.; Simmerling, C. ff14SB: Improving the Accuracy of Protein Side Chain and Backbone Parameters from ff99SB. *J. Chem. Theory Comput.* **2015**, *11* (8), 3696–3713. https://doi.org/10.1021/acs.jctc.5b00255.

(9) Jorgensen, W. L.; Chandrasekhar, J.; Madura, J. D.; Impey, R. W.; Klein, M. L. Comparison of Simple Potential Functions for Simulating Liquid Water. *J. Chem. Phys.* **1983**, *79* (2), 926–935. https://doi.org/10.1063/1.445869.

(10) Pettersen, E. F.; Goddard, T. D.; Huang, C. C.; Couch, G. S.; Greenblatt, D. M.; Meng, E. C.; Ferrin, T. E. UCSF Chimera—A Visualization System for Exploratory Research and Analysis. *J. Comput. Chem.* **2004**, *25* (13), 1605–1612. https://doi.org/10.1002/jcc.20084.

(11) Davidchack, R. L.; Handel, R.; Tretyakov, M. V. Langevin Thermostat for Rigid Body Dynamics. *J. Chem. Phys.* **2009**, *130* (23), 234101. https://doi.org/10.1063/1.3149788.

(12) Liu, J.; Li, D.; Liu, X. A Simple and Accurate Algorithm for Path Integral Molecular Dynamics with the Langevin Thermostat. *J. Chem. Phys.* **2016**, *145* (2), 024103. https://doi.org/10.1063/1.4954990.

(13) Darden, T.; York, D.; Pedersen, L. Particle Mesh Ewald: AnN·log(N) Method for Ewald Sums in Large Systems. *J. Chem. Phys.* **1993**, *98* (12), 10089–10092. https://doi.org/10.1063/1.464397.
(14) Salomon-Ferrer, R.; Götz, A. W.; Poole, D.; Le Grand, S.; Walker, R. C. Routine Microsecond Molecular Dynamics Simulations with AMBER on GPUs. 2. Explicit Solvent Particle Mesh Ewald. *J. Chem. Theory Comput.* **2013**, *9* (9), 3878–3888. https://doi.org/10.1021/ct400314y.
(15) Miyamoto, S.; Kollman, P. A. Settle: An Analytical Version of the SHAKE and RATTLE Algorithm for Rigid Water Models. *J. Comput. Chem.* **1992**, *13* (8), 952–962. https://doi.org/10.1002/jcc.540130805.
(16) Bussi, G.; Donadio, D.; Parrinello, M. Canonical Sampling through Velocity Rescaling. *J. Chem. Phys.* **2007**, *126* (1), 014101. https://doi.org/10.1063/1.2408420.
(17) Roe, D. R.; Cheatham, T. E. PTRAJ and CPPTRAJ: Software for Processing and Analysis of Molecular Dynamics Trajectory Data. *J. Chem. Theory Comput.* **2013**, *9* (7), 3084–3095. https://doi.org/10.1021/ct400341p.
(18) Grant, B. J.; Rodrigues, A. P. C.; ElSawy, K. M.; McCammon, J. A.; Caves, L. S. D. Bio3d: An R Package for the Comparative Analysis of Protein Structures. *Bioinformatics* **2006**, *22* (21), 2695–2696. https://doi.org/10.1093/bioinformatics/btl461.
(19) Metz, S.; Kästner, J.; Sokol, A. A.; Keal, T. W.; Sherwood, P. ChemShell—a Modular Software Package for QM/MM Simulations. *WIREs Comput Mol Sci* **2014**, *4* (2), 101–110. https://doi.org/10.1002/wcms.1163.
(20) Smith, W.; Yong, C. W.; Rodger, P. M. DL_POLY: Application to Molecular Simulation. *Mol Simul* **2002**, *28* (5), 385–471.
(21) Balasubramani, S. G.; Chen, G. P.; Coriani, S.; Diedenhofen, M.; Frank, M. S.; Franzke, Y. J.; Furche, F.; Grotjahn, R.; Harding, M. E.; Hättig, C.; Hellweg, A.; Helmich-Paris, B.; Holzer, C.; Huniar, U.; Kaupp, M.; Marefat Khah, A.; Karbalaei Khani, S.; Müller, T.; Mack, F.; Nguyen, B. D.; Parker, S. M.; Perlt, E.; Rappoport, D.; Reiter, K.; Roy, S.; Rückert, M.; Schmitz, G.; Sierka, M.; Tapavicza, E.; Tew, D. P.; van Wüllen, C.; Voora, V. K.; Weigend, F.; Wodyński, A.; Yu, J. M. TURBOMOLE: Modular Program Suite for Ab Initio Quantum-Chemical and Condensed-Matter Simulations. *J. Chem. Phys.* **2020**, *152* (18), 184107. https://doi.org/10.1063/5.0004635.
(22) Field, M. J.; Bash, P. A.; Karplus, M. A Combined Quantum Mechanical and Molecular Mechanical Potential for Molecular Dynamics Simulations. *J. Comput. Chem.* **1990**, *11* (6), 700–733. https://doi.org/10.1002/jcc.540110605.
(23) Weigend, F. Accurate Coulomb-Fitting Basis Sets for H to Rn. *Phys. Chem. Chem. Phys.* **2006**, *8* (9), 1057–1065. https://doi.org/10.1039/B515623H.
(24) Henkelman, G.; Jónsson, H. A Dimer Method for Finding Saddle Points on High Dimensional Potential Surfaces Using Only First Derivatives. *J. Chem. Phys.* **1999**, *111* (15), 7010–7022. https://doi.org/10.1063/1.480097.
(25) Kästner, J.; Carr, J. M.; Keal, T. W.; Thiel, W.; Wander, A.; Sherwood, P. DL-FIND: An Open-Source Geometry Optimizer for Atomistic Simulations. *J. Phys. Chem. A* **2009**, *113* (43), 11856–11865. https://doi.org/10.1021/jp9028968.

(26) Neese, F. The ORCA Program System. *Wiley Interdiscip. Rev. Comput. Mol. Sci.* **2012**, *2* (1), 73–78. https://doi.org/10.1002/wcms.81.
(27) Neese, F.; Wennmohs, F.; Becker, U.; Riplinger, C. The ORCA Quantum Chemistry Program Package. *J. Chem. Phys.* **2020**, *152* (22), 224108. https://doi.org/10.1063/5.0004608.
(28) Römelt, M.; Ye, S.; Neese, F. Calibration of Modern Density Functional Theory Methods for the Prediction of 57 Fe Mössbauer Isomer Shifts: Meta-GGA and Double-Hybrid Functionals. *Inorg. Chem.* **2009**, *48* (3), 784–785. https://doi.org/10.1021/ic801535v.
(29) Genz, F.; Friedrich, F.; Lönarz, C.; Einsle, O.; Jung, M.; Müller, M.; Fessner, N. D. Identification and Characterization of Pyrimidine Nucleoside 2′-Hydroxylase. *ACS Catal.* **2025**, *15* (5), 3611–3618. https://doi.org/10.1021/acscatal.4c07764.

Coordinates of QM/MM Optimized Geometries

Off-SO-RC
QM(B1)/MM Energy = -4509.329820405043 a.u.
QM(B2)/MM Energy = -4512.576183000000 a.u.
QM(B3)/MM Energy = -4512.015685913992 a.u.

1 C	29.6430358	42.1660034	41.4856149
2 C	30.7171456	41.2662328	42.0251880
3 C	31.6555716	40.4984913	41.3702136
4 N	30.8767096	40.9532849	43.3587823
5 C	31.8508844	40.0329844	43.4750098
6 N	32.3496101	39.7300992	42.2819042
7 H	29.9270209	42.4960899	40.4744232
8 H	29.5297863	43.0769563	42.0936875
9 H	31.8477748	40.4095717	40.3039715
10 H	32.1652361	39.5951661	44.4205125
11 H	30.3106093	41.3252446	44.1317985
12 C	28.5217230	35.9440411	44.2758491
13 C	29.5443886	36.2699625	43.2325793
14 C	30.8376514	36.7225483	43.3516798
15 N	29.3306071	36.1637719	41.8672559
16 C	30.4456487	36.5681662	41.2249779
17 N	31.3808278	36.9178831	42.0978315
18 H	29.0161291	35.6502328	45.2135121
19 H	27.9070670	35.0870666	43.9724532
20 H	31.4150368	36.8959431	44.2561424
21 H	30.5483152	36.5970779	40.1416222
22 H	28.4595442	35.8844111	41.4040642
23 Fe	33.3009648	37.8447018	41.7122063
24 O	33.9581030	35.8569436	41.7289192
25 O	34.9692886	35.4890705	42.3793991
26 Cl	33.1063952	38.1420516	39.3912299
27 C	35.9180061	38.1226230	42.8245511
28 O	35.2082803	38.4549510	41.7987282
29 O	37.1246499	38.2576807	42.9634865
30 C	35.0564378	37.5381870	43.9695886
31 O	33.8309670	37.6168581	43.8208105
32 C	35.7096190	36.9813741	45.1815753
33 C	34.7359111	36.3982189	46.1910327
34 C	35.4089904	35.7147296	47.3935165
35 O	34.9167983	34.6428225	47.7787231
36 O	36.4169163	36.2997940	47.9115594
37 H	36.3506581	37.7632943	45.6257530
38 H	36.4392064	36.2346046	44.8177068
39 H	34.0939143	37.2119173	46.5678233
40 H	34.0673324	35.6756259	45.7042296
41 O	32.1410511	45.5510225	39.9065495
42 C	32.6030501	44.2387730	39.6655512
43 C	34.0868948	44.1737121	39.3204800
44 O	34.8446390	44.7323878	40.3858316
45 C	34.6431850	42.7314986	39.1191889
46 O	34.8824119	42.3780125	37.7649589
47 C	35.9546500	42.7328158	39.9362219
48 C	36.1571191	44.2125586	40.2916719
49 N	36.8066351	44.4875907	41.5514219
50 C	36.3041695	44.1645701	42.7869763
51 N	36.9492119	44.7322531	43.7811279
52 C	37.9468266	45.4699761	43.1681493
53 C	38.9783568	46.2966249	43.6673920
54 N	39.1705716	46.4747928	45.0052502
55 N	39.7992594	46.8808723	42.7867900
56 C	39.6024993	46.6862712	41.4749992
57 N	38.6599709	45.9469986	40.8856733
58 C	37.8715201	45.3455759	41.7750427
59 P	31.5822734	46.4872992	38.6157654
60 O	32.2995912	45.9343760	37.3587538
61 O	30.0537371	46.2948340	38.5682211
62 O	32.0101313	47.9228698	38.9562025
63 H	32.0456205	43.7520600	38.8435502
64 H	32.4352543	43.6482959	40.5849638
65 H	34.2643213	44.7664801	38.4068368
66 H	33.9396564	41.9964423	39.5541570
67 H	36.7884523	42.3204500	39.3534259
68 H	35.8561082	42.1278221	40.8491199
69 H	36.7486444	44.7385618	39.5200429
70 H	35.4350455	43.5171991	42.8943902
71 H	40.3026369	47.2026329	40.8080208
72 H	39.7232077	47.2905238	45.2782205
73 H	38.3607744	46.2930973	45.5923113
74 H	34.4021631	42.9892769	37.1447772
75 H	28.6623557	41.6928207	41.4363020
76 H	27.7900022	36.7263136	44.4775954

Off-SO-TS1
QM(B1)/MM Energy = -4509.314117314249 a.u.
QM(B2)/MM Energy = -4512.560919000000 a.u.
QM(B3)/MM Energy = -4511.998791556231 a.u.

1 C	29.6356064	42.1651997	41.4613982
2 C	30.7195508	41.2709733	41.9906350
3 C	31.6313485	40.4796826	41.3256323
4 N	30.9140264	40.9852559	43.3259424
5 C	31.8823467	40.0576793	43.4350924
6 N	32.3410275	39.7253322	42.2338144
7 H	29.9068097	42.4967514	40.4470672
8 H	29.5238016	43.0754013	42.0707300
9 H	31.7984715	40.3691047	40.2574048
10 H	32.2229333	39.6276726	44.3754163
11 H	30.3660494	41.3726593	44.1044412
12 C	28.5133999	35.9280961	44.2928185
13 C	29.5419837	36.2468635	43.2537749
14 C	30.8363993	36.6936354	43.3790499
15 N	29.3325099	36.1441146	41.8868998

16 C	30.4508277	36.5443170	41.2488369
17 N	31.3832363	36.8881796	42.1272945
18 H	29.0032305	35.6371083	45.2337007
19 H	27.8979309	35.0714561	43.9902333
20 H	31.4145209	36.8675182	44.2831574
21 H	30.5611836	36.5747373	40.1664207
22 H	28.4618078	35.8691801	41.4202266
23 Fe	33.2873356	37.8192476	41.8108481
24 O	34.2367206	36.0601879	41.9231537
25 O	35.1517949	36.0628605	42.9243837
26 Cl	33.1207276	38.0541111	39.4715469
27 C	35.9900797	38.2657435	42.7507005
28 O	35.2558496	38.6498369	41.8083063
29 O	37.1465611	38.4464290	43.0512754
30 C	35.0201205	37.2217101	43.7749788
31 O	33.8246536	37.7462429	43.7427064
32 C	35.7089658	36.9303774	45.0976197
33 C	34.7343854	36.3074622	46.0963539
34 C	35.3946665	35.6818314	47.3398560
35 O	34.8827916	34.6338751	47.7668215
36 O	36.4005373	36.2761269	47.8477077
37 H	36.1380827	37.8622359	45.4879171
38 H	36.5595495	36.2647943	44.8854885
39 H	34.0298560	37.0849238	46.4358155
40 H	34.1251914	35.5356248	45.6073855
41 O	32.1424436	45.5634306	39.9127230
42 C	32.5981246	44.2478105	39.6779356
43 C	34.0819642	44.1740787	39.3356497
44 O	34.8413287	44.7314839	40.4002464
45 C	34.6306221	42.7284707	39.1397019
46 O	34.8583318	42.3638705	37.7860415
47 C	35.9460395	42.7288871	39.9507376
48 C	36.1524949	44.2082116	40.3037237
49 N	36.8074113	44.4828374	41.5601006
50 C	36.3142968	44.1522108	42.7977446
51 N	36.9564469	44.7283081	43.7888559
52 C	37.9429285	45.4776305	43.1720622
53 C	38.9693061	46.3125959	43.6677907
54 N	39.1662043	46.4895658	45.0049507
55 N	39.7815029	46.9050847	42.7844752
56 C	39.5810583	46.7096104	41.4734539
57 N	38.6434801	45.9614850	40.8873011
58 C	37.8636112	45.3526208	41.7794849
59 P	31.5841854	46.4945066	38.6185853
60 O	32.3045822	45.9393629	37.3642745
61 O	30.0560094	46.2983291	38.5685449
62 O	32.0083244	47.9316634	38.9566430
63 H	32.0394167	43.7605251	38.8571714
64 H	32.4261177	43.6617771	40.5993374
65 H	34.2642253	44.7638075	38.4209126
66 H	33.9263091	42.0005808	39.5850485
67 H	36.7769862	42.3181509	39.3629585
68 H	35.8508380	42.1229958	40.8634003
69 H	36.7420908	44.7326028	39.5294788
70 H	35.4569401	43.4896984	42.9095386
71 H	40.2736120	47.2331151	40.8041241
72 H	39.7158032	47.3074237	45.2775686
73 H	38.3605420	46.3006082	45.5954284
74 H	34.3922983	42.9849125	37.1644624
75 H	28.6580401	41.6846483	41.4226474
76 H	27.7842913	36.7144711	44.4879654

Off-SO-IM1

QM(B1)/MM Energy = -4509.389339978116 a.u.

QM(B2)/MM Energy = -4512.639422000000 a.u.

QM(B3)/MM Energy = -4512.069135571418 a.u.

1 C	29.5684179	42.1472609	41.4804881
2 C	30.6395633	41.2420496	42.0156014
3 C	31.5529211	40.4570813	41.3454605
4 N	30.7930344	40.9048144	43.3441425
5 C	31.7411296	39.9505784	43.4378435
6 N	32.2208812	39.6513986	42.2393148
7 H	29.8507650	42.4777240	40.4687977
8 H	29.4519094	43.0580382	42.0883884
9 H	31.7477166	40.3768386	40.2787674
10 H	32.0489947	39.4859199	44.3728315
11 H	30.2381043	41.2786855	44.1231781
12 C	28.4499094	35.8918293	44.3133090
13 C	29.4804443	36.1615458	43.2628430
14 C	30.8002821	36.5349590	43.3714590
15 N	29.2548814	36.0615672	41.8990997
16 C	30.3937530	36.3912916	41.2522309
17 N	31.3534942	36.6835838	42.1169183
18 H	28.9395612	35.6114900	45.2575236
19 H	27.8179399	35.0390940	44.0341574
20 H	31.3855166	36.6929355	44.2746439
21 H	30.4890490	36.4228182	40.1675329
22 H	28.3699279	35.8246229	41.4399720
23 Fe	33.1415406	37.7686371	41.6852740
24 O	34.7423265	36.6357755	41.5168139
25 O	35.2420502	36.0585779	42.7276367
26 Cl	33.1685414	38.3419027	39.3342653
27 C	36.1638593	39.2740671	42.5567678
28 O	35.3003365	39.9971935	42.2741834
29 O	37.0624390	38.5848218	42.8317577
30 C	34.8209280	36.6682346	43.8139344
31 O	33.9620012	37.5564880	43.7760033
32 C	35.5248382	36.1959010	45.0527555
33 C	34.7302300	36.4508702	46.3308627
34 C	35.3925491	35.7169404	47.5064022
35 O	34.8948563	34.6332935	47.8508966
36 O	36.4390543	36.2509680	48.0005756
37 H	36.4986430	36.7146022	45.0990691
38 H	35.7563980	35.1265848	44.9278902
39 H	34.6912306	37.5341883	46.5254523

40 H	33.7043504	36.0774527	46.2075030
41 O	32.1327827	45.5128962	39.9083591
42 C	32.6117621	44.2034342	39.6672901
43 C	34.0805054	44.1750494	39.2574187
44 O	34.8748146	44.7755009	40.2836539
45 C	34.6805496	42.7535744	39.0097718
46 O	35.2552507	42.6265425	37.7182802
47 C	35.7987485	42.6612558	40.0544427
48 C	36.1326188	44.1329523	40.3020349
49 N	36.7561674	44.4497325	41.5683766
50 C	36.2234263	44.2018414	42.8086909
51 N	36.8719037	44.7945433	43.7869856
52 C	37.8999001	45.4744892	43.1583969
53 C	38.9516975	46.2887821	43.6368024
54 N	39.1323969	46.5147614	44.9691934
55 N	39.8041158	46.8077458	42.7449609
56 C	39.6184691	46.5606311	41.4396356
57 N	38.6621127	45.8244641	40.8696799
58 C	37.8406338	45.2879805	41.7712038
59 P	31.5900858	46.4528783	38.6143053
60 O	32.3093244	45.9022285	37.3557989
61 O	30.0599021	46.2679527	38.5569144
62 O	32.0199694	47.8881462	38.9575595
63 H	32.0307231	43.6983330	38.8730442
64 H	32.4842780	43.6224309	40.5988012
65 H	34.1824931	44.7749471	38.3404457
66 H	33.9308305	41.9558001	39.1697429
67 H	36.6478415	42.0717776	39.6852843
68 H	35.4265901	42.1906373	40.9748192
69 H	36.8073047	44.5308007	39.5222279
70 H	35.3358063	43.5832671	42.9359215
71 H	40.3433078	47.0246302	40.7605350
72 H	39.7090546	47.3205479	45.2206847
73 H	38.3090273	46.3864594	45.5513754
74 H	34.6862239	43.0880573	37.0530455
75 H	28.5912617	41.6667915	41.4316801
76 H	27.7319215	36.6911065	44.4969294

Off-SO-TS2
QM(B1)/MM Energy = -4509.370382279554 a.u.
QM(B2)/MM Energy = -4512.625296000000 a.u.
QM(B3)/MM Energy = -4512.065606145698 a.u.

1 C	29.6259477	42.1080151	41.5548002
2 C	30.6797613	41.1787168	42.0844816
3 C	31.5849795	40.4004953	41.3987601
4 N	30.8407765	40.8244379	43.4082181
5 C	31.7909080	39.8688878	43.4824630
6 N	32.2603830	39.5803668	42.2751903
7 H	29.9301257	42.4519858	40.5540146
8 H	29.5156189	43.0083198	42.1795006
9 H	31.7704205	40.3358692	40.3303818
10 H	32.1117438	39.3975952	44.4100267
11 H	30.2888117	41.1967059	44.1909888
12 C	28.4769889	35.8816580	44.2918256
13 C	29.4964811	36.1484605	43.2299682
14 C	30.8226388	36.4974718	43.3297617
15 N	29.2537245	36.0684898	41.8673892
16 C	30.3826840	36.3932377	41.2068799
17 N	31.3554577	36.6587471	42.0667021
18 H	28.9794953	35.6052169	45.2303729
19 H	27.8438716	35.0268319	44.0225697
20 H	31.4246553	36.6284142	44.2264888
21 H	30.4677992	36.4399575	40.1220942
22 H	28.3633301	35.8401759	41.4134164
23 Fe	33.1800717	37.6550961	41.6918473
24 O	34.4505866	36.5757329	41.2196670
25 O	35.2628938	35.8597648	42.7899209
26 Cl	33.0730360	38.4317076	39.3561704
27 C	36.1884820	39.2404700	42.5734176
28 O	35.3019763	39.9379074	42.2963183
29 O	37.1062089	38.5686357	42.8245049
30 C	34.8693096	36.5787670	43.7478233
31 O	34.0016367	37.5064917	43.6434357
32 C	35.5496933	36.2752134	45.0713710
33 C	34.7004519	36.4991617	46.3214975
34 C	35.3525989	35.7669044	47.5095337
35 O	34.8453458	34.6892498	47.8603253
36 O	36.4045006	36.2911174	48.0030372
37 H	36.4620734	36.8951152	45.1161969
38 H	35.8895202	35.2308929	45.0109473
39 H	34.6164079	37.5783103	46.5272366
40 H	33.6920332	36.0900841	46.1681744
41 O	32.1306855	45.5213951	39.9077291
42 C	32.6057866	44.2108695	39.6673000
43 C	34.0763174	44.1754464	39.2651545
44 O	34.8676464	44.7688410	40.2968595
45 C	34.6671078	42.7493538	39.0212197
46 O	35.2158515	42.6014862	37.7205377
47 C	35.8023340	42.6607921	40.0499159
48 C	36.1293196	44.1333489	40.3046529
49 N	36.7572714	44.4460213	41.5694477
50 C	36.2295451	44.1898409	42.8104047
51 N	36.8768200	44.7830197	43.7890021
52 C	37.8986364	45.4720369	43.1601961
53 C	38.9468134	46.2903556	43.6396468
54 N	39.1301647	46.5106297	44.9725125
55 N	39.7934492	46.8185612	42.7476892
56 C	39.6057799	46.5760196	41.4419502
57 N	38.6527526	45.8359734	40.8710863
58 C	37.8369225	45.2908101	41.7725973
59 P	31.5853462	46.4603825	38.6138226
60 O	32.3040695	45.9091084	37.3554517
61 O	30.0554446	46.2745097	38.5573130
62 O	32.0145781	47.8957852	38.9572425
63 H	32.0265135	43.7081630	38.8702455

64 H	32.4735704	43.6294164	40.5978781
65 H	34.1886540	44.7747722	38.3484960
66 H	33.9157648	41.9579728	39.2037677
67 H	36.6512331	42.0818674	39.6637053
68 H	35.4489831	42.1794947	40.9725856
69 H	36.7976567	44.5398864	39.5236524
70 H	35.3467788	43.5643267	42.9379137
71 H	40.3259162	47.0475640	40.7630537
72 H	39.7035139	47.3178702	45.2268943
73 H	38.3098938	46.3729673	45.5570285
74 H	34.6512689	43.0780672	37.0613489
75 H	28.6412011	41.6472770	41.4769433
76 H	27.7587977	36.6790164	44.4828442

Off-SO-IM2
QM(B1)/MM Energy = -4509.376424433942 a.u.
QM(B2)/MM Energy = -4512.626022000000 a.u.
QM(B3)/MM Energy = -4512.066197925940 a.u.

1 C	29.7112211	41.9872852	41.5762096
2 C	30.7314774	41.0132180	42.0956570
3 C	31.5986524	40.1901765	41.4089867
4 N	30.8940959	40.6664221	43.4205028
5 C	31.8089230	39.6717417	43.4880699
6 N	32.2514666	39.3485063	42.2812353
7 H	30.0301042	42.3345258	40.5806900
8 H	29.6304236	42.8824850	42.2128446
9 H	31.7779338	40.1274495	40.3393862
10 H	32.1253474	39.2095875	44.4215115
11 H	30.3667026	41.0741631	44.2026995
12 C	28.3159420	35.8563954	44.3832401
13 C	29.3281559	36.0974302	43.3118444
14 C	30.6437336	36.4888832	43.3844393
15 N	29.0720080	35.9694171	41.9588502
16 C	30.1832249	36.2971905	41.2732626
17 N	31.1543185	36.6198099	42.1119415
18 H	28.8148842	35.5831142	45.3240268
19 H	27.6641317	35.0129452	44.1261811
20 H	31.2495944	36.6594821	44.2709712
21 H	30.2525153	36.2906980	40.1867423
22 H	28.1807853	35.7169587	41.5211119
23 Fe	33.0955362	37.2636706	41.6124475
24 O	33.6797610	35.6952487	41.0961208
25 O	34.8513370	35.1551678	42.7710284
26 Cl	33.2886469	38.2626691	39.4463475
27 C	36.1398618	39.2406839	42.7370360
28 O	35.2980862	40.0300494	42.6058496
29 O	37.0319726	38.4964168	42.8299411
30 C	34.8724269	36.1451816	43.5143211
31 O	34.2654210	37.2665607	43.2467233
32 C	35.6511009	36.0352517	44.8199458
33 C	34.8455571	36.4368992	46.0697646
34 C	35.4563251	35.7805394	47.3291006
35 O	34.9337369	34.7217107	47.7157566
36 O	36.4768840	36.3354515	47.8503786
37 H	36.5589530	36.6554352	44.7383147
38 H	35.9759112	34.9887634	44.8999151
39 H	34.8265265	37.5337570	46.1758858
40 H	33.8124899	36.0709510	45.9747082
41 O	32.1385416	45.5218073	39.9117722
42 C	32.6121452	44.2102203	39.6806740
43 C	34.0815616	44.1748733	39.2733298
44 O	34.8724816	44.7847519	40.2952092
45 C	34.6782526	42.7495194	39.0445972
46 O	35.2321310	42.5919215	37.7472928
47 C	35.8087569	42.6733617	40.0793229
48 C	36.1333512	44.1497900	40.3177432
49 N	36.7529081	44.4787037	41.5822583
50 C	36.2071979	44.2572822	42.8221793
51 N	36.8574256	44.8536865	43.7963380
52 C	37.9005123	45.5083514	43.1657968
53 C	38.9623951	46.3118151	43.6401781
54 N	39.1386541	46.5511566	44.9705162
55 N	39.8284922	46.8051347	42.7470261
56 C	39.6468676	46.5446248	41.4439209
57 N	38.6826700	45.8157984	40.8774897
58 C	37.8480088	45.3037382	41.7808707
59 P	31.6054357	46.4570011	38.6105915
60 O	32.3290558	45.8985922	37.3581694
61 O	30.0751688	46.2755169	38.5458135
62 O	32.0379734	47.8920902	38.9504808
63 H	32.0297442	43.6994984	38.8907696
64 H	32.4844447	43.6373669	40.6174143
65 H	34.1865897	44.7652137	38.3504989
66 H	33.9264846	41.9591758	39.2316888
67 H	36.6612638	42.0930521	39.7035162
68 H	35.4527857	42.2030260	41.0072455
69 H	36.8069401	44.5455525	39.5356708
70 H	35.3074856	43.6568347	42.9503291
71 H	40.3827048	46.9888376	40.7633951
72 H	39.7265119	47.3500019	45.2175417
73 H	38.3097740	46.4434946	45.5487424
74 H	34.6743077	43.0698014	37.0828272
75 H	28.7092768	41.5672939	41.4879937
76 H	27.6204727	36.6783009	44.5531249

Off-SO-PD
QM(B1)/MM Energy = -4509.376424433942 a.u.
QM(B2)/MM Energy = -4512.646526000000 a.u.
QM(B3)/MM Energy = -4512.086573303258 a.u.

1 C	29.7688994	41.9073921	41.6483715
2 C	30.7571640	40.8968002	42.1603664
3 C	31.5835243	40.0519166	41.4551284
4 N	30.9232619	40.5320492	43.4807871
5 C	31.7997968	39.5038529	43.5312241

6 N 32.2132506 39.1768968 42.3127215
7 H 30.1117327 42.2641678 40.6642811
8 H 29.7007455 42.7913132 42.3016600
9 H 31.7563109 40.0004156 40.3851624
10 H 32.1151244 39.0215667 44.4541298
11 H 30.4131126 40.9515906 44.2688246
12 C 28.3005333 35.8213589 44.4121649
13 C 29.3218027 36.0487496 43.3470878
14 C 30.6395063 36.4314764 43.4306711
15 N 29.0762801 35.9129080 41.9927958
16 C 30.1961828 36.2249208 41.3140392
17 N 31.1583870 36.5499966 42.1613345
18 H 28.7908858 35.5435174 45.3559178
19 H 27.6412839 34.9852048 44.1510565
20 H 31.2424910 36.5941718 44.3203996
21 H 30.2794543 36.2068318 40.2289072
22 H 28.1862781 35.6675451 41.5481258
23 Fe 33.0388559 37.2530496 41.6568593
24 O 33.3921540 35.8243279 40.9994251
25 O 35.1973463 35.1957602 42.8310323
26 Cl 33.3459563 38.2960969 39.5301520
27 C 36.0163716 39.2976189 42.5983297
28 O 35.1611124 40.0824904 42.5375215
29 O 36.9246286 38.5697450 42.6361727
30 C 35.0276304 36.1858370 43.5039304
31 O 34.2475479 37.2199067 43.1309334
32 C 35.7097180 36.3372877 44.8618088
33 C 34.7448914 36.4277324 46.0630921
34 C 35.3652652 35.7894515 47.3272006
35 O 34.8333369 34.7497264 47.7519888
36 O 36.3979428 36.3460575 47.8259943
37 H 36.3823565 37.2095383 44.8498256
38 H 36.3411589 35.4427032 44.9573511
39 H 34.4912945 37.4824752 46.2664230
40 H 33.8135913 35.8848324 45.8444069
41 O 32.1557401 45.4955853 39.9035886
42 C 32.6371236 44.1877326 39.6778218
43 C 34.1144959 44.1532659 39.2998389
44 O 34.8773862 44.7789375 40.3302102
45 C 34.7162729 42.7242009 39.1140422
46 O 35.1848837 42.4730864 37.7955449
47 C 35.9020225 42.7112385 40.0928499
48 C 36.1650072 44.2007873 40.3310007
49 N 36.7844930 44.5455517 41.5900518
50 C 36.2451580 44.3207172 42.8320498
51 N 36.9005959 44.9148394 43.8039042
52 C 37.9416669 45.5686232 43.1692414
53 C 39.0086910 46.3672264 43.6393054
54 N 39.1914073 46.6037942 44.9690941
55 N 39.8725272 46.8576437 42.7427037
56 C 39.6841254 46.6006538 41.4400429
57 N 38.7140499 45.8769393 40.8770927
58 C 37.8825352 45.3668401 41.7842055
59 P 31.6394311 46.4340035 38.5972418
60 O 32.3685065 45.8678775 37.3524183
61 O 30.1084756 46.2639554 38.5243649
62 O 32.0803977 47.8664906 38.9378675
63 H 32.0713047 43.6744905 38.8775356
64 H 32.4970450 43.6136202 40.6123132
65 H 34.2428724 44.7292059 38.3701016
66 H 33.9816870 41.9455224 39.3951802
67 H 36.7662800 42.1794175 39.6740120
68 H 35.6194308 42.2212468 41.0355252
69 H 36.8105481 44.6309924 39.5431311
70 H 35.3452061 43.7211428 42.9619356
71 H 40.4188458 47.0426822 40.7570358
72 H 39.7830149 47.4002388 45.2142011
73 H 38.3617403 46.5062569 45.5476692
74 H 34.6472317 42.9838973 37.1369855
75 H 28.7593747 41.5126808 41.5337852
76 H 27.6140368 36.6518753 44.5765799

In-SO-RC
QM(B1)/MM Energy = -4510.639684194954 a.u.
QM(B2)/MM Energy = -4513.882590000000 a.u.
QM(B3)/MM Energy = -4513.320492683790 a.u.

1 C 41.5267912 25.9982206 33.5310905
2 C 40.5212368 27.1127146 33.4877526
3 C 40.1663436 28.0345423 34.4466769
4 N 39.7009407 27.3950311 32.4126906
5 C 38.9129268 28.4409148 32.7295104
6 N 39.1646320 28.8541604 33.9653566
7 H 42.2690449 26.2188689 34.3129712
8 H 42.1016404 25.9125597 32.5967116
9 H 40.5514039 28.1543155 35.4550207
10 H 38.1684082 28.8712632 32.0636076
11 H 39.6689403 26.8712164 31.5300150
12 C 34.6355527 25.4127941 33.7029376
13 C 35.4312435 26.4224093 34.4642284
14 C 35.8717140 27.6820452 34.1337994
15 N 35.8999082 26.2089829 35.7462065
16 C 36.6039420 27.2899416 36.1365015
17 N 36.6033794 28.2042159 35.1778907
18 H 33.9688631 25.9171131 32.9889619
19 H 33.9870106 24.8447216 34.3854734
20 H 35.7050109 28.2406697 33.2167564
21 H 37.1093829 27.3850074 37.0967391
22 H 35.7317389 25.3670937 36.3044077
23 Fe 37.8118035 29.9985039 35.2218113
24 O 38.6421467 31.8143377 34.7483714
25 O 37.9536240 32.8535943 34.5340457
26 Cl 38.9873632 29.5912387 37.1906076
27 C 35.4127392 31.5694738 35.2042443
28 O 36.2441083 30.9858810 35.9913877
29 O 34.3439744 32.0846500 35.5034454

30 C	35.8394130	31.5227354	33.7087204
31 O	36.6524805	30.6481655	33.3922937
32 C	35.1358741	32.4314346	32.7455582
33 C	35.8482239	32.7273290	31.4281917
34 C	35.0502651	33.6259964	30.4578932
35 O	33.8238738	33.8111717	30.6855344
36 O	35.6807219	34.1138645	29.4879742
37 H	34.8800254	33.3550971	33.2874563
38 H	34.1475802	31.9705850	32.5514064
39 H	36.8217405	33.2096423	31.6138257
40 H	36.0810158	31.7916756	30.8895800
41 O	44.8998646	28.8914424	36.2798452
42 C	43.9847526	29.2504709	35.2701469
43 C	43.7935693	30.7784611	35.1879034
44 O	44.2262637	31.2719486	33.8995234
45 C	42.3556494	31.3148064	35.3605907
46 O	42.3577829	32.5765291	36.0243213
47 C	41.8970111	31.4862695	33.9089844
48 C	43.1937571	31.9213466	33.2202441
49 N	43.2853890	31.6202801	31.7805831
50 C	43.7420022	32.5199897	30.8594307
51 N	43.7825900	32.0607331	29.6236726
52 C	43.3324946	30.7565981	29.7399308
53 C	43.0428716	29.7576064	28.7748939
54 N	43.1232721	29.9694229	27.4469330
55 N	42.5991907	28.5722142	29.2246631
56 C	42.4130087	28.3912552	30.5414688
57 N	42.5812245	29.2730387	31.5223526
58 C	43.0327813	30.4506039	31.0786322
59 P	46.5402986	29.0034332	35.9480848
60 O	46.7865128	28.1693591	34.6822408
61 O	46.8901120	30.5105906	35.7388200
62 O	47.1329580	28.4510564	37.2550317
63 H	43.0284443	28.7524169	35.5075338
64 H	44.3149326	28.8789049	34.2852145
65 H	44.4397301	31.2265989	35.9534895
66 H	41.7095954	30.6116907	35.9078403
67 H	41.0709505	32.2027448	33.8092777
68 H	41.5755289	30.5146804	33.5089378
69 H	43.3206415	33.0199208	33.2724880
70 H	44.0517905	33.5106307	31.1764909
71 H	42.0690623	27.3936162	30.8311764
72 H	42.9551547	29.2146996	26.7801740
73 H	43.5984399	30.7831657	27.0520189
74 H	42.1102975	32.4498433	36.9731695
75 H	41.0946562	25.0139816	33.7116457
76 H	35.2242135	24.6558051	33.1847548

In-SO-TS1
QM(B1)/MM Energy = -4510.639684194954 a.u.
QM(B2)/MM Energy = -4513.882590000000 a.u.
QM(B3)/MM Energy = -4513.320492683790 a.u.

1 C	41.5101029	26.0112548	33.5195655
2 C	40.4958318	27.1174895	33.4762099
3 C	40.1383423	28.0376620	34.4358479
4 N	39.6721299	27.3951123	32.4023678
5 C	38.8789271	28.4362076	32.7186516
6 N	39.1330098	28.8510072	33.9534042
7 H	42.2517107	26.2392736	34.2999057
8 H	42.0826412	25.9286558	32.5837180
9 H	40.5230038	28.1632145	35.4434138
10 H	38.1259810	28.8649138	32.0602994
11 H	39.6420371	26.8712774	31.5187319
12 C	34.6416461	25.4304051	33.7236215
13 C	35.4365492	26.4355025	34.4935499
14 C	35.8446819	27.7123307	34.1879749
15 N	35.9381739	26.1965783	35.7598265
16 C	36.6336966	27.2753106	36.1655250
17 N	36.5961932	28.2129148	35.2308217
18 H	33.9770235	25.9404132	33.0120580
19 H	33.9891336	24.8598660	34.4007114
20 H	35.6403008	28.3108952	33.3038964
21 H	37.1518763	27.3541210	37.1208758
22 H	35.7821982	25.3436151	36.3039291
23 Fe	37.7424386	29.9942865	35.1411581
24 O	38.3305413	31.9748846	34.7915161
25 O	37.3295730	32.6733775	34.2465818
26 Cl	39.0099983	29.7177703	37.0960767
27 C	35.4259703	31.6451872	35.2907320
28 O	36.1556455	30.9671846	36.0893202
29 O	34.3161157	32.1154686	35.4376725
30 C	36.2353601	31.8085623	33.8266394
31 O	36.6822264	30.5906413	33.5403146
32 C	35.3796271	32.5689984	32.8165041
33 C	36.0214333	32.8212886	31.4508599
34 C	35.1614339	33.6563153	30.4746379
35 O	33.9312813	33.7961564	30.7123653
36 O	35.7575935	34.1453570	29.4837472
37 H	35.0856253	33.5199424	33.2880615
38 H	34.4478461	31.9994778	32.7006139
39 H	36.9927349	33.3311113	31.5558823
40 H	36.2405673	31.8628411	30.9471822
41 O	44.8986128	28.9030775	36.2694077
42 C	43.9941821	29.2648856	35.2509035
43 C	43.7943111	30.7925889	35.1788110
44 O	44.2090314	31.2981679	33.8894943
45 C	42.3547836	31.3155210	35.3670324
46 O	42.3548602	32.5827076	36.0195676
47 C	41.8758838	31.4721164	33.9193570
48 C	43.1594982	31.9304774	33.2186010
49 N	43.2526060	31.6309260	31.7790480
50 C	43.7251634	32.5267203	30.8617331
51 N	43.7759344	32.0660580	29.6269726
52 C	43.3168208	30.7642781	29.7402707
53 C	43.0379638	29.7637394	28.7742825

54 N	43.1286386	29.9735486	27.4468052
55 N	42.5900705	28.5787944	29.2213458
56 C	42.3908487	28.4002705	30.5363692
57 N	42.5479717	29.2838756	31.5171942
58 C	43.0031848	30.4613679	31.0755774
59 P	46.5407967	29.0104268	35.9462303
60 O	46.7901938	28.1764916	34.6809605
61 O	46.8931393	30.5174354	35.7379298
62 O	47.1292229	28.4568432	37.2541670
63 H	43.0381234	28.7581612	35.4707014
64 H	44.3401208	28.9044459	34.2675933
65 H	44.4452698	31.2407446	35.9402907
66 H	41.7223444	30.6112037	35.9278108
67 H	41.0336193	32.1722803	33.8267221
68 H	41.5711668	30.4927425	33.5250357
69 H	43.2664330	33.0310776	33.2720079
70 H	44.0395673	33.5155883	31.1808727
71 H	42.0445444	27.4025474	30.8235055
72 H	42.9626706	29.2190725	26.7791770
73 H	43.6047448	30.7877329	27.0535162
74 H	42.1064899	32.4647559	36.9696359
75 H	41.0850358	25.0244807	33.7030377
76 H	35.2232530	24.6718406	33.1998082

In-SO-IM1
QM(B1)/MM Energy = -4510.707748673438 a.u.
QM(B2)/MM Energy = -4513.956885000000 a.u.
QM(B3)/MM Energy = -4513.382073213980 a.u.

1 C	41.5626195	25.9426459	33.5560113
2 C	40.5656949	27.0630977	33.4902872
3 C	40.2142358	28.0110934	34.4262287
4 N	39.7380580	27.3135414	32.4136844
5 C	38.9464385	28.3639675	32.7118391
6 N	39.2077520	28.8137569	33.9299884
7 H	42.2929951	26.1595843	34.3499013
8 H	42.1534143	25.8458226	32.6322593
9 H	40.5896469	28.1517816	35.4366834
10 H	38.1926762	28.7703017	32.0407586
11 H	39.6953249	26.7705796	31.5445124
12 C	34.6296383	25.4886794	33.7363070
13 C	35.4055238	26.4985151	34.5205342
14 C	35.7901943	27.7879710	34.2322151
15 N	35.8840385	26.2647176	35.7975434
16 C	36.5407135	27.3662520	36.2207519
17 N	36.4994884	28.3123373	35.2930540
18 H	33.9755628	25.9986828	33.0141727
19 H	33.9650509	24.9208637	34.4046742
20 H	35.6074495	28.3728265	33.3336691
21 H	37.0622806	27.4509376	37.1740077
22 H	35.7484917	25.4013545	36.3318359
23 Fe	37.8848143	29.9706669	35.1582427
24 O	37.5745389	31.9025936	35.4357848
25 O	36.8546008	32.6435134	34.4380425
26 Cl	39.0114706	29.5516653	37.2385004
27 C	35.0224553	31.6320660	36.6735235
28 O	35.4991547	31.5451638	37.7301441
29 O	34.4637813	31.7278503	35.6568076
30 C	36.4489110	31.9197428	33.4142993
31 O	36.7608319	30.7355225	33.2750606
32 C	35.5440035	32.7107096	32.5004499
33 C	36.1115928	32.9771850	31.1049289
34 C	35.1780323	33.8232925	30.2176305
35 O	33.9576933	33.8903324	30.5245720
36 O	35.7094045	34.3872450	29.2300097
37 H	35.2997368	33.6614564	32.9946967
38 H	34.6020888	32.1470895	32.4055731
39 H	37.0820042	33.4962251	31.1605768
40 H	36.3021702	32.0268282	30.5747587
41 O	44.8999910	28.9156265	36.2723374
42 C	43.9923340	29.2711596	35.2535001
43 C	43.7737758	30.7965988	35.1884078
44 O	44.1963222	31.3158556	33.9053616
45 C	42.3251494	31.3014782	35.3644965
46 O	42.3012381	32.5679143	36.0196633
47 C	41.8602208	31.4579310	33.9127301
48 C	43.1453780	31.9290472	33.2218235
49 N	43.2491049	31.6251243	31.7824546
50 C	43.7107648	32.5234477	30.8623679
51 N	43.7613442	32.0605938	29.6278139
52 C	43.3144078	30.7547574	29.7442798
53 C	43.0307728	29.7528765	28.7805747
54 N	43.1157210	29.9615959	27.4519769
55 N	42.5886056	28.5672942	29.2312806
56 C	42.3986184	28.3884389	30.5483401
57 N	42.5600042	29.2740880	31.5266800
58 C	43.0077678	30.4518901	31.0816173
59 P	46.5411408	29.0266507	35.9508175
60 O	46.7938782	28.1935787	34.6849063
61 O	46.8917920	30.5339860	35.7419062
62 O	47.1316412	28.4737210	37.2585292
63 H	43.0428110	28.7517293	35.4676630
64 H	44.3448358	28.9191871	34.2692976
65 H	44.4121971	31.2490439	35.9580408
66 H	41.6932604	30.5894068	35.9171459
67 H	41.0193872	32.1592328	33.8135529
68 H	41.5608534	30.4789732	33.5136174
69 H	43.2393123	33.0312599	33.2688584
70 H	44.0189584	33.5155823	31.1774774
71 H	42.0559109	27.3914756	30.8408283
72 H	42.9503780	29.2054460	26.7866708
73 H	43.5856504	30.7778478	27.0563222
74 H	42.0648323	32.4375262	36.9716016
75 H	41.1136582	24.9653316	33.7330921
76 H	35.2126220	24.7249861	33.2215549

In-SO-TS2
QM(B1)/MM Energy = -4510.692712533562 a.u.
QM(B2)/MM Energy = -4513.944586000000 a.u.
QM(B3)/MM Energy = -4513.384308456381 a.u.

1 C	41.5498446	25.9632756	33.5456553
2 C	40.5580519	27.0891449	33.4943031
3 C	40.2193538	28.0241321	34.4456785
4 N	39.7265785	27.3612024	32.4240691
5 C	38.9465591	28.4140221	32.7376358
6 N	39.2170761	28.8427996	33.9646305
7 H	42.2887751	26.1754684	34.3329042
8 H	42.1307782	25.8670609	32.6157600
9 H	40.5976811	28.1451600	35.4566532
10 H	38.1922018	28.8398988	32.0794744
11 H	39.6781257	26.8272678	31.5487751
12 C	34.6116892	25.5068895	33.7279429
13 C	35.3726603	26.5228264	34.5191466
14 C	35.7324208	27.8222441	34.2455318
15 N	35.8587745	26.2820570	35.7932367
16 C	36.4989690	27.3847894	36.2293806
17 N	36.4382866	28.3431123	35.3131276
18 H	33.9594606	26.0123394	33.0010167
19 H	33.9455816	24.9349996	34.3913088
20 H	35.5397480	28.4199627	33.3579388
21 H	37.0281950	27.4650599	37.1783063
22 H	35.7374570	25.4114853	36.3197176
23 Fe	37.8378905	29.9699405	35.1565792
24 O	37.6213436	31.6546249	35.6611311
25 O	36.7919265	32.6567490	34.4096861
26 Cl	38.9964399	29.3929612	37.2133169
27 C	34.9092427	31.6696619	36.7465074
28 O	35.3638096	31.6199215	37.8155305
29 O	34.3806255	31.7260976	35.7121726
30 C	36.4033628	31.8844119	33.4777332
31 O	36.7325039	30.6739318	33.3658652
32 C	35.4504123	32.5538483	32.4943741
33 C	36.0972084	32.9497665	31.1657463
34 C	35.1832209	33.7908618	30.2533041
35 O	33.9608352	33.8822973	30.5456487
36 O	35.7332584	34.3347240	29.2638060
37 H	35.0412657	33.4511489	32.9785545
38 H	34.6111715	31.8648281	32.3115889
39 H	37.0185613	33.5329296	31.3320481
40 H	36.4053637	32.0553289	30.5955922
41 O	44.8979955	28.9103440	36.2789828
42 C	43.9843217	29.2652084	35.2658126
43 C	43.7719423	30.7912732	35.1935375
44 O	44.1978657	31.3016690	33.9084610
45 C	42.3254870	31.3046022	35.3666555
46 O	42.3074687	32.5713089	36.0205173
47 C	41.8636613	31.4629101	33.9141494
48 C	43.1527375	31.9258142	33.2255051
49 N	43.2549982	31.6242006	31.7860509
50 C	43.7188930	32.5227085	30.8669908
51 N	43.7688491	32.0611621	29.6321402
52 C	43.3189077	30.7563658	29.7469649
53 C	43.0350612	29.7556930	28.7820103
54 N	43.1220723	29.9645834	27.4539050
55 N	42.5908647	28.5702524	29.2314213
56 C	42.3985657	28.3908217	30.5477352
57 N	42.5595873	29.2751897	31.5273529
58 C	43.0107299	30.4526394	31.0838709
59 P	46.5381961	29.0237567	35.9513755
60 O	46.7883896	28.1904466	34.6852165
61 O	46.8855947	30.5315685	35.7412748
62 O	47.1325194	28.4718330	37.2577035
63 H	43.0340079	28.7520168	35.4917853
64 H	44.3266156	28.9060845	34.2804854
65 H	44.4112366	31.2444063	35.9620661
66 H	41.6900928	30.5952627	35.9190029
67 H	41.0268056	32.1685449	33.8118441
68 H	41.5613581	30.4851671	33.5145377
69 H	43.2550039	33.0271021	33.2757766
70 H	44.0284494	33.5138636	31.1835741
71 H	42.0547020	27.3935531	30.8382480
72 H	42.9546856	29.2094434	26.7878380
73 H	43.5920389	30.7809783	27.0585534
74 H	42.0695561	32.4444863	36.9725622
75 H	41.1008686	24.9864814	33.7255461
76 H	35.2017692	24.7454241	33.2179987

In-SO-IM2
QM(B1)/MM Energy = -4510.701541477710 a.u.
QM(B2)/MM Energy = -4513.953701000000 a.u.
QM(B3)/MM Energy = -4513.390958619338 a.u.

1 C	41.5828332	25.9085087	33.5769158
2 C	40.5985871	27.0400339	33.5288237
3 C	40.2532074	27.9662026	34.4855367
4 N	39.7682019	27.3150931	32.4585887
5 C	38.9826178	28.3601013	32.7741933
6 N	39.2465743	28.7815075	34.0067879
7 H	42.3182821	26.1085108	34.3703509
8 H	42.1675485	25.8151749	32.6492220
9 H	40.6327734	28.0834295	35.4958175
10 H	38.2240875	28.7877389	32.1233222
11 H	39.7209498	26.7885089	31.5786997
12 C	34.5877131	25.4714766	33.7249095
13 C	35.3423473	26.4852681	34.5235896
14 C	35.7262371	27.7753580	34.2449516
15 N	35.8053311	26.2461786	35.8065134
16 C	36.4565138	27.3388089	36.2463033
17 N	36.4254056	28.2903656	35.3206596
18 H	33.9374893	25.9771796	32.9966441
19 H	33.9208407	24.8949003	34.3830643

20 H	35.5664352	28.3708588	33.3503329
21 H	36.9707384	27.4172782	37.2030885
22 H	35.6651966	25.3797055	36.3350022
23 Fe	37.8162967	29.9025290	35.1525494
24 O	37.5286431	31.5426721	35.7001127
25 O	36.9434868	32.5570799	33.9431270
26 Cl	38.9470669	29.2907706	37.1953778
27 C	34.8947774	31.6913185	36.4891729
28 O	35.2515912	31.6250216	37.5946862
29 O	34.4385492	31.7659133	35.4218506
30 C	36.6300164	31.6450130	33.1548449
31 O	36.9122722	30.4028052	33.3273037
32 C	35.8187655	32.0304670	31.9183472
33 C	36.2358533	33.3669995	31.3039338
34 C	35.2487001	33.9523268	30.2844486
35 O	34.0148295	33.8921958	30.5429762
36 O	35.7450940	34.5060616	29.2703141
37 H	34.7679056	32.1142227	32.2468129
38 H	35.8665968	31.2065246	31.1894900
39 H	36.3269715	34.1046519	32.1198960
40 H	37.2257764	33.2948420	30.8280053
41 O	44.8902294	28.9060263	36.2861339
42 C	43.9697053	29.2605212	35.2793500
43 C	43.7599672	30.7869082	35.2024884
44 O	44.1895330	31.2921308	33.9173522
45 C	42.3137924	31.3041464	35.3714437
46 O	42.2960516	32.5693411	36.0276429
47 C	41.8559709	31.4665660	33.9181853
48 C	43.1491024	31.9244721	33.2336389
49 N	43.2532756	31.6259829	31.7946119
50 C	43.7279193	32.5226758	30.8786453
51 N	43.7795452	32.0629269	29.6436267
52 C	43.3188056	30.7616384	29.7543810
53 C	43.0303351	29.7643536	28.7870668
54 N	43.1187715	29.9750931	27.4594361
55 N	42.5791477	28.5804588	29.2338365
56 C	42.3823863	28.3998786	30.5491171
57 N	42.5438592	29.2822772	31.5304360
58 C	43.0034999	30.4575116	31.0898693
59 P	46.5292807	29.0218526	35.9520872
60 O	46.7776813	28.1888701	34.6856291
61 O	46.8729465	30.5302860	35.7417494
62 O	47.1278185	28.4703491	37.2568176
63 H	43.0198636	28.7512342	35.5157889
64 H	44.3020770	28.8964843	34.2923280
65 H	44.3977843	31.2409002	35.9718940
66 H	41.6763109	30.5938611	35.9203257
67 H	41.0227079	32.1762672	33.8138397
68 H	41.5507341	30.4907610	33.5159427
69 H	43.2570496	33.0249771	33.2878744
70 H	44.0431044	33.5108479	31.1986590
71 H	42.0337985	27.4035520	30.8372003
72 H	42.9463743	29.2218097	26.7922122
73 H	43.5943545	30.7886678	27.0650274
74 H	42.0609017	32.4420212	36.9800153
75 H	41.1190283	24.9369184	33.7471055
76 H	35.1877959	24.7162473	33.2173699

In-SO-PD
QM(B1)/MM Energy = -4510.723857784473 a.u.
QM(B2)/MM Energy = -4513.978871000000 a.u.
QM(B3)/MM Energy = -4513.419504134100 a.u.

1 C	41.5504842	25.9500094	33.5722502
2 C	40.5448141	27.0647845	33.5557548
3 C	40.1798567	27.9502007	34.5444450
4 N	39.7154595	27.3658412	32.4926877
5 C	38.9116813	28.3875148	32.8457045
6 N	39.1597017	28.7634579	34.0949580
7 H	42.2993177	26.1555518	34.3520781
8 H	42.1168506	25.8737493	32.6319748
9 H	40.5787553	28.0591895	35.5475049
10 H	38.1638425	28.8391049	32.1996921
11 H	39.6870920	26.8692641	31.5942506
12 C	34.6381580	25.4710338	33.7427919
13 C	35.4434537	26.4534516	34.5318173
14 C	35.8874597	27.7228309	34.2411480
15 N	35.9146578	26.1969237	35.8058316
16 C	36.6255472	27.2614081	36.2316118
17 N	36.6231889	28.2015202	35.3019700
18 H	33.9892496	25.9988758	33.0290085
19 H	33.9687164	24.9075082	34.4088007
20 H	35.7270122	28.3224227	33.3486973
21 H	37.1351941	27.3215205	37.1924287
22 H	35.7400787	25.3395847	36.3383084
23 Fe	37.8011133	29.9544870	35.2337019
24 O	36.7809709	30.8442628	36.1191404
25 O	37.6628166	32.8396796	33.9526465
26 Cl	39.2991077	29.7773854	37.0169118
27 C	34.3690910	32.0262454	36.3001989
28 O	34.3578805	31.8722275	37.4526778
29 O	34.3050241	32.2092639	35.1525612
30 C	37.0990482	31.9712056	33.3121078
31 O	37.2452370	30.6766496	33.5495008
32 C	36.1493591	32.2758746	32.1492167
33 C	36.4066626	33.6272906	31.4837834
34 C	35.3939433	34.0351745	30.3995226
35 O	34.1612593	33.8884564	30.6303895
36 O	35.8688658	34.5400944	29.3484966
37 H	35.1251083	32.2624614	32.5607369
38 H	36.1976927	31.4491102	31.4212626
39 H	36.3899610	34.4053356	32.2670612
40 H	37.4129071	33.6576540	31.0394469
41 O	44.8924457	28.9805628	36.1910313
42 C	44.0646608	29.3816414	35.1214201
43 C	43.8328400	30.9063891	35.1261706

44	O	44.2029572	31.4901236	33.8555469
45	C	42.3830646	31.3690413	35.3603961
46	O	42.3446182	32.6351426	36.0157539
47	C	41.8585027	31.5039291	33.9254418
48	C	43.0986111	32.0254835	33.1879466
49	N	43.1856262	31.6931891	31.7530539
50	C	43.6450635	32.5768881	30.8184680
51	N	43.7149387	32.0884871	29.5951171
52	C	43.2850782	30.7799797	29.7353861
53	C	43.0287677	29.7543532	28.7895606
54	N	43.1302256	29.9359814	27.4592203
55	N	42.6010007	28.5709394	29.2608452
56	C	42.3993050	28.4172862	30.5789579
57	N	42.5350772	29.3252288	31.5408054
58	C	42.9690490	30.5015321	31.0756850
59	P	46.5475841	29.0320784	35.9329407
60	O	46.8082794	28.1941607	34.6714405
61	O	46.9521144	30.5278170	35.7388731
62	O	47.0860902	28.4508661	37.2516193
63	H	43.1096058	28.8367920	35.2158096
64	H	44.5055760	29.0925052	34.1529268
65	H	44.4917691	31.3333497	35.8920629
66	H	41.7985827	30.6364008	35.9354732
67	H	40.9841133	32.1669993	33.8523557
68	H	41.5860682	30.5119453	33.5388478
69	H	43.1372386	33.1309419	33.2073350
70	H	43.9378838	33.5788785	31.1155688
71	H	42.0710272	27.4201516	30.8879547
72	H	42.9733902	29.1693008	26.8048650
73	H	43.5764623	30.7595883	27.0513472
74	H	42.1081352	32.5040244	36.9671932
75	H	41.1092317	24.9683401	33.7445542
76	H	35.2132222	24.7060082	33.2211558

Off1-RC
QM(B1)/MM Energy = -4319.697449014833 a.u.
QM(B2)/MM Energy = -4322.717226000000 a.u.
QM(B3)/MM Energy = -4322.170332979453 a.u.

1	N	37.3165099	40.8368694	26.4725519
2	C	38.0100768	41.8172366	27.1573900
3	C	38.0138950	43.2747632	26.8171596
4	N	38.4183441	39.7931471	28.0583077
5	C	38.6906935	41.1430983	28.1427250
6	C	37.5738671	39.6450114	27.0418798
7	H	37.0296092	43.6348542	26.4859900
8	H	38.2482646	43.8413858	27.7316848
9	H	39.3484263	41.5400532	28.9092392
10	H	37.1386127	38.7069716	26.7131927
11	H	36.6734197	40.9763444	25.6962480
12	N	42.2176650	38.9037927	25.9553607
13	C	41.2676515	38.2132636	25.2247986
14	C	41.3135448	37.9719437	23.7559352
15	N	40.7167742	38.2338089	27.3936631
16	C	40.3438024	37.7919178	26.1484469
17	C	41.8508255	38.8984406	27.2522357
18	H	42.3422639	37.7489028	23.4359022
19	H	40.7171727	37.0785576	23.5327819
20	H	39.4436866	37.2003420	26.0014036
21	H	42.4109663	39.3807598	28.0508820
22	H	43.0657298	39.3624211	25.6070031
23	Fe	39.4070812	38.2123495	29.0207300
24	O	40.0982954	36.9342373	29.7522850
25	Cl	40.4478654	39.7187431	30.3847084
26	O	36.8222539	37.1615743	30.1427186
27	C	37.0220734	36.7450485	29.0013505
28	O	38.0141738	37.1184274	28.2500857
29	C	36.0823966	35.7487450	28.3542593
30	C	35.8528328	34.5093608	29.2331288
31	C	34.5416368	33.8519686	28.8001930
32	O	34.6247622	32.7724089	28.1392656
33	O	33.4974334	34.4759047	29.0777368
34	H	36.4506230	35.4630553	27.3588312
35	H	35.1182306	36.2710425	28.2216676
36	H	36.6999761	33.8153987	29.1641151
37	H	35.7599955	34.8242689	30.2824823
38	O	37.8589252	45.6356980	33.8980550
39	C	37.7684270	44.5049790	33.0759068
40	C	37.2415575	43.2382206	33.7947071
41	O	36.1747798	42.5994584	33.0496481
42	C	38.2901731	42.1307489	33.9251923
43	O	37.8941587	41.2375087	34.9650686
44	C	38.1636140	41.4661393	32.5522398
45	C	36.6353305	41.4716893	32.3762709
46	N	36.1567475	41.4594026	30.9793863
47	C	35.6563136	40.3473326	30.3447961
48	N	35.1047135	40.5924342	29.1780403
49	C	35.2234424	41.9603635	29.0293310
50	C	34.8739068	42.8504464	27.9919821
51	N	34.2698919	42.4457000	26.8219481
52	N	35.1285402	44.1548309	28.1543706
53	C	35.7626812	44.5651728	29.2671155
54	N	36.2116998	43.8080755	30.2636505
55	C	35.8878713	42.5247271	30.1369583
56	P	36.4433351	46.5695016	34.0442221
57	O	35.9125579	46.7317358	32.5989102
58	O	35.5367295	45.7270210	34.9706837
59	O	36.9621002	47.8868827	34.6540103
60	H	38.7800019	44.3108504	32.6609667
61	H	37.0945338	44.6760350	32.2230929
62	H	36.8174301	43.5532996	34.7572762
63	H	39.2990465	42.5344009	34.1260508
64	H	38.6354411	42.0926264	31.7797379
65	H	38.6128663	40.4654288	32.4897452
66	H	36.2039296	40.5555809	32.8217276
67	H	35.7564596	39.3535754	30.7827821

68 H 35.9400334 45.6426434 29.3571646
69 H 34.2752803 43.1462825 26.0712199
70 H 34.4141740 41.4846356 26.5177709
71 H 38.3920709 40.4106831 34.8841424
72 H 38.7207956 43.5334815 26.0288554
73 H 40.9898851 38.7977138 23.1223619

Off1-meta-InTS
QM(B1)/MM Energy = -4319.686713678297 a.u.
QM(B2)/MM Energy = -4322.702462000000 a.u.
QM(B3)/MM Energy = -4322.156171392624 a.u.

1 N 37.2844430 41.3876171 26.3445822
2 C 37.9212308 42.4017769 27.0392318
3 C 38.0163072 43.8272861 26.6066741
4 N 38.2493177 40.4310561 28.0635696
5 C 38.5142606 41.7823506 28.1079634
6 C 37.4917815 40.2259569 26.9913295
7 H 37.0577997 44.2234093 26.2406485
8 H 38.2872549 44.4390343 27.4808463
9 H 39.1230171 42.2239508 28.8905285
10 H 37.0806525 39.2703041 26.6800006
11 H 36.7430046 41.4923340 25.4901636
12 N 42.0947037 38.8704530 26.1133262
13 C 41.0752488 38.2405398 25.4181342
14 C 41.1254312 37.9567750 23.9519390
15 N 40.5413925 38.4191035 27.6025953
16 C 40.1262830 37.9518018 26.3726117
17 C 41.7364588 38.9604212 27.4075997
18 H 42.1485317 37.6762239 23.6563422
19 H 40.4928279 37.0877256 23.7241545
20 H 39.1692787 37.4494938 26.2579261
21 H 42.3407900 39.4481081 28.1668518
22 H 42.9703487 39.2466780 25.7351805
23 Fe 39.1450233 38.9414281 29.2280398
24 O 39.0487754 38.3926998 30.7720358
25 Cl 40.7484080 40.4183197 29.9716215
26 O 36.5737219 37.3381916 30.2559937
27 C 36.9525151 37.0701758 29.1238752
28 O 37.9328767 37.6753328 28.4917285
29 C 36.2334894 35.9999835 28.3120751
30 C 35.9842615 34.7163946 29.1264437
31 C 34.6610127 34.0518000 28.7214211
32 O 34.7157998 32.9319000 28.1267537
33 O 33.6242983 34.6906347 28.9951584
34 H 36.7659760 35.7932429 27.3722645
35 H 35.2576349 36.4511133 28.0551067
36 H 36.8279009 34.0216762 29.0349951
37 H 35.8884007 34.9950145 30.1863310
38 O 38.0264472 45.4681068 33.7025034
39 C 37.9824822 44.3403437 32.8682857
40 C 37.4611773 43.0530519 33.5594411
41 O 36.2766154 42.5308595 32.9068334
42 C 38.4308988 41.8559145 33.5584149
43 O 38.1133705 41.0456077 34.6827912
44 C 38.0757244 41.1798143 32.2297244
45 C 36.5477671 41.3523390 32.2154405
46 N 35.9707570 41.3976191 30.8587090
47 C 35.4347637 40.3123661 30.2144493
48 N 34.9025888 40.5897216 29.0448268
49 C 35.0722221 41.9519510 28.9110339
50 C 34.7826285 42.8605174 27.8715538
51 N 34.1921063 42.4800178 26.6874828
52 N 35.0769617 44.1555574 28.0509062
53 C 35.6855937 44.5369211 29.1896783
54 N 36.0827503 43.7580732 30.1891656
55 C 35.7391819 42.4816064 30.0343109
56 P 36.6055490 46.3929746 33.8871712
57 O 36.0364854 46.5357461 32.4485171
58 O 35.7211492 45.5616171 34.8387514
59 O 37.1219171 47.7212164 34.4717969
60 H 39.0138898 44.1672499 32.4930218
61 H 37.3340771 44.5103510 31.9942853
62 H 37.1680126 43.3285645 34.5817491
63 H 39.4883887 42.1766459 33.6011267
64 H 38.5120319 41.7735068 31.4110960
65 H 38.4206354 40.1130494 32.0690849
66 H 36.0501748 40.4966426 32.7072470
67 H 35.5042629 39.3141589 30.6446050
68 H 35.8880890 45.6089157 29.2998915
69 H 34.2332140 43.1898287 25.9472163
70 H 34.3438729 41.5229311 26.3698147
71 H 38.4416061 40.1434869 34.5537032
72 H 38.7558400 43.9521227 25.8157416
73 H 40.8652875 38.7773614 23.2833358

Off1-meta-InRC
QM(B1)/MM Energy = -4319.696507685440 a.u.
QM(B2)/MM Energy = -4322.713531000000 a.u.
QM(B3)/MM Energy = -4322.167662224179 a.u.

1 N 37.1712694 41.2414349 26.2742916
2 C 37.8688754 42.1862764 27.0008421
3 C 37.9696724 43.6390168 26.6655354
4 N 38.1928059 40.1424274 27.8663567
5 C 38.4963659 41.4771688 27.9956836
6 C 37.3802341 40.0281766 26.8278318
7 H 37.0149045 44.0601196 26.3182335
8 H 38.2318870 44.1926161 27.5802134
9 H 39.1480789 41.8407957 28.7861808
10 H 36.9114985 39.1157332 26.4682475
11 H 36.5770782 41.4185416 25.4680754
12 N 42.0590473 38.9495329 26.0436919
13 C 41.0713474 38.2860056 25.3370426
14 C 41.1475145 37.9895168 23.8769409
15 N 40.4792856 38.4978781 27.4990594

16 C	40.1043573	37.9971187	26.2711912
17 C	41.6677806	39.0596903	27.3266460
18 H	42.1782523	37.7266997	23.5938065
19 H	40.5296721	37.1104020	23.6503616
20 H	39.1685362	37.4599935	26.1371213
21 H	42.2441379	39.5545570	28.1033926
22 H	42.9435434	39.3228405	25.6839780
23 Fe	39.1362837	38.7748739	29.1185741
24 O	38.1569021	39.0955441	30.3582097
25 Cl	40.8377658	39.9623879	30.1703790
26 O	36.9805339	36.7832908	30.4528147
27 C	37.5305610	36.3817291	29.4426377
28 O	38.5977576	36.9377352	28.8850661
29 C	36.9823265	35.1911570	28.6595496
30 C	36.0806117	34.2566591	29.4962596
31 C	34.7413576	33.9863416	28.7970518
32 O	34.6583079	32.9261803	28.0971634
33 O	33.8471146	34.8440638	28.9336670
34 H	37.8173816	34.6636079	28.1790324
35 H	36.3997930	35.6527092	27.8398789
36 H	36.5973380	33.3109465	29.7067976
37 H	35.8644524	34.7525303	30.4502154
38 O	37.9520846	45.7609659	33.8162161
39 C	37.8803883	44.6237004	33.0008676
40 C	37.3301882	43.3633699	33.7135893
41 O	36.2058534	42.7926185	32.9951598
42 C	38.3368349	42.2102271	33.7834050
43 O	37.9669991	41.3332170	34.8429367
44 C	38.1132691	41.5583096	32.4199779
45 C	36.5801677	41.6286649	32.3178953
46 N	36.0487798	41.6237864	30.9453055
47 C	35.5456959	40.5095073	30.3168310
48 N	35.0023474	40.7505085	29.1462442
49 C	35.1319744	42.1142240	28.9898669
50 C	34.7964420	42.9926862	27.9402761
51 N	34.1871404	42.5691830	26.7806375
52 N	35.0616974	44.2983797	28.0858516
53 C	35.6751167	44.7192644	29.2075839
54 N	36.0990908	43.9738075	30.2235441
55 C	35.7877447	42.6847153	30.0987598
56 P	36.5228998	46.6647270	33.9963066
57 O	35.9521126	46.7824913	32.5571049
58 O	35.6509023	45.8233184	34.9520288
59 O	37.0289433	47.9991955	34.5724595
60 H	38.9037426	44.4243199	32.6195281
61 H	37.2294592	44.7902114	32.1282418
62 H	36.9614004	43.6696465	34.7009019
63 H	39.3708854	42.5742630	33.9284780
64 H	38.5708935	42.1699991	31.6264941
65 H	38.5118762	40.5397702	32.3220447
66 H	36.1236097	40.7453067	32.7999129
67 H	35.6335210	39.5227023	30.7692953
68 H	35.8560214	45.7977331	29.2871257
69 H	34.2002486	43.2514717	26.0142749
70 H	34.3280931	41.6010695	26.4990645
71 H	38.3636264	40.4606035	34.7021638
72 H	38.7104833	43.8205789	25.8868818
73 H	40.8774059	38.8078132	23.2094835

Off1-TS1

QM(B1)/MM Energy = -4319.657124489926 a.u.

QM(B2)/MM Energy = -4322.673607000000 a.u.

QM(B3)/MM Energy = -4322.133585044438 a.u.

1 N	37.1991737	41.2748226	26.3657060
2 C	37.8878402	42.2483357	27.0644648
3 C	37.9918594	43.6873530	26.6759450
4 N	38.1627257	40.2435286	28.0396589
5 C	38.4829253	41.5807818	28.1063354
6 C	37.3791786	40.0890498	26.9837177
7 H	37.0342922	44.0992682	26.3248652
8 H	38.2634484	44.2702424	27.5696600
9 H	39.1345708	41.9745675	28.8825614
10 H	36.9118955	39.1641644	26.6552289
11 H	36.6266324	41.4189559	25.5377104
12 N	42.0530147	38.9602412	26.0763050
13 C	41.0380780	38.3139395	25.3942233
14 C	41.0997887	37.9936492	23.9367276
15 N	40.4871706	38.5620562	27.5707999
16 C	40.0811246	38.0615493	26.3522315
17 C	41.6838679	39.0902930	27.3661796
18 H	42.1251829	37.7093143	23.6543110
19 H	40.4676988	37.1212771	23.7206429
20 H	39.1306344	37.5449290	26.2357341
21 H	42.2893372	39.5788548	28.1248978
22 H	42.9373936	39.3128275	25.6979369
23 Fe	39.1134707	38.9280269	29.3761099
24 O	38.2548241	39.3772154	30.8312985
25 Cl	41.0161231	40.0757410	30.2105617
26 O	36.7730742	36.9869120	30.4794788
27 C	37.4356863	36.5378443	29.5580717
28 O	38.5468329	37.0850150	29.0986105
29 C	36.9843714	35.2999546	28.7805158
30 C	36.0582242	34.3507202	29.5760486
31 C	34.7468439	34.0485031	28.8324014
32 O	34.6972801	32.9750900	28.1497327
33 O	33.8332852	34.8914500	28.9287399
34 H	37.8675029	34.7852236	28.3774672
35 H	36.4463982	35.7159975	27.9077042
36 H	36.5844740	33.4143377	29.8052901
37 H	35.7914190	34.8432587	30.5193521
38 O	37.9994859	45.5993258	33.7286075
39 C	37.9571993	44.4778014	32.8932449
40 C	37.4064202	43.1956436	33.5686617
41 O	36.1976448	42.7176486	32.9135597
42 C	38.3679255	41.9783577	33.5133082

43 O	38.0206232	41.0960885	34.5681692
44 C	37.9829627	41.4655286	32.1378433
45 C	36.4623776	41.5619414	32.1704103
46 N	35.8623484	41.6005078	30.8403283
47 C	35.3656935	40.4937455	30.1963360
48 N	34.8522642	40.7534927	29.0177168
49 C	35.0035090	42.1161341	28.8719467
50 C	34.7290450	43.0002209	27.8101337
51 N	34.1620916	42.5835122	26.6302569
52 N	35.0203268	44.2998011	27.9680684
53 C	35.6059799	44.7060941	29.1100088
54 N	35.9798607	43.9506782	30.1383806
55 C	35.6413409	42.6707490	29.9977367
56 P	36.5714374	46.5125575	33.9165671
57 O	35.9982348	46.6435793	32.4810248
58 O	35.7024281	45.6684624	34.8728444
59 O	37.0799828	47.8416874	34.5026354
60 H	38.9930384	44.2922042	32.5390316
61 H	37.3258678	44.6548021	32.0061804
62 H	37.1350279	43.4446094	34.6040833
63 H	39.4265920	42.2846168	33.5838341
64 H	38.4183765	42.0832786	31.3392182
65 H	38.2809707	40.2901350	31.6854327
66 H	36.0247114	40.6782095	32.6679711
67 H	35.4530088	39.5003583	30.6358989
68 H	35.8102397	45.7797606	29.1971350
69 H	34.1984653	43.2678955	25.8670129
70 H	34.2977890	41.6121440	26.3535510
71 H	38.3894691	40.2135867	34.4063670
72 H	38.7253515	43.8466302	25.8855734
73 H	40.8461309	38.8050800	23.2545950

Off1-IM1
QM(B1)/MM Energy = -4319.695388291268 a.u.
QM(B2)/MM Energy = -4322.714699000000 a.u.
QM(B3)/MM Energy = -4322.166631711479 a.u.

1 N	37.1548783	41.0105174	26.4158936
2 C	37.8536605	42.0005509	27.0865231
3 C	37.9141262	43.4475672	26.7135381
4 N	38.2013038	40.0030907	28.0584661
5 C	38.4963835	41.3497140	28.1080973
6 C	37.3727220	39.8339023	27.0317665
7 H	36.9493846	43.8343362	26.3544747
8 H	38.1530663	44.0243272	27.6204441
9 H	39.1576635	41.7567595	28.8665752
10 H	36.9161698	38.8923930	26.7386736
11 H	36.5310738	41.1340242	25.6211008
12 N	42.1364382	38.8435139	26.0228415
13 C	41.1773356	38.1586008	25.2926962
14 C	41.2329359	37.9306597	23.8199531
15 N	40.6299094	38.1878265	27.4713613
16 C	40.2546496	37.7425488	26.2207730
17 C	41.7722544	38.8465762	27.3165044
18 H	42.2624461	37.6938177	23.5092931
19 H	40.6263316	37.0486541	23.5776767
20 H	39.3423606	37.1671304	26.0816647
21 H	42.3184937	39.3510425	28.1096800
22 H	42.9890095	39.2923037	25.6703120
23 Fe	39.2163664	38.4290331	28.9999108
24 O	39.3513971	37.5470143	30.6390203
25 Cl	40.6728710	40.1138250	30.0942297
26 O	36.3115113	37.2126379	29.6988685
27 C	37.0734839	36.5425978	29.0202613
28 O	38.0238743	37.0761297	28.2718597
29 C	37.0865278	35.0209618	29.0021010
30 C	35.9524794	34.2982653	29.7439323
31 C	34.6966144	34.0450513	28.8896871
32 O	34.7603453	33.0585141	28.0844571
33 O	33.7137522	34.7960810	29.0344479
34 H	38.0561092	34.7535059	29.4570184
35 H	37.1620157	34.6860480	27.9553889
36 H	36.3329397	33.3168687	30.0668902
37 H	35.6756105	34.8786546	30.6334297
38 O	37.9600507	45.7593833	33.7192420
39 C	37.8313474	44.6247858	32.9028106
40 C	37.3470314	43.3694222	33.6629114
41 O	36.1792366	42.7665645	33.0348303
42 C	38.3764260	42.2303644	33.7192110
43 O	38.1711790	41.4783268	34.9262943
44 C	37.9851054	41.4287676	32.5234979
45 C	36.5050574	41.5968532	32.3526826
46 N	36.0358113	41.6095489	30.9444215
47 C	35.5661942	40.4912550	30.2928814
48 N	35.0266817	40.7391769	29.1233509
49 C	35.1206156	42.1070887	28.9898747
50 C	34.7751448	42.9894208	27.9460826
51 N	34.1921727	42.5648418	26.7759461
52 N	35.0085237	44.2988598	28.1094003
53 C	35.5971387	44.7203706	29.2436317
54 N	36.0240389	43.9723430	30.2565428
55 C	35.7508060	42.6781304	30.1127122
56 P	36.5434559	46.6697432	33.9624528
57 O	35.9332245	46.8128965	32.5417959
58 O	35.6909453	45.8163967	34.9263984
59 O	37.0692964	47.9918475	34.5475158
60 H	38.8186920	44.4283585	32.4368566
61 H	37.1119304	44.7925239	32.0877769
62 H	37.0305422	43.6921722	34.6634559
63 H	39.4155309	42.6025662	33.6866599
64 H	38.6118686	40.7391820	31.9546154
65 H	39.5872612	38.2246491	31.2925574
66 H	35.9535057	40.7437675	32.8026073
67 H	35.6748838	39.4919790	30.7156380
68 H	35.7523436	45.8015748	29.3387574
69 H	34.1980375	43.2484525	26.0106982

70 H	34.3287707	41.5953002	26.4977737
71 H	38.5087861	40.5773629	34.8151936
72 H	38.6493778	43.6599113	25.9374053
73 H	40.9335239	38.7628653	23.1828868

Off1-TS2
QM(B1)/MM Energy = -4319.680017932885 a.u.
QM(B2)/MM Energy = -4322.698831000000 a.u.
QM(B3)/MM Energy = -4322.154842308773 a.u.

1 N	37.1227763	41.0100632	26.5563189
2 C	37.9045974	41.9837983	27.1501059
3 C	37.9638564	43.4247927	26.7516520
4 N	38.2175280	39.9979010	28.1611452
5 C	38.5836392	41.3244731	28.1473625
6 C	37.3213761	39.8400447	27.1979123
7 H	36.9940290	43.8052424	26.4015775
8 H	38.2126092	44.0178414	27.6458559
9 H	39.3056026	41.7268730	28.8546137
10 H	36.7776866	38.9234635	26.9796468
11 H	36.4432488	41.1440656	25.8094812
12 N	42.0871066	38.9331138	26.1019282
13 C	41.1233294	38.2661762	25.3642779
14 C	41.2154690	37.9801086	23.9011072
15 N	40.5233535	38.3515379	27.5323960
16 C	40.1639690	37.9073058	26.2833408
17 C	41.6872160	38.9632448	27.3927423
18 H	42.2529352	37.7358400	23.6242253
19 H	40.6180839	37.0872946	23.6715895
20 H	39.2449410	37.3487134	26.1169157
21 H	42.2471426	39.4214907	28.2052430
22 H	42.9586527	39.3482891	25.7568466
23 Fe	39.2168733	38.5704652	29.3159849
24 O	40.8677251	38.9318959	30.2302558
25 Cl	38.1694861	39.1786517	31.3752666
26 O	36.4393004	37.1221782	29.2951846
27 C	37.3737356	36.3371037	29.1934472
28 O	38.6222871	36.7215708	29.0266316
29 C	37.2116007	34.8233145	29.2079763
30 C	35.9353663	34.2498680	29.8495433
31 C	34.7184706	34.0694447	28.9169437
32 O	34.7822990	33.0956752	28.0962360
33 O	33.7499747	34.8416807	29.0436896
34 H	38.1067858	34.4110848	29.6952186
35 H	37.2942598	34.4916294	28.1589266
36 H	36.1870617	33.2444585	30.2245084
37 H	35.6358640	34.8716722	30.7034128
38 O	37.9740878	45.7472127	33.7276130
39 C	37.8916144	44.6219192	32.9010597
40 C	37.3216114	43.3638819	33.6001827
41 O	36.1275751	42.8660283	32.9290631
42 C	38.2861394	42.1502573	33.5971682
43 O	37.9212385	41.2818424	34.6600006
44 C	37.9242809	41.5985171	32.2451688
45 C	36.4196395	41.7064940	32.1929995
46 N	35.8712278	41.7445446	30.8396538
47 C	35.3505651	40.6403854	30.2059367
48 N	34.8377932	40.8967295	29.0292372
49 C	35.0066795	42.2550941	28.8731684
50 C	34.7295663	43.1264115	27.8038058
51 N	34.1593864	42.6880875	26.6354680
52 N	35.0253704	44.4267503	27.9451878
53 C	35.6231760	44.8403741	29.0776744
54 N	36.0055612	44.0925639	30.1111194
55 C	35.6570434	42.8133435	29.9889937
56 P	36.5486180	46.6548611	33.9455387
57 O	35.9536583	46.7851881	32.5185363
58 O	35.6963368	45.8011453	34.9090639
59 O	37.0667875	47.9807016	34.5277324
60 H	38.9140896	44.4102265	32.5248517
61 H	37.2449174	44.8047529	32.0270822
62 H	37.0213295	43.6451596	34.6191133
63 H	39.3409895	42.4646887	33.6842179
64 H	38.5054607	41.8961907	31.3717387
65 H	40.5297799	39.3582466	31.0347968
66 H	35.9495962	40.8310382	32.6756447
67 H	35.4152505	39.6505125	30.6536089
68 H	35.8314055	45.9141478	29.1519482
69 H	34.1849334	43.3504525	25.8529845
70 H	34.2608815	41.7051014	26.3882325
71 H	38.2577421	40.3888545	34.4881123
72 H	38.6836630	43.6335412	25.9602168
73 H	40.9293948	38.7855625	23.2247399

Off-2'-Cl-dAMP
QM(B1)/MM Energy = -4319.742267468399 a.u.
QM(B2)/MM Energy = -4322.763912000000 a.u.
QM(B3)/MM Energy = -4322.205824008754 a.u.

1 N	37.1643328	40.7770371	26.4903380
2 C	37.9121655	41.7312511	27.1507850
3 C	37.9454464	43.1900464	26.8138077
4 N	38.2297599	39.6981343	28.0836298
5 C	38.5686995	41.0322667	28.1401706
6 C	37.3724219	39.5790359	27.0831130
7 H	36.9692943	43.5615569	26.4721858
8 H	38.1750885	43.7523692	27.7328248
9 H	39.2633932	41.4093533	28.8880964
10 H	36.8722163	38.6637094	26.7737505
11 H	36.5081768	40.9321603	25.7273783
12 N	42.1986908	38.8820832	25.9545926
13 C	41.2582711	38.1886544	25.2157988
14 C	41.3091232	37.9589709	23.7448868
15 N	40.7041162	38.1629821	27.3932217
16 C	40.3444115	37.7392887	26.1393313
17 C	41.8252454	38.8456643	27.2546253

18 H	42.3392784	37.7375352	23.4262780
19 H	40.7139958	37.0666751	23.5140539
20 H	39.4552562	37.1336070	25.9733705
21 H	42.3744627	39.3154674	28.0686802
22 H	43.0395535	39.3562004	25.6124473
23 Fe	39.4036216	38.2317530	29.0732495
24 O	40.7895145	38.7925019	30.2712982
25 Cl	38.7826106	40.1624374	32.2290446
26 O	36.9409718	36.8500339	30.2350025
27 C	37.5779203	36.0646130	29.5279538
28 O	38.6698707	36.3875540	28.9017116
29 C	37.2038711	34.5980894	29.3420577
30 C	35.8940126	34.0960459	29.9678667
31 C	34.7012784	34.0627914	28.9919904
32 O	34.6720303	33.0817071	28.1775591
33 O	33.8555625	34.9754670	29.0573484
34 H	38.0648716	34.0380015	29.7411668
35 H	37.2385367	34.3960483	28.2615652
36 H	36.0510795	33.0649613	30.3230708
37 H	35.6397117	34.7275379	30.8282915
38 O	37.9241693	45.9169493	33.7986806
39 C	37.8482603	44.7792859	32.9954338
40 C	37.2604929	43.5429512	33.7190513
41 O	36.1475935	42.9672465	32.9719999
42 C	38.2638988	42.3927662	33.8308624
43 O	37.8661172	41.4882062	34.8379277
44 C	38.0695273	41.8002518	32.4357850
45 C	36.5334351	41.8005160	32.3168478
46 N	36.0418070	41.7486538	30.9440621
47 C	35.5631739	40.6101317	30.3275170
48 N	35.0298257	40.8290619	29.1522664
49 C	35.1379467	42.1927602	28.9737599
50 C	34.7976470	43.0496987	27.9066081
51 N	34.2199233	42.6006763	26.7472538
52 N	35.0386817	44.3623283	28.0403470
53 C	35.6420121	44.8100338	29.1539100
54 N	36.0728494	44.0854469	30.1852928
55 C	35.7738704	42.7931908	30.0751007
56 P	36.4784937	46.7977185	33.9820705
57 O	35.9058115	46.8937266	32.5440974
58 O	35.6288743	45.9337556	34.9397349
59 O	36.9747046	48.1331865	34.5574389
60 H	38.8750232	44.5578403	32.6355353
61 H	37.2112136	44.9401966	32.1108140
62 H	36.8642659	43.8702421	34.6878648
63 H	39.2939692	42.7532859	33.9991337
64 H	38.5162126	42.4443576	31.6671017
65 H	40.4494123	39.4527807	30.8901169
66 H	36.1277985	40.9050871	32.8203000
67 H	35.6619577	39.6278555	30.7912681
68 H	35.8087587	45.8917915	29.2156018
69 H	34.2140953	43.2699425	25.9690992
70 H	34.3435692	41.6236172	26.4899867
71 H	38.3177269	40.6375939	34.7127571
72 H	38.6551718	43.4618298	26.0324685
73 H	40.9878014	38.7852830	23.1108303

Off1-TS2'
QM(B1)/MM Energy = -4319.674688303870 a.u.
QM(B2)/MM Energy = -4322.691666000000 a.u.
QM(B3)/MM Energy = -4322.147927561659 a.u.

1 N	37.2370742	41.0984826	26.6185085
2 C	37.9351211	42.1249092	27.2258308
3 C	37.9768883	43.5496111	26.7733167
4 N	38.2711049	40.1891457	28.3254712
5 C	38.5780219	41.5305650	28.2847333
6 C	37.4528022	39.9596001	27.3090838
7 H	37.0003261	43.9092636	26.4176207
8 H	38.2301922	44.1775950	27.6416961
9 H	39.2422271	41.9932130	29.0102790
10 H	36.9828209	39.0101284	27.0655048
11 H	36.6188913	41.1770368	25.8136973
12 N	42.1054729	38.9691417	26.3909131
13 C	41.1043821	38.2618253	25.7418139
14 C	41.1434887	37.8843795	24.2898768
15 N	40.6155096	38.4849523	27.9416235
16 C	40.1974703	37.9608606	26.7358260
17 C	41.7721598	39.0811471	27.6970425
18 H	42.1577639	37.5500666	24.0139717
19 H	40.4910239	37.0145349	24.1218877
20 H	39.2796922	37.3823173	26.6588956
21 H	42.3831237	39.5902580	28.4408441
22 H	42.9598101	39.3582025	25.9797961
23 Fe	39.1978922	38.8946007	29.7016191
24 O	38.3355176	39.4688468	31.2716988
25 Cl	41.2182628	39.5644044	30.9022550
26 O	36.4819991	37.1744313	30.3387166
27 C	37.3426592	36.5513648	29.7376893
28 O	38.5035037	37.0689907	29.3856284
29 C	37.1769893	35.0844062	29.3208973
30 C	35.9640668	34.3272178	29.9102891
31 C	34.7443308	34.1533765	28.9764209
32 O	34.7402326	33.1158346	28.2354502
33 O	33.8413209	35.0094888	29.0121026
34 H	38.1117965	34.5766422	29.6036711
35 H	37.1746793	35.0568897	28.2162017
36 H	36.2979630	33.3174064	30.1948039
37 H	35.6291762	34.8537213	30.8139877
38 O	37.9637541	45.8732844	33.6414842
39 C	37.9041907	44.7609345	32.7954638
40 C	37.6728912	43.4168498	33.5264308
41 O	36.4710451	42.7388016	33.0644606
42 C	38.8193041	42.3992216	33.2889420
43 O	38.9916416	41.5000691	34.3812942
44 C	38.2811865	41.7376352	32.0398166

45 C	36.7911857	41.6578521	32.2239565
46 N	36.0438426	41.6628562	30.9627897
47 C	35.5412313	40.5327610	30.3594195
48 N	34.9565728	40.7635225	29.2066059
49 C	35.0641547	42.1282282	29.0332949
50 C	34.7407602	42.9906079	27.9650521
51 N	34.1618652	42.5555042	26.7975600
52 N	35.0032223	44.3000943	28.0957655
53 C	35.6105861	44.7391455	29.2110007
54 N	36.0459867	44.0043290	30.2312324
55 C	35.7410994	42.7140789	30.1200508
56 P	36.5105238	46.7289902	33.8750250
57 O	35.8841199	46.8008054	32.4563068
58 O	35.7152562	45.8758247	34.8838938
59 O	36.9897328	48.0945109	34.3980347
60 H	38.8607928	44.7120462	32.2332386
61 H	37.0973334	44.8584506	32.0543885
62 H	37.5383116	43.6295211	34.5966087
63 H	39.7603170	42.9339011	33.0932253
64 H	38.6966207	41.9810035	31.0649040
65 H	38.9140585	39.5063980	32.0482098
66 H	36.5144036	40.7063053	32.7140845
67 H	35.6943636	39.5453646	30.7958005
68 H	35.7807946	45.8199684	29.2801081
69 H	34.1785576	43.2325188	26.0264093
70 H	34.2947342	41.5831078	26.5248133
71 H	39.9458427	41.4548062	34.5747756
72 H	38.6954711	43.7225479	25.9721932
73 H	40.9020492	38.6540639	23.5568284

Off1-2'-OH-dAMP
QM(B1)/MM Energy = -4319.775416118612 a.u.
QM(B2)/MM Energy = -4322.787435000000 a.u.
QM(B3)/MM Energy = -4322.228443126526 a.u.

1 N	37.3755167	41.2195367	26.7618415
2 C	38.0271477	42.2909684	27.3381655
3 C	38.0474776	43.6897585	26.8106726
4 N	38.4105156	40.4106845	28.5277457
5 C	38.6684178	41.7597251	28.4328780
6 C	37.6232061	40.1167618	27.5046049
7 H	37.0597999	44.0269822	26.4630010
8 H	38.3217575	44.3646486	27.6367977
9 H	39.3013305	42.2809661	29.1469761
10 H	37.2009955	39.1402706	27.2815875
11 H	36.7863486	41.2482414	25.9335403
12 N	42.1820584	39.0048629	26.4118056
13 C	41.1380400	38.3315575	25.7974407
14 C	41.1327057	37.9208829	24.3528130
15 N	40.7141279	38.6010927	28.0068005
16 C	40.2443151	38.0829245	26.8185810
17 C	41.8867127	39.1436532	27.7264350
18 H	42.1322586	37.5554467	24.0633085
19 H	40.4572347	37.0620265	24.2208230
20 H	39.3035148	37.5393537	26.7701914
21 H	42.5406926	39.6316896	28.4476624
22 H	43.0362796	39.3637942	25.9750599
23 Fe	39.4458409	39.0570569	29.8255858
24 O	38.6643348	40.2420718	31.7033919
25 Cl	41.4283462	39.4522639	31.1393758
26 O	36.6053729	37.3046461	30.8210559
27 C	37.3020341	36.8763289	29.9021396
28 O	38.4818257	37.3425025	29.6033448
29 C	36.7634089	35.7858113	28.9667184
30 C	36.0167786	34.6187687	29.6547668
31 C	34.7133412	34.2348815	28.9209279
32 O	34.6515704	33.1077214	28.3356959
33 O	33.8010441	35.0858629	28.9513670
34 H	37.5724177	35.4208909	28.3179263
35 H	36.0408336	36.3260253	28.3267462
36 H	36.6783954	33.7466656	29.7629419
37 H	35.7126510	34.9532480	30.6579992
38 O	38.0160862	45.7985755	33.6201848
39 C	38.0637327	44.6968829	32.7666395
40 C	37.7827964	43.3372785	33.4600263
41 O	36.5207356	42.7584153	33.0272314
42 C	38.8498391	42.2653020	33.1511062
43 O	39.0827141	41.3219469	34.1908170
44 C	38.2149262	41.5565586	31.9448182
45 C	36.7111167	41.6168154	32.2585364
46 N	35.9289298	41.6140266	31.0194057
47 C	35.4377342	40.4831299	30.4086617
48 N	34.8934197	40.7102124	29.2350211
49 C	35.0233324	42.0718741	29.0511935
50 C	34.7396238	42.9341937	27.9684458
51 N	34.1861521	42.5077574	26.7884079
52 N	35.0156562	44.2408278	28.1037900
53 C	35.5999080	44.6785182	29.2319537
54 N	35.9956123	43.9443898	30.2678451
55 C	35.6728329	42.6588818	30.1531634
56 P	36.5393656	46.6373685	33.7864560
57 O	35.9683891	46.6930936	32.3466677
58 O	35.7231082	45.7849285	34.7780736
59 O	36.9909855	48.0099254	34.3187210
60 H	39.0822893	44.6655571	32.3233712
61 H	37.3390727	44.7851391	31.9413484
62 H	37.7084633	43.5273264	34.5402628
63 H	39.7986819	42.7443122	32.8710297
64 H	38.3957955	42.1665994	31.0494890
65 H	39.0689568	39.9346446	32.5362640
66 H	36.4113232	40.7013753	32.8030898
67 H	35.5573060	39.4970006	30.8609276
68 H	35.7828792	45.7570352	29.3025442
69 H	34.2115468	43.1929636	26.0244784
70 H	34.3122834	41.5354607	26.5089917
71 H	40.0138213	41.4032386	34.4748603

72 H 38.7461253 43.8184981 25.9840017
73 H 40.8944801 38.6766162 23.6043594

Off2-RC
QM(B1)/MM Energy = -4320.454151771458 a.u.
QM(B2)/MM Energy = -4323.470526000000 a.u.
QM(B3)/MM Energy = -4322.924326711517 a.u.

1 N 46.7995207 27.4665163 37.1120945
2 C 46.0146022 27.6832634 38.2288648
3 C 46.1819396 26.9867298 39.5437143
4 N 45.2922102 28.9476241 36.5109367
5 C 45.0810342 28.6093719 37.8293499
6 C 46.3423476 28.2458788 36.1051032
7 H 47.2373143 26.7559535 39.7506187
8 H 45.8458894 27.6577108 40.3491369
9 H 44.2829866 29.0662700 38.4063777
10 H 46.7803617 28.2930004 35.1114028
11 H 47.6149928 26.8603272 37.0673593
12 N 41.3786294 27.0444142 34.1517638
13 C 42.5296707 26.2803688 34.2083899
14 C 42.6110412 24.8839698 33.6814889
15 N 42.9428942 28.3688302 34.9254583
16 C 43.4899259 27.1319682 34.7014369
17 C 41.6699932 28.2964014 34.5734235
18 H 41.5982521 24.4686405 33.5605469
19 H 43.0567972 24.8895379 32.6760078
20 H 44.5369223 26.9290089 34.9000924
21 H 40.9534873 29.1133504 34.5796413
22 H 40.4504519 26.7510367 33.8501291
23 Fe 44.0307588 30.1706399 35.3244692
24 O 43.0036604 31.0006432 34.3878757
25 Cl 43.1315781 31.0316514 37.2627986
26 O 45.6755913 31.6017026 34.7640443
27 C 45.9144404 30.8250166 33.7987487
28 O 45.3214628 29.6943209 33.7688400
29 C 46.7923745 31.1992784 32.6340177
30 C 45.9584778 31.2559694 31.3476839
31 C 46.7621486 31.1146881 30.0499510
32 O 47.9827354 30.8647247 30.1224355
33 O 46.1001143 31.2212905 28.9751108
34 H 47.5645989 30.4260609 32.5073675
35 H 47.2986302 32.1550285 32.8325220
36 H 45.2219753 30.4349798 31.3486966
37 H 45.3665480 32.1844186 31.2931169
38 O 45.6355019 32.8778410 44.4389114
39 C 45.9708246 32.5817759 43.0939143
40 C 45.3389237 33.5596318 42.0932361
41 O 46.0719644 33.5622167 40.8506412
42 C 43.9124331 33.1891998 41.6761690
43 O 43.2560620 34.3634496 41.2193098
44 C 44.1806688 32.1996659 40.5329625
45 C 45.4269321 32.8186286 39.8694343
46 N 46.3658468 31.8510757 39.2633997
47 C 46.7272221 31.8101204 37.9408662
48 N 47.6901851 30.9544039 37.6673372
49 C 47.9940221 30.3907938 38.8912802
50 C 48.8808280 29.3699667 39.2967475
51 N 49.7405579 28.7201933 38.4519187
52 N 48.9204174 29.0422805 40.5980498
53 C 48.0941990 29.6540758 41.4585532
54 N 47.1823712 30.5770562 41.1809700
55 C 47.1769816 30.9346153 39.9004171
56 P 46.8963392 33.5930867 45.3333044
57 O 47.1068244 34.9666194 44.6703039
58 O 46.2919276 33.6509832 46.7513629
59 O 48.0848589 32.6150905 45.2032837
60 H 45.6585265 31.5488144 42.8763153
61 H 47.0586045 32.6096522 42.9368960
62 H 45.3941242 34.5732798 42.5092819
63 H 43.3706182 32.7274339 42.5251696
64 H 44.4099365 31.2004386 40.9302313
65 H 43.3503507 32.1040562 39.8200114
66 H 45.1289283 33.4686730 39.0296742
67 H 46.2081651 32.4194647 37.2038275
68 H 48.1786044 29.3521328 42.5081283
69 H 50.1006563 27.8390038 38.8273305
70 H 49.5495555 28.7200971 37.4547742
71 H 42.4143559 34.1386512 40.7976225
72 H 45.6314132 26.0476932 39.6002670
73 H 43.1349338 24.1755537 34.3231667

Off2-meta-InTS
QM(B1)/MM Energy = -4320.428471599934 a.u.
QM(B2)/MM Energy = -4323.447206000000 a.u.
QM(B3)/MM Energy = -4322.902949964928 a.u.

1 N 46.8181068 27.2794977 37.5234859
2 C 46.1020579 27.4782453 38.6919149
3 C 46.2454223 26.6814038 39.9475349
4 N 45.2969428 28.7901656 37.0557076
5 C 45.1673216 28.4303936 38.3778334
6 C 46.3157387 28.0912877 36.5688584
7 H 47.2890134 26.3994858 40.1403821
8 H 45.9185537 27.2923872 40.8021550
9 H 44.3928740 28.8501491 39.0118303
10 H 46.7125620 28.1819195 35.5608388
11 H 47.6097971 26.6499040 37.4125195
12 N 41.3529355 26.9801832 34.4423421
13 C 42.5733642 26.4039305 34.1388083
14 C 42.7135689 25.0003997 33.6447480
15 N 42.8552219 28.5395032 34.7929221
16 C 43.4940172 27.4054790 34.3491767
17 C 41.5632820 28.2583986 34.8217982
18 H 41.7182690 24.5743277 33.4462264
19 H 43.2336271 25.0012015 32.6768000

20 H	44.5719683	27.3802043	34.2148915
21 H	40.7705446	28.9381819	35.1259217
22 H	40.4311592	26.5483592	34.3779199
23 Fe	43.9487875	30.0239535	35.9794901
24 O	43.6089596	31.6156113	36.0663530
25 Cl	42.3218775	29.6145651	37.5897264
26 O	45.9229371	32.1361923	34.3624138
27 C	45.8503217	30.9893528	33.9465939
28 O	45.1833332	30.0210751	34.5411245
29 C	46.5333508	30.5696781	32.6498701
30 C	45.8682531	31.1906123	31.4177142
31 C	46.6883856	31.0574485	30.1208187
32 O	47.9173485	30.8554712	30.2062397
33 O	46.0463289	31.1824393	29.0359468
34 H	46.5576891	29.4733874	32.5715938
35 H	47.5742247	30.9209560	32.6895843
36 H	44.8714221	30.7599013	31.2341318
37 H	45.7152523	32.2720956	31.5870891
38 O	45.3991699	32.8549854	43.8133080
39 C	45.7687319	32.5134390	42.4940338
40 C	45.3130909	33.5342460	41.4303900
41 O	46.2521398	33.5743738	40.3296206
42 C	43.9606558	33.2768466	40.7325156
43 O	43.4562595	34.5372196	40.3298250
44 C	44.3763980	32.3842715	39.5450531
45 C	45.7574158	32.9632765	39.1900704
46 N	46.7200597	31.9525201	38.6892423
47 C	47.1529192	31.8195183	37.3951223
48 N	48.0630689	30.8802024	37.2286693
49 C	48.2481761	30.3568497	38.4931858
50 C	49.0380114	29.3021603	39.0069513
51 N	49.9154527	28.5695088	38.2560402
52 N	48.9591494	29.0304224	40.3199813
53 C	48.1133621	29.7332289	41.0901950
54 N	47.3001877	30.7067785	40.7083816
55 C	47.4105285	31.0068722	39.4185451
56 P	46.6194323	33.4147536	44.8436470
57 O	46.8669423	34.8765766	44.4345016
58 O	45.9569418	33.2486082	46.2435459
59 O	47.8286936	32.4700358	44.6569039
60 H	45.3599580	31.5126610	42.2662541
61 H	46.8589450	32.4270120	42.4012056
62 H	45.3193401	34.5303225	41.8944613
63 H	43.2538392	32.7578341	41.4105012
64 H	44.4923821	31.3491671	39.9005751
65 H	43.7024993	32.3580134	38.6430485
66 H	45.6749768	33.6910322	38.3629013
67 H	46.7172179	32.4120139	36.5911992
68 H	48.0880890	29.4685405	42.1535144
69 H	50.2124756	27.7014190	38.7095152
70 H	49.7697128	28.5084397	37.2515537
71 H	42.7595934	34.4259953	39.6676398
72 H	45.6564314	25.7656354	39.8972859
73 H	43.1918310	24.2956637	34.3249536

Off2-meta-InRC

QM(B1)/MM Energy = -4320.448329419379 a.u.

QM(B2)/MM Energy = -4323.467157000000 a.u.

QM(B3)/MM Energy = -4322.921448123569 a.u.

1 N	46.7646146	27.4106410	37.3615682
2 C	46.0071943	27.6161785	38.4999999
3 C	46.1826930	26.8765493	39.7893301
4 N	45.1947995	28.8321256	36.7950594
5 C	45.0373266	28.5146434	38.1225722
6 C	46.2546003	28.1662854	36.3623224
7 H	47.2366795	26.6289370	39.9819091
8 H	45.8532125	27.5144342	40.6236244
9 H	44.2268743	28.9344723	38.7123752
10 H	46.6883346	28.2408869	35.3675401
11 H	47.5964692	26.8288024	37.3014319
12 N	41.3509147	26.9739552	34.2349866
13 C	42.5607305	26.3203884	34.0902210
14 C	42.6704303	24.9039244	33.6303170
15 N	42.8943677	28.4429935	34.7510076
16 C	43.5097221	27.2671532	34.3990994
17 C	41.5928169	28.2436654	34.6220351
18 H	41.6649750	24.4797152	33.4867244
19 H	43.1528034	24.8729279	32.6441045
20 H	44.5906328	27.1638145	34.4082355
21 H	40.8147852	28.9752127	34.8212804
22 H	40.4178576	26.5924704	34.0847071
23 Fe	43.8347135	30.0736313	35.7345759
24 O	44.2146024	31.5049088	36.3801752
25 Cl	42.0183118	29.8577325	37.2078190
26 O	45.9188938	32.1855244	34.3595661
27 C	45.7485415	31.1198630	33.7883013
28 O	44.8435365	30.2205601	34.1244592
29 C	46.5468604	30.7493417	32.5474349
30 C	45.8076473	31.1468391	31.2679844
31 C	46.6408752	31.0113378	29.9839979
32 O	47.8665443	30.7983399	30.0827041
33 O	46.0064574	31.1364784	28.8938788
34 H	46.7576683	29.6694974	32.5421183
35 H	47.5077987	31.2785700	32.5846750
36 H	44.8875320	30.5543429	31.1384853
37 H	45.4841063	32.2025307	31.3245619
38 O	45.5430876	32.7806423	43.9960584
39 C	45.9615465	32.5023253	42.6787544
40 C	45.4387986	33.5223146	41.6548905
41 O	46.2727999	33.5368095	40.4720476
42 C	44.0409389	33.2235251	41.1044198
43 O	43.4804303	34.4447548	40.6489298
44 C	44.3650397	32.2649393	39.9479850
45 C	45.6815006	32.8622049	39.4095462
46 N	46.6333279	31.8763612	38.8468653

47 C	47.0581674	31.7972898	37.5425980
48 N	47.9896236	30.8869928	37.3368750
49 C	48.2051519	30.3305301	38.5836706
50 C	49.0250342	29.2805087	39.0587264
51 N	49.8988791	28.5743484	38.2801617
52 N	48.9733556	28.9756743	40.3668397
53 C	48.1263592	29.6381160	41.1677538
54 N	47.2807247	30.5981920	40.8217039
55 C	47.3622122	30.9309515	39.5372361
56 P	46.7107228	33.3993854	45.0443932
57 O	46.9238644	34.8564384	44.6074188
58 O	46.0080534	33.2307765	46.4224226
59 O	47.9502230	32.4852824	44.8999878
60 H	45.6254940	31.4866941	42.4171317
61 H	47.0578224	32.4899535	42.5994189
62 H	45.4815805	34.5234203	42.1017172
63 H	43.4072973	32.7521824	41.8818050
64 H	44.5407067	31.2523928	40.3406169
65 H	43.5833317	32.2007483	39.1754841
66 H	45.4809953	33.5536424	38.5737814
67 H	46.6066506	32.4080787	36.7616319
68 H	48.1282936	29.3479038	42.2247070
69 H	50.2229997	27.7048374	38.7116704
70 H	49.7542559	28.5404009	37.2746015
71 H	42.7225934	34.2724043	40.0734034
72 H	45.6166970	25.9450370	39.7900861
73 H	43.1704119	24.2222643	34.3183760

Off2-InTS1
QM(B1)/MM Energy = -4320.401812267024 a.u.
QM(B2)/MM Energy = -4323.420046000000 a.u.
QM(B3)/MM Energy = -4322.880229989578 a.u.

1 N	46.8207760	27.3990003	37.5296194
2 C	46.0492897	27.5722364	38.6645335
3 C	46.1799032	26.7728496	39.9220425
4 N	45.3032310	28.8897217	37.0057656
5 C	45.1104686	28.5122115	38.3138698
6 C	46.3506135	28.2137572	36.5580405
7 H	47.2247913	26.5004050	40.1289236
8 H	45.8404028	27.3803024	40.7746648
9 H	44.2908688	28.9132043	38.9049785
10 H	46.8007586	28.3161961	35.5729348
11 H	47.6436586	26.8055743	37.4594864
12 N	41.4882143	27.1538146	34.1440619
13 C	42.6474703	26.4013955	34.1787916
14 C	42.6987228	24.9890139	33.6884026
15 N	43.0517961	28.5006644	34.9047387
16 C	43.6084220	27.2722115	34.6459564
17 C	41.7759556	28.4021610	34.5783658
18 H	41.6750812	24.6025616	33.5613478
19 H	43.1576578	24.9498179	32.6890795
20 H	44.6598626	27.0698887	34.8312533
21 H	41.0299492	29.1862820	34.6738841
22 H	40.5570860	26.8437312	33.8734348
23 Fe	43.9572614	30.2546503	36.1244412
24 O	44.3178754	31.6244848	37.1520526
25 Cl	41.9271546	29.8327531	37.3201165
26 O	45.9815299	32.5055620	34.7205841
27 C	45.7145040	31.4942508	34.0800967
28 O	44.7496886	30.6661805	34.3998360
29 C	46.5130543	31.1170760	32.8296381
30 C	45.7692727	31.3239542	31.5056558
31 C	46.6305017	31.1471494	30.2343939
32 O	47.8543117	30.9304461	30.3453479
33 O	46.0105191	31.2394131	29.1315931
34 H	46.8236503	30.0638501	32.9147114
35 H	47.4283398	31.7241715	32.8224335
36 H	44.9140565	30.6328345	31.4219240
37 H	45.3405618	32.3413698	31.4514486
38 O	45.4096955	32.6976383	43.8274351
39 C	45.8028118	32.3404191	42.5235192
40 C	45.4684396	33.3994088	41.4513096
41 O	46.5309729	33.4807675	40.4648048
42 C	44.1954181	33.1559715	40.5934511
43 O	43.7644844	34.4189611	40.1300538
44 C	44.7991774	32.2581826	39.5152552
45 C	46.1622848	32.8993078	39.2605039
46 N	47.1710404	31.9348266	38.7961688
47 C	47.6201457	31.7929117	37.5085229
48 N	48.4560976	30.7867620	37.3463253
49 C	48.5581069	30.2191590	38.6028526
50 C	49.2092268	29.0665146	39.1038950
51 N	50.0100023	28.2627616	38.3534983
52 N	49.0453383	28.7582114	40.4026102
53 C	48.2606711	29.5274051	41.1709082
54 N	47.5737016	30.5995740	40.8002946
55 C	47.7545634	30.9183273	39.5216422
56 P	46.6045427	33.3328324	44.8496183
57 O	46.7906814	34.7922493	44.4082002
58 O	45.9417655	33.1662185	46.2477734
59 O	47.8489483	32.4306003	44.6789023
60 H	45.3286606	31.3721496	42.2726151
61 H	46.8860934	32.1734910	42.4756006
62 H	45.4050472	34.3821895	41.9382092
63 H	43.4095383	32.6417434	41.1778737
64 H	44.8801334	31.2154019	39.8529803
65 H	44.2878151	32.0506428	38.3223685
66 H	46.0928299	33.6553808	38.4574317
67 H	47.2446983	32.4292177	36.7074743
68 H	48.1694404	29.2319818	42.2222887
69 H	50.2512936	27.3713897	38.7932191
70 H	49.9501468	28.2828402	37.3390591
71 H	43.1634531	34.3251254	39.3757977
72 H	45.5967227	25.8536359	39.8671452
73 H	43.1847799	24.2721346	34.3501425

Off2-IM1
QM(B1)/MM Energy = -4320.463922881029 a.u.
QM(B2)/MM Energy = -4323.488235000000 a.u.
QM(B3)/MM Energy = -4322.939022982749 a.u.

1 N	46.7062094	27.4729690	37.2147111
2 C	45.9722311	27.6437691	38.3767147
3 C	46.1868982	26.9066832	39.6626425
4 N	45.1070531	28.8927971	36.7150256
5 C	44.9869108	28.5373825	38.0416214
6 C	46.1683222	28.2438345	36.2457485
7 H	47.2463561	26.6662463	39.8348753
8 H	45.8726373	27.5521790	40.4969096
9 H	44.1707415	28.9086039	38.6522723
10 H	46.5699761	28.3419148	35.2400658
11 H	47.5424708	26.9027028	37.1196875
12 N	41.2261716	26.8145010	34.3660544
13 C	42.4495578	26.2802876	33.9972780
14 C	42.6345504	24.8618466	33.5759846
15 N	42.6645846	28.4571597	34.4958838
16 C	43.3306874	27.3314337	34.0682766
17 C	41.3939683	28.1202651	34.6501361
18 H	41.6542827	24.3920599	33.4051552
19 H	43.1486880	24.8445841	32.6074581
20 H	44.3926091	27.3571954	33.8386672
21 H	40.6035521	28.7840530	34.9903563
22 H	40.3310695	26.3248781	34.4070385
23 Fe	43.6929654	29.9669118	35.5310292
24 O	42.9672906	31.6491598	35.4755529
25 Cl	42.0728006	29.5507057	37.3990911
26 O	45.9696097	31.8615413	34.8268311
27 C	45.8350149	31.0194911	33.9399360
28 O	44.9946376	30.0202307	34.0403465
29 C	46.6520690	31.0601747	32.6593728
30 C	45.8197929	31.1184123	31.3782988
31 C	46.6454511	31.0239684	30.0838565
32 O	47.8670408	30.7757934	30.1689214
33 O	46.0111258	31.1823115	28.9986681
34 H	47.2830017	30.1571800	32.6276518
35 H	47.3330674	31.9219708	32.7148353
36 H	45.0836000	30.2959516	31.3617882
37 H	45.2265132	32.0477659	31.3265749
38 O	46.4615023	32.8441930	43.8036549
39 C	46.8106461	33.2594217	42.4891981
40 C	45.5642859	33.6562327	41.6734070
41 O	45.9094363	33.7195535	40.2590172
42 C	44.3871511	32.6628266	41.7548878
43 O	43.0792696	33.2796631	41.7604935
44 C	44.5798496	31.8460895	40.5243868
45 C	45.4879002	32.5737765	39.5836300
46 N	46.6001992	31.7251266	39.0786020
47 C	47.0512467	31.6800783	37.7791014
48 N	48.0071470	30.7985634	37.5755443
49 C	48.2066124	30.2219330	38.8162230
50 C	48.9949158	29.1368873	39.2655944
51 N	49.8408090	28.4306235	38.4689820
52 N	48.9119791	28.7893530	40.5618698
53 C	48.0593654	29.4331791	41.3685489
54 N	47.2172925	30.4081178	41.0398267
55 C	47.3331944	30.7806535	39.7664258
56 P	47.0914194	33.7329816	45.1107243
57 O	46.9551583	35.1998941	44.6753940
58 O	46.1576755	33.3277029	46.2886243
59 O	48.5293364	33.2204983	45.2964590
60 H	47.3195660	32.4316555	41.9835526
61 H	47.4926385	34.1215512	42.5068046
62 H	45.2339867	34.6616053	41.9712849
63 H	44.4437957	32.0936118	42.6936227
64 H	44.0155477	30.9510991	40.2599045
65 H	42.7620158	31.9802817	36.3653727
66 H	44.9687517	32.8766491	38.6512353
67 H	46.5936886	32.2814942	36.9941751
68 H	48.0478953	29.1111545	42.4143039
69 H	50.1689483	27.5474637	38.8669997
70 H	49.7606062	28.4809515	37.4580050
71 H	42.9000954	33.6084542	40.8645576
72 H	45.6280933	25.9717248	39.7033474
73 H	43.1435659	24.2116323	34.2874561

Off2-TS2
QM(B1)/MM Energy = -4320.444295449830 a.u.
QM(B2)/MM Energy = -4323.462273000000 a.u.
QM(B3)/MM Energy = -4322.919670525368 a.u.

1 N	46.9419930	27.2275036	37.4958905
2 C	46.1576508	27.4448032	38.6147644
3 C	46.2042244	26.6523342	39.8821183
4 N	45.5747159	28.8701434	36.9705207
5 C	45.3122492	28.4662045	38.2587301
6 C	46.5654985	28.1045829	36.5339937
7 H	47.2291240	26.3456665	40.1372995
8 H	45.8538345	27.2852375	40.7114412
9 H	44.5231732	28.9304520	38.8422815
10 H	47.0194412	28.1727189	35.5476811
11 H	47.6958430	26.5479328	37.4200186
12 N	41.4770304	27.2466587	34.4363339
13 C	42.6220511	26.4700651	34.4050969
14 C	42.6699377	25.1110238	33.7721233
15 N	43.0629061	28.4812543	35.3345508
16 C	43.5931354	27.2672834	34.9690086
17 C	41.7873305	28.4474870	34.9866897
18 H	41.6461132	24.7418252	33.6022446
19 H	43.1395943	25.1575467	32.7760320
20 H	44.6426597	27.0378461	35.1232189
21 H	41.0692988	29.2562717	35.0938594

22 H	40.5435694	27.0030459	34.1041792
23 Fe	44.3335740	30.2554406	35.8151898
24 O	43.1389743	31.4232142	34.9551204
25 Cl	43.4870643	31.1574805	38.1766785
26 O	45.9663318	31.5983229	34.9519917
27 C	46.1077749	30.7462746	34.0113375
28 O	45.5507069	29.6162450	34.0964481
29 C	46.8916945	31.0819819	32.7612758
30 C	46.0146376	31.0544862	31.5049886
31 C	46.7973841	31.0343706	30.1823747
32 O	48.0269946	30.8180429	30.2209027
33 O	46.1234445	31.2008052	29.1222447
34 H	47.6849259	30.3297938	32.6388031
35 H	47.3765619	32.0634158	32.8681116
36 H	45.3806390	30.1513129	31.5214203
37 H	45.3159219	31.9074292	31.4820838
38 O	46.2845616	32.8187483	43.7253546
39 C	46.7588976	33.1421694	42.4252031
40 C	45.6428567	33.6061950	41.4689932
41 O	46.1980018	33.7186978	40.1311310
42 C	44.3997160	32.6862544	41.2847573
43 O	43.1963577	33.4068582	41.0432786
44 C	44.8509188	31.8160674	40.1268683
45 C	45.9101784	32.5880062	39.3703811
46 N	47.0613130	31.7340824	39.0188048
47 C	47.5917779	31.6577233	37.7531733
48 N	48.4878188	30.7123987	37.6005641
49 C	48.5498241	30.1042904	38.8407359
50 C	49.1907566	28.9331856	39.3085692
51 N	50.0046913	28.1677041	38.5432988
52 N	48.9889250	28.5720324	40.5894912
53 C	48.1503762	29.2802135	41.3526361
54 N	47.4252963	30.3367330	40.9917426
55 C	47.6638060	30.7219928	39.7392907
56 P	46.9657857	33.6947674	45.0266661
57 O	46.8373130	35.1654149	44.6036327
58 O	46.0593139	33.2952610	46.2264286
59 O	48.4018231	33.1626834	45.1706498
60 H	47.2442968	32.2547324	42.0026559
61 H	47.5019370	33.9543556	42.4542413
62 H	45.2970605	34.6061973	41.7705056
63 H	44.2342968	32.0962877	42.1976353
64 H	44.9459478	30.7442902	40.2716810
65 H	43.6481410	32.2303072	34.8056354
66 H	45.5339240	32.9145452	38.3884977
67 H	47.2108053	32.2895527	36.9566673
68 H	48.0386689	28.9450475	42.3883777
69 H	50.2456459	27.2594644	38.9457215
70 H	49.9843201	28.2469206	37.5300587
71 H	43.1635805	33.6818298	40.1136831
72 H	45.5910445	25.7531109	39.8231696
73 H	43.1506426	24.3339691	34.3664094

Off2-2'-Cl-dAMP
QM(B1)/MM Energy = -4320.504505405672 a.u.
QM(B2)/MM Energy = -4323.527680000000 a.u.
QM(B3)/MM Energy = -4322.969688922935 a.u.

1 N	46.7661507	27.4490563	37.2337686
2 C	45.9794561	27.6963434	38.3420170
3 C	46.1172590	26.9868099	39.6540478
4 N	45.3018948	28.9685116	36.6047578
5 C	45.0732855	28.6462649	37.9243925
6 C	46.3333069	28.2363256	36.2172834
7 H	47.1671001	26.7474309	39.8823471
8 H	45.7681469	27.6475703	40.4622577
9 H	44.2837736	29.1263997	38.4985595
10 H	46.8043443	28.2727409	35.2366267
11 H	47.5653279	26.8219410	37.2027406
12 N	41.2658073	27.0038002	34.3512394
13 C	42.4672485	26.3475230	34.1736364
14 C	42.5816841	24.9476968	33.6602333
15 N	42.8311317	28.4598706	34.8733074
16 C	43.4244672	27.2799906	34.5038930
17 C	41.5288062	28.2725171	34.7598902
18 H	41.5788790	24.5180621	33.5132648
19 H	43.0561878	24.9489021	32.6681092
20 H	44.5046321	27.1609880	34.5027279
21 H	40.7576569	29.0201730	34.9387360
22 H	40.3267201	26.6450443	34.1778547
23 Fe	43.9593908	30.2168003	35.4691145
24 O	42.6253026	31.2036526	36.3889615
25 Cl	43.4570299	31.5593648	39.5647680
26 O	45.8014501	32.0557852	34.8290763
27 C	45.6645848	31.4183221	33.7622483
28 O	44.7959980	30.4794104	33.6484057
29 C	46.5414664	31.6870203	32.5484015
30 C	45.8040809	31.5162562	31.2249786
31 C	46.6687700	31.2705636	29.9857461
32 O	47.8871652	31.0337546	30.1201373
33 O	46.0479890	31.2782983	28.8784570
34 H	47.3727037	30.9603468	32.5799396
35 H	46.9951911	32.6859245	32.6412889
36 H	45.1378241	30.6414274	31.3044514
37 H	45.1467141	32.3765369	31.0162836
38 O	46.4704049	32.9330769	43.8104533
39 C	46.9973390	33.2958123	42.5423323
40 C	45.9552524	33.9383478	41.6090031
41 O	46.4659396	33.8821117	40.2493005
42 C	44.5812046	33.2606611	41.5088163
43 O	43.6686670	34.2026278	40.9860001
44 C	44.8906390	32.1587397	40.4897909
45 C	45.9027242	32.8315873	39.5382012
46 N	46.8926397	31.8702505	39.0167271
47 C	47.2932814	31.7686698	37.7022487
48 N	48.1805091	30.8239660	37.4858058

49 C	48.3779408	30.2472343	38.7263044
50 C	49.1065009	29.1155407	39.1686064
51 N	49.9026064	28.3623012	38.3716981
52 N	49.0150995	28.7727186	40.4674717
53 C	48.2279719	29.4744735	41.2867759
54 N	47.4660242	30.5172850	40.9721419
55 C	47.5775959	30.8784180	39.6932856
56 P	47.0738460	33.8085252	45.1409611
57 O	46.9238664	35.2782487	44.7209414
58 O	46.1296246	33.3715840	46.2968538
59 O	48.5176053	33.3099308	45.3235344
60 H	47.3848025	32.3892791	42.0638774
61 H	47.8332099	34.0057882	42.6351132
62 H	45.8064763	34.9960242	41.8655356
63 H	44.2626680	32.8580616	42.4853868
64 H	45.3296853	31.2881507	40.9887607
65 H	43.0350867	31.9760581	36.8031484
66 H	45.3897677	33.2165161	38.6413348
67 H	46.8421698	32.3821222	36.9238713
68 H	48.1991983	29.1498294	42.3319483
69 H	50.2015617	27.4733717	38.7799703
70 H	49.8302500	28.4158967	37.3593562
71 H	42.9219217	33.7394103	40.5770661
72 H	45.5607642	26.0501285	39.6858084
73 H	43.0966513	24.2491927	34.3197542

Off2-TS2'
QM(B1)/MM Energy = -4320.425094109016 a.u.
QM(B2)/MM Energy = -4323.443636000000 a.u.
QM(B3)/MM Energy = -4322.900318052007 a.u.

1 N	46.8172529	27.3830110	37.5836540
2 C	46.0291524	27.5170508	38.7118161
3 C	46.1403042	26.6797296	39.9467014
4 N	45.3246218	28.9160988	37.0962822
5 C	45.1061385	28.4808348	38.3828492
6 C	46.3700501	28.2434828	36.6388246
7 H	47.1810839	26.3950154	40.1577495
8 H	45.7951512	27.2667926	40.8112751
9 H	44.2743384	28.8557032	38.9740280
10 H	46.8339058	28.3748756	35.6633282
11 H	47.6365546	26.7859562	37.5017553
12 N	41.5380376	27.1877232	34.0689660
13 C	42.6674784	26.4010293	34.2017715
14 C	42.7084444	24.9934654	33.6954290
15 N	43.0695505	28.4914902	34.9564339
16 C	43.6100806	27.2446471	34.7501102
17 C	41.8234462	28.4277986	34.5255699
18 H	41.6808309	24.6180562	33.5641082
19 H	43.1666832	24.9615837	32.6948889
20 H	44.6353291	27.0129487	35.0270734
21 H	41.0916071	29.2291004	34.5685126
22 H	40.6235152	26.8979396	33.7307563
23 Fe	43.9031908	30.2174918	36.2225219
24 O	43.9547992	31.6152750	37.5802988
25 Cl	41.7356934	29.7568931	37.2999424
26 O	45.9977234	32.4514771	35.0671004
27 C	45.6673501	31.5820706	34.2523766
28 O	44.6920635	30.7540645	34.4680980
29 C	46.4716228	31.4059736	32.9530745
30 C	45.7062028	31.3333822	31.6282329
31 C	46.5882854	31.1884262	30.3592990
32 O	47.8100191	30.9594619	30.4781859
33 O	45.9854372	31.2960764	29.2480663
34 H	47.0632891	30.4814630	33.0681072
35 H	47.2004528	32.2283016	32.9067735
36 H	45.0052425	30.4800522	31.6357757
37 H	45.0836038	32.2322484	31.4758418
38 O	46.2594669	32.8524060	43.4730563
39 C	46.7873699	33.1803401	42.1915478
40 C	45.7271119	33.6283203	41.1604664
41 O	46.3816796	33.7763532	39.8686163
42 C	44.5119393	32.7120390	40.8443950
43 O	43.3304779	33.4486453	40.5193026
44 C	45.0293005	31.8991900	39.6791703
45 C	46.1353462	32.6832266	39.0352877
46 N	47.2971909	31.8143376	38.7403674
47 C	47.8762796	31.6972908	37.4996733
48 N	48.7211712	30.6949156	37.3973413
49 C	48.6919530	30.0956670	38.6432367
50 C	49.2442171	28.8973468	39.1549546
51 N	50.0422045	28.0744238	38.4341024
52 N	48.9626885	28.5627042	40.4276783
53 C	48.1284684	29.3270968	41.1420213
54 N	47.4857151	30.4185563	40.7362611
55 C	47.8050939	30.7756486	39.4934452
56 P	46.9318429	33.6932801	44.8062614
57 O	46.7792218	35.1752550	44.4323720
58 O	46.0391147	33.2532826	46.0040571
59 O	48.3741731	33.1736655	44.9379908
60 H	47.3145121	32.3051542	41.7956800
61 H	47.5090653	34.0095369	42.2543047
62 H	45.3443532	34.6191684	41.4504055
63 H	44.2535352	32.0833043	41.7080257
64 H	45.0223896	30.8147026	39.6810149
65 H	43.0829931	31.6515728	38.0089253
66 H	45.8351912	33.0434558	38.0395084
67 H	47.5632729	32.3377425	36.6774386
68 H	47.9420887	29.0092667	42.1721985
69 H	50.2458801	27.1712112	38.8671920
70 H	50.0841083	28.1488323	37.4214094
71 H	43.4773173	33.9390739	39.6931234
72 H	45.5503715	25.7668161	39.8652418
73 H	43.1863850	24.2614401	34.3464120

Off2-2'-OH-dAMP

QM(B1)/MM Energy = -4320.542199768865 a.u.
QM(B2)/MM Energy = -4323.555628000000 a.u.
QM(B3)/MM Energy = -4322.994808151024 a.u.

1	N	46.9020719	27.3115059	37.2506841
2	C	46.0593494	27.5817703	38.3104250
3	C	46.1307375	26.9048464	39.6440216
4	N	45.4954858	28.8400417	36.5242462
5	C	45.1871121	28.5333257	37.8324348
6	C	46.5379134	28.0894017	36.2030725
7	H	47.1680099	26.6602541	39.9171143
8	H	45.7613592	27.5925373	40.4198089
9	H	44.3566377	29.0156398	38.3427488
10	H	47.0670751	28.1054274	35.2523500
11	H	47.6908140	26.6683088	37.2683866
12	N	41.3618578	27.0766328	34.3501404
13	C	42.5254045	26.3599454	34.1332361
14	C	42.5622211	24.9439029	33.6545406
15	N	43.0128879	28.4660691	34.7668315
16	C	43.5365174	27.2544501	34.3959626
17	C	41.6997110	28.3383812	34.7193398
18	H	41.5391005	24.5500889	33.5563133
19	H	42.9979037	24.9035260	32.6460005
20	H	44.6098582	27.0863541	34.3542233
21	H	40.9766595	29.1227609	34.9375694
22	H	40.3998423	26.7610386	34.2162997
23	Fe	44.2247609	30.1408517	35.3862939
24	O	43.2327463	32.7022752	39.5951847
25	Cl	42.7408913	30.9469591	37.0052821
26	O	45.8663558	31.8406927	34.8015190
27	C	45.5800293	31.4602671	33.6414735
28	O	44.5728275	30.6822820	33.4615815
29	C	46.4441514	31.8145297	32.4496741
30	C	45.7408189	31.6672058	31.1094023
31	C	46.6245320	31.3406381	29.9033657
32	O	47.8331200	31.0798243	30.0735028
33	O	46.0195222	31.3096470	28.7884502
34	H	47.3047122	31.1208637	32.4735860
35	H	46.8684067	32.8195590	32.6006268
36	H	45.0260430	30.8321561	31.1754740
37	H	45.1398807	32.5578860	30.8652202
38	O	46.4678531	32.8766261	43.8455045
39	C	46.8708414	33.2966441	42.5480529
40	C	45.6615562	33.6799927	41.6793870
41	O	46.1235814	33.8388135	40.3130129
42	C	44.4902099	32.6650293	41.6358516
43	O	43.2382093	33.2132956	42.0177153
44	C	44.4230843	32.1909374	40.1457056
45	C	45.6401767	32.8621824	39.4656057
46	N	46.6743846	31.8956318	39.0125079
47	C	47.1621813	31.8265838	37.7303079
48	N	48.0799492	30.9021653	37.5459881
49	C	48.2080237	30.3105618	38.7876814
50	C	48.9507613	29.2031079	39.2582699
51	N	49.8163184	28.4934103	38.4864947
52	N	48.8071180	28.8462204	40.5466103
53	C	47.9390824	29.5090758	41.3236053
54	N	47.1473206	30.5198087	40.9758624
55	C	47.3291901	30.9048834	39.7126887
56	P	47.0895298	33.7512289	45.1625133
57	O	46.9603453	35.2236709	44.7446573
58	O	46.1516871	33.3368866	46.3342425
59	O	48.5280222	33.2352663	45.3467040
60	H	47.4177460	32.4811809	42.0607927
61	H	47.5371162	34.1698128	42.6001735
62	H	45.2729727	34.6543613	42.0114489
63	H	44.7194378	31.8207791	42.3013847
64	H	44.4640367	31.0925139	40.0663691
65	H	43.0395419	32.2614250	38.7483901
66	H	45.2938411	33.3298323	38.5303311
67	H	46.7516033	32.4578694	36.9461170
68	H	47.8690095	29.1716883	42.3618134
69	H	50.1270302	27.6051315	38.8863226
70	H	49.7741302	28.5561927	37.4740342
71	H	42.7546725	33.3317871	41.1747178
72	H	45.5612409	25.9759388	39.6735399
73	H	43.0768004	24.2415179	34.3102315

In1-RC
QM(B1)/MM Energy = -4319.579626755539 a.u.
QM(B2)/MM Energy = -4322.598390000000 a.u.
QM(B3)/MM Energy = -4322.024595550384 a.u.

1	N	30.6319064	34.9432718	40.1938569
2	C	30.1357088	35.0615739	38.9092854
3	C	28.6885231	35.0251636	38.5536687
4	N	32.3773962	34.9797981	38.8833010
5	C	31.2519704	35.0949458	38.1052086
6	C	31.9809661	34.8941185	40.1437067
7	H	28.0726201	35.4452280	39.3572588
8	H	28.4744446	35.6855441	37.7040593
9	H	31.3139734	35.2118830	37.0275190
10	H	32.6320996	34.7807676	41.0063988
11	H	30.0829729	34.8882372	41.0549145
12	N	32.8001473	30.8447538	36.9539208
13	C	33.1208462	30.5839067	38.2722407
14	C	32.8279735	29.3110967	39.0015245
15	N	33.6836135	32.6932159	37.7441525
16	C	33.6768834	31.7458612	38.7419644
17	C	33.1509862	32.1171824	36.6735181
18	H	32.7392257	28.4605512	38.3112646
19	H	33.6592766	29.0736452	39.6802138
20	H	34.0722373	31.9658652	39.7274296
21	H	33.0432901	32.5891925	35.6990333
22	H	32.4018709	30.1696722	36.2879151
23	Fe	34.2667704	34.6820082	38.1537292

24 O	34.7740337	36.1799112	38.4869820
25 Cl	34.3755768	35.0205969	35.8555905
26 O	36.4790297	34.4274246	41.3030033
27 C	36.3804990	34.0775020	40.1369093
28 O	35.2133875	33.8686338	39.5728347
29 C	37.5995401	33.8190737	39.2718727
30 C	38.3353616	35.1282911	38.9340542
31 C	38.9513851	35.7394924	40.2136485
32 O	38.4646519	36.8064709	40.6453793
33 O	39.8793098	35.0618441	40.7310590
34 H	37.2936259	33.2837623	38.3591848
35 H	38.2997889	33.1922159	39.8487169
36 H	39.1432367	34.8751653	38.2307721
37 H	37.6510140	35.8446685	38.4535429
38 O	29.6459064	38.0864668	38.2376950
39 C	30.9297624	38.5794754	38.0250007
40 C	31.1750240	39.9251305	38.7034165
41 O	30.8433520	39.8349456	40.1205354
42 C	32.6420907	40.3714283	38.6598297
43 O	32.8181064	41.7638141	38.4869989
44 C	33.1794945	39.8920272	40.0153874
45 C	31.9680620	40.1497885	40.9067628
46 N	31.9124948	39.3717158	42.1417318
47 C	32.3484034	38.0997550	42.3802264
48 N	32.0643849	37.6549980	43.5902888
49 C	31.3748292	38.6980450	44.1877555
50 C	30.7625286	38.8867580	45.4525303
51 N	30.7901999	37.9406688	46.4323242
52 N	30.1042980	40.0402573	45.6740266
53 C	30.0607492	40.9760095	44.7085990
54 N	30.6457067	40.9318069	43.5128675
55 C	31.2602152	39.7712232	43.2907504
56 P	28.3529525	38.6727625	37.2716280
57 O	27.2771345	37.6260361	37.5580765
58 O	28.0447788	40.0865740	37.8069500
59 O	28.9191529	38.6358427	35.8279612
60 H	31.1377151	38.7076289	36.9461249
61 H	31.6566131	37.8410140	38.4170189
62 H	30.5111624	40.6771397	38.2496959
63 H	33.1795531	39.8857704	37.8306055
64 H	33.4133870	38.8179775	39.9576039
65 H	34.0828513	40.4342443	40.3188899
66 H	31.9258188	41.2044216	41.2334569
67 H	32.8825644	37.5326512	41.6191277
68 H	29.4828351	41.8821155	44.9309538
69 H	31.5858653	37.2941909	46.4728076
70 H	30.2981873	38.1841799	47.2968413
71 H	32.1055845	42.3055972	38.8994184
72 H	28.3329160	34.0069078	38.3962969
73 H	31.8963671	29.3421953	39.5665108

In1-TS1

QM(B1)/MM Energy = -4319.541250340883 a.u.

QM(B2)/MM Energy = -4322.555045000000 a.u.

QM(B3)/MM Energy = -4321.973359518810 a.u.

1 N	30.2490631	34.9353352	40.2382298
2 C	29.7946158	35.0843370	38.9419356
3 C	28.3727157	34.9647419	38.5146483
4 N	32.0334896	35.1509152	39.0046557
5 C	30.9339380	35.2232953	38.1875309
6 C	31.5966134	34.9746307	40.2400826
7 H	27.6891047	35.3395380	39.2847989
8 H	28.1749319	35.6135078	37.6530014
9 H	31.0191737	35.3977145	37.1191477
10 H	32.2138679	34.8651319	41.1264822
11 H	29.6912778	34.8012661	41.0817793
12 N	32.8883180	31.2413551	37.0442529
13 C	33.2024436	30.9413698	38.3547069
14 C	32.9758634	29.5956120	38.9771860
15 N	33.6841045	33.1058260	37.9201227
16 C	33.6977159	32.1171104	38.8785528
17 C	33.1924344	32.5436998	36.8257015
18 H	32.9713281	28.8013730	38.2167953
19 H	33.7978020	29.3547610	39.6684976
20 H	34.0624783	32.2987362	39.8877858
21 H	33.0764164	33.0382635	35.8626839
22 H	32.5090883	30.5738633	36.3615559
23 Fe	33.9725619	35.4444743	38.3929349
24 O	33.9000192	37.1692644	38.7835782
25 Cl	34.6060429	35.5327081	36.1604231
26 O	36.0434089	33.7065511	41.3637612
27 C	36.0363926	34.1109034	40.2074082
28 O	35.0920390	34.9248046	39.8059062
29 C	37.1009200	33.7286070	39.1938070
30 C	38.0355095	34.9053840	38.8443264
31 C	38.7049557	35.4410400	40.1283760
32 O	38.1617096	36.4350117	40.6703467
33 O	39.6922800	34.7946331	40.5601305
34 H	36.6007281	33.3630963	38.2810426
35 H	37.6945489	32.9145215	39.6347656
36 H	38.8007713	34.5360871	38.1438075
37 H	37.4599252	35.7049462	38.3533945
38 O	29.5713964	38.0904531	38.1063033
39 C	30.8630562	38.5057386	37.8013555
40 C	31.2419468	39.8195271	38.4824280
41 O	31.0150036	39.7150007	39.9172472
42 C	32.7288091	40.1578270	38.3446658
43 O	33.0054283	41.5484628	38.3704779
44 C	33.3284648	39.5158857	39.5977141
45 C	32.2359598	39.8375476	40.6031718
46 N	32.1644146	39.0864761	41.8436341
47 C	32.5315711	37.7970687	42.1067976
48 N	32.1861113	37.3905008	43.3132520
49 C	31.5333253	38.4712468	43.8807088
50 C	30.9096748	38.7165666	45.1317963

51 N	30.8654469	37.7981838	46.1414273
52 N	30.3056246	39.9052098	45.3163897
53 C	30.3300113	40.8251816	44.3373108
54 N	30.9431501	40.7322442	43.1576420
55 C	31.5017543	39.5366346	42.9683457
56 P	28.2623771	38.7208438	37.1986610
57 O	27.1636017	37.7091792	37.5214302
58 O	28.0279658	40.1391224	37.7598446
59 O	28.7784236	38.6818864	35.7357662
60 H	30.9943270	38.6436523	36.7131844
61 H	31.5712260	37.7269619	38.1263798
62 H	30.5981938	40.6268764	38.0992355
63 H	33.1588767	39.7450620	37.4199780
64 H	33.5143272	38.2591734	39.2893789
65 H	34.3386551	39.8521684	39.8634300
66 H	32.3861579	40.8887994	40.9207384
67 H	33.0686127	37.2082673	41.3620331
68 H	29.7903869	41.7603250	44.5316334
69 H	31.6545549	37.1497881	46.2412615
70 H	30.3902703	38.1139252	46.9922169
71 H	32.2658359	42.0961385	38.7333093
72 H	28.1004084	33.9221852	38.3503575
73 H	32.0303753	29.5014261	39.5112725

In1-IM1
QM(B1)/MM Energy = -4319.572886609418 a.u.
QM(B2)/MM Energy = -4322.591840000000 a.u.
QM(B3)/MM Energy = -4322.008255577630 a.u.

1 N	30.5074996	34.9158430	40.1744045
2 C	29.9673473	35.1313296	38.9193411
3 C	28.5218831	35.0353102	38.5741681
4 N	32.2077346	35.1916324	38.8316033
5 C	31.0535489	35.3159085	38.0994419
6 C	31.8548469	34.9515929	40.0859751
7 H	27.8885436	35.4117497	39.3863221
8 H	28.2810091	35.6979461	37.7334325
9 H	31.0639184	35.5631588	37.0419192
10 H	32.5388534	34.7874431	40.9156474
11 H	29.9915646	34.7631775	41.0434826
12 N	32.8964088	31.0656309	36.9486167
13 C	33.1851445	30.8533158	38.2822212
14 C	32.9532649	29.5506514	38.9884272
15 N	33.6834854	32.9790112	37.7090415
16 C	33.6749458	32.0592282	38.7350701
17 C	33.2098553	32.3459338	36.6455715
18 H	32.9382621	28.7120954	38.2771190
19 H	33.7792811	29.3446169	39.6856038
20 H	34.0172180	32.3099328	39.7370927
21 H	33.1166540	32.7783240	35.6507496
22 H	32.5281969	30.3578353	36.2999174
23 Fe	34.1000807	35.2462513	37.9725603
24 O	34.1138455	37.1137050	38.0742929
25 Cl	34.6674660	35.2296773	35.7214347
26 O	35.7218289	33.7242691	41.2576908
27 C	35.9773122	34.0513394	40.1013475
28 O	35.1904448	34.8585016	39.4473580
29 C	37.1913117	33.5470934	39.3376596
30 C	38.1490233	34.6800534	38.9198939
31 C	38.6986024	35.3654123	40.1880494
32 O	38.0161237	36.3113362	40.6511550
33 O	39.7365964	34.8644742	40.6896274
34 H	36.8175720	33.0169706	38.4434007
35 H	37.7266526	32.8321428	39.9794958
36 H	38.9766656	34.2477135	38.3370728
37 H	37.6093533	35.4064024	38.2934587
38 O	29.5121898	38.1693115	38.2782341
39 C	30.7941781	38.6704413	38.0897307
40 C	31.0251565	40.0110310	38.7740310
41 O	30.6554653	39.9196561	40.1846485
42 C	32.4970876	40.4579664	38.7441202
43 O	32.6761096	41.8131549	38.3459526
44 C	32.9368983	40.2555807	40.1591545
45 C	31.7162463	40.3300643	41.0098806
46 N	31.7456923	39.5169667	42.2263476
47 C	32.2457316	38.2591939	42.4187689
48 N	32.0168024	37.7765191	43.6255995
49 C	31.3056417	38.7778326	44.2706654
50 C	30.7197388	38.9147842	45.5556011
51 N	30.7914615	37.9455199	46.5071685
52 N	30.0389847	40.0460851	45.8266358
53 C	29.9359049	41.0027566	44.8865323
54 N	30.4764645	40.9999484	43.6705072
55 C	31.1207375	39.8662562	43.4051346
56 P	28.2409447	38.7296051	37.2705212
57 O	27.1652373	37.6817662	37.5519407
58 O	27.9123295	40.1504755	37.7766482
59 O	28.8439760	38.6747256	35.8425811
60 H	31.0234836	38.8058872	37.0160450
61 H	31.5253641	37.9384908	38.4815701
62 H	30.3769036	40.7678228	38.3067346
63 H	33.0860479	39.8572552	38.0315415
64 H	33.7312844	37.4694402	38.8888757
65 H	33.9550918	40.4114240	40.5187262
66 H	31.5310891	41.3605295	41.3851227
67 H	32.7794939	37.7374409	41.6241718
68 H	29.3457931	41.8898801	45.1498364
69 H	31.5877569	37.2988075	46.5089955
70 H	30.3067398	38.1529842	47.3850826
71 H	32.0531061	42.4142513	38.8223886
72 H	28.2186972	34.0023006	38.4037664
73 H	32.0106655	29.4947251	39.5328957

In1-TS2
QM(B1)/MM Energy = -4319.490826914429 a.u.
QM(B2)/MM Energy = -4322.500954000000 a.u.

QM(B3)/MM Energy = -4321.917946258294 a.u.

1 N	30.4703195	34.7324878	40.4604448
2 C	30.0810860	34.9740085	39.1519919
3 C	28.6692708	35.0086394	38.6590218
4 N	32.3274530	34.9579476	39.3274834
5 C	31.2600047	35.1044900	38.4641713
6 C	31.8141652	34.7150276	40.5214873
7 H	27.9917036	35.4331316	39.4105087
8 H	28.5807234	35.6943734	37.8074465
9 H	31.4038087	35.3394564	37.4152768
10 H	32.3877242	34.5488132	41.4255621
11 H	29.8736424	34.6056621	41.2785915
12 N	32.7433785	31.3009549	36.8468877
13 C	33.2004430	30.8995816	38.0871364
14 C	32.9402664	29.5730898	38.7376625
15 N	33.8830691	33.0197012	37.6626422
16 C	33.9073962	31.9731488	38.5660528
17 C	33.1653495	32.5723070	36.6357198
18 H	32.8479223	28.7706813	37.9904989
19 H	33.8007677	29.3054348	39.3700975
20 H	34.3972741	32.0753307	39.5252731
21 H	32.9626833	33.1225541	35.7181891
22 H	32.2375809	30.6995575	36.1846905
23 Fe	34.3320915	35.1839673	38.7382777
24 O	35.5543240	36.4488138	39.2696144
25 Cl	33.5606968	36.9168728	37.3143941
26 O	36.4551552	33.9407941	41.8022346
27 C	36.2355843	33.7546095	40.6122297
28 O	35.0644009	33.9412946	40.0812006
29 C	37.3603282	33.2888137	39.6867940
30 C	38.1317738	34.4787472	39.0696734
31 C	38.6613421	35.3855947	40.1945158
32 O	37.8934045	36.3226262	40.5714167
33 O	39.7681558	35.0914054	40.6893577
34 H	36.9506851	32.6484114	38.8881026
35 H	38.0682635	32.7064102	40.2970046
36 H	38.9788527	34.0917528	38.4839845
37 H	37.4745081	35.0544254	38.4005101
38 O	29.2432980	38.1520245	38.2292545
39 C	30.5628666	38.3177983	37.8279497
40 C	31.1571172	39.6809180	38.1653402
41 O	31.1624261	39.8826358	39.6056155
42 C	32.6391465	39.7544851	37.8134042
43 O	33.1565303	41.0772965	37.9304202
44 C	33.2169581	38.9334996	38.9486927
45 C	32.4348152	39.5625860	40.1121223
46 N	32.2649214	38.8370277	41.3800866
47 C	32.6324063	37.5694982	41.7138805
48 N	32.2670502	37.2127369	42.9323700
49 C	31.6245211	38.3209545	43.4466318
50 C	31.0337240	38.6273202	44.7007701
51 N	30.9652545	37.7499155	45.7500841
52 N	30.4811161	39.8433696	44.8557849
53 C	30.5072793	40.7222281	43.8368457
54 N	31.0820345	40.5659868	42.6460792
55 C	31.6125120	39.3499535	42.4872377
56 P	28.0150896	38.8769926	37.2802118
57 O	26.8332814	37.9575439	37.5704015
58 O	27.8972182	40.3138100	37.8362940
59 O	28.5882066	38.7975941	35.8361264
60 H	30.6748508	38.1572401	36.7401437
61 H	31.1652711	37.5581171	38.3423022
62 H	30.5585652	40.4926282	37.7251356
63 H	32.8640978	39.3537814	36.8180690
64 H	36.3809918	36.3535345	39.8277389
65 H	34.2749821	38.7115325	39.0751068
66 H	32.9614938	40.4985029	40.3934704
67 H	33.2078077	36.9628434	41.0183453
68 H	30.0075077	41.6823773	44.0119121
69 H	31.7410855	37.0951795	45.8933398
70 H	30.5268583	38.1306152	46.5927518
71 H	32.4458516	41.7599052	38.0889296
72 H	28.2907222	34.0111335	38.4359745
73 H	32.0402072	29.5101228	39.3492144

In1-2'-Cl-dAMP

QM(B1)/MM Energy = -4319.648863043029 a.u.
QM(B2)/MM Energy = -4322.680115000000 a.u.
QM(B3)/MM Energy = -4322.093387847307 a.u.

1 N	30.8436021	35.0181525	39.9865417
2 C	30.2440446	35.1936689	38.7534173
3 C	28.7762776	35.1554804	38.4725209
4 N	32.4882356	35.2045296	38.5378530
5 C	31.2935313	35.3165424	37.8738426
6 C	32.1905055	35.0281677	39.8172849
7 H	28.1836986	35.5898739	39.2890392
8 H	28.5672110	35.7873319	37.6011522
9 H	31.2568025	35.4939352	36.8045681
10 H	32.9244086	34.8775724	40.6097667
11 H	30.3570233	34.9033056	40.8787534
12 N	33.0322910	30.7401428	36.8193751
13 C	33.2728308	30.6367022	38.1747128
14 C	32.9689251	29.4190196	38.9913733
15 N	33.8534343	32.6851508	37.4387135
16 C	33.7911136	31.8595833	38.5392044
17 C	33.3936816	31.9797346	36.4165482
18 H	32.9174327	28.5221205	38.3581385
19 H	33.7783026	29.2355702	39.7130168
20 H	34.1128908	32.1933448	39.5251298
21 H	33.3360431	32.3227648	35.3843826
22 H	32.6569711	30.0043557	36.2071047
23 Fe	34.3251699	34.7732504	37.5542039
24 O	34.8608385	35.2652496	35.8020376
25 Cl	34.2033923	39.2406238	40.5963327

26 O	35.4557475	33.9584841	41.0948176
27 C	35.9446325	34.2561889	39.9931592
28 O	35.3848993	35.0706612	39.1696879
29 C	37.2483189	33.6228968	39.5068096
30 C	38.2891523	34.6614977	39.0529836
31 C	38.8191721	35.4108217	40.2903860
32 O	38.1285447	36.3673258	40.7196487
33 O	39.8639513	34.9454411	40.8141347
34 H	36.9772358	32.9654489	38.6597757
35 H	37.6674644	32.9944233	40.3071753
36 H	39.1261787	34.1537111	38.5505151
37 H	37.8104616	35.3575518	38.3470209
38 O	29.0218574	38.4973460	37.9929950
39 C	30.3868711	38.7829999	37.8562142
40 C	30.8624848	39.7911696	38.8788984
41 O	30.7580885	39.2224481	40.1893578
42 C	32.3469271	40.1819778	38.7507715
43 O	32.5760429	41.2768203	37.9215929
44 C	32.8162264	40.3537687	40.2280262
45 C	31.5089176	40.0567273	41.0181837
46 N	31.6029972	39.4396273	42.3219831
47 C	32.1076881	38.2034739	42.6376754
48 N	31.9540168	37.8758283	43.9039954
49 C	31.2761913	38.9512434	44.4595191
50 C	30.8199025	39.2595056	45.7630817
51 N	31.1618132	38.5174848	46.8531309
52 N	30.0814935	40.3758497	45.9323261
53 C	29.8485109	41.1790162	44.8762808
54 N	30.3210997	41.0440405	43.6383366
55 C	31.0140246	39.9193368	43.4784007
56 P	27.9489307	39.4912576	37.1225347
57 O	26.5926433	38.8159526	37.4462377
58 O	28.0758236	40.9148853	37.7165669
59 O	28.4516952	39.3453963	35.6696152
60 H	30.6189976	39.1748701	36.8507643
61 H	30.9577104	37.8500083	38.0024395
62 H	30.2359980	40.6956874	38.8073977
63 H	32.9071653	39.3379421	38.3185548
64 H	35.4376680	36.0378183	35.8695374
65 H	33.1916886	41.3668702	40.4132579
66 H	30.9929956	41.0235749	41.1797721
67 H	32.5735207	37.5820774	41.8769375
68 H	29.2147991	42.0552439	45.0607189
69 H	31.7103785	37.6629169	46.7130733
70 H	30.5012012	38.5440526	47.6367702
71 H	32.0801367	42.0866841	38.2460296
72 H	28.4065904	34.1404244	38.3274015
73 H	32.0167186	29.4594133	39.5202762

In1-TS2'
QM(B1)/MM Energy = -4319.546702652320 a.u.
QM(B2)/MM Energy = -4322.560295000000 a.u.
QM(B3)/MM Energy = -4321.987590705621 a.u.

1 N	30.2815646	34.9362729	40.2555075
2 C	29.8282045	35.1017063	38.9601999
3 C	28.4050371	34.9942210	38.5313371
4 N	32.0717171	35.1617307	39.0271700
5 C	30.9696723	35.2424450	38.2098623
6 C	31.6291984	34.9697696	40.2583700
7 H	27.7225175	35.3755340	39.2996980
8 H	28.2134201	35.6413757	37.6676599
9 H	31.0597904	35.4111727	37.1404080
10 H	32.2441289	34.8431230	41.1447895
11 H	29.7223433	34.7976605	41.0977179
12 N	32.8999985	31.2234372	37.0588797
13 C	33.2309945	30.9156710	38.3633648
14 C	32.9782461	29.5771080	38.9903792
15 N	33.7632649	33.0684609	37.9175673
16 C	33.7682052	32.0781824	38.8762113
17 C	33.2347318	32.5181139	36.8347879
18 H	32.9646733	28.7794756	38.2336921
19 H	33.7932020	29.3262358	39.6860092
20 H	34.1602518	32.2507917	39.8776672
21 H	33.1066679	33.0195915	35.8764213
22 H	32.4985997	30.5644451	36.3811683
23 Fe	33.9973026	35.4808032	38.3724315
24 O	33.8039156	37.4785054	38.5721274
25 Cl	34.3633780	35.5826951	36.0404983
26 O	35.7846069	33.7506557	41.2788161
27 C	36.0183736	34.2308032	40.1728328
28 O	35.2934246	35.2097891	39.6970469
29 C	37.1371495	33.7218505	39.2787911
30 C	38.1555292	34.8128356	38.8984076
31 C	38.7993241	35.3803294	40.1797411
32 O	38.1956888	36.3315210	40.7344977
33 O	39.8281713	34.7970212	40.6048738
34 H	36.6671243	33.3104356	38.3681435
35 H	37.6450863	32.9049646	39.8122187
36 H	38.9264319	34.3653679	38.2526241
37 H	37.6414522	35.6135054	38.3454241
38 O	29.4658673	38.1614759	38.1612838
39 C	30.7623207	38.5175213	37.8254040
40 C	31.2144293	39.8360765	38.4510816
41 O	31.0272579	39.7848784	39.8911510
42 C	32.7121478	40.1132008	38.2729759
43 O	33.0449703	41.4949335	38.3307484
44 C	33.3147925	39.4510258	39.4959026
45 C	32.2760248	39.7995991	40.5371348
46 N	32.1871451	39.0492589	41.7755238
47 C	32.5453846	37.7598624	42.0450597
48 N	32.1945783	37.3625766	43.2527612
49 C	31.5481699	38.4494259	43.8145397
50 C	30.9283024	38.7043858	45.0661022
51 N	30.8812640	37.7939319	46.0830949
52 N	30.3305356	39.8967114	45.2447332

53 C	30.3595192	40.8115202	44.2615630
54 N	30.9729622	40.7109215	43.0819810
55 C	31.5252868	39.5112708	42.8975418
56 P	28.1690850	38.7819901	37.2214770
57 O	27.0669599	37.7743369	37.5372603
58 O	27.9427759	40.2067679	37.7680038
59 O	28.7169378	38.7247263	35.7700973
60 H	30.8863049	38.5984088	36.7297861
61 H	31.4258400	37.7192816	38.1942568
62 H	30.5942481	40.6581605	38.0604379
63 H	33.1047758	39.7180395	37.3234801
64 H	33.2154596	37.7119886	37.8392181
65 H	34.3752942	39.5593708	39.7248103
66 H	32.5198516	40.8366113	40.8575300
67 H	33.0825218	37.1697062	41.3024061
68 H	29.8239847	41.7495409	44.4524581
69 H	31.6671937	37.1428158	46.1886071
70 H	30.4084471	38.1181580	46.9320287
71 H	32.3026137	42.0703352	38.6548450
72 H	28.1258725	33.9540948	38.3631951
73 H	32.0290834	29.4976402	39.5203246

In1-2'-OH-dAMP
QM(B1)/MM Energy = -4319.654909325184 a.u.
QM(B2)/MM Energy = -4322.669084000000 a.u.
QM(B3)/MM Energy = -4322.080609336094 a.u.

1 N	30.7526299	35.0244883	40.1474158
2 C	30.2100792	35.1966171	38.8865486
3 C	28.7587273	35.1120213	38.5546199
4 N	32.4607000	35.2782035	38.7931232
5 C	31.2971639	35.3642850	38.0617769
6 C	32.1028816	35.0714280	40.0541159
7 H	28.1349425	35.5307388	39.3539703
8 H	28.5293384	35.7502227	37.6934715
9 H	31.3112431	35.5536528	36.9919812
10 H	32.7971321	34.9077584	40.8776578
11 H	30.2306633	34.8906555	41.0163806
12 N	32.9065315	30.7869276	36.9743544
13 C	33.2215692	30.5976681	38.3049281
14 C	32.9193954	29.3481578	39.0693360
15 N	33.7770212	32.6809599	37.6678621
16 C	33.7715481	31.7898031	38.7182896
17 C	33.2523284	32.0461573	36.6306487
18 H	32.8579868	28.4767985	38.4028326
19 H	33.7323270	29.1379401	39.7777828
20 H	34.1487330	32.0672231	39.7021431
21 H	33.1372628	32.4689738	35.6336596
22 H	32.5077177	30.0811927	36.3420437
23 Fe	34.2398999	34.7376894	37.8455319
24 O	33.4942370	38.5263452	39.9361376
25 Cl	34.4299455	35.3170059	35.6211773
26 O	35.3837524	33.8991871	41.0331258
27 C	36.0275167	34.2078658	40.0179130
28 O	35.6096192	35.0605808	39.1433471
29 C	37.3332411	33.5025237	39.6667929
30 C	38.4476490	34.4368295	39.1800294
31 C	38.9494861	35.2882122	40.3594157
32 O	38.1450582	36.1354752	40.8180466
33 O	40.0943577	35.0199278	40.8045096
34 H	37.0675896	32.7825818	38.8689664
35 H	37.6702815	32.9211632	40.5385473
36 H	39.2882316	33.8505871	38.7816347
37 H	38.0480498	35.0834482	38.3832557
38 O	29.5517682	38.1256960	38.0216940
39 C	30.7696750	38.7547644	37.8244314
40 C	31.0139924	39.8933670	38.8063002
41 O	30.8121289	39.3879778	40.1495686
42 C	32.4727846	40.4135165	38.7650360
43 O	32.6307989	41.8040410	38.6380628
44 C	33.0543087	39.8734637	40.0897904
45 C	31.7931379	39.9787548	40.9602599
46 N	31.8035283	39.3314232	42.2564118
47 C	32.3374224	38.1201290	42.6083608
48 N	32.1079433	37.7821441	43.8617927
49 C	31.3652564	38.8339863	44.3729674
50 C	30.8271033	39.1275389	45.6498416
51 N	31.0764352	38.3576566	46.7406892
52 N	30.0863231	40.2458433	45.7871268
53 C	29.9187152	41.0590391	44.7268544
54 N	30.4495160	40.9272184	43.5122187
55 C	31.1436583	39.7990145	43.3799119
56 P	28.1826173	38.7622623	37.2069911
57 O	27.1282941	37.7138738	37.5514967
58 O	27.9510425	40.1564714	37.8318086
59 O	28.6377091	38.7809089	35.7262950
60 H	30.8694084	39.1559390	36.8009587
61 H	31.5762385	38.0005721	37.9457155
62 H	30.2920151	40.7026893	38.6138241
63 H	33.0281177	39.9580275	37.9283718
64 H	32.7574876	37.9995420	39.5834149
65 H	33.9104188	40.4611341	40.4401164
66 H	31.5647483	41.0432061	41.1625551
67 H	32.9142995	37.5461244	41.8871521
68 H	29.2850746	41.9409676	44.8835561
69 H	31.6889855	37.5407775	46.6605298
70 H	30.4297515	38.4422004	47.5300745
71 H	31.8982933	42.3392976	39.0220114
72 H	28.4213988	34.0859992	38.4078027
73 H	31.9694347	29.3829859	39.6026546

In2-RC
QM(B1)/MM Energy = -4320.540932976467 a.u.
QM(B2)/MM Energy = -4323.560859000000 a.u.
QM(B3)/MM Energy = -4323.013657040619 a.u.

1 N 39.8013676 26.9932687 41.5361885
2 C 39.8181865 28.2509534 42.1093177
3 C 39.5996361 28.5483066 43.5533911
4 N 40.1983494 28.3985082 39.9091890
5 C 40.0706928 29.1159787 41.0725144
6 C 40.0316809 27.1216653 40.2128006
7 H 40.0329984 27.7719605 44.1983796
8 H 40.1109130 29.4889857 43.8014341
9 H 40.1620769 30.1969489 41.0992392
10 H 40.0825622 26.2897029 39.5149323
11 H 39.6487172 26.0946712 42.0050162
12 N 36.2786623 29.4674919 38.5866368
13 C 36.3294086 28.1748730 38.0999032
14 C 35.1745727 27.2263457 38.0977869
15 N 38.3494577 29.1578523 37.9441059
16 C 37.6326455 28.0077046 37.6880917
17 C 37.5025461 30.0177357 38.4862648
18 H 34.2331232 27.7360156 37.8425678
19 H 35.3343531 26.4697545 37.3182321
20 H 38.1040671 27.1458153 37.2237147
21 H 37.7459961 31.0222413 38.8183699
22 H 35.5084922 29.9498689 39.0528420
23 Fe 40.4571217 29.2344679 38.0272672
24 O 42.0770792 29.2578427 38.1313832
25 Cl 40.2581384 31.5378855 38.2076707
26 O 42.2481985 26.8233134 36.2072098
27 C 41.3413372 27.5595184 35.8738805
28 O 40.3571841 27.9253934 36.6954404
29 C 41.1831277 28.2064012 34.5037491
30 C 42.0658172 27.6885776 33.3715331
31 C 41.5968450 26.3006262 32.8757313
32 O 42.1288198 25.2948777 33.4287179
33 O 40.7122933 26.2656914 31.9947291
34 H 41.3607252 29.2835074 34.6858848
35 H 40.1188674 28.1330031 34.2229579
36 H 41.9971133 28.3960859 32.5306173
37 H 43.1112167 27.6382821 33.7103024
38 O 46.4149708 30.7513216 42.2422500
39 C 46.0051536 29.4572497 42.5619827
40 C 45.7602678 28.5691495 41.3305090
41 O 45.8300057 27.1781941 41.7450059
42 C 44.3664290 28.7283922 40.7163752
43 O 44.3762247 28.2424495 39.3772117
44 C 43.5531558 27.7792227 41.6068874
45 C 44.5339471 26.6171328 41.7906327
46 N 44.3949416 25.8712123 43.0388421
47 C 44.6422107 26.3445786 44.3123726
48 N 44.6373053 25.3980810 45.2224272
49 C 44.3930145 24.2360987 44.5224791
50 C 44.2811806 22.8811409 44.9198935
51 N 44.3536882 22.4873575 46.2049945
52 N 44.1240851 21.9581510 43.9499567
53 C 43.9656720 22.3540227 42.6775270
54 N 43.9876696 23.6006687 42.1968665
55 C 44.2337841 24.5062182 43.1575755
56 P 45.2672494 32.0203688 42.4395396
57 O 46.1510618 33.2625157 42.2955743
58 O 44.3009802 31.8137933 41.2553405
59 O 44.6349396 31.7610747 43.8230969
60 H 45.0942209 29.4847597 43.1851110
61 H 46.7750286 28.9572861 43.1805404
62 H 46.5453664 28.7450760 40.5777752
63 H 44.0431253 29.7824156 40.7753357
64 H 43.3123528 28.2545350 42.5699470
65 H 42.6161154 27.4631906 41.1316408
66 H 44.4131100 25.8701799 40.9915369
67 H 44.8249791 27.4028231 44.5056856
68 H 43.8070083 21.5539720 41.9458750
69 H 44.1179705 21.5310758 46.4511819
70 H 44.4154359 23.1759376 46.9510867
71 H 43.5094607 28.4681033 38.9774719
72 H 38.5508650 28.5998343 43.8457802
73 H 34.9941232 26.7278605 39.0501617

In2-TS1
QM(B1)/MM Energy = -4320.488883200316 a.u.
QM(B2)/MM Energy = -4323.504165000000 a.u.
QM(B3)/MM Energy = -4322.962305480488 a.u.

1 N 39.6921511 26.8512700 42.0861702
2 C 39.6884504 28.1639196 42.5234992
3 C 39.4507577 28.6010890 43.9272024
4 N 39.9002807 28.0873250 40.2982034
5 C 39.8257182 28.9205879 41.3861666
6 C 39.8181972 26.8504846 40.7466540
7 H 39.8831173 27.9093607 44.6616524
8 H 39.9329041 29.5751250 44.0886386
9 H 39.8726461 30.0016201 41.2951434
10 H 39.8844201 25.9538745 40.1404064
11 H 39.6128501 25.9904368 42.6288277
12 N 36.5144153 29.3722293 38.5810174
13 C 36.4466120 28.0710958 38.1226468
14 C 35.2073870 27.2333355 38.1224492
15 N 38.5607243 28.8447449 37.9769665
16 C 37.7365187 27.7706825 37.7347607
17 C 37.7937811 29.7926521 38.4824608
18 H 34.3111816 27.8280468 37.8881186
19 H 35.2885019 26.4771761 37.3297963
20 H 38.1202317 26.8499364 37.3002537
21 H 38.1303901 30.7760432 38.7985705
22 H 35.7854147 29.9289161 39.0355321
23 Fe 40.7464466 28.6027893 38.4403324
24 O 42.2744324 27.9550952 39.1331482
25 Cl 40.9958335 30.8925052 38.3919273
26 O 42.9137228 27.2484560 36.1953335
27 C 41.7752022 27.5966127 35.9277799

28 O 40.7987816 27.6445978 36.8224335
29 C 41.3159520 28.1407713 34.5816100
30 C 42.0897803 27.7146462 33.3359164
31 C 41.7278298 26.2759519 32.9092810
32 O 42.1852556 25.3491745 33.6347290
33 O 40.9972690 26.1197088 31.9038224
34 H 41.3717843 29.2384000 34.7197835
35 H 40.2418122 27.9184886 34.4794498
36 H 41.8520606 28.4034104 32.5114608
37 H 43.1698928 27.7703010 33.5472296
38 O 46.4500030 30.7088952 42.2577365
39 C 46.0178531 29.4136843 42.5453823
40 C 45.9397438 28.5316230 41.2906525
41 O 45.8317762 27.1343450 41.6763067
42 C 44.7035313 28.7750344 40.4350357
43 O 44.9206160 28.1945753 39.1509320
44 C 43.6736316 27.9266236 41.1721133
45 C 44.4851626 26.6896442 41.5664051
46 N 44.1459412 26.0225746 42.8267894
47 C 44.3502203 26.5576815 44.0878618
48 N 44.4209766 25.6515684 45.0329539
49 C 44.2823788 24.4450588 44.3768551
50 C 44.3447178 23.1015156 44.8228046
51 N 44.5104054 22.7681306 46.1156380
52 N 44.2719344 22.1272857 43.8925415
53 C 44.0555463 22.4585490 42.6098625
54 N 43.9600084 23.6833018 42.0846225
55 C 44.1093843 24.6486912 43.0037098
56 P 45.2948750 31.9793375 42.4343936
57 O 46.1758754 33.2230204 42.2941416
58 O 44.3362390 31.7682648 41.2443323
59 O 44.6538466 31.7211126 43.8138798
60 H 45.0357304 29.4359164 43.0517457
61 H 46.7093005 28.9245178 43.2610355
62 H 46.8551430 28.6512597 40.6894998
63 H 44.4362336 29.8441696 40.4055595
64 H 43.1210475 28.4284153 41.9787417
65 H 42.7750679 27.7637848 40.2509446
66 H 44.4112052 25.9063728 40.7974127
67 H 44.4587944 27.6309049 44.2445992
68 H 43.9626145 21.6204136 41.9099838
69 H 44.4660162 21.7947169 46.3915250
70 H 44.5383194 23.4825043 46.8389939
71 H 44.0156776 28.0243193 38.7992432
72 H 38.3863550 28.6432823 44.1581431
73 H 34.9971740 26.7307574 39.0665324

In2-IM1
QM(B1)/MM Energy = -4320.535155656551 a.u.
QM(B2)/MM Energy = -4323.555294000000 a.u.
QM(B3)/MM Energy = -4323.006459902070 a.u.

1 N 39.8505059 26.9971357 41.5406848
2 C 39.8473666 28.2531991 42.1224887
3 C 39.6253908 28.5259731 43.5700752
4 N 40.2436410 28.4158566 39.9257025
5 C 40.0975239 29.1270025 41.0939998
6 C 40.0900435 27.1326057 40.2224999
7 H 40.0509951 27.7262729 44.1919788
8 H 40.1467115 29.4546149 43.8403020
9 H 40.1804122 30.2085167 41.1251324
10 H 40.1636982 26.3075865 39.5183710
11 H 39.7197857 26.0970360 42.0149132
12 N 36.2912106 29.4898521 38.5071742
13 C 36.3427016 28.1870283 38.0474450
14 C 35.1930124 27.2322632 38.0823227
15 N 38.3464933 29.1944935 37.7976001
16 C 37.6346214 28.0291521 37.5953967
17 C 37.5038224 30.0526983 38.3507034
18 H 34.2469432 27.7344077 37.8286476
19 H 35.3458664 26.4645326 37.3122205
20 H 38.1058648 27.1566039 37.1504631
21 H 37.7489905 31.0632784 38.6655543
22 H 35.5345457 29.9652825 39.0024214
23 Fe 40.5292429 29.2207652 38.0473242
24 O 42.4043643 29.4454561 37.9966868
25 Cl 40.3143552 31.5872642 38.3242236
26 O 42.3644243 26.6046912 36.1985038
27 C 41.4492408 27.3803737 36.0073275
28 O 40.5582533 27.6729636 36.9503417
29 C 41.2148761 28.1439436 34.7092860
30 C 42.0310549 27.7101002 33.4911279
31 C 41.5561031 26.3499826 32.9219394
32 O 42.1071964 25.3190393 33.4053152
33 O 40.6564196 26.3627094 32.0571406
34 H 41.4268329 29.2006279 34.9649243
35 H 40.1350729 28.1036592 34.4847310
36 H 41.9102636 28.4712331 32.7043677
37 H 43.0949374 27.6480545 33.7662482
38 O 46.3697907 30.7006121 42.2324337
39 C 45.9565722 29.4073600 42.5631414
40 C 45.7632408 28.5156066 41.3279464
41 O 45.8484685 27.1156834 41.7257199
42 C 44.3809323 28.6696760 40.6843785
43 O 44.4246834 28.2423697 39.3172948
44 C 43.6105148 27.6539352 41.4693703
45 C 44.5582543 26.5414277 41.7641252
46 N 44.3617290 25.8475624 43.0391336
47 C 44.5574315 26.3560489 44.3069618
48 N 44.5381268 25.4297340 45.2381799
49 C 44.3301712 24.2475714 44.5586819
50 C 44.2413692 22.8969895 44.9772601
51 N 44.3037479 22.5247174 46.2686342
52 N 44.1168723 21.9536784 44.0211121
53 C 43.9795127 22.3217457 42.7381470
54 N 44.0003347 23.5587042 42.2344898

55 C	44.2097631	24.4853081	43.1844703
56 P	45.2260065	31.9783190	42.3857370
57 O	46.1166838	33.2156324	42.2356859
58 O	44.2753755	31.7572823	41.1875756
59 O	44.5760814	31.7517479	43.7651906
60 H	45.0188608	29.4317494	43.1480733
61 H	46.7096607	28.9224394	43.2145466
62 H	46.5637753	28.7080584	40.5969679
63 H	44.0282062	29.7121509	40.7793938
64 H	42.5273030	27.5401830	41.4866981
65 H	42.6834119	30.3671657	38.0978832
66 H	44.4821778	25.7373443	41.0080989
67 H	44.7165447	27.4222357	44.4764551
68 H	43.8428368	21.5057262	42.0191907
69 H	44.0970847	21.5647495	46.5257098
70 H	44.3717668	23.2234344	47.0043250
71 H	43.5993879	28.5631519	38.8687037
72 H	38.5765629	28.5838427	43.8610708
73 H	35.0188778	26.7442694	39.0412838

In2-TS2
QM(B1)/MM Energy = -4320.493756819794 a.u.
QM(B2)/MM Energy = -4323.513101000000 a.u.
QM(B3)/MM Energy = -4322.967750887678 a.u.

1 N	39.5937755	26.8719429	41.6359977
2 C	39.4661639	28.1585928	42.1244095
3 C	39.4309313	28.5153717	43.5720169
4 N	39.3862964	28.1688320	39.8868197
5 C	39.3289493	28.9512571	41.0146145
6 C	39.5495654	26.9219921	40.2943501
7 H	39.9214891	27.7450255	44.1833059
8 H	39.9749440	29.4565130	43.7441031
9 H	39.2265714	30.0312846	40.9639040
10 H	39.7223024	26.0662023	39.6482966
11 H	39.7181029	26.0080752	42.1719201
12 N	36.2256004	29.5102018	38.4470304
13 C	36.2309816	28.2180498	37.9531187
14 C	35.0913934	27.2544808	38.0384131
15 N	38.2047515	29.2321939	37.5316593
16 C	37.4723949	28.0772656	37.3736002
17 C	37.4236258	30.0754626	38.1864782
18 H	34.1361874	27.7491164	37.8054417
19 H	35.2296625	26.4887692	37.2636406
20 H	37.8987499	27.2034349	36.8867614
21 H	37.6985561	31.0744588	38.5234485
22 H	35.5179746	29.9734035	39.0220317
23 Fe	40.3098640	28.9841370	38.1813824
24 O	40.7092154	30.7756814	38.4270799
25 Cl	42.2630289	28.2490086	39.7202777
26 O	42.9338295	27.4090109	36.2196779
27 C	41.7878183	27.7355089	35.9421276
28 O	40.8238689	27.7724464	36.8423891
29 C	41.3326697	28.2347998	34.5753177
30 C	42.1027255	27.7639568	33.3403535
31 C	41.7231238	26.3222250	32.9341568
32 O	42.1901428	25.3959272	33.6542019
33 O	40.9730229	26.1632639	31.9433525
34 H	41.3955926	29.3367777	34.6644117
35 H	40.2574839	28.0150441	34.4765915
36 H	41.8773804	28.4380395	32.5003200
37 H	43.1829533	27.8093033	33.5519215
38 O	46.5037854	30.8548471	42.2953489
39 C	46.2122416	29.5515962	42.7062980
40 C	46.1843170	28.5887734	41.5089769
41 O	46.0519140	27.2095896	41.9646776
42 C	44.9805233	28.8054538	40.6114136
43 O	45.2230354	28.1530832	39.3572486
44 C	43.9524158	28.0301164	41.3860115
45 C	44.6961838	26.7718249	41.7786757
46 N	44.2818556	26.0752627	42.9921708
47 C	44.4323920	26.5491128	44.2839209
48 N	44.3859389	25.6047353	45.1915351
49 C	44.2162965	24.4354444	44.4789305
50 C	44.1750416	23.0730926	44.8681464
51 N	44.2477517	22.6752025	46.1518636
52 N	44.0930449	22.1443477	43.8932933
53 C	43.9707871	22.5389155	42.6169397
54 N	43.9811443	23.7871434	42.1397391
55 C	44.1413984	24.7022342	43.1074703
56 P	45.2668201	32.0578918	42.3910545
57 O	46.0808987	33.3334976	42.1546112
58 O	44.2889820	31.7121112	41.2467109
59 O	44.6708867	31.8720109	43.8000539
60 H	45.2481071	29.5197890	43.2472988
61 H	46.9699600	29.1967415	43.4313482
62 H	47.1117822	28.6738971	40.9210200
63 H	44.7278878	29.8709772	40.5135012
64 H	43.3551551	28.5415382	42.1427232
65 H	41.6311779	30.9297486	38.6840355
66 H	44.6590407	26.0138581	40.9799930
67 H	44.5932445	27.6065512	44.4903850
68 H	43.8635238	21.7372523	41.8774654
69 H	44.1261866	21.6960245	46.3828401
70 H	44.3301105	23.3524379	46.9055509
71 H	44.3301517	27.9228355	39.0348677
72 H	38.4067429	28.5854690	43.9383386
73 H	34.9301150	26.7595743	38.9960790

In2-2'-Cl-dAMP
QM(B1)/MM Energy = -4320.555507211437 a.u.
QM(B2)/MM Energy = -4323.582774000000 a.u.
QM(B3)/MM Energy = -4323.023418757972 a.u.

1 N	39.4667853	26.9807429	41.4809412
2 C	39.5138181	28.2155038	42.0915095

3 C 39.4025986 28.4682259 43.5557494
4 N 39.7438327 28.4483329 39.8729626
5 C 39.6713250 29.1153162 41.0682191
6 C 39.6170022 27.1623972 40.1495552
7 H 39.8499631 27.6560397 44.1464721
8 H 39.9551568 29.3861047 43.8079960
9 H 39.7358851 30.1957780 41.1227217
10 H 39.6683998 26.3495766 39.4288992
11 H 39.3851824 26.0649073 41.9296775
12 N 36.1822356 29.5853607 38.4652566
13 C 36.2452968 28.3049805 37.9484414
14 C 35.1408753 27.2970900 38.0113392
15 N 38.1619512 29.4275537 37.5186226
16 C 37.4867472 28.2380177 37.3509227
17 C 37.3475367 30.2156543 38.2007792
18 H 34.1760129 27.7599110 37.7518969
19 H 35.3218303 26.5342049 37.2421059
20 H 37.9440224 27.3956452 36.8364891
21 H 37.5929953 31.2098368 38.5722525
22 H 35.4634641 30.0023401 39.0601826
23 Fe 40.2497359 29.4796873 38.1130222
24 O 40.5220156 31.2338700 38.8230934
25 Cl 42.5059444 27.0192883 41.1614471
26 O 43.1412361 27.4207890 36.4060725
27 C 42.0022821 27.8538175 36.2124323
28 O 41.1602314 28.0566799 37.1785292
29 C 41.4743061 28.2571899 34.8312986
30 C 42.2283998 27.7906195 33.5811048
31 C 41.8760972 26.3450779 33.1568197
32 O 42.4092826 25.4168172 33.8225395
33 O 41.0848759 26.1896096 32.1945210
34 H 41.4504395 29.3635901 34.8621714
35 H 40.4134989 27.9572835 34.7839991
36 H 41.9794334 28.4618369 32.7447635
37 H 43.3105792 27.8528789 33.7757461
38 O 46.5529129 30.8920832 42.2966908
39 C 46.2628882 29.6194987 42.7807967
40 C 46.1416831 28.5852195 41.6527150
41 O 46.2531160 27.2501587 42.2473387
42 C 44.8018960 28.5935184 40.9139190
43 O 44.9621766 27.9631971 39.6442311
44 C 44.0027336 27.6940112 41.8571425
45 C 45.0175310 26.5875239 42.1518798
46 N 44.7698207 25.8180417 43.3541450
47 C 44.9709374 26.2027346 44.6655694
48 N 44.7680060 25.2258528 45.5184310
49 C 44.4226257 24.1395542 44.7407668
50 C 44.1299372 22.7883741 45.0444298
51 N 44.1186140 22.3060696 46.3012571
52 N 43.8662696 21.9575442 44.0195668
53 C 43.8306740 22.4353893 42.7677914
54 N 44.0760323 23.6845035 42.3614016
55 C 44.3920792 24.4897442 43.3863574
56 P 45.3037269 32.0863162 42.3355422
57 O 46.1052682 33.3655428 42.0806329
58 O 44.3650606 31.6751527 41.1839632
59 O 44.6892431 31.9334113 43.7411129
60 H 45.3423006 29.6336058 43.3930179
61 H 47.0667264 29.2741683 43.4579760
62 H 46.9626051 28.7190907 40.9318020
63 H 44.4062618 29.6234321 40.8379483
64 H 43.7178049 28.2409178 42.7655054
65 H 40.8383949 31.8850048 38.1836929
66 H 45.0040636 25.8620929 41.3219450
67 H 45.2640867 27.2195025 44.9279540
68 H 43.5807663 21.7081930 41.9879267
69 H 43.8420122 21.3450766 46.4776088
70 H 44.4260110 22.8634010 47.0895397
71 H 44.0823088 27.6753171 39.3414720
72 H 38.3662593 28.5402612 43.8857175
73 H 34.9639052 26.7962150 38.9631128

In2-TS2'
QM(B1)/MM Energy = -4320.497075206624 a.u.
QM(B2)/MM Energy = -4323.510822000000 a.u.
QM(B3)/MM Energy = -4322.965861072580 a.u.

1 N 39.7720087 26.8393355 42.0007058
2 C 39.7434000 28.1450600 42.4550547
3 C 39.5197751 28.5469375 43.8711313
4 N 39.9183848 28.1030326 40.2269194
5 C 39.8452615 28.9206339 41.3279491
6 C 39.8778552 26.8578218 40.6600345
7 H 39.9413801 27.8141726 44.5708116
8 H 40.0209289 29.5045810 44.0668997
9 H 39.8758615 30.0037622 41.2480677
10 H 39.9527327 25.9735118 40.0360161
11 H 39.7531402 25.9737966 42.5452375
12 N 36.5314046 29.4109697 38.4559921
13 C 36.4849036 28.0922691 38.0471261
14 C 35.2568706 27.2378563 38.0869199
15 N 38.5726789 28.9209680 37.7925441
16 C 37.7696981 27.8145037 37.6253703
17 C 37.7933634 29.8634248 38.2936099
18 H 34.3534779 27.8174621 37.8414565
19 H 35.3394596 26.4638279 37.3117282
20 H 38.1673045 26.8853083 37.2218869
21 H 38.1165483 30.8636944 38.5695031
22 H 35.8070437 29.9534560 38.9339698
23 Fe 40.7408803 28.6931057 38.4309172
24 O 42.4937040 28.5724653 39.2946710
25 Cl 40.8710762 31.0610828 38.3971464
26 O 43.0735638 27.2365404 36.2426337
27 C 41.8873238 27.4952922 36.0847187
28 O 41.0135310 27.3927397 37.0735377
29 C 41.2930927 28.0644599 34.8020979

30 C	41.9928385	27.7391807	33.4792424
31 C	41.6897787	26.3044821	32.9935246
32 O	42.1683379	25.3689420	33.6936348
33 O	40.9892070	26.1569623	31.9657842
34 H	41.2968426	29.1596222	34.9738560
35 H	40.2284746	27.7834614	34.7691205
36 H	41.6650667	28.4519445	32.7073680
37 H	43.0820247	27.8460728	33.6145799
38 O	46.4421023	30.6518747	42.2269905
39 C	46.0226011	29.3555437	42.5408184
40 C	45.9924837	28.4477433	41.3018915
41 O	45.7631578	27.0655840	41.6794953
42 C	44.8383145	28.7593842	40.3707653
43 O	45.0646794	28.1048560	39.1196727
44 C	43.7146958	28.0367937	41.0641485
45 C	44.3755182	26.7392412	41.5126752
46 N	43.9400526	26.1077063	42.7650458
47 C	44.0928711	26.6694609	44.0249456
48 N	44.2010606	25.7844847	44.9850247
49 C	44.1440654	24.5605752	44.3462654
50 C	44.3027845	23.2304868	44.8117127
51 N	44.5021412	22.9313520	46.1073477
52 N	44.2913321	22.2364328	43.8985061
53 C	44.0657214	22.5358910	42.6100708
54 N	43.9118555	23.7455539	42.0656175
55 C	43.9833525	24.7342171	42.9687552
56 P	45.2724924	31.9145960	42.3600911
57 O	46.1385810	33.1653757	42.1922882
58 O	44.3132582	31.6563505	41.1740533
59 O	44.6254876	31.6951805	43.7410133
60 H	45.0241070	29.3757510	43.0123764
61 H	46.6963306	28.8983353	43.2928079
62 H	46.9505461	28.5056353	40.7623187
63 H	44.6748692	29.8448853	40.2959982
64 H	42.9894720	28.5458605	41.7009020
65 H	42.7044626	29.4813923	39.5662193
66 H	44.2819003	25.9503688	40.7502335
67 H	44.1409231	27.7493341	44.1683787
68 H	44.0239972	21.6846154	41.9211451
69 H	44.5613493	21.9647104	46.4018187
70 H	44.5117675	23.6612168	46.8155502
71 H	44.1512771	28.0006368	38.7706914
72 H	38.4574604	28.6042440	44.1083456
73 H	35.0484995	26.7536425	39.0409564

In2-2'-OH-dAMP
QM(B1)/MM Energy = -4320.607111115708 a.u.
QM(B2)/MM Energy = -4323.623922000000 a.u.
QM(B3)/MM Energy = -4323.061341607020 a.u.

1 N	39.6689907	26.9462190	41.4128113
2 C	39.7442332	28.1965242	41.9906482
3 C	39.5962302	28.4762610	43.4465130
4 N	40.0149726	28.3528163	39.7655286
5 C	39.9576944	29.0631702	40.9449581
6 C	39.8364622	27.0782299	40.0817954
7 H	40.0303898	27.6681824	44.0510941
8 H	40.1439016	29.3961082	43.6940186
9 H	40.0544631	30.1442184	40.9667636
10 H	39.8780986	26.2429118	39.3872481
11 H	39.5864177	26.0478496	41.8992654
12 N	36.1078554	29.4502342	38.3007073
13 C	36.1484168	28.1226638	37.9230453
14 C	35.0181554	27.1572545	38.0515298
15 N	38.1224045	29.1213776	37.4908627
16 C	37.4134760	27.9441686	37.4098788
17 C	37.3073600	30.0094353	38.0369742
18 H	34.0553733	27.6430922	37.8367903
19 H	35.1418814	26.3790620	37.2884984
20 H	37.8587817	27.0374179	37.0062646
21 H	37.5678129	31.0348561	38.2911044
22 H	35.3838161	29.9389816	38.8295520
23 Fe	40.2048127	29.1878160	37.8400995
24 O	42.7813754	27.2515903	40.7611202
25 Cl	40.4983643	31.4468134	38.2365541
26 O	43.1819536	27.3415993	36.2523890
27 C	42.0248292	27.7048149	36.0520696
28 O	41.1371078	27.7397774	37.0122421
29 C	41.5185807	28.2409468	34.7126404
30 C	42.2020592	27.7634869	33.4283268
31 C	41.7789203	26.3332956	33.0213325
32 O	42.2261472	25.3946097	33.7369266
33 O	41.0254128	26.1938406	32.0295466
34 H	41.6288945	29.3395429	34.8027855
35 H	40.4322806	28.0573675	34.6661116
36 H	41.9479101	28.4524991	32.6085158
37 H	43.2932739	27.7816076	33.5795302
38 O	46.5310984	30.7806302	42.3069529
39 C	46.1800643	29.4888858	42.6917258
40 C	46.0880833	28.5209772	41.5035044
41 O	46.0759529	27.1598147	42.0262388
42 C	44.8113651	28.6329886	40.6844145
43 O	44.9872075	27.9943687	39.4187150
44 C	43.8515277	27.7685886	41.5151941
45 C	44.7827262	26.6134930	41.9193181
46 N	44.4645835	25.9177110	43.1551346
47 C	44.6649445	26.3796542	44.4423549
48 N	44.5711949	25.4325797	45.3452286
49 C	44.3131256	24.2816586	44.6297868
50 C	44.1575389	22.9260605	45.0110751
51 N	44.1930346	22.5212155	46.2941583
52 N	43.9985658	22.0166827	44.0289278
53 C	43.9039093	22.4255347	42.7542236
54 N	43.9977405	23.6724971	42.2832199
55 C	44.2314262	24.5605051	43.2614609
56 P	45.3119579	31.9967948	42.4164148

57	O	46.1335855	33.2727100	42.2169429
58	O	44.3668115	31.6655921	41.2421291
59	O	44.6794930	31.7784849	43.8059499
60	H	45.2267513	29.4937311	43.2483909
61	H	46.9331066	29.0775929	43.3927337
62	H	46.9674587	28.6378268	40.8512875
63	H	44.4906112	29.6867802	40.5972260
64	H	43.4951149	28.3240621	42.3991748
65	H	42.1384306	27.9600908	40.6013099
66	H	44.7305545	25.8442842	41.1327958
67	H	44.8888942	27.4276161	44.6473813
68	H	43.7381163	21.6373281	42.0109016
69	H	43.9558452	21.5631679	46.5335933
70	H	44.3042376	23.1967371	47.0452814
71	H	44.1477294	27.5381106	39.2226878
72	H	38.5606802	28.5539086	43.7776839
73	H	34.8980565	26.6890126	39.0284602
